# An Optimized Method for LC–MS-Based Quantification of Endogenous Organic Acids: Metabolic Perturbations in Pancreatic Cancer

**DOI:** 10.3390/ijms25115901

**Published:** 2024-05-28

**Authors:** Shreyans K. Jain, Shivani Bansal, Sunil Bansal, Baldev Singh, William Klotzbier, Khyati Y. Mehta, Amrita K. Cheema

**Affiliations:** 1 Department of Oncology, Lombardi Comprehensive Cancer Centre, Georgetown University Medical Center, E-415, New Research Building, 3900 Reservoir Road NW, Washington, DC 20057, USA; shreyansrj@gmail.com (S.K.J.); sm3451@georgetown.edu (S.B.); sb1886@georgetown.edu (S.B.); bs1126@georgetown.edu (B.S.); wek11@georgetown.edu (W.K.); kym8@georgetown.edu (K.Y.M.); 2 Department of Biochemistry, Molecular and Cellular Biology, Georgetown University Medical Centre, Washington, DC 20057, USA

**Keywords:** CCMs, 4-Chloro-o-phenylenediamine, LC–MRM, quantification, pancreatic cancer

## Abstract

Accurate and reliable quantification of organic acids with carboxylic acid functional groups in complex biological samples remains a major analytical challenge in clinical chemistry. Issues such as spontaneous decarboxylation during ionization, poor chromatographic resolution, and retention on a reverse-phase column hinder sensitivity, specificity, and reproducibility in multiple-reaction monitoring (MRM)-based LC–MS assays. We report a targeted metabolomics method using phenylenediamine derivatization for quantifying carboxylic acid-containing metabolites (CCMs). This method achieves accurate and sensitive quantification in various biological matrices, with recovery rates from 90% to 105% and CVs ≤ 10%. It shows linearity from 0.1 ng/mL to 10 µg/mL with linear regression coefficients of 0.99 and LODs as low as 0.01 ng/mL. The library included a wide variety of structurally variant CCMs such as amino acids/conjugates, short- to medium-chain organic acids, di/tri-carboxylic acids/conjugates, fatty acids, and some ring-containing CCMs. Comparing CCM profiles of pancreatic cancer cells to normal pancreatic cells identified potential biomarkers and their correlation with key metabolic pathways. This method enables sensitive, specific, and high-throughput quantification of CCMs from small samples, supporting a wide range of applications in basic, clinical, and translational research.

## 1. Introduction

Carboxylic acid is among the most abundant functional groups in biologically important small molecules, particularly relevant in central carbon energy metabolism such as the tricarboxylic acid (TCA) cycle, fatty acid and cholesterol metabolism, etc. Of ∼5000 endogenous human metabolites, about 65% contain at least one carboxylic acid group [1], and their abundance can vary in abnormal physiological states. For example, glycolytic carbon flux results in increased intracellular levels of lactic acid in cancer [2,3], while fumaric and succinic acids have been reported as potential biomarkers of renal carcinoma [4]. Methyl malonic acid has been reported as a potential biomarker of vitamin B12 deficiency, while α-ketoglutaric and pyruvic acid levels in urine are considered to be potential biomarkers for type II diabetes and liver cancer [5,6]. Short-chain fatty acids (SCFAs) (fewer than six carbon atoms) are known to play crucial roles in human health and disease [7]. SCFAs influence systemic metabolism by modulating glucose and lipid metabolism, enhancing insulin sensitivity, and reducing fat accumulation [8]. SCFAs modulate immune responses by influencing the production of cytokines and the differentiation of T cells [9]. Alterations in SCFA production are linked to gut disorders, including IBD, irritable bowel syndrome (IBS), metabolic disorders such as obesity, type 2 diabetes, colorectal cancer, and cardiovascular diseases [10]. Emerging research suggests that SCFAs may influence brain function and behavior through the gut–brain axis, potentially impacting conditions like autism and depression [11].

Analyzing carboxyl-containing metabolites (CCMs) using mass spectrometry faces challenges due to instability and poor resolution. Current methods like UPLC coupled with multiple-reaction monitoring (MRM) enhance sensitivity but struggle with ionization efficiency and structural diversity [12]. In addition, spontaneous decarboxylation caused by changes in temperature and pH during sample processing could lead to lower measured levels of CCMs, ultimately compromising the sensitivity of the assay [12]. Structurally, the lack of a functional group that could help in ionization in ESI-MS is the main cause of low detection potential/sensitivity for this class of metabolites. Hence, chemical labelling/derivatization with small molecular weight compounds to improve CCMs’ ease of ionization could yield desired sensitivity for their quantification, especially for matrices where samples volumes are limited. Recently, a number of RP LC–MS/MS methodologies have been introduced for assessing carboxylic acids (CAs) [13,14,15,16,17]. These techniques utilize derivatization of the carboxylic group, which serves to decrease the polarity of the analyte, facilitating RP-HPLC separation while simultaneously enhancing compound ionization. Various derivatization agents have been employed, such as aniline [13,18], 3-nitrophenylhydrazine (3-NPH) [17,19], and dansylhydrazine (DnsHz) [14,20], in conjunction with 1-ethyl-3-(3-dimethylaminopropyl)carbodiimide (EDC). Despite the advantages of derivatization, several uncertainties persist, ranging from the completeness of the derivatization reaction, particularly in the presence of a matrix, to the selectivity of the chosen labeling reagent and the stability of the derived compounds or harsh reaction conditions that are not favorable for biological samples. In summary, there is a critical need to develop a high throughput method for the accurate and reliable quantification of such an important class of metabolites, in biological matrices, which may tolerate aqueous media as present in most of the biological matrices (without sacrificing sensitivity of the method) to augment clinical chemistry-based applications. This work presents a phenylenediamine-based chemical derivatization method that has been applied to the quantitative LC–MS/MS analyses of carboxylic acid sub-metabolome in a variety of biological matrices. Benzimidazole-based ionizable groups were purposely introduced to the molecules of interest, wherein the presence of nitrogen atoms helps to improve ionization efficiency and, subsequently, the detection sensitivity of the derivatized CCMs. The method was validated in commonly employed biological matrices such as urine, plasma, serum, cells, tissue, and saliva, with very low sample volumes.

**Figure 1:**
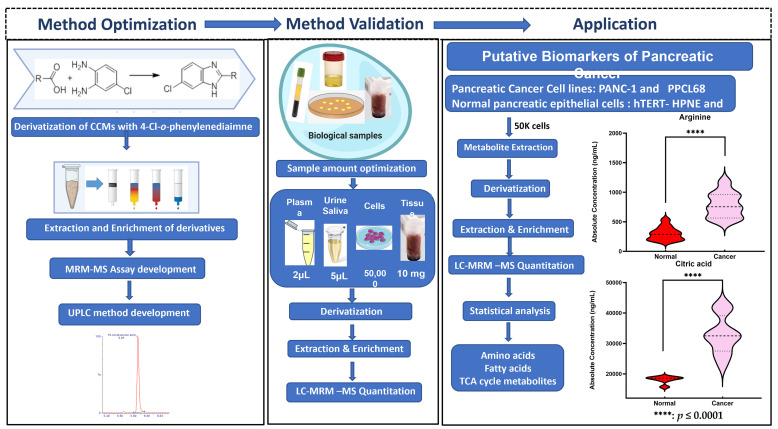
Acidomics: Quantification of CCMs by LC–MRM-MS.

To our knowledge, this is the first report that provides a highly accurate, sensitive, and reliable assay for the analyses of small-, medium-, and long-chain CCMs belonging to different classes of compounds that are integral to endogenous metabolism and important as potential clinical biomarkers. The workflow for the derivatization protocol for the quantitation of CCMs is illustrated in Figure 1. The developed analytical method was thereafter validated with biological samples using pancreatic cancer and normal pancreatic epithelial cell samples as an example of a complex metabolomic system. Pancreatic ductal adenocarcinoma (PDAC) is a lethal malignancy that aggressively invades the adjacent vasculatures and quickly spreads to far-flung areas by the time it is clinically detected. Although complete surgical resection remains the only curative treatment, fewer than 20% of patients are candidates for surgery at the time of presentation. Hence, there is a critical need to identify diagnostic biomarkers with potential clinical utility in this pathology. In this context, metabolomics could be a powerful tool to search for new robust biomarkers. Comparative metabolomic profiling for the assessment of differential CCM abundance was performed from two pancreatic cancer cell lines (PANC-1 and PPCL68, *n* = biological replicates each) and two normal pancreatic epithelial cells (HPDE and hTERT-HPNE, control, *n* = 6 biological replicates each) cell lines. Several CCMs were observed to exhibit altered levels in cancer cells, including TCA cycle intermediates, amino acids, fatty acids, etc. The dysregulation corresponds to pathways such as energy and protein metabolism and fatty acid biosynthesis, and these are hallmarks of pancreatic and other cancer types. These findings offer an information-rich matrix for discovering novel candidate biomarkers with diagnostic or prognostic potential in PDAC and are well supported by several literature reports. A pictorial representation of workflow is shown in Supplementary Figure S1.

## 2. Results

**Figure 2:**
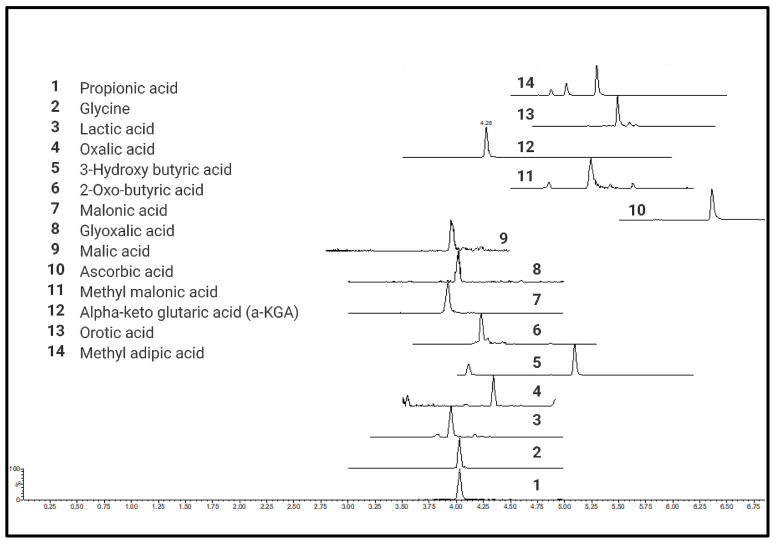
UPLC-MRM-MS profiles of standard mixtures of carboxylic acid derivatives.

A total of 76 different CCMs including TCA metabolites, short-chain organic acids (e.g., pyruvic acid), short-chain fatty acids (e.g., propionic acid), hydroxyl fatty acids (e.g., hydroxybutyric acid), heterocyclic amino acids (e.g., 4-OH proline), and long-chain mono- (palmitic acid) and dicarboxylic acids (octadecane dioic acid) were tested for quantitative analysis (details provided in Supplementary Table S1) using current protocol in six different biological matrices (Supplementary Table S2). Chromatograms and calibration curves for all the quantified metabolites are provided in Supplementary Figures S2 and S3, respectively. All derivatized CCMs showed symmetrical peak shapes, and baseline separations were obtained for most of the derivatives. Peak separation was achieved within a short time of 4 to 10 min, making the method ideal for high throughput analysis. UPLC-MRM-MS profiles of the standard mixtures of derivatized CCMs are shown in Figure 2.

### 2.1. MRM Protocol Optimization

**Figure 3:**
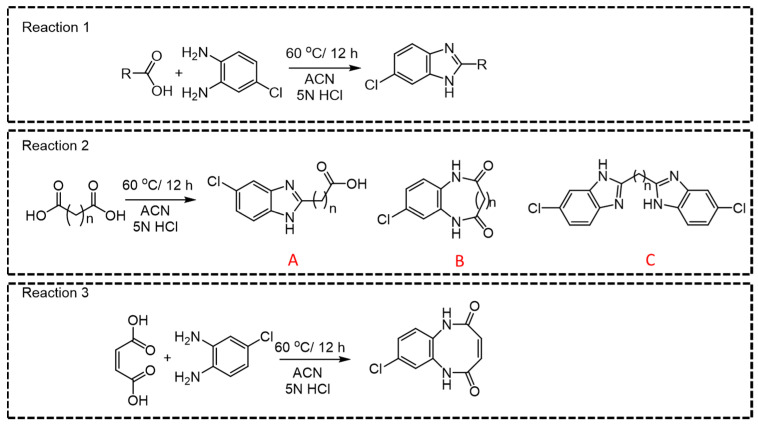
General reaction for derivatization of mono- and dicarboxylic acids.

Among all CCMs studied, for the parent ions where a linear derivative was formed with mono- or dicarboxylic acids, *m*/*z* 166.06 was observed as the most intense daughter ion (Q3) (Figure 3, Reactions 1, 2A, and 2C). In the case of dicarboxylic acids like fumaric acid, oxalic acid, malic acid, succinic acid, malonic acid, methylmalonic acid, glutaric acid, adipic acid, 2-hydroxy glutaric acid, 2,2-dimethylglutaric acid, 3-methyl adipic acid, and dodecanedioic acid, two kinds of products, linear (A or C) and cyclic derivatives (B), are possible (Figure 3, Reaction 2) depending upon the number of carboxylic acid groups participating in the derivatization reaction. For example, linear derivative A (Q1 > Q3; 225.195 > 166.01) was observed as the major product for succinic acid. However, linear derivative C (Q1 > Q3; 353.14 > 180.01) was the preferred product with methylmalonic acid. Due to enhanced lipophilicity, the derivatized product of methyl malonic acid eluted at relatively higher retention time (5.24 min) compared to that of succinic acid (4.05 min). Previous reports on methylmalonic acid quantification have cited issues either with the identification of Q1 > Q3 ions, the interference of succinic acid in chromatographic resolution, or sensitivity.

For small-chain acids, such as maleic acid and oxalic acid, cyclic derivatives (Figure 3, Reaction 3) were observed as the major product, which is possible with flat planer structures. Moreover, it was also possible to distinguish between maleic acid and fumaric acid based upon the difference in their major derivative that was formed (Supplementary Table S1, entry 10 and 11) that resulted in unique Q1 > Q3 pairs. For malic acid, along with the linear major product (*m*/*z* 240.6790 [M + H]+ Figure 3, Reaction 2A), we also detected another two major peaks (*m*/*z* 245.1729 and 351.1878). Perhaps malic acid dehydrates immediately into maleic acid and yields the cyclic derivative (*m*/*z* 245.1729 [M + Na]+), while *m*/*z* 351.2517 [M + Na]+ could be the parent ion where both carboxylic acid groups react during the derivatization reaction. The observed *m*/*z* (Q1; 301.05) for the derivatized ascorbic acid is suggestive of the lactone-ring hydrolysis under the optimized reaction conditions (Supplementary Table S1, entry 25) [21]. The derivatized product of citric acid was observed as unstable and immediately converted to a metastable parent ion. Observance of the [M + H]+ ion peak (511.021) indicates that the derivatization reaction occurring at all three carboxylic acid groups quickly converted to 493.074 [M + H-H_2_O]+, indicative of the respective product for cis-aconitic acid. For quantification purposes, we developed a specific MRM pair as (Q1 > Q3; 281.288 > 166.06) for cis-aconitic acid (Supplementary Table S1, entry 39). The parent ion at 237.259 was reflective of a linear derivative from one carboxylic acid group, which may be indicative of citric acid degradation to citraconic acid/itaconic acid (Supplementary Table S1, entries 39 and 40) [22]. While we also observed 511.021 > 248.9438 and 493.074 > 338.9858 MRM pairs, the transition 237.259 > 166.115 was selected for quantification of citric acid, and the quantification results are the sum of citric, iso-citric, itaconic, and citraconic acids. 

### 2.2. Assay Development

The developed LC–MRM methods were evaluated for various quantification parameters such as sensitivity (LOD, S/N > 3), lower limits of quantitation (LLOQ, S/N > 10), linearity range, S/N ratios, linear regression coefficients (r2) from the calibrators of each derivatized CCM, etc., as listed in Supplementary Table S3. The linearity of the standard curve ranges from 3 × 10^3^ to 3 × 10^4^ fold of the LOD, and linear regression coefficients (r2) were 0.9990, in most cases. The calibration curves and related details for each quantified metabolite are provided in Supplementary Figure S3. To our knowledge, these are the lowest reported LLOQs for all the CCMs, including 0.01 ng/mL for lactic acid, 2-hydroxy glutaric acid, and 3-hydroxybutyric acid and 1.0 ng/mL for pyruvic acid and α-ketoglutaric acid, with improved S/N ratios. Quantitative results of all the metabolites from six different biological matrices studied herein are compiled in Supplementary Table S2. For assessing intra- and inter-day variability, the samples (*n* = 6), including the standard solutions, were analyzed for six consecutive days. While we observed consistent peak areas for all the CCMs, the intra- and inter-day precision (RSDs < 0.5% and <2%) were well indicative of metabolite stability and assay reproducibility. To study the matrix effects and have an estimation of percent metabolite recovery for each matrix, known amounts of derivatized standards were spiked in all six different matrices studied herein at three different concentrations. Thereafter, the same protocol as defined for biological samples was followed, except that 50 µL of methanol (solvent alone) was added instead of a derivatizing solution. The recovery percentages calculated as [(mean observed concentration/spiked concentration) × 100%] were observed to be ranging from 90% to 105% (Supplementary Table S4).

### 2.3. Method Validation

**Figure 4:**
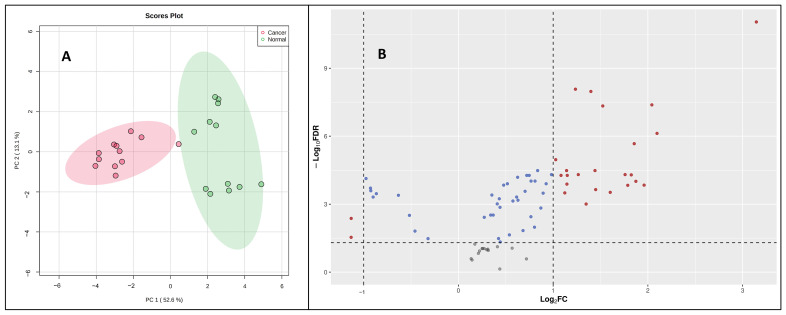
(**A**) A two-dimensional PCA plot showing separation of pancreatic cancer cells (PANC-1 and PPCL68) and normal pancreatic epithelial cells (HPDE and HPNE) based on CCM profile samples. (**B**) Volcano plot showing dysregulated metabolites in pancreatic cancer cells (PANC-1 and PPCL68). All annotated metabolites have an FDR adjusted significant *p*-value (<0.05) comparing PDAC to normal pancreatic epithelial cells. Each dot on the plot represents a metabolite delineated in this study. Statistically significant changes is based on log2 fold change (X-axis) and *p*-value (-log10(FDR), Y-axis). The red dots represent significantly dysregulated metabolites.

**Figure 5:**
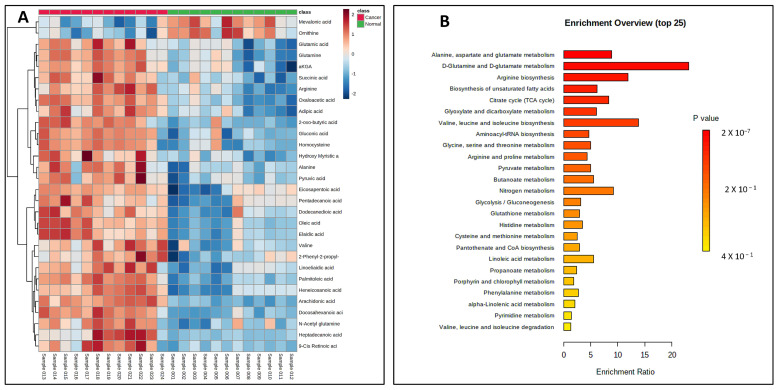
(**A**) Heat map showing significant dysregulations of various CCMs in pancreatic cancer cell lines (PANC-1 and PPCL68 combined for analysis) when compared to normal pancreatic epithelial cells (HPDE and HPNE combined for analysis). Color corresponds to log2 fold change, with red indicating upregulation and blue indicating downregulation, compared to normal cells. (**B**) Summary of pathway enrichment analysis shows significantly affected pathways. The most significant *p*-values are in red, while the least significant are in yellow.

Once validated across six different biological matrices, we investigated the utility of this methodology to search the potential biomarkers of pancreatic cancer. The metabolomic investigation of cellular content is of fundamental biological interest to unravel metabolic processes [23,24]. For this, two pancreatic cancer cell lines (PANC-1, PPCL68) and two normal pancreatic epithelial cells (HPDE and HPNE) were grown to a density of 50,000 cells/mL in twelve-well costar plates, as explained previously. The cell samples (24) for all four cell lines (*n* = 6) were processed with the derivatization reaction and followed by LC–MS data acquisition. Principal component analysis (PCA) demonstrated distinct CCM profiles of cancerous cells when compared to normal pancreatic epithelial cells (Figure 4A). A volcano plot further helped visualize the significant dysregulations for CCMs in cancerous cells (Figure 4B). Univariate analysis was performed to compare the CCM profiles of cancerous cells to normal cells, revealing altered levels of several CCMs in cancer cells. Overall, 25 CCMs were identified as significantly altered (0.5 > FC > 2, FDR adjusted *p*-value < 0.05) across the two cancer cell lines when compared to normal pancreatic epithelial cells (Supplementary Table S5). In addition to the majorly impacted energy and protein metabolism and fatty acid biosynthesis, pathway analyses revealed perturbations in the glyoxylate and dicarboxylate metabolism, aminoacyl-tRNA biosynthesis, pyruvate metabolism, butanoate metabolism, etc., in pancreatic cancer cells (Figure 5). The media isolated from cancerous and normal pancreatic epithelial cells were also collected, and their secretory CCMs profiles (Supplementary Table S6) were compared with control media. The upregulation of γ-amino butyric acid and pyruvic acid were common for both cancerous and normal pancreatic epithelial cell media compared to the control. On the other hand, media from normal pancreatic epithelial cell lines showed elevated levels of some amino acids, organic acids, and fatty acids compared to control media. These sets of metabolites discussed herein could potentially be a biomarker group for pancreatic cancer to distinguish between pancreatic cancer and healthy individuals and increase the likelihood of providing a reliable diagnosis of PDAC.

## 3. Discussion

Spontaneous degradation/decarboxylation during ionization, poor chromatographic resolution, and retention on a reverse-phase column are several factors that limit the sensitivity, specificity, and reproducibility during the downstream LC–MS analysis of CCMs in biological matrices. There is an urgent and unmet need to develop a reliable method for quantitative analysis of CCMs from biological samples that would provide adequate sensitivity for quantitation in the physiological range. The discovery of a universal derivatizing reagent to label the carboxylic acid sub-metabolome that could impart stability to the CCMs from any degradation could be the way forward. For the reliable quantification of CCMs, we used a phenylenediamine-based (4-Cl-o-PD) derivatization reaction that would generate benzimidazoles (N-containing/basic heterocyclic moiety), which ionize/protonate readily in ESI positive (+) mode, in contrast to free CCMs, and thus can be detected with ultra-high sensitivity in conjunction with reverse-phase chromatography [25,26]. The benzimidazole derivatives with enhanced lipophilicity (higher log *p* values calculated with ChemDraw 18.1, PerkinElmer, Shelton, CT, USA) as imparted by phenylenediamine-based derivatization of CCMs showed enhanced resolution and optimal retention on a reverse-phase column, especially in complex biological matrices like plasma or cell extracts. We describe an optimized method for the analysis of this functionally important class of compounds from an array of biological matrices, hitherto unreported. 

Central carbon metabolism is the most significantly perturbed pathway in cancer, in addition to fatty-acid biosynthesis and amino acid metabolism, which include CCMs [27,28]. Some of the CCMs, including tricarboxylic acid (TCA) cycle intermediates, amino acids (AAs), fatty acids/oxylipins, short-chain organic acids, etc., have been reported to be dysregulated in pancreatic and other cancer types [29,30]. We observed upregulation for several amino acids (AAs), which is in accordance with our previous cancer metabolomics studies in pancreatic cancer [28,31,32,33]. For example, arginine and glutamate metabolism were found to be upregulated in pancreatic cancer cells. Arginine and its metabolites regulate the proliferation, growth, autophagy, apoptosis, and metastasis of cancer cells [34]. The pathogenesis of pancreatic cancer is reported to cause perturbations in the Gln-Glu pathway [32,35]. Glutamate has been shown to promote pancreatic cancer cell growth, invasion, and migration [36]. In cancer cells, glutamine is the major amino acid that replenishes and drives the tricarboxylic acid (TCA) cycle for continuous energy production in mitochondria [37]. Elevated homocysteine levels could be due to lower betaine abundance. Betaine as a methyl donor supports methionine homeostasis and thus maintains proper pancreatic function and cellular replication [38]. Elevated γ—Aminobutyric acid (GABA) could play important roles in PDAC development and progression growth through overexpressing the GABAA receptor π-subunit [39]. Histamine, another upregulated metabolite, is an important mediator of cell proliferation in different types of cancers and thus is critical for tumor development and progression [40]. Histamine is a versatile molecule with diverse functions in the body, playing roles in both normal physiological processes and pathological conditions such as allergies and inflammation [41]. Observations in our study, well supported by several literature reports, point to the potential use of AAs as biomarkers for PDAC detection. 

Free fatty acids (FFAs) are vital endogenous molecules for cellular energy metabolism, and their altered levels could be attributed to disorders associated with fatty-acid beta oxidation and perturbed carnitine shuttle, indicative of disrupted mitochondrial function. Polyunsaturated fatty acids (PUFAs) such as arachidonic acid, EPA, and DHA, form precursors to various PGLs, TXs, and LTs, which are pro-inflammatory in nature, cause immunosuppression, and promote tumor cell proliferation [42,43,44]. 

## 4. Conclusions

In summary, we demonstrate the applicability of a novel analytical method that can be used for the quantification of a wide range of clinically important CCMs, including amino acids, short- to medium-chain organic/fatty acids, keto acids, hydroxyl acids, and di/tri-carboxylic acids. This method enables effective quantification with small sample size and allows distinctive quantification of structural isomers such as methylmalonic acid and succinic acid. To our knowledge, this is the first report wherein 4-Cl-o-PD was used for chemical derivatization of CCMs followed by their quantification by LC-MRM-based mass spectrometry. This method was optimized for targeted analysis of human plasma, serum, urine, saliva, tissue, and cell extracts. Since these matrices are widely used in clinical chemistry-based biomedical research, we anticipate broad applicability of this high-throughput methodology for bio-medical researchers. This method may be extended to other CCMs like bile acids, prostaglandins, etc. Clinical metabolomics has made quick progress to identify potential metabolic biomarkers for PDAC; however, there are still important informative gaps to be filled to increase the robustness and the reliability of these outcomes. We believe that a phenylenediamine derivatization-based LC–MS approach would be imperative in future studies on any biological sample for comprehensive metabolome profiling to investigate the subtle physiological changes in various diseased states and biomarker discovery. 

## 5. Materials and Methods

### 5.1. Chemicals and Reagents

LC–MS-grade acetonitrile, methanol, water and other organic solvents, and formic acid were purchased from Optima, Fisher Scientific. HPLC grade ethyl acetate was purchased from Thermo Fisher Scientific. The compounds 4-Chloro-o-phenylenediamine (4-Cl-o-PD) and HCl were obtained from Sigma-Aldrich (Burlington, MA, USA). All the standards were purchased from Sigma-Aldrich or CDN Isotopes (Pointe-Claire, QC, Canada). The reagents were stored at −20 °C and vacuum-dried in a desiccator over anhydrous phosphorus pentoxide under a vacuum of 30 mmHg, at 4 °C for 12 h, before use. NIST plasma was purchased as Standard Reference Materials (SRM) from the National Institute of Standards and Technology (NIST), a physical sciences laboratory, Rockville, MD, USA. All the biological reagents for the cell culture were purchased from Gibco Fisher Scientific (Waltham, MA, USA).

Detailed information on the collection and handling of biospecimens, cell culture, metabolite extraction from cells, and metabolite extraction from growth media has been provided in the Supplementary Information.

### 5.2. Derivatization Protocol for Standards, Internal Standards, and Biological Samples

A literature search revealed that o-phenylenediamines (o-PD) react readily with most CCMs to produce 2-substituted benzimidazoles, usually in high yields [21]. This involves the condensation of o-PD with a carboxylic acid group in the presence of concentrated hydrochloric acid. However, most of the currently reported phenylenediamine-based derivatization methods need non-aqueous/moisture-free conditions, which are incompatible with downstream LC–MS analysis of biological matrices [22]. The derivatization methodology reported here can be performed in almost all polar protic (including water) and aprotic solvents commonly used in research laboratories. The amount of reagent was selected based on a preliminary experiment. Standard compounds and internal standards (ISs) (1 mmol each) were subjected to derivatization with 4-Cl-o-PD (1.1 mmol for monocarboxylic acids and 2.1 mmol for dicarboxylic acids). For example, 0.9 mg of lactic acid (monocarboxylic acid) would need 1.50 mg of 4-Cl-o-PD, while 1.19 mg of succinic acid (dicarboxylic acid) would need 3.0 mg of 4-Cl-o-PD. For all biological matrices and cell samples, 50 µg of 4-Cl-o-PD was found as enough for the optimized sample amounts. Addition of 10 µL of 5N HCl drastically improved the product yield at 60 °C for 12 h in methanol as general reaction conditions for the derivatization method. Sample processing, extraction, and derivatization steps were performed simultaneously to avoid loss of analyte content due to spontaneous decarboxylation of CCMs under normal laboratory conditions. We used 2 µL of plasma, serum and NIST plasma, and 5 µL of urine and saliva, 10 mg of tissue, and 50,000 cells for the developed derivatization assay. Each biological sample (matrix) was analyzed in duplicate over a 6-day period. Without sacrificing sensitivity, we observed excellent S/N ratios for derivatized CCMs for the optimized sample amounts taken, based upon preliminary experiments. The derivation reaction for standards, ISs, and biological samples were done as per the standard operating procedure (SOP) as mentioned in Supplemental Information.

### 5.3. MRM Development and LC–MS Conditions

The derivatized standards (1 µg/mL in water: MeOH (1:1)) as enriched by cartridge fractionation were infused onto Xevo-TQS (Waters Corporation, Milford, MA, USA), and the sample cone voltage and collision energies were optimized for the analyte to obtain maximum ion intensity for parent and daughter ions using the “IntelliStart” feature of MassLynx V4.2 software (Waters Corporation, Milford, MA, USA). The most intense precursor and fragment ion pair (Q1 > Q3) of each analyte was selected for MRM-based quantitation. A complete list of CCMs, the structures of the parent molecules, and their corresponding benzimidazole derivatives along with MRM transitions (Q1 > Q3) and other parameters are provided in Supplementary Table S1. 

The derivatized CCMs were resolved on BEH C-18, 1.7 µM, 2.1 mm, 100 mm column (35 °C) using an Acquity UPLC system coupled with a Xevo-triple quadrupole mass spectrometer (Xevo-TQS, Waters Corporation, Milford, MA, USA) operating in the ESI + MRM mode. Further details on the LC conditions, data acquisition, and data processing have been provided in the Supplementary Information.

### 5.4. Statistical Analysis

Binary comparisons were done using unpaired *t*-tests, and fold changes were calculated to identify dysregulations in CCM levels for pancreatic cancer cells, compared to normal pancreatic epithelial cells. Similarly, we also compared the CCM profiles of media collected during cell harvest to those of control media. All analyses were performed using MetaboAnalyst 5.0. Figures were created using R and ChemDraw 18.1

## Data Availability

No new data were created.

## Supplementary Materials

The following supporting information can be downloaded at https://www.mdpi.com/article/10.3390/ijms25115901/s1.
