# Supplementary material for: An Optimized Method for LC–MS-Based Quantification of Endogenous Organic Acids: Metabolic Perturbations in Pancreatic Cancer
Source: Int J Mol Sci. 2024 May 28;25(11):5901. doi: 10.3390/ijms25115901 (PMC11172734; doi:10.3390/ijms25115901)
Supplement: Supplementary file 1 [file ijms-25-05901-s001.zip › 20240522_Supplementary Information Jain et al.pdf]

Supplementary Information

An optimized method for LC-MS based quantification of endogenous organic acids: metabolic perturbations in pancreatic cancer

[$]Shreyans K. Jain[a], [$]Shivani Bansal[a], [$]Sunil Bansal[a], Baldev Singh[a], William Klotzbier[a], Khyati Y. Mehta[a], Amrita K. Cheema[a,b]*

[a]Department of Oncology, Lombardi Comprehensive Cancer Centre, Georgetown University Medical Centre, Washington, DC, United States of America

[b]Department of Biochemistry Molecular and Cellular Biology, Georgetown University Medical Centre, Washington, DC, United States of America

$: Both authors contributed equally.

*Corresponding Author

Professor, Department of Oncology, Lombardi Comprehensive Cancer Center,

E-415, New Research Building

3900 Reservoir Road NW, Washington D.C. 20057

Georgetown University Medical Center

E-mail: akc27@georgetown.edu

Phone: (202)-687-2756 Fax: (202)-687-8860

**CONTENTS**	**PAGE NO.**
1. **List of acronyms**	3
2. **Supplementary Figure S1.** Pictorial presentation of workflow	5
3. **Supplementary Table S1.** List of CCMs, the structures of parent molecules and their corresponding benzimidazole derivatives along with MRM Transitions and other parameters.	6-14
4. **Supplementary Table S2.** Quantitation results of 76 CCMs in human body fluids, tissue, and cell lines (in ng/mL).	15-17
5. **Supplementary Figure S2.** LC Chromatogram of derivatized CCMs	18-93
6. **Supplementary Figure S3.** Standard curve of derivatized CCMs	94-171
7. **Supplementary Table S3.** List of metabolites with respective MRM transitions, retention time (RT), limit of detection (LOD), linearity range, single to noise (S/N) ratio and r^2 values.	172-174
8. **Supplementary Table S4.** %Recovery of 76 CCMs in human body fluids, tissue, and cell lines (in ng/mL).	174-176
9. **Supplementary Table S5.** List of dysregulated CCMs (carbon containing metabolites) in pancreatic cancer cell lines (PANC-1 and PPCL68 individually as well as combined) when compared to normal pancreatic epithelial cells (HPDE and HPNE individually as well as combined).	177
10. **Supplementary Table S6.** List of dysregulated CCMs (carbon containing metabolites) in the media isolated from pancreatic cancer cell lines (PANC-1 and PPCL68) and normal pancreatic epithelial cells (HPDE and HPNE) when compared to the control media used for the growth of each mentioned cell line, respectively.	178

1. List of acronyms

1. MRM- multiple reaction monitoring
2. TCA- tricarboxylic acid
3. CA- carboxylic acid
4. CV- coefficient of variation
5. 4-Cl-OPD- 4-Chloro-*o*-phenylenediamine
6. OPD- *o*-phenylenediamine
7. NIST- National Institute of Standards and Technology
8. CID- collision induced dissociation
9. RSD- relative standard deviation
10. RT- retention time
11. LOD- limit of detection
12. S/N ratio- signal to noise ratio
13. CCM-carboxylic containing metabolite

2. Materials and Methods

1.1 Collection and Handling of biospecimens

Samples (plasma, serum, saliva, urine, and liver tissue) were made available through the Indivumed repository at the MedStar-Georgetown University hospital protocols under approved institutional review board (IRB) protocols. On the day of analysis, samples were thawed on ice and immediately refrozen (-80 °C) after an appropriate aliquot was taken for analysis.

1.2 Cell culture

hTERT-HPNE and HPDE-H6c7 cells were grown in keratinocyte serum free media (K-SFM, 1 ml), containing K-SFM supplements, epithermal growth factor (EGF) and bovine pituitary extract (BPE) and 1% penicillin-streptomycin. PANC-1 cells were cultured in modified-improved minimum essential medium (modified-IMEM, 1 ml) harboring 2 mM L-glutamine, 1% penicillin-streptomycin and 10% heat- inactivated fetal bovine serum (Hi-FBS) and PPCL68 cells were cultured in advanced minimum essential medium (advanced-MEM, 1 ml) having 2 mM L-glutamine, 1% penicillin-streptomycin and 10% heat-inactivated fetal bovine serum (Hi-FBS). For each cell lines, cells were seeded in twelve well plates and allowed to grow for 24 h under humidified environment (CO_2 incubator) at 37 °C with 5% CO_2.

1.3 Metabolite extraction from cells

Culture plates were taken out from the incubator and kept on ice. Media (800 µl) was collected from each well and rest was discarded. After washing with PBS, 300 µl of extraction solvent (100% methanol) was added to cells in each well. Cells were scraped after 5 min and the suspension was transferred to 1.5 ml of micro centrifuge tubes. The tubes were centrifuged at 13,000 rpm at 4 °C for 20 min. The supernatant was collected, dried under nitrogen flow, and stored at -80 °C till further processing.

1.4 Metabolite extraction from growth media

Collected media (800 µl) was centrifuged at 13,000 rpm at 4 °C for 20 min to remove cellular debris. Media (500 µl) was collected into fresh micro centrifuge tube, lyophilized, and stored at -80 °C till further processing. Media without cells were incubated and processed under similar conditions and were taken as control.

1.5 LC conditions and data acquisition

The mobile phase was composed of water with 0.2% formic acid (solvent A) and acetonitrile with 0.2% formic acid (solvent B). A gradient elution was used over 15 min with a flow rate of 0.4 mL/min: 0-2 min 100% A; 2-8 min 0-100% B; 8-12 min 100% B;12-14 min 100-0% B and 14-15 min 0% B.

We used six to eight-point calibration curve, generated by serially diluting (in extraction buffer) the main stock (10 µg/ml) in a range of 0.001 ng/ml to 5 µg/ml. The quality and reproducibility of LC-MS data was ensured using several measures. The sample queue was randomized in prior, and solvent blanks were injected between sets of samples to monitor and ensure there was no sample-to-sample carry-over. QC standard was injected periodically (after every 10 sample injections) to monitor shifts in signal intensities and retention time as measures of reproducibility and to ensure high quality LC-MS data. The coefficient of variation for QC standard was well within permissible limits (<5%). The data were processed using TargetLynx 4.1. The metabolite abundance was calculated by normalizing the peak areas of metabolites to the peak areas of respective internal standard (IS) used for a particular metabolite. Herein, we used 12 internal standards such as valine-d_8, methionine-^{13}C, alanine-^{13}C, octanoic acid-^{13}C, succinic acid-d_6, arachidonic acid-d_8, docosahexenoic acid-d_5, 4-aminobutyric acid-4,4-d_2, pyruvic acid-$^{13}C_3$, malic acid-d_3 and α-keto glutaric acid-d_4.

Supplementary Figure S1. Pictorial presentation of workflow

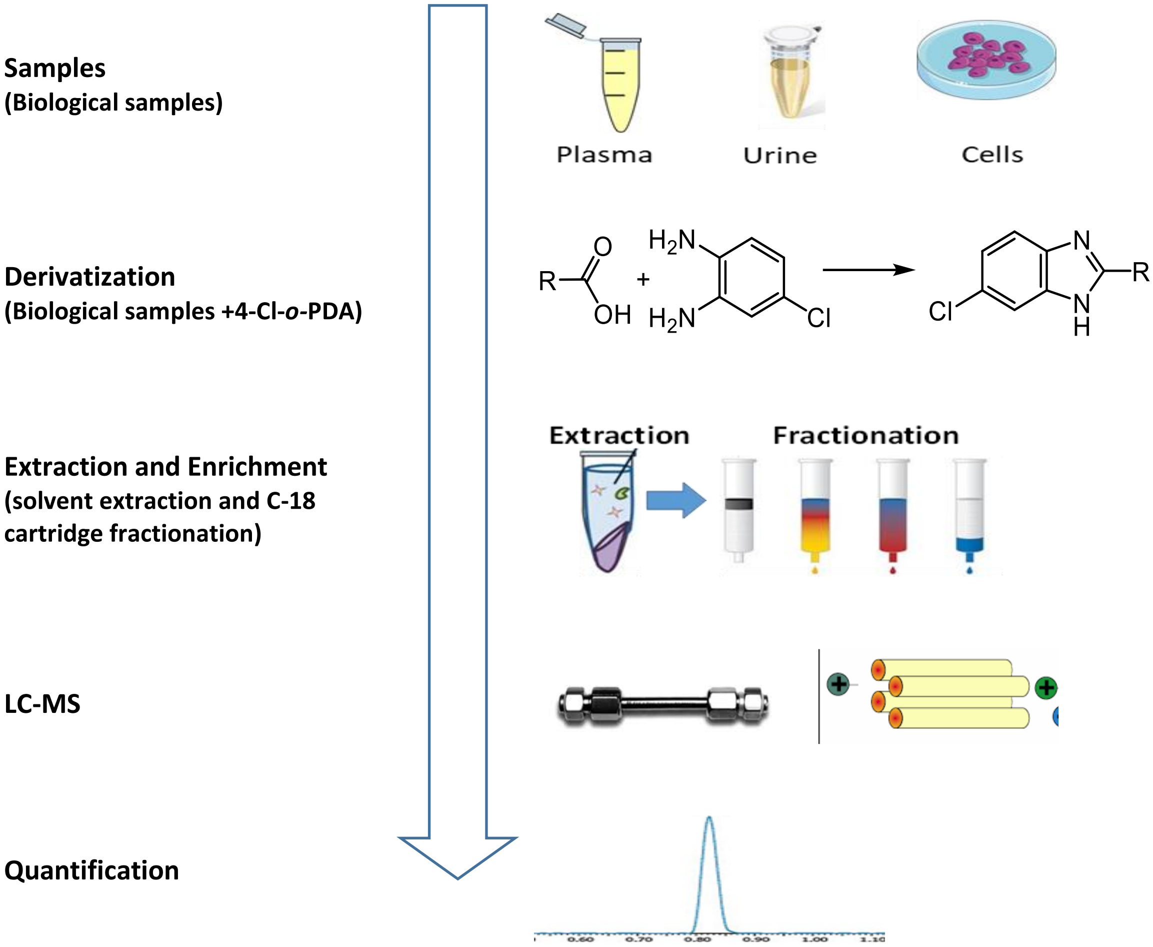

Supplementary Table S1. List of CCMs, the structures of parent molecules and their corresponding benzimidazole derivatives along with MRM Transitions and other parameters.

S. No.	Name	Compound (m/z)	Derivatized parent (m/z)	MRM transition Q1>Q3 (CV, CE)	RT
1	Glyceric acid	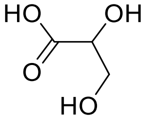	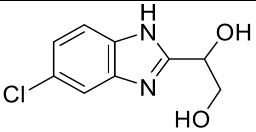	213.26>196.01 (20, 20)	0.71
2	Glyoxalic acid	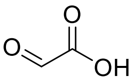	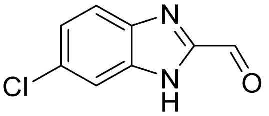	180.913>152 (20, 20)	4.02
3	Propionic acid	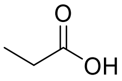	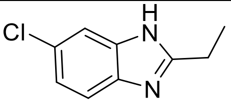	181.259>166.06 (2, 26)	4.03
4	Pyruvic acid	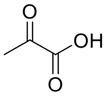	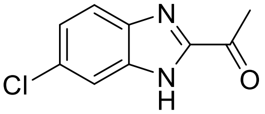	194.929>179 (34, 26)	4.16
5	Alanine	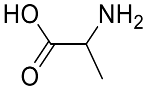	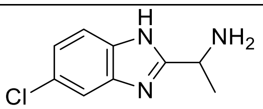	196.273>181.01 (20, 20)	4.16
6	2-Oxo butyric acid	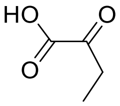	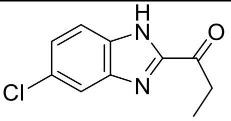	209.270>194.035 (20, 20)	4.23
7	Malonic acid	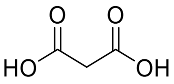	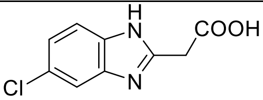	211.243>152.07 (20, 30)	3.92
8	Serine	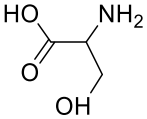	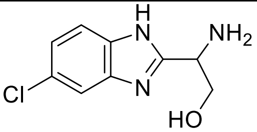	212.270>165.062	3.92
9	4-Methyl-2-oxo-pentanoic acid	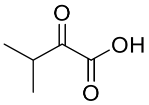	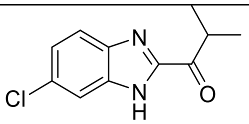	223.216>180 (58, 24)	4.82
10	Fumaric acid	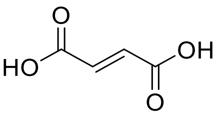	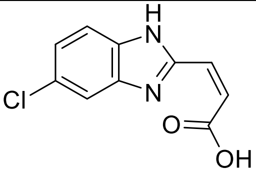	223.252>165.061 (20, 40)	4.82
11	Maleic acid	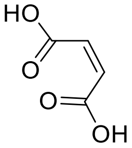	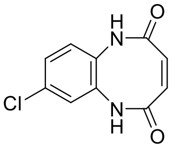	245.179>182.998 (44, 26)	3.61

12	Valine	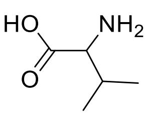	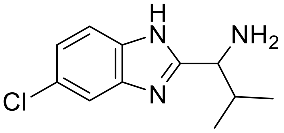	224.33>165.03 (30, 25)	4.76
13	Succinic acid	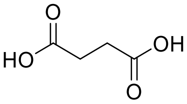	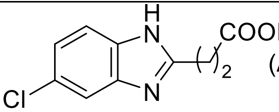 (A) 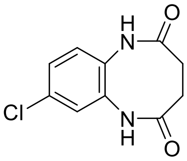 (B)	225.195>166.01 (20, 25)	4.05
14	Ureidopropionic acid	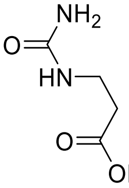	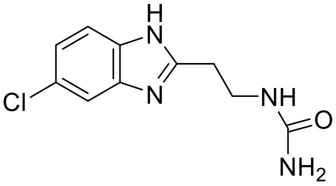	238.966>166.036 (20, 12)	8.71
15	Oxaloacetic acid	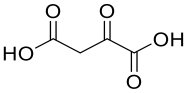	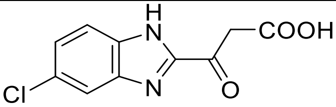	239.238>152.03 (20, 20)	6.16
16	Aspartic acid	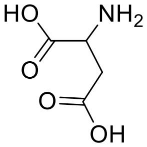	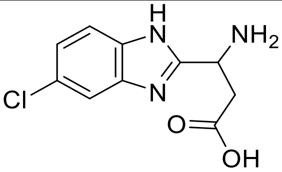	240.36>165.061 (20, 30)	4.19
17	Malic acid	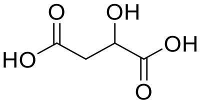	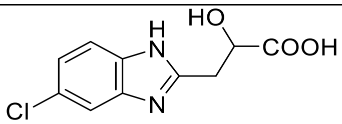	240.679>179.032 (20, 15)	3.96
18	Homocysteine	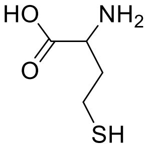	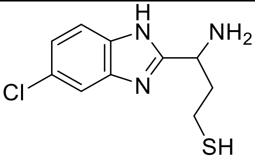	242>209 (20, 15)	4.19
19	α-Keto glutaric acid	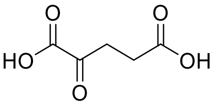	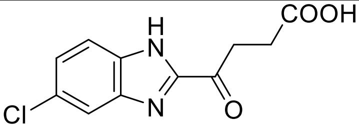	252.871>152.01 (30, 40)	4.28
20	Glutamine	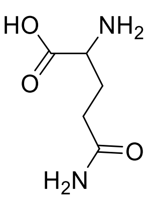	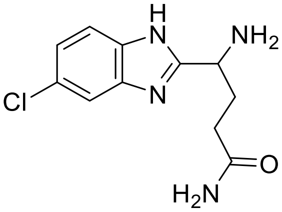	253.33>152.04 (20, 40)	4.28

21	Methionine	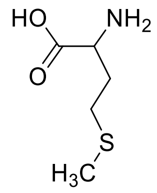	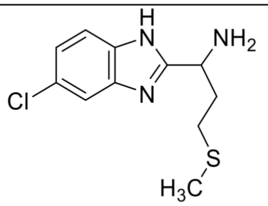	256.39>152.06 (20, 50)	5.26
22	Orotic acid	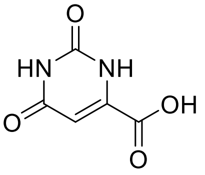	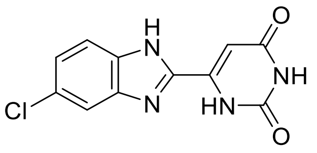	263.5>165.062 (30, 25)	5.49
23	3-Methyl adipic acid	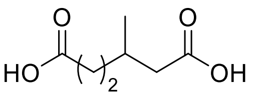	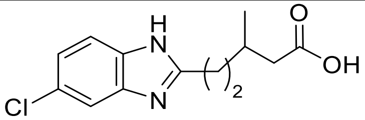	267.114>166.061 (30, 25)	5.3
24	N-Acetyl glutamine	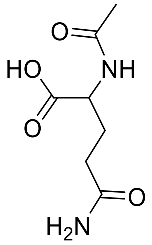	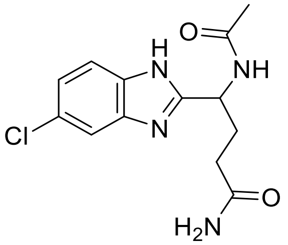	296.346>165.061 (20, 32)	4.79
25	Ascorbic acid	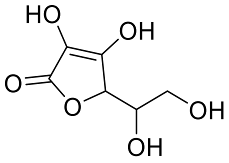	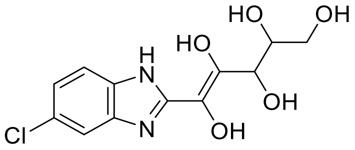	301.05>266.01 (40, 35)	6.36
26	3-Nitrotyrosine	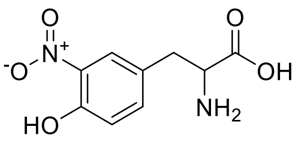	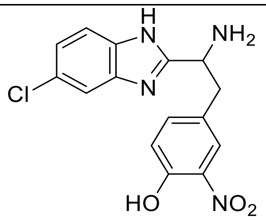	333.37>181.03 (10, 30)	4.85
27	Methylmalonic acid	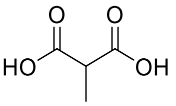	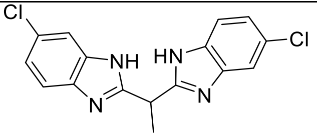 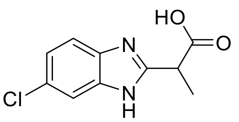	353.14>180.011 (30, 60)	5.24
28	Docosatetraenoic acid	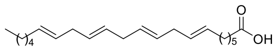	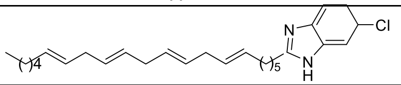	439.68>179.035 (20, 46)	7.43
29	Palmitic acid	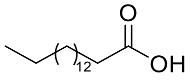	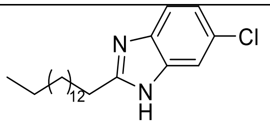	363.345>179.132 (28, 40)	7.31

30	Glycine	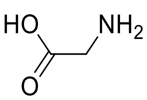	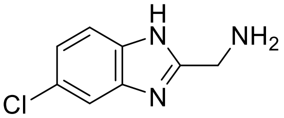	182.251>166.060 (20, 30)	4.02
31	Sarcosine	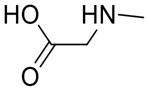	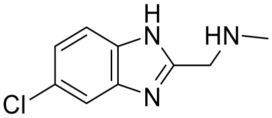	196.273>166.060 (20, 40)	4.24
32	Lactic acid	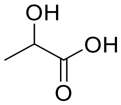	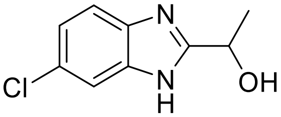	196.817>143.802 (2, 24)	3.95
33	Oxalic acid	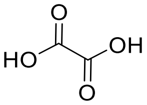	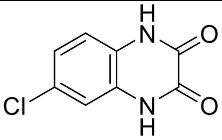	196.972>151.042 (34, 22)	4.34
34	γ-Amino butyric acid	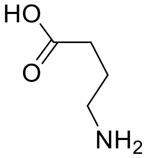	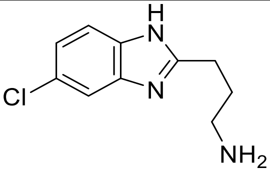	210.300>166.05 (20, 40)	4.44
35	3-Hydrobutyric acid	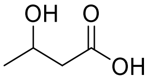	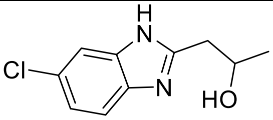	211.152>179.077 (26, 16)	5.09
36	Threonine	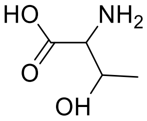	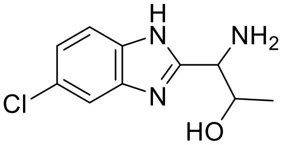	226.300>166.014 (25, 30)	4.24
37	Cysteine	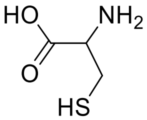	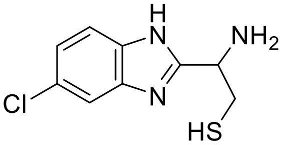	228.34>166.036 (25, 22)	5.65
38	Pyroglutamic acid	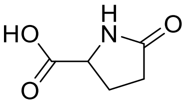	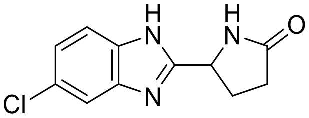	236.295>165.996 (30, 30)	4.89
39	Cis-Aconitic acid	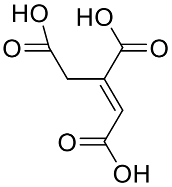	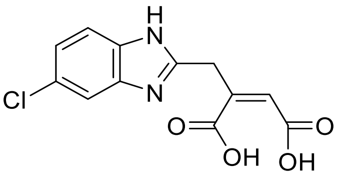	281.288>166.06 (20, 40)	4.62
40	Citric/Isocitric acid	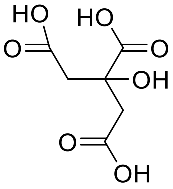	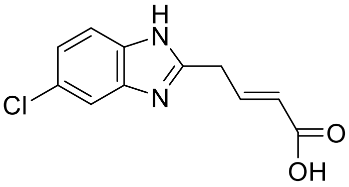	237.259>166.115 (74, 26)	5.08

41	Glutaconic acid	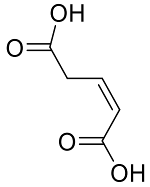	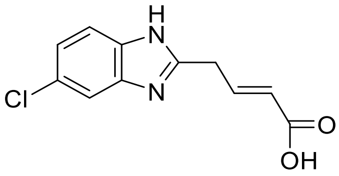	237.279>166.06 (20, 40)	5.13
42	4-Hydroxy proline	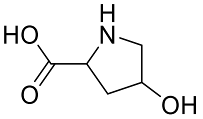	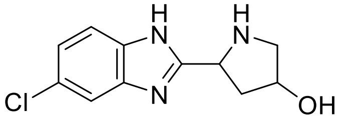	238.966>179.133 (10, 40)	4.20
43	Asparagine/Glutaric acid	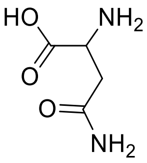	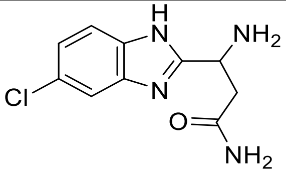	239.299>166.06 (20, 40)	4.26
44	Ornithine	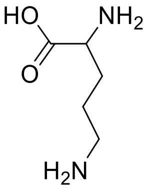	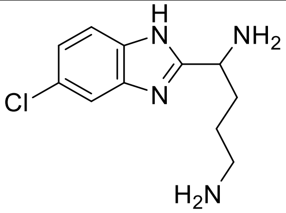	239.299>209.06 (20, 40)	6.28
45	Adipic acid	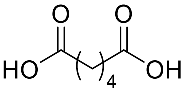	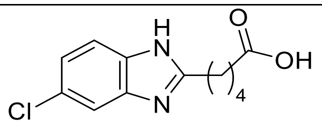	253.16>166.06 (20, 40)	4.21
46	Glutamic acid	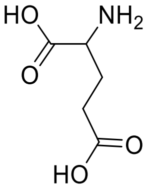	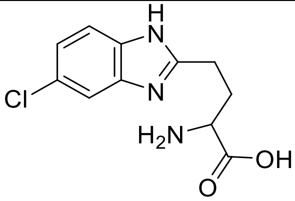	366.23>304.053 (20, 40)	6.81
47	Mevalonic acid	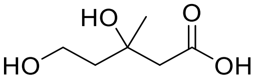	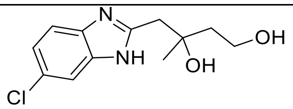	255.11 > 219.09 (34, 18)	3.93
48	2-Hydroxy glutaric acid	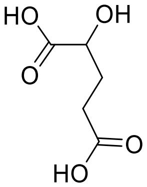	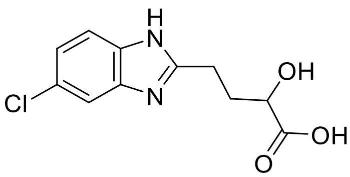	255.142>209.101 (46, 18)	3.92
49	Dihydroorotic acid	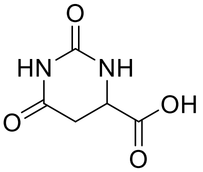	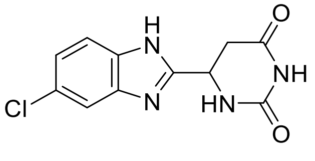	265.29>166.06 (20, 40)	5.62
50	2,2-Dimethylglutaric acid	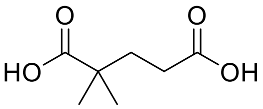	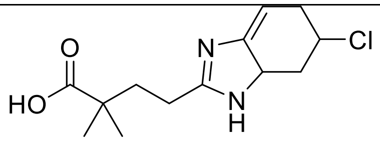	267.35>180.07 (20, 40)	6.51

51	2-Hydroxy octanoic acid	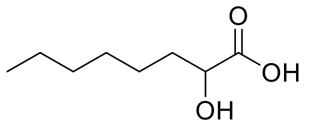	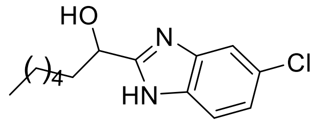	267.39>182.02 (20, 40)	5.31
52	3-Hydroxy octanoic acid	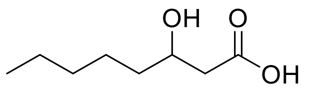	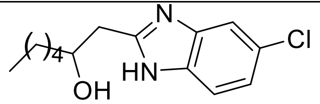	267.39>250.12 (20, 40)	6.51
53	Hippuric acid	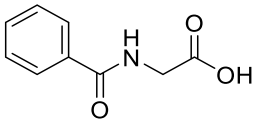	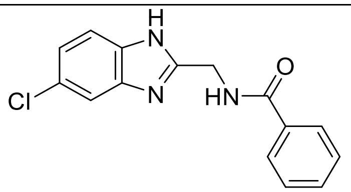	286.355>166.062 (60, 60)	4.66
54	Arginine	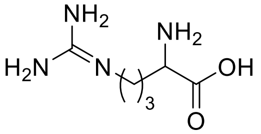	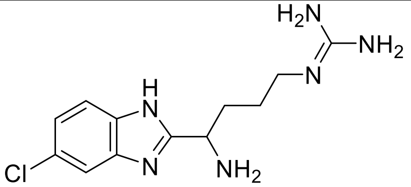	281>166.06 (20, 40)	4.61
55	10-Undecenoic acid	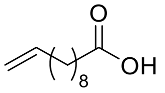	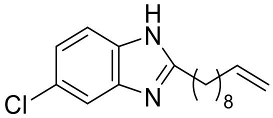	291.46>166.06 (20, 40)	5.86
56	Tridecanoic acid	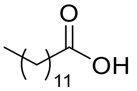	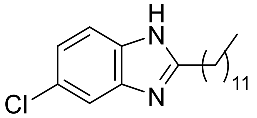	321.48>166.06 (20, 40)	6.61
57	2/3-Hydroxy dodecanoic acid	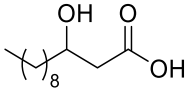	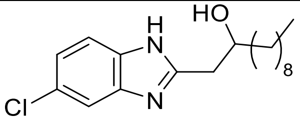	323.5>166.026 (20, 40)	6.45
58	Myristic acid	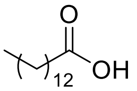	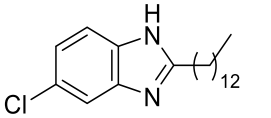	335.55>166.06 (20, 40)	6.85
59	Dodecanedioic acid	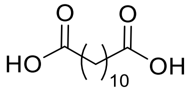	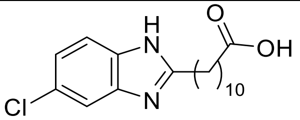	337.48>165.96 (30, 30)	5.45
60	3-Hydroxy myristic acid	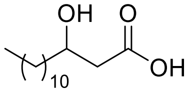	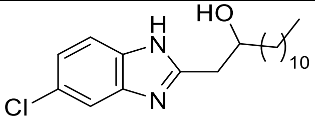	351.55>166.06 (20, 40)	5.65
61	Pentadecanoic acid	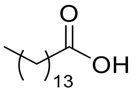	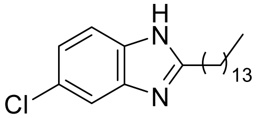	349.58>166.06 (20, 40)	7.09
62	Palmitoleic acid	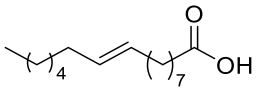	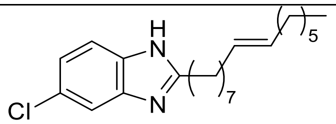	361.59>166.06 (20, 40)	6.98

63	Heptadecenoic acid	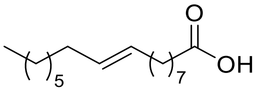	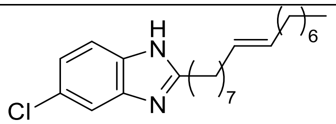	375.58>166.06 (20, 40)	7.19
64	Heptadecanoic acid	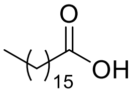	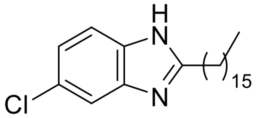	377.63>166.06 (20, 40)	7.58
65	16-Hydroxy palmitic acid	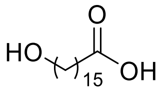	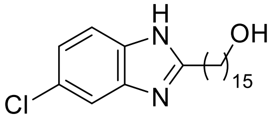	379.6>166.06 (20, 40)	6.05
66	Linoeliaidic acid	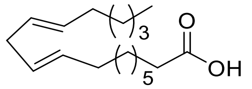	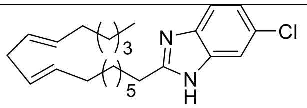	387.345>166.06 (20, 40)	7.23
67	Oleic acid/ Elaidic acid	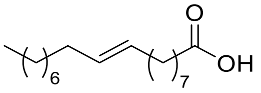	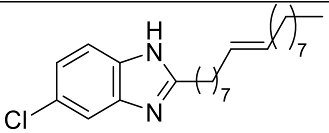	389.64>166.06 (20, 40)	7.45
68	Stearic acid	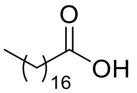	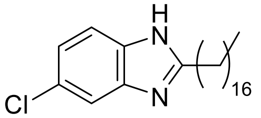	391.66>166.06 (20, 40)	7.82
69	9-Cis Retinoic acid	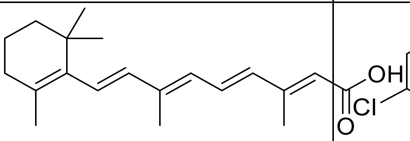	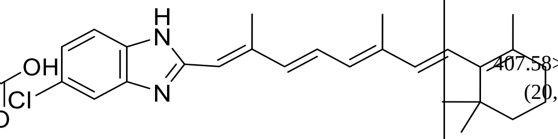	407.58>166.06 (20, 40)	6.46
70	Eicosapentaenoic acid	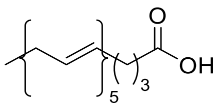	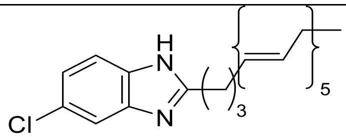	409.63>166.06 (20, 40)	7.30
71	Arachidonic acid	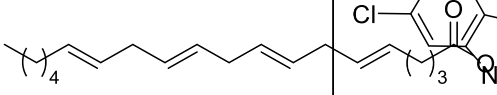	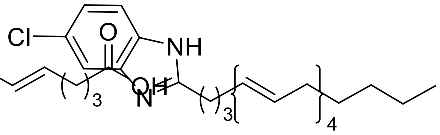	411.65>165.96 (30, 30)	7.17
72	Cis-11-Eicosenoic acid	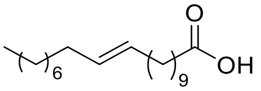	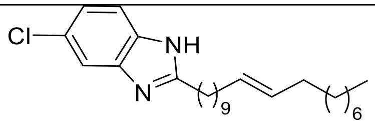	417.69>166.06 (20, 40)	7.89
73	Docosahexaenoic acid	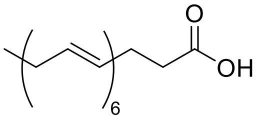	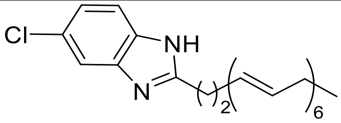	435.67>166.06 (20, 40)	6.87
74	Heneicosanoic acid	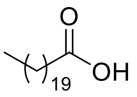	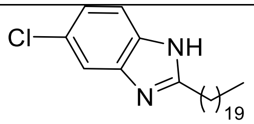	433.74>166.06 (20, 40)	8.71
75	Erucic acid	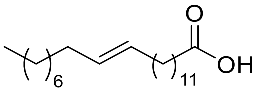	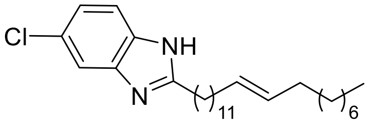	445.75>166.06 (20, 40)	8.38

76	2-Phenyl-2-propyl-succinic acid	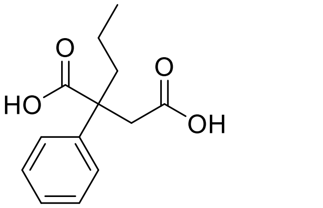	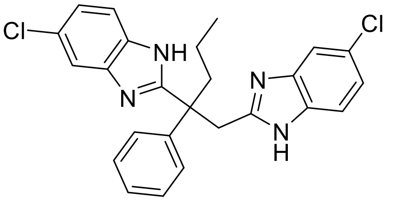	450.3>166.06 (30, 30)	9.91
77	Valine-d8	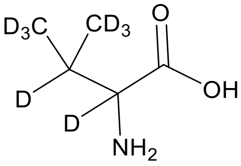	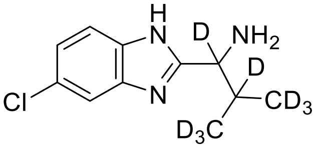	232.23>166.03 (30, 25)	4.71
78	Methionine-1-13C	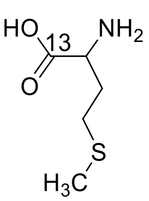	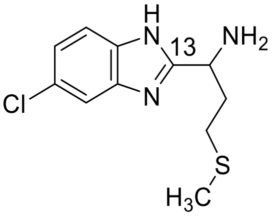	257.3>153.06 (20, 50)	5.23
79	Alanine-13C	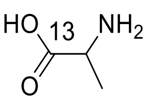	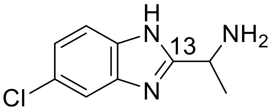	197.25>182.01 (20, 20)	4.18
80	Octanoic acid-13C	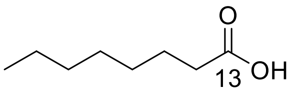	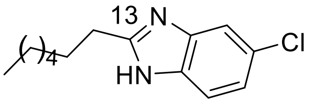	251.29>153.02 (20, 40)	5.6
81	Succinic acid-d6	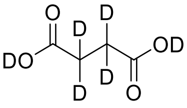	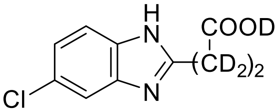	230.195>168.01 (20, 25)	4.25
82	Arachidonic acid-d8	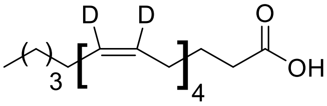	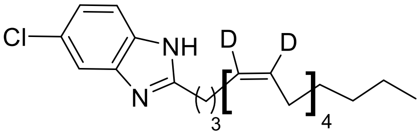	418.75>166.96 (30, 30)	7.27
83	Docosahexaenoic acid-d5	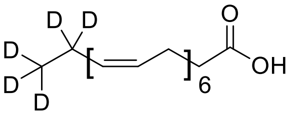	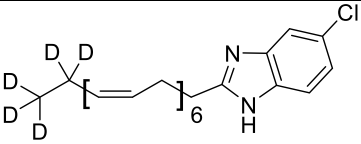	439.55>166.96 (25, 30)	7.52
84	4-Aminobutyric acid-4,4-d2	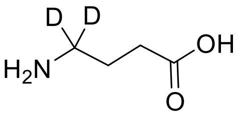	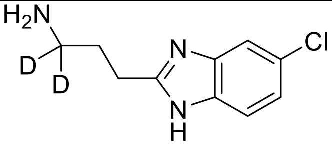	212.18>166.67 (28, 30)	4.52
85	Pyruvic acid-13C3	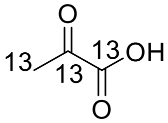	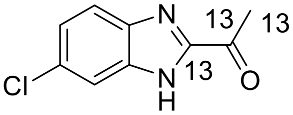	197.83>181 (34, 28)	4.18

86	Malic acid-d3	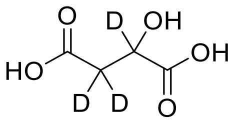	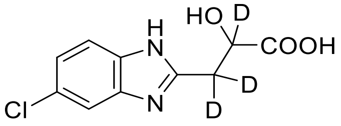	244.479>182.032 (20, 16)	3.98
87	α-Keto glutaric acid-d4	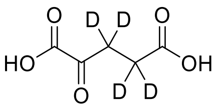	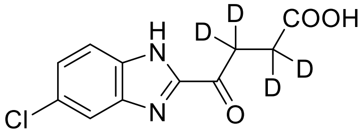	257.671>152.01 (30, 40)	4.31

Supplementary Table S2. Quantitation results of 76 CCMs in human body fluids, tissue, and cell lines (in ng/mL).

S. No.	Carboxyl-containing metabolites	Concentration of CCMs in ng/mL						
		Tissue (10 mg)	NIST (2 µL)	Plasma (2 µL)	Serum (2 µL)	Urine (5 µL)	Saliva (5 µL)	PANC-1 (50K)
1	Glyceric acid	ND	98.12	355.40	132.66	193.07	119.00	1.1342
2	Glyoxalic acid	154.10	11.66	32.20	46.15	22.33	110.80	0.0332
3	Propionic acid	182.18	30.90	29.12	32.95	23.43	29.35	0.0918
4	Pyruvic acid	1105.78	1316.98	1973.82	2830.12	1763.53	1132.22	7.6327
5	Alanine	1304.73	1679.32	2121.02	4852.65	2336.25	2689.17	7.2958
6	2-Oxo butyric acid	3988.87	102.08	141.13	120.10	152.90	88.18	0.416
7	Malonic acid	664.33	168.90	211.70	102.62	173.03	110.85	0.4858
8	Serine	86.45	150.12	218.68	86.60	163.03	92.65	0.4296
9	4-Methyl-2-oxo-pentanoic acid	560.00	237.47	99.38	79.33	161.73	161.62	1.5342
10	Fumaric acid	213.4	84.2	64.2	96.4	65.8	69.7	0.495
11	Maleic acid	912.53	328.42	101.28	81.36	203.17	202.50	1.8687
12	Valine	1362.48	276.18	89.13	63.78	159.32	168.82	1.4862
13	Succinic acid	3028.47	277.80	213.00	235.63	292.37	338.43	0.729
14	Ureidopropionic acid	2.50	1.12	1.35	1.95	1.20	1.12	0.0107
15	Oxaloacetic acid	525.73	10.75	12.18	390.53	13.26	29.30	ND
16	Aspartic acid	1673.23	12.47	2.33	52.77	10.70	4.52	0.0935
17	Malic acid	3134.72	26.68	29.60	80.67	34.72	19.18	0.1598
18	Homocysteine	1392.50	8.77	ND	33.95	15.92	0.20	0.0532
19	α-Keto glutaric acid	701.40	33.20	4.13	55.12	23.43	3.13	0.2932
20	Glutamine	642.23	46.65	10.07	49.97	28.00	4.87	0.2538
21	Methionine	2806.95	4734.87	8862.48	8494.47	5466.43	3031.80	7.7685
22	Orotic acid	2444.88	1545.65	131.72	50.40	540.52	191.05	1.878
23	3-Methyl adipic acid	269.53	32.63	34.78	41.85	106.98	41.58	0.2828
24	N-Acetyl glutamine	2183.42	333.35	201.58	211.63	237.47	107.95	1.861
25	Ascorbic acid	302.10	248.55	663.23	344.05	178.22	95.06	0.077

26	3-Nitrotyrosine	4518.22	406.68	552.67	238.15	935.85	99.08	0.8855
27	Methylmalonic acid	ND	1140.25	3391.00	3120.35	2113.27	640.90	1.6825
28	Docosatetraenoic acid	434.68	29.53	63.18	47.00	73.35	110.53	1.5563
29	Palmitic acid	6436.25	2162.38	928.32	208.87	2523.72	3032.07	41.3982
30	Glycine	199.47	22.78	18.90	24.65	15.73	11.88	0.0773
31	Sarcosine	155.50	233.53	133.62	200.55	230.53	438.27	0.3562
32	Lactic acid	65.22	18.38	26.65	29.87	9.98	29.25	0.093
33	Oxalic acid	899.32	166.82	146.90	379.53	125.20	85.55	0.2363
34	γ-Amino butyric acid	86.18	78.12	18.80	124.22	41.17	10.07	0.092
35	3-Hydrobutyric acid	3995.53	1199.93	1468.93	807.88	1995.62	208.73	1.0618
36	Threonine	2293.17	99.22	38.18	638.75	112.22	41.70	0.4547
37	Cysteine	207.63	56.00	48.53	21.67	48.40	9.78	0.0474
38	Pyroglutamic acid	401.03	43.82	18.75	43.13	89.52	32.12	0.468
39	Cis-Aconitic acid	66.17	1.87	0.28	5.15	0.15	ND	0.0064
40	Citric/Isocitric acid	1636.68	426.05	293.03	217.20	294.13	257.07	5.681
41	Glutaconic acid	2027.37	459.38	321.40	227.28	327.50	272.02	6.6508
42	4-Hydroxy proline	1086.52	11.20	4.03	30.67	7.13	3.55	0.0872
43	Asparagine/Glutaric acid	987.37	10.02	7.88	37.13	8.70	5.20	0.070
44	Ornithine	1067.32	43.70	17.28	92.65	38.20	19.13	0.2073
45	Adipic acid	347.13	23.75	5.27	35.43	15.85	4.95	0.2353
46	Glutamic acid	46.95	145.82	222.68	182.57	142.23	194.20	0.3415
47	Mevalonic acid	5.13	1.08	0.95	3.67	2.57	0.20	0.002
48	2-Hydroxy glutaric acid	ND	14.08	7.60	14.45	11.90	7.12	0.0522
49	Dihydroorotic acid	2778.13	2525.03	2960.85	2537.67	2627.30	2551.38	18.282
50	2,2-Dimethylglutaric acid	39.80	29.62	32.67	22.12	25.78	25.37	0.1993
51	2-Hydroxy octanoic acid	1.71	1.50	1.48	1.56	1.48	1.38	0.0139

52	3-Hydroxy octanoic acid	212.12	45.08	28.10	38.32	73.38	45.75	0.4668
53	Hippuric acid	168.95	100.07	170.97	61.27	118.27	135.63	0.5823
54	Arginine	59.13	4.92	3.33	7.87	3.10	1.85	0.0372
55	10-Undecenoic acid	7.27	5.78	6.32	3.92	4.72	7.43	0.0438
56	Tridecanoic acid	89.27	150.90	161.78	117.43	113.92	102.97	0.7718
57	2/3-Hydroxy dodecanoic acid	3.18	3.32	3.53	2.67	2.27	2.40	0.0167
58	Myristic acid	389.10	207.17	248.07	162.40	219.13	205.63	2.1272
59	Dodecanedioic acid	2870.18	148.65	130.38	72.82	111.03	57.60	5.2045
60	3-Hydroxy myristic acid	59.78	8.05	6.57	6.53	5.38	6.70	0.0928
61	Pentadecanoic acid	739.93	477.18	457.40	350.88	417.22	469.80	5.7507
62	Palmitoleic acid	456.73	71.02	15.98	12.60	37.52	118.72	1.9232
63	Heptadecenoic acid	158.18	44.48	12.52	3.72	16.53	48.87	0.974
64	Heptadecanoic acid	1547.23	556.53	299.08	217.70	372.70	669.23	5.6572
65	16-Hydroxy palmitic acid	71.15	9.88	8.12	8.10	6.72	8.30	0.1133
66	Linoeliaidic acid	7997.12	951.87	276.58	23.97	431.35	729.05	3.8245
67	Oleic acid/ Elaidic acid	6208.93	3711.07	623.55	64.35	1386.43	3895.68	33.125
68	Stearic acid	5236.50	2637.82	1495.55	582.73	2293.77	3426.10	24.2295
69	9-Cis Retinoic acid	56.20	6.47	4.70	3.20	4.73	1.95	0.044
70	Eicosapentoic acid	458.93	40.35	32.30	137.27	26.88	22.82	0.4723
71	Arachidonic acid	718.98	191.85	31.30	4.53	139.32	94.70	1.4938
72	Cis-11-Eicosenoic acid	1341.63	363.13	187.38	57.55	322.00	1472.93	4.5103
73	Docosahexanoic acid	47.62	8.44	10.97	4.20	2.28	4.80	0.3647
74	Heneicosanoic acid	312.67	44.75	38.18	16.28	56.07	167.02	1.5405
75	Erucic acid	1508.10	320.70	71.97	8.55	178.43	2324.15	2.5322
76	2-Phenyl-2-propyl-succinic acid	3684.73	886.83	857.35	806.33	663.23	497.13	20.3593
ND = not detected								

Supplementary Figure S2. LC Chromatogram of selected carboxylic acids

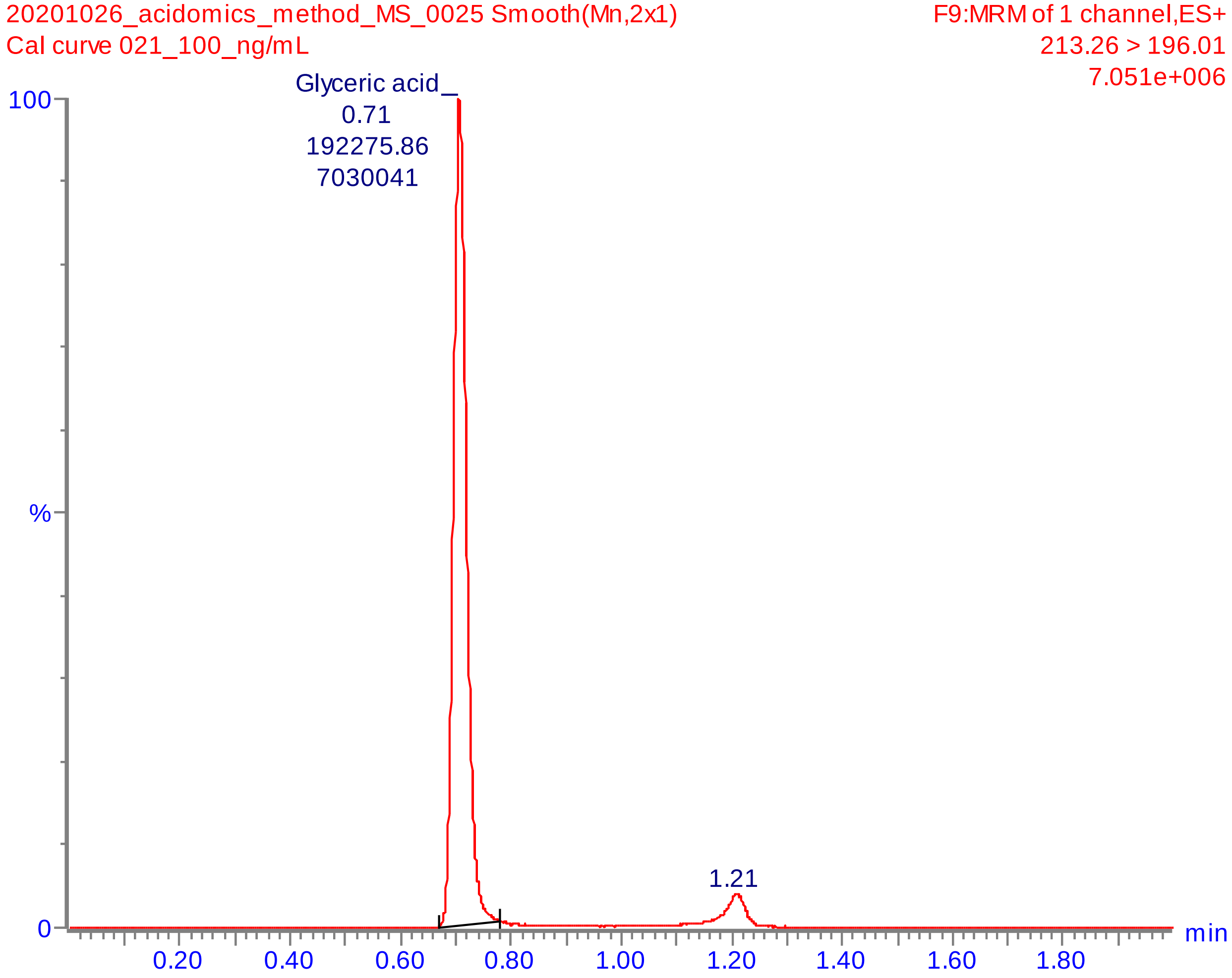

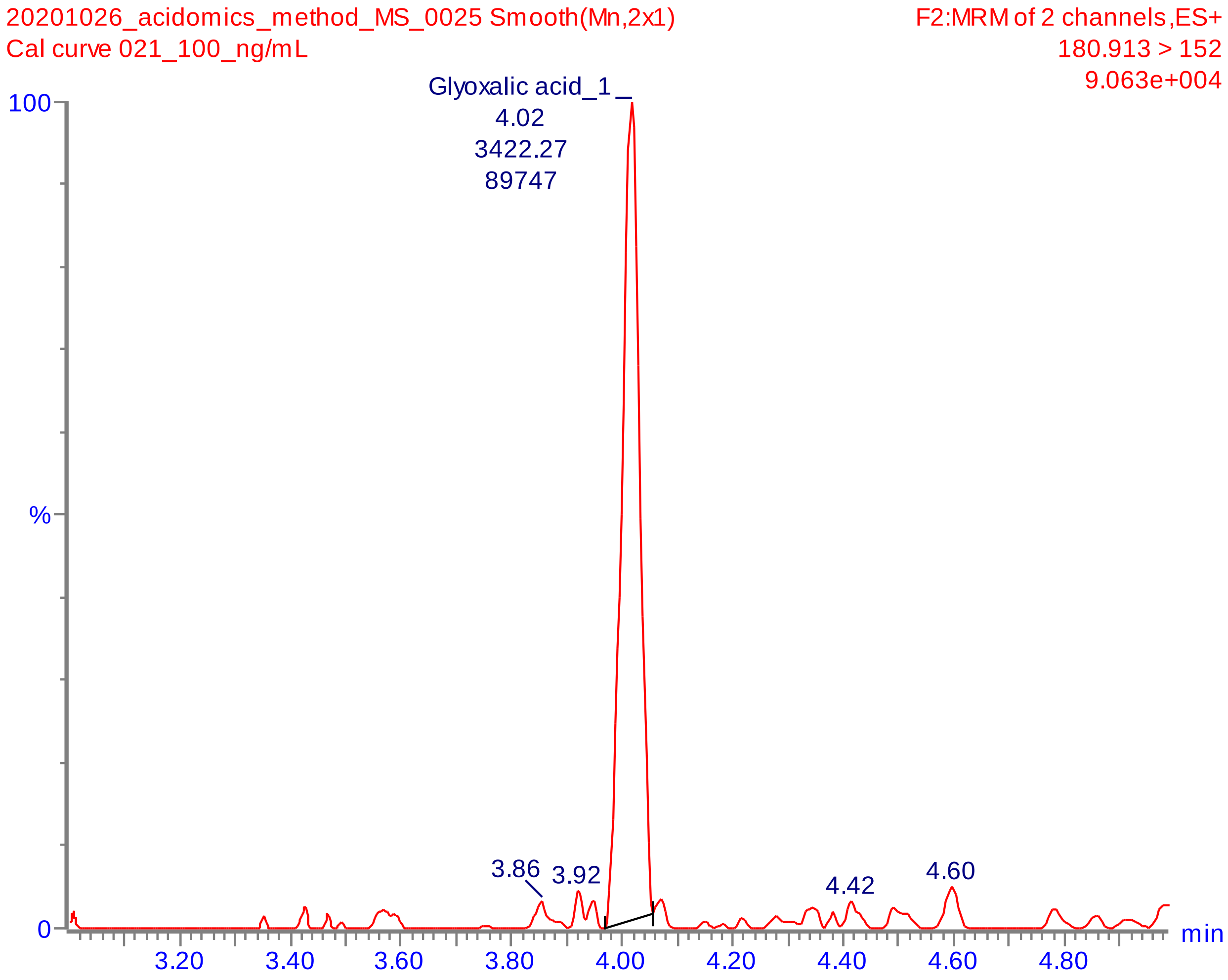
20201026_acidomics_method_MS_0025 Smooth(Mn,2x1)
Cal curve 021_100_ng/mL
F2:MRM of 2 channels,ES+
180.913 > 152
9.063e+004
Glyoxalic acid_1
4.02
3422.27
89747
100
%
0
3.86
3.92
4.42
4.60
3.20
3.40
3.60
3.80
4.00
4.20
4.40
4.60
4.80
min

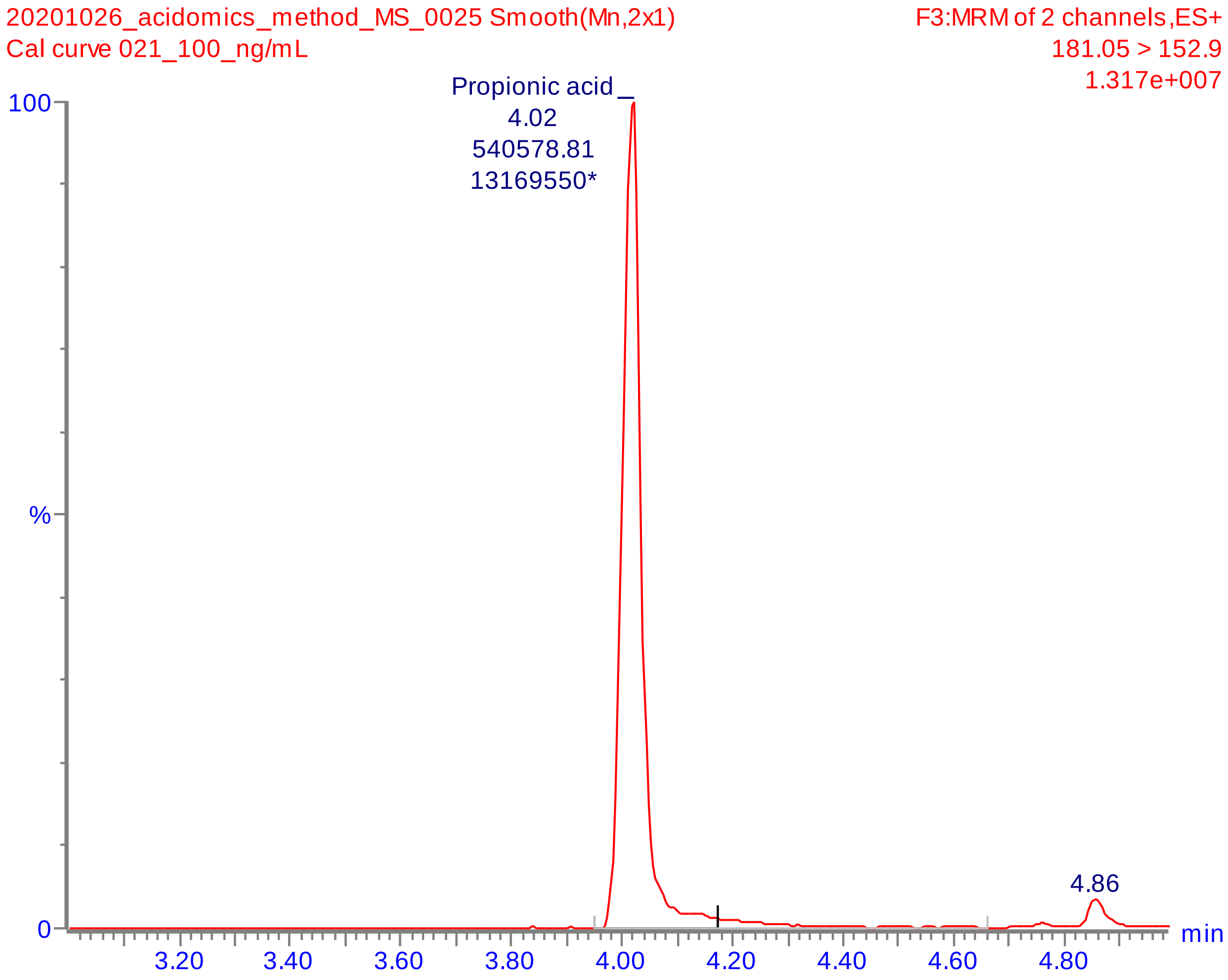
20201026_acidomics_method_MS_0025 Smooth(Mn,2x1)
Cal curve 021_100_ng/mL
F3:MRM of 2 channels,ES+
181.05 > 152.9
1.317e+007
Propionic acid
4.02
540578.81
13169550*
4.86
100
%
0
3.20
3.40
3.60
3.80
4.00
4.20
4.40
4.60
4.80
min

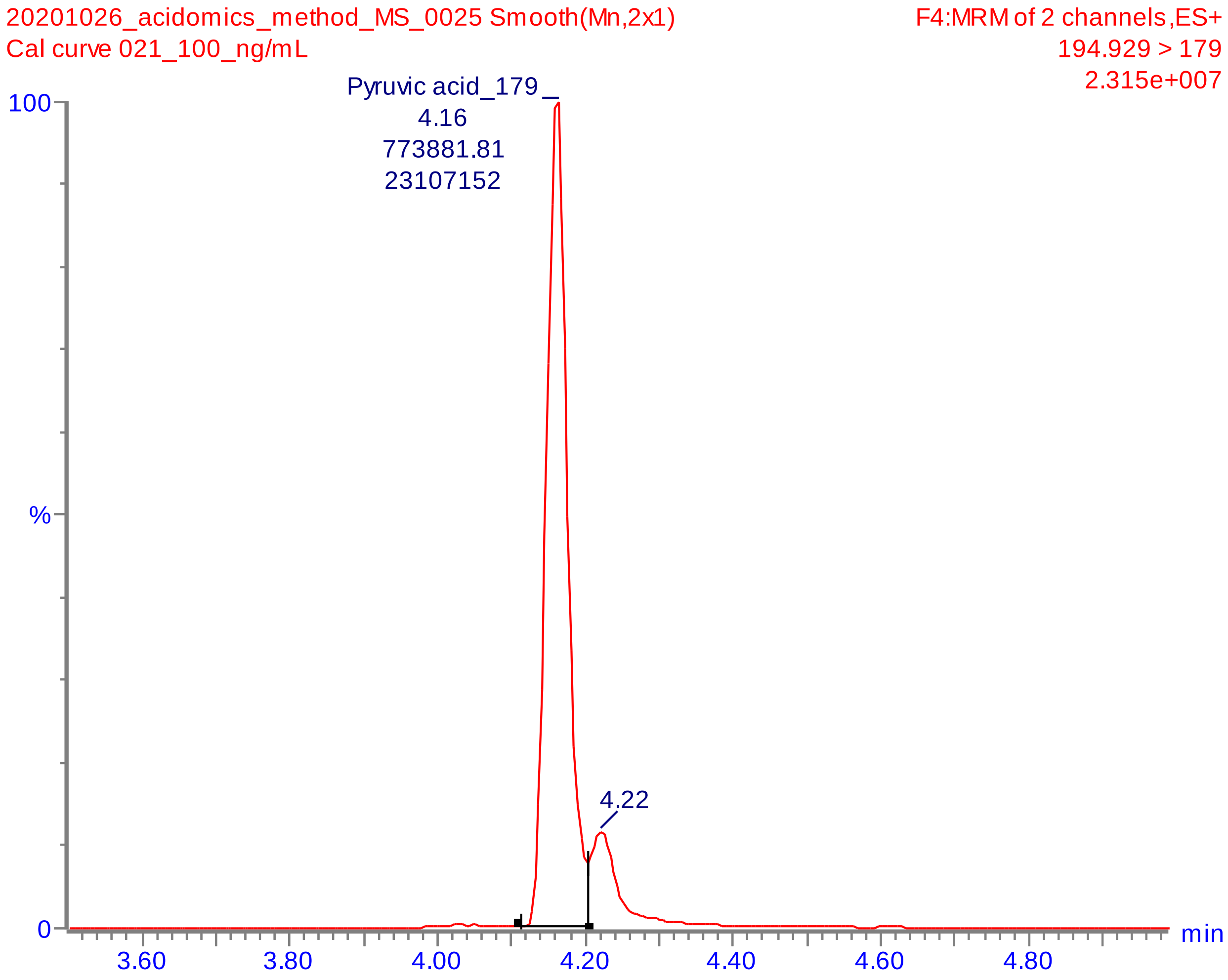
20201026_acidomics_method_MS_0025 Smooth(Mn,2x1)
Cal curve 021_100_ng/mL
F4:MRM of 2 channels,ES+
194.929 > 179
2.315e+007
Pyruvic acid_179
4.16
773881.81
23107152
4.22
100
%
0
3.60
3.80
4.00
4.20
4.40
4.60
4.80
min

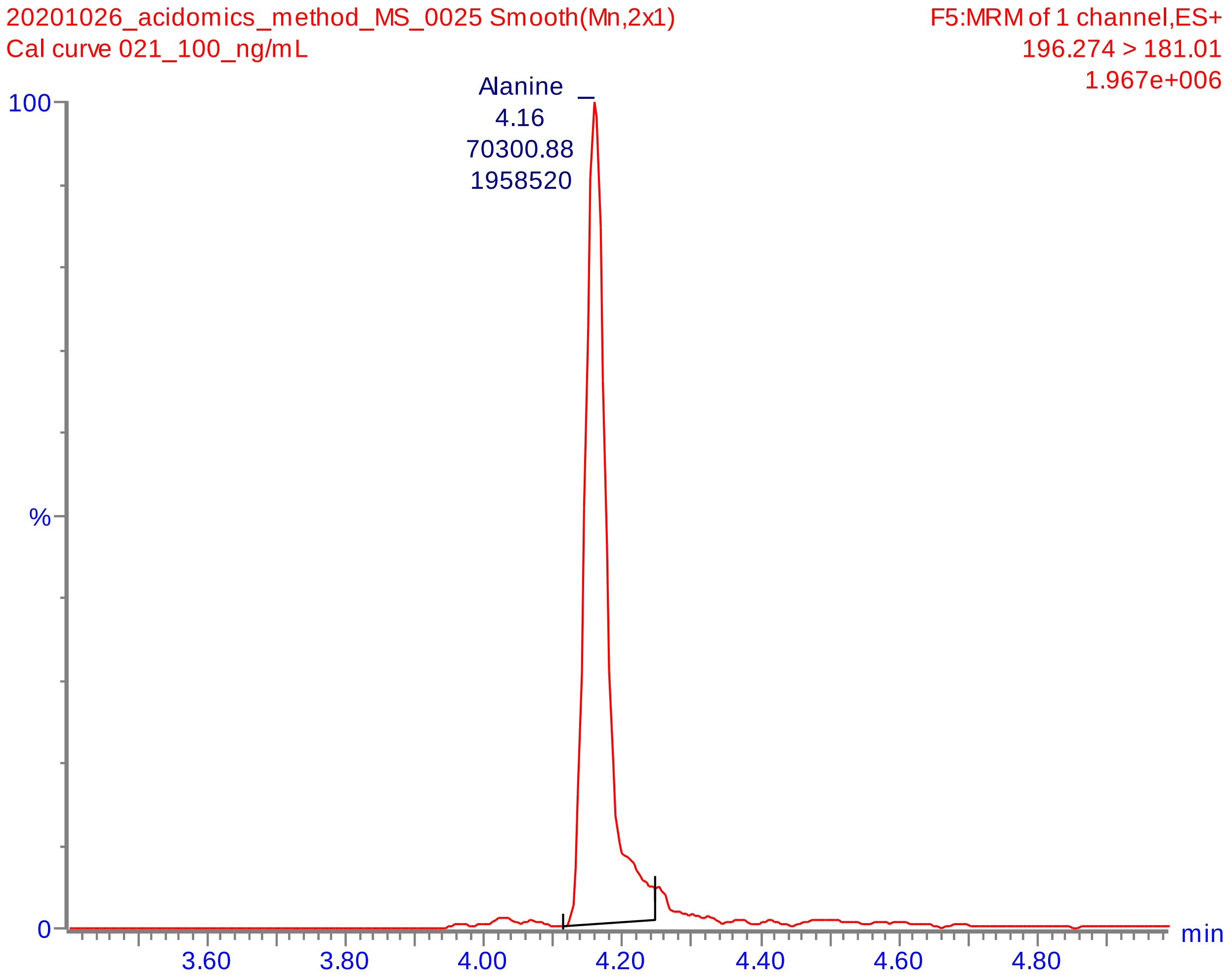
20201026_acidomics_method_MS_0025 Smooth(Mn,2x1)
Cal curve 021_100_ng/mL
F5:MRM of 1 channel,ES+
196.274 > 181.01
1.967e+006
Alanine
4.16
70300.88
1958520
100
%
0
3.60
3.80
4.00
4.20
4.40
4.60
4.80
min

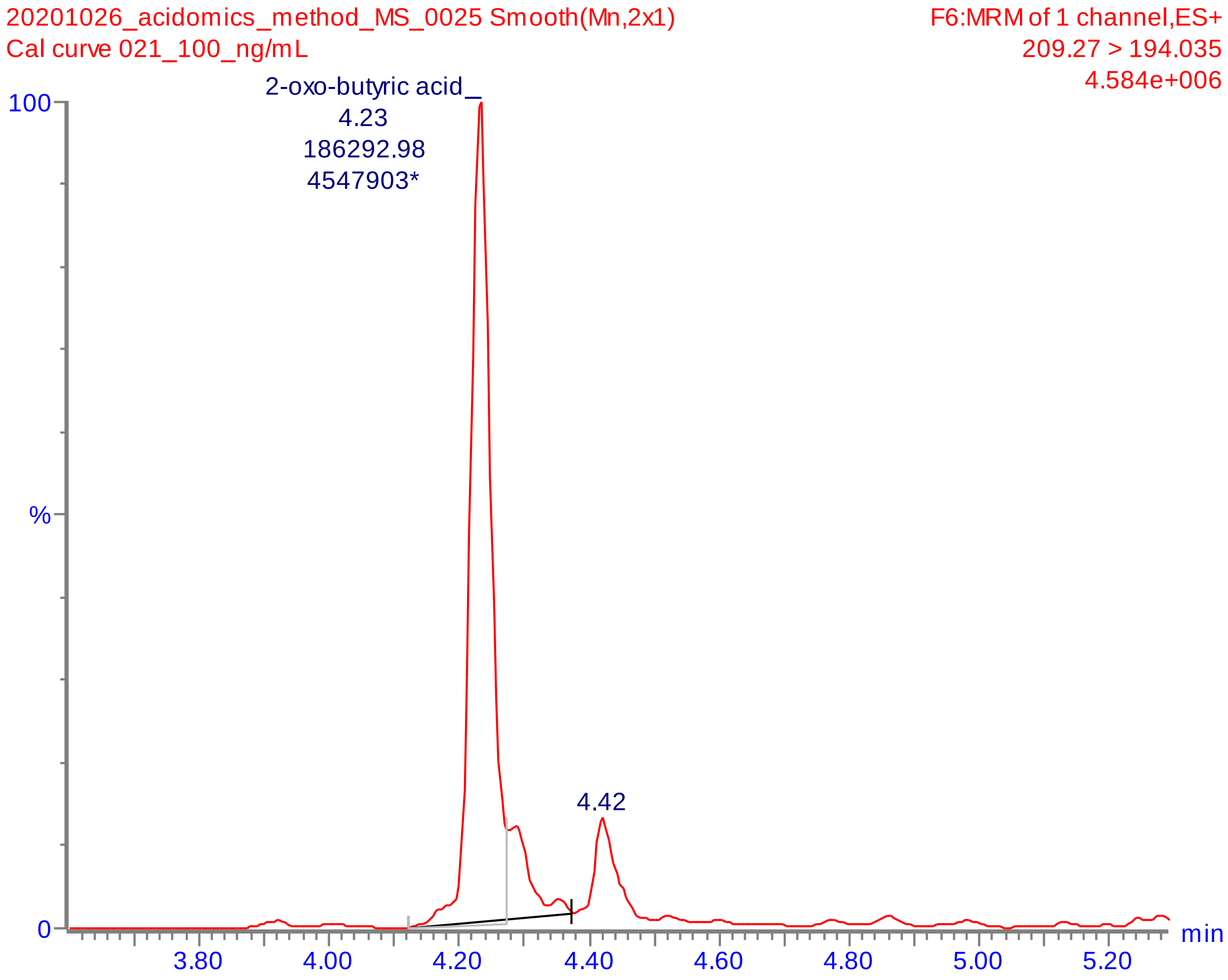

20201026_acidomics_method_MS_0025 Smooth(Mn,2x1)
Cal curve 021_100_ng/mL
F6:MRM of 1 channel,ES+
209.27 > 194.035
4.584e+006
2-oxo-butyric acid_
4.23
186292.98
4547903*
4.42
100
%
0
3.80
4.00
4.20
4.40
4.60
4.80
5.00
5.20
min

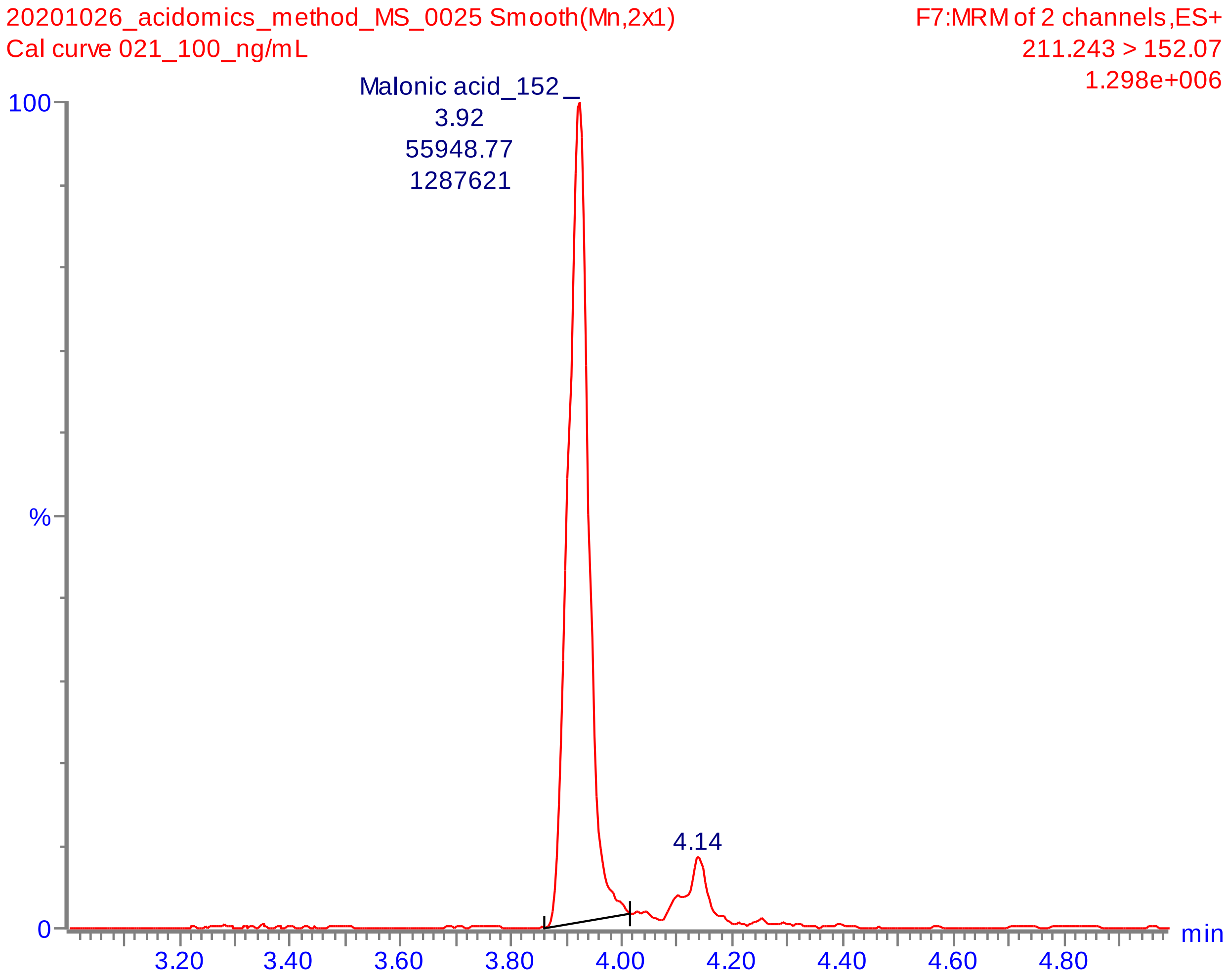
20201026_acidomics_method_MS_0025 Smooth(Mn,2x1)
Cal curve 021_100_ng/mL
F7:MRM of 2 channels,ES+
211.243 > 152.07
1.298e+006
Malonic acid_152
3.92
55948.77
1287621
4.14
100
%
0
3.20
3.40
3.60
3.80
4.00
4.20
4.40
4.60
4.80
min

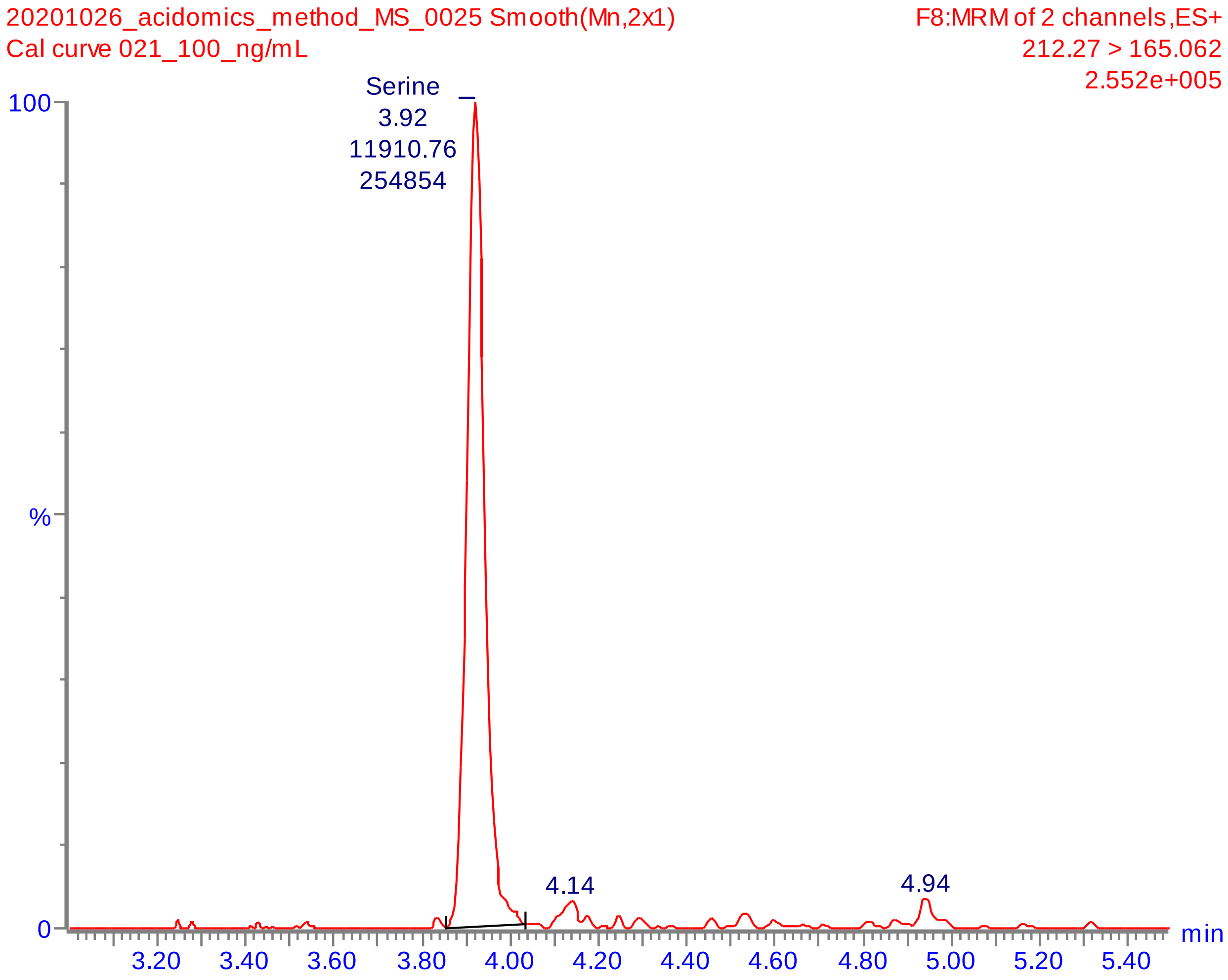

20201026_acidomics_method_MS_0025 Smooth(Mn,2x1)
Cal curve 021_100_ng/mL
F8:MRM of 2 channels,ES+
212.27 > 165.062
2.552e+005
Serine
3.92
11910.76
254854
4.14
4.94
100
%
0
3.20
3.40
3.60
3.80
4.00
4.20
4.40
4.60
4.80
5.00
5.20
5.40
min

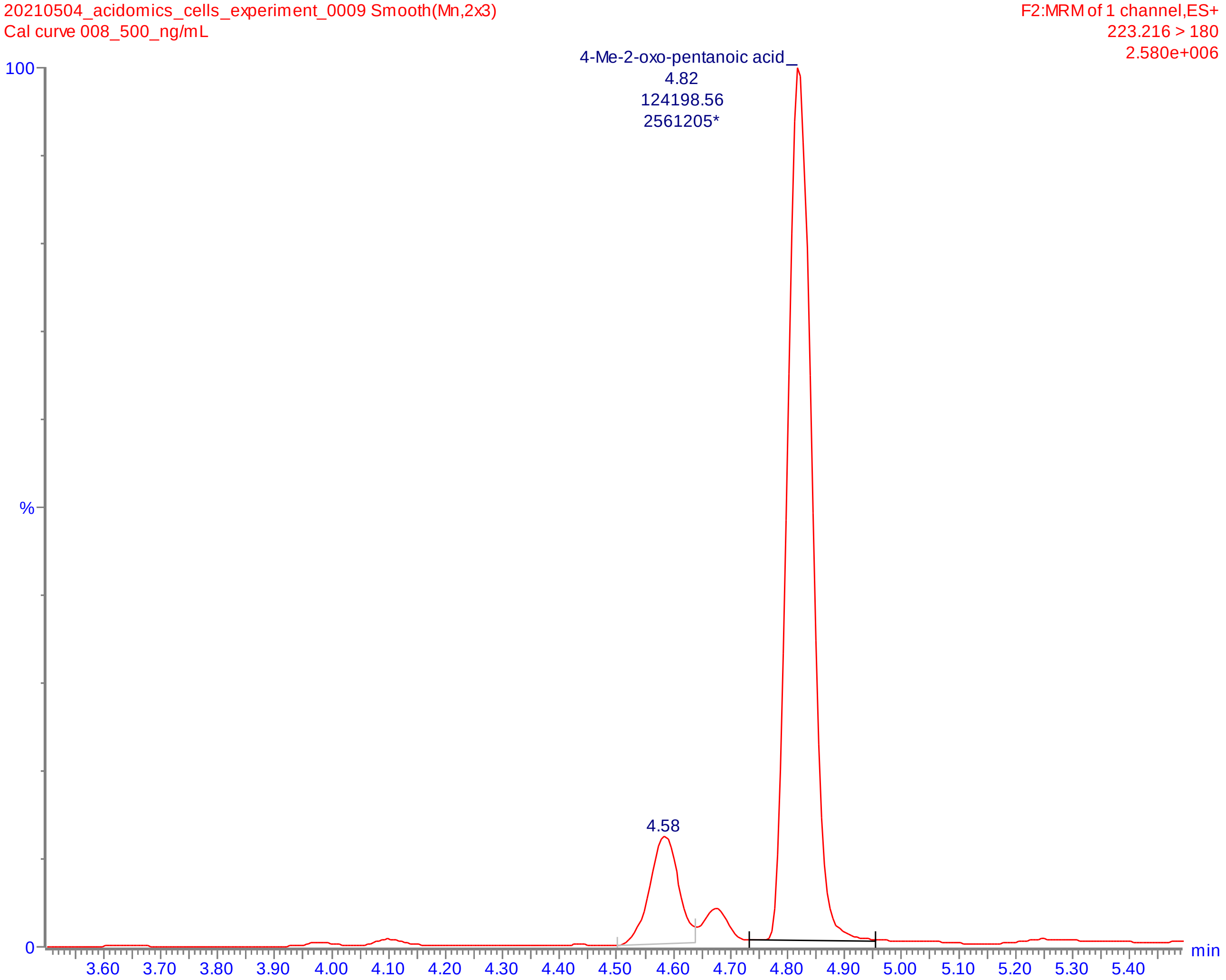
20210504_acidomics_cells_experiment_0009 Smooth(Mn,2x3)
Cal curve 008_500_ng/mL
F2:MRM of 1 channel,ES+
223.216 > 180
2.580e+006
4-Me-2-oxo-pentanoic acid
4.82
124198.56
2561205*
4.58
100
%
0
3.60
3.70
3.80
3.90
4.00
4.10
4.20
4.30
4.40
4.50
4.60
4.70
4.80
4.90
5.00
5.10
5.20
5.30
5.40
min

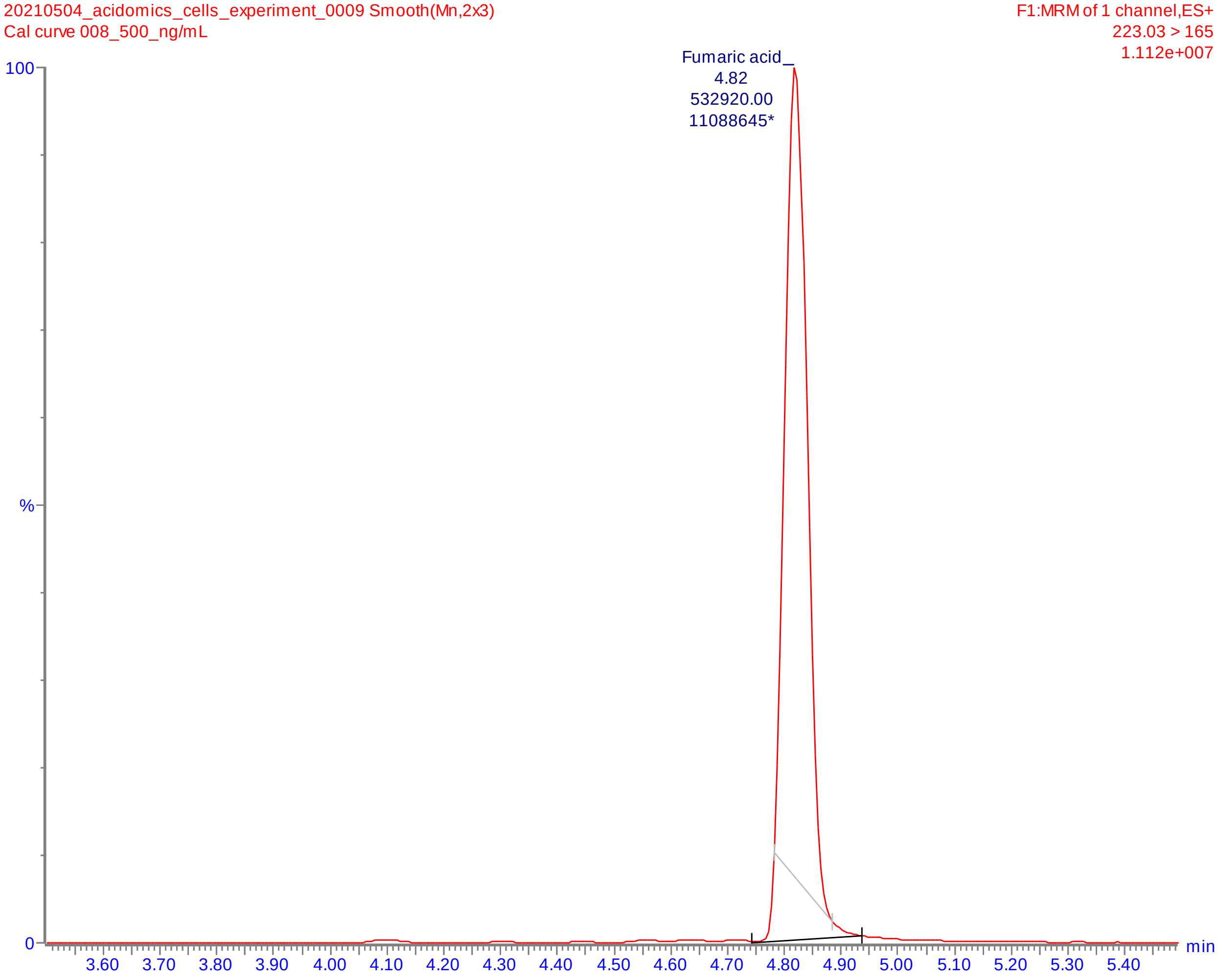

20210504_acidomics_cells_experiment_0009 Smooth(Mn,2x3)
Cal curve 008_500_ng/mL
F1:MRM of 1 channel,ES+
223.03 > 165
1.112e+007
Fumaric acid_
4.82
532920.00
11088645*
100
%
0
3.60
3.70
3.80
3.90
4.00
4.10
4.20
4.30
4.40
4.50
4.60
4.70
4.80
4.90
5.00
5.10
5.20
5.30
5.40
min

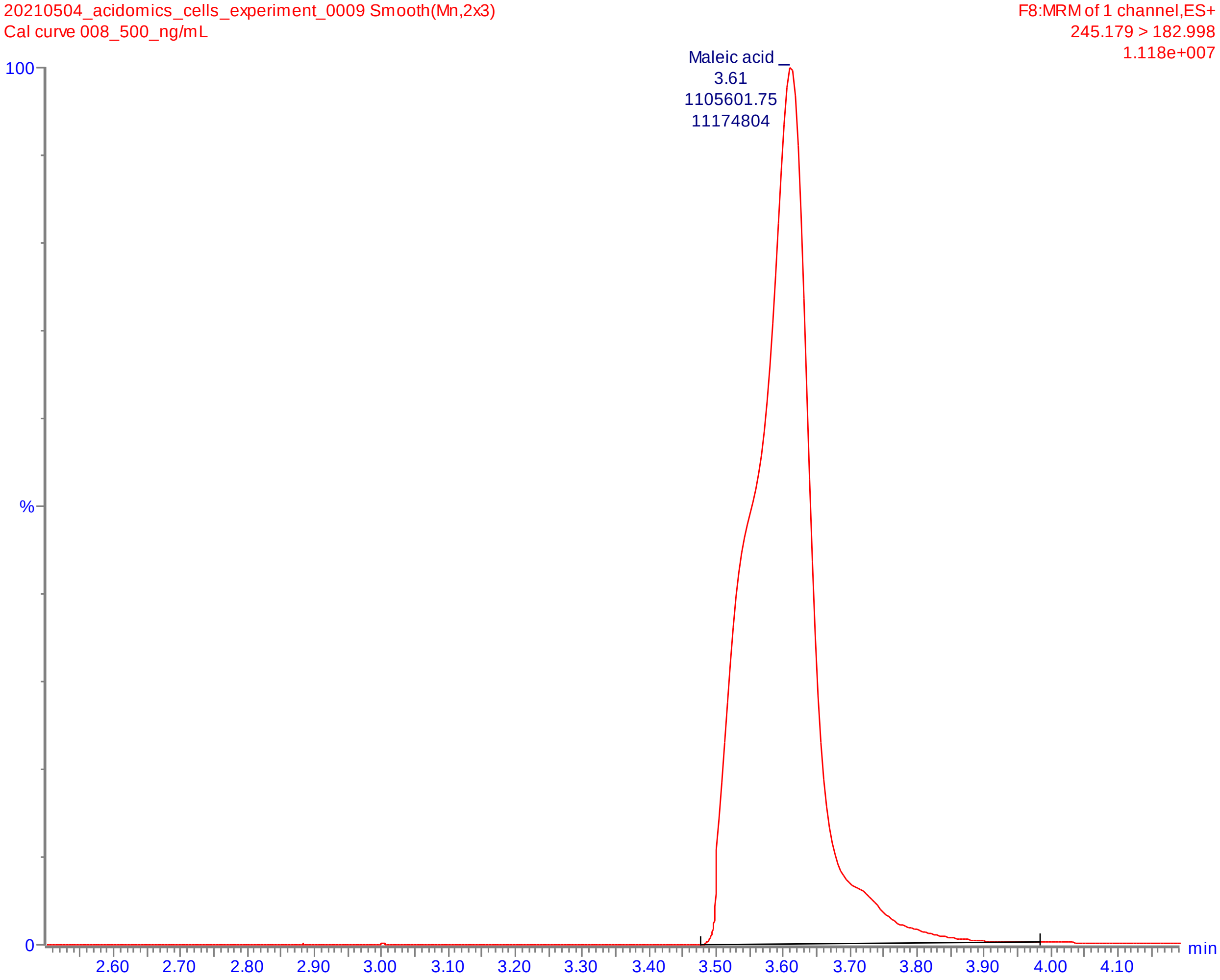

20210504_acidomics_cells_experiment_0009 Smooth(Mn,2x3)
Cal curve 008_500_ng/mL
F8:MRM of 1 channel,ES+
245.179 > 182.998
1.118e+007
Maleic acid
3.61
1105601.75
11174804
100
%
0
2.60
2.70
2.80
2.90
3.00
3.10
3.20
3.30
3.40
3.50
3.60
3.70
3.80
3.90
4.00
4.10
min

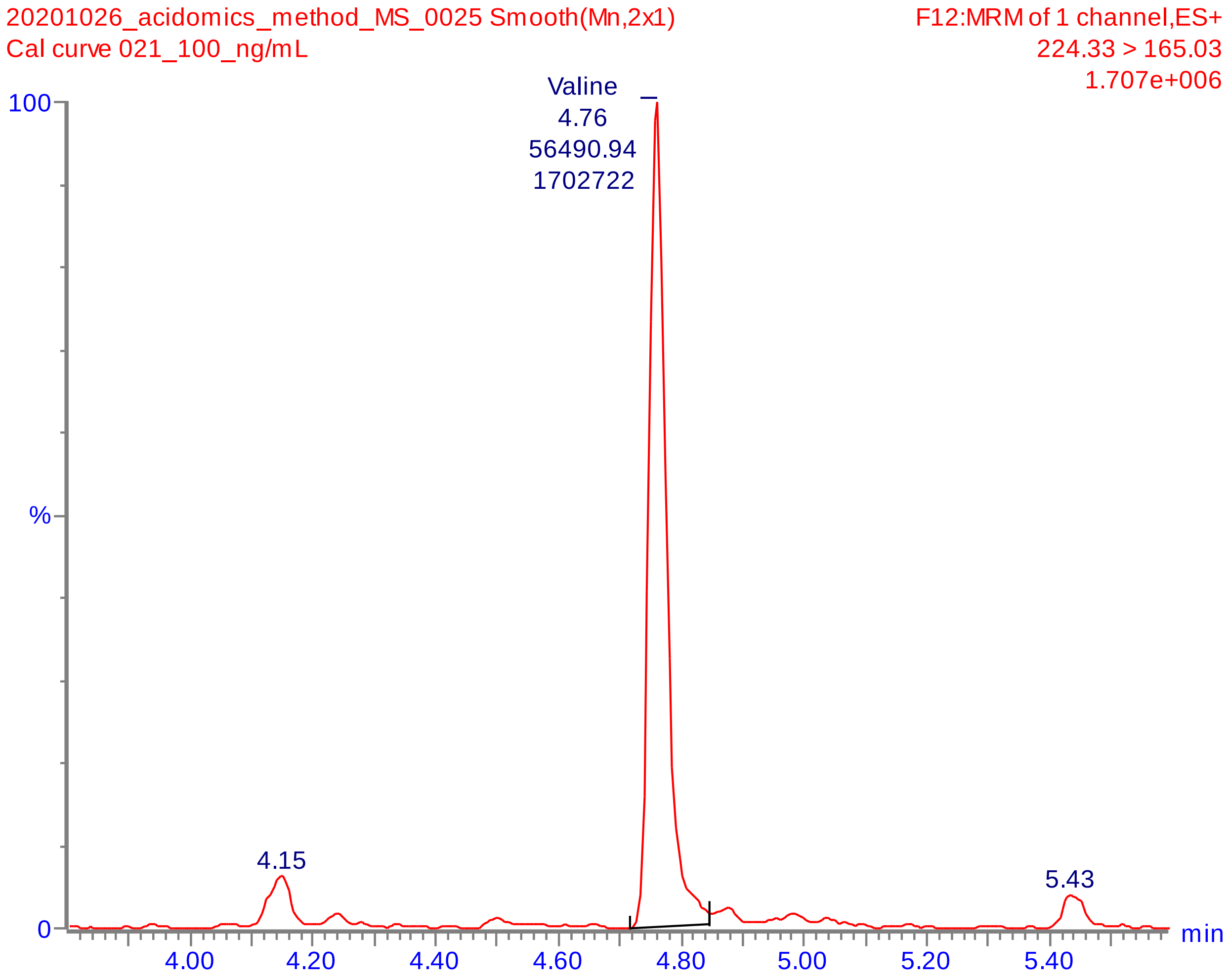
20201026_acidomics_method_MS_0025 Smooth(Mn,2x1)
Cal curve 021_100_ng/mL
F12:MRM of 1 channel,ES+
224.33 > 165.03
1.707e+006
Valine
4.76
56490.94
1702722
4.15
5.43
100
%
0
4.00
4.20
4.40
4.60
4.80
5.00
5.20
5.40
min

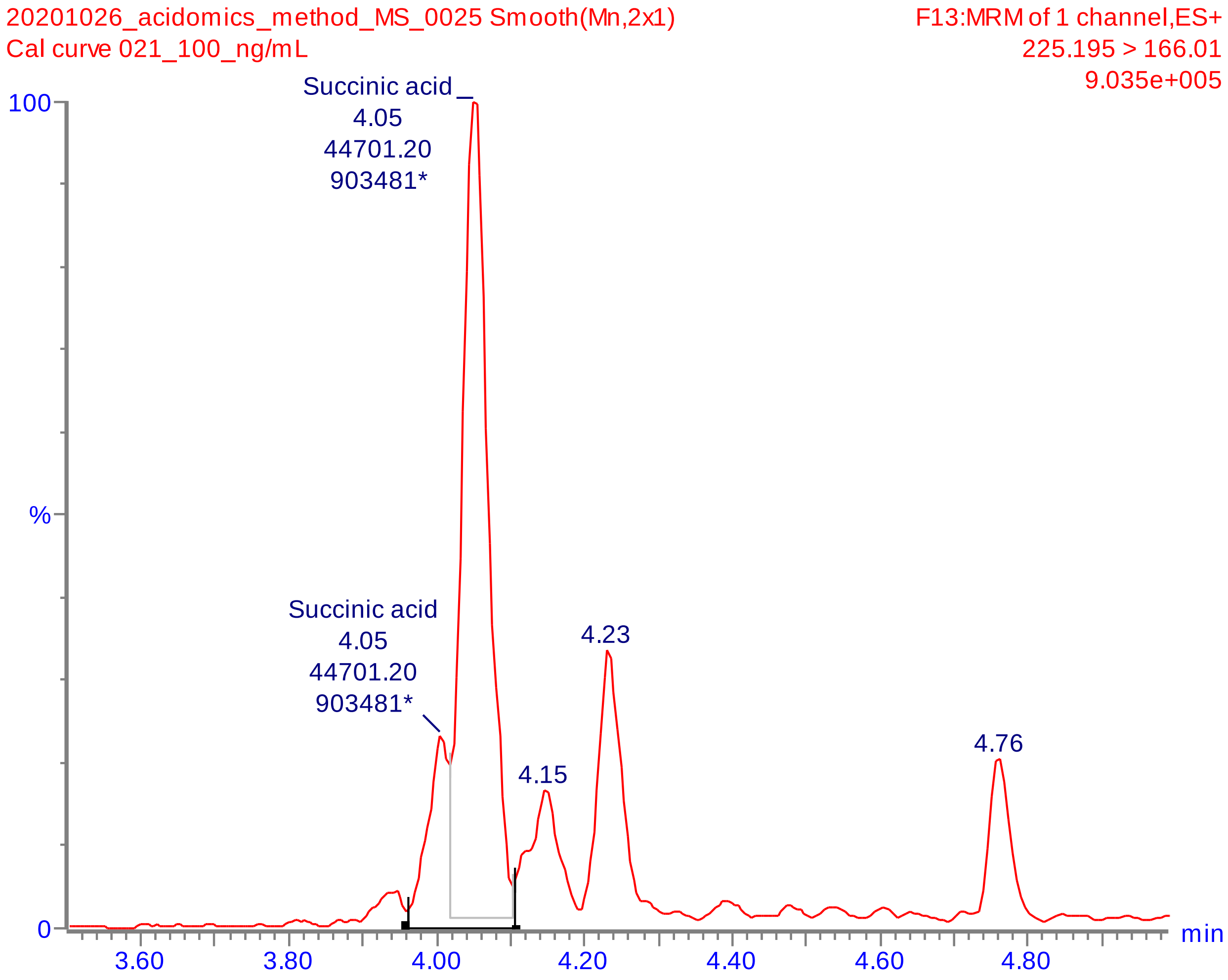
20201026_acidomics_method_MS_0025 Smooth(Mn,2x1)
Cal curve 021_100_ng/mL
F13:MRM of 1 channel,ES+
225.195 > 166.01
9.035e+005
Succinic acid
4.05
44701.20
903481*
Succinic acid
4.05
44701.20
903481*
4.15
4.23
4.76
100
%
0
3.60
3.80
4.00
4.20
4.40
4.60
4.80
min

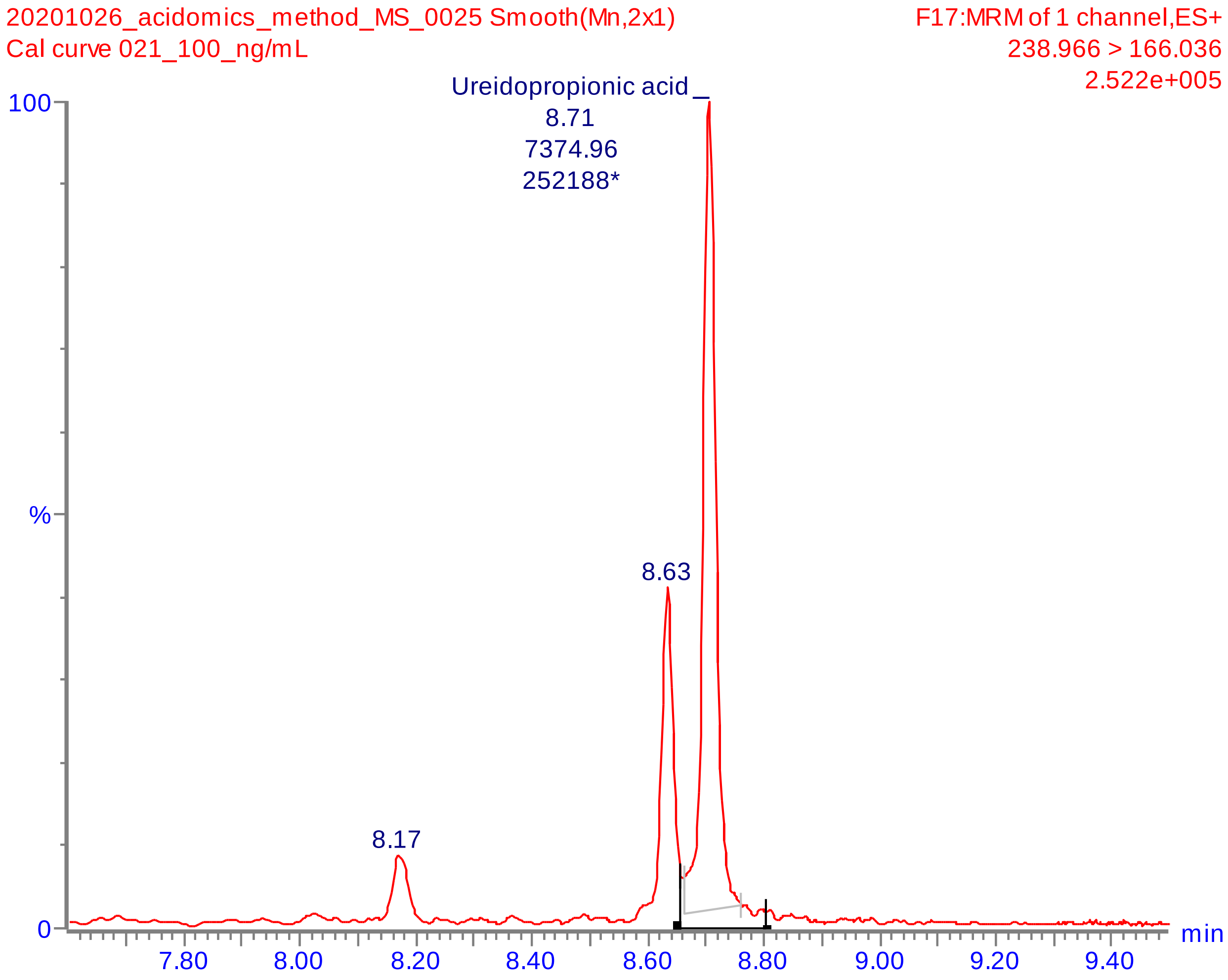
20201026_acidomics_method_MS_0025 Smooth(Mn,2x1)
Cal curve 021_100_ng/mL
F17:MRM of 1 channel,ES+
238.966 > 166.036
2.522e+005
Ureidopropionic acid
8.71
7374.96
252188*
100
%
0
8.63
8.17
7.80
8.00
8.20
8.40
8.60
8.80
9.00
9.20
9.40
min

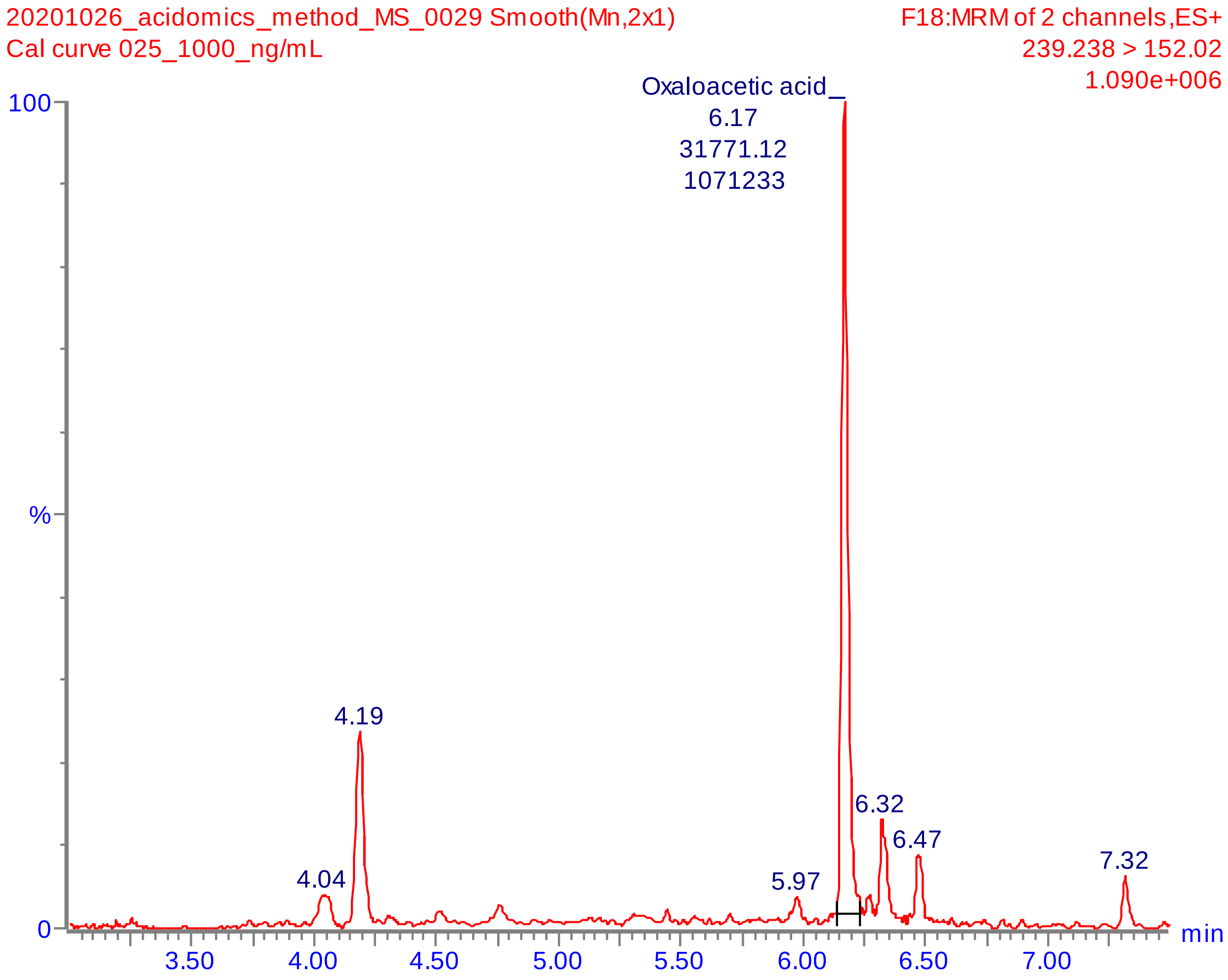

20201026_acidomics_method_MS_0029 Smooth(Mn,2x1)
Cal curve 025_1000_ng/mL
F18:MRM of 2 channels,ES+
239.238 > 152.02
1.090e+006
Oxaloacetic acid
6.17
31771.12
1071233
100
%
0
4.19
4.04
5.97
6.32
6.47
7.32
3.50
4.00
4.50
5.00
5.50
6.00
6.50
7.00
min

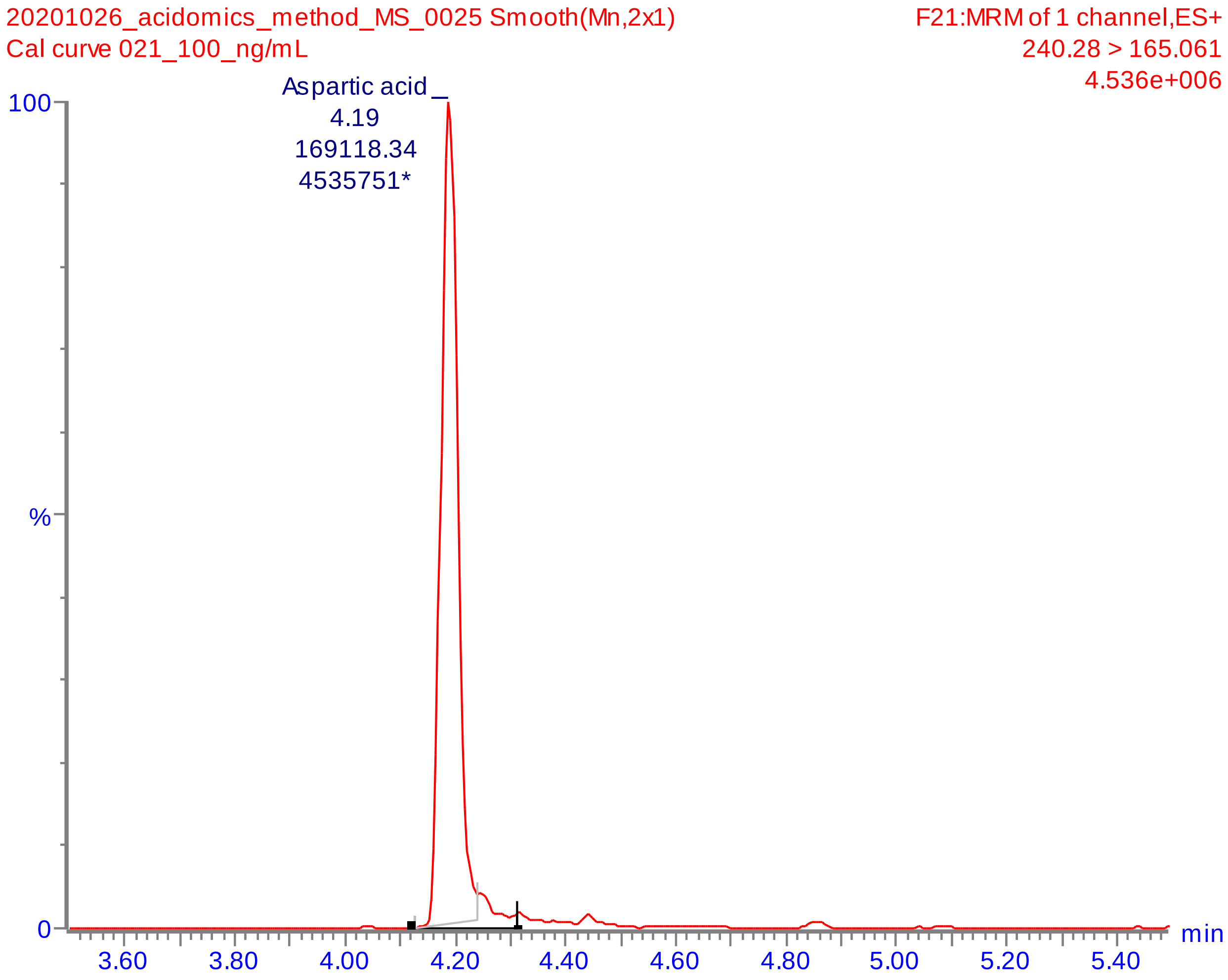

20201026_acidomics_method_MS_0025 Smooth(Mn,2x1)
Cal curve 021_100_ng/mL
F21:MRM of 1 channel,ES+
240.28 > 165.061
4.536e+006
Aspartic acid
4.19
169118.34
4535751*
100
%
0
3.60
3.80
4.00
4.20
4.40
4.60
4.80
5.00
5.20
5.40
min

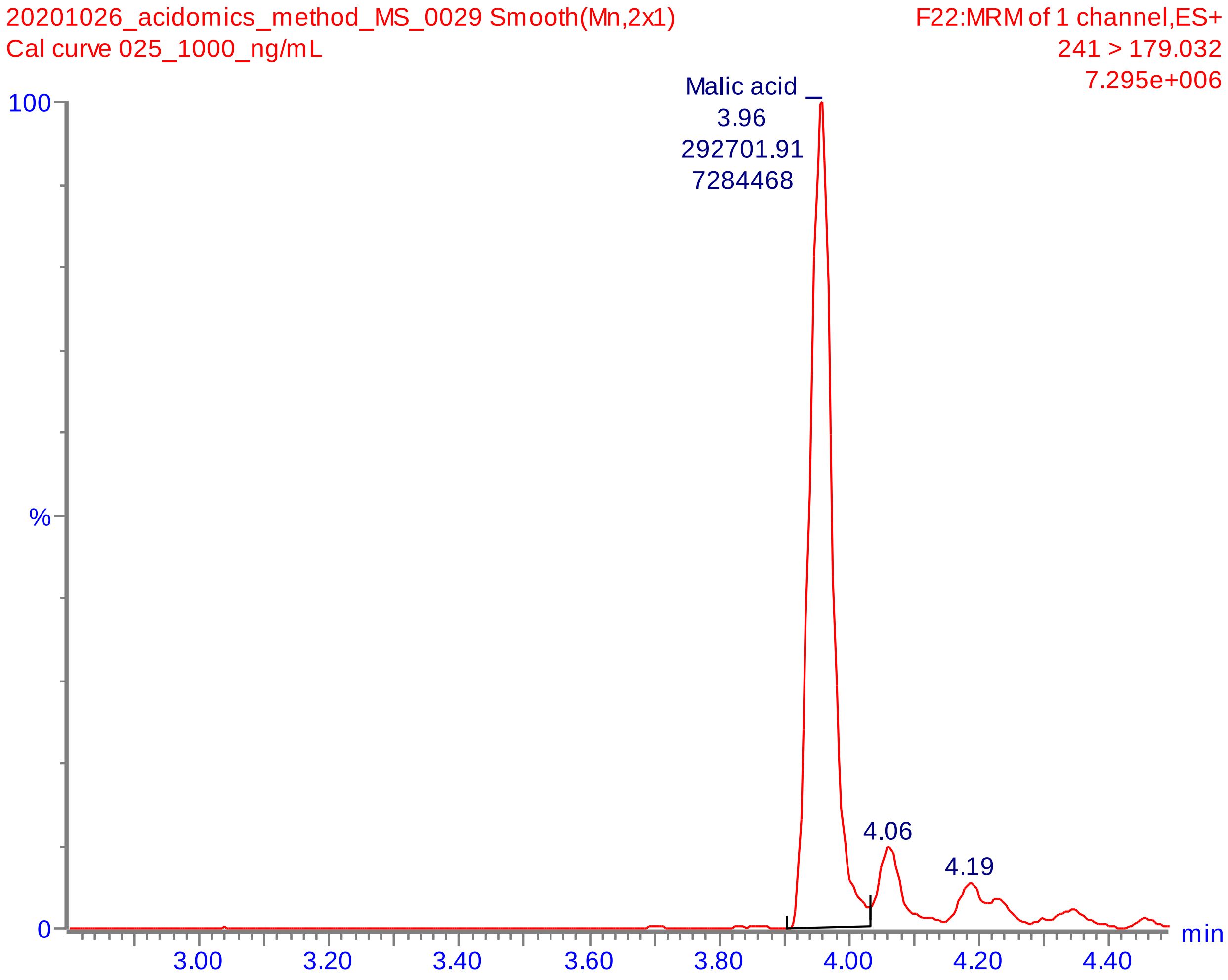
20201026_acidomics_method_MS_0029 Smooth(Mn,2x1)
Cal curve 025_1000_ng/mL
F22:MRM of 1 channel,ES+
241 > 179.032
7.295e+006
Malic acid
3.96
292701.91
7284468
4.06
4.19
100
%
0
3.00
3.20
3.40
3.60
3.80
4.00
4.20
4.40
min

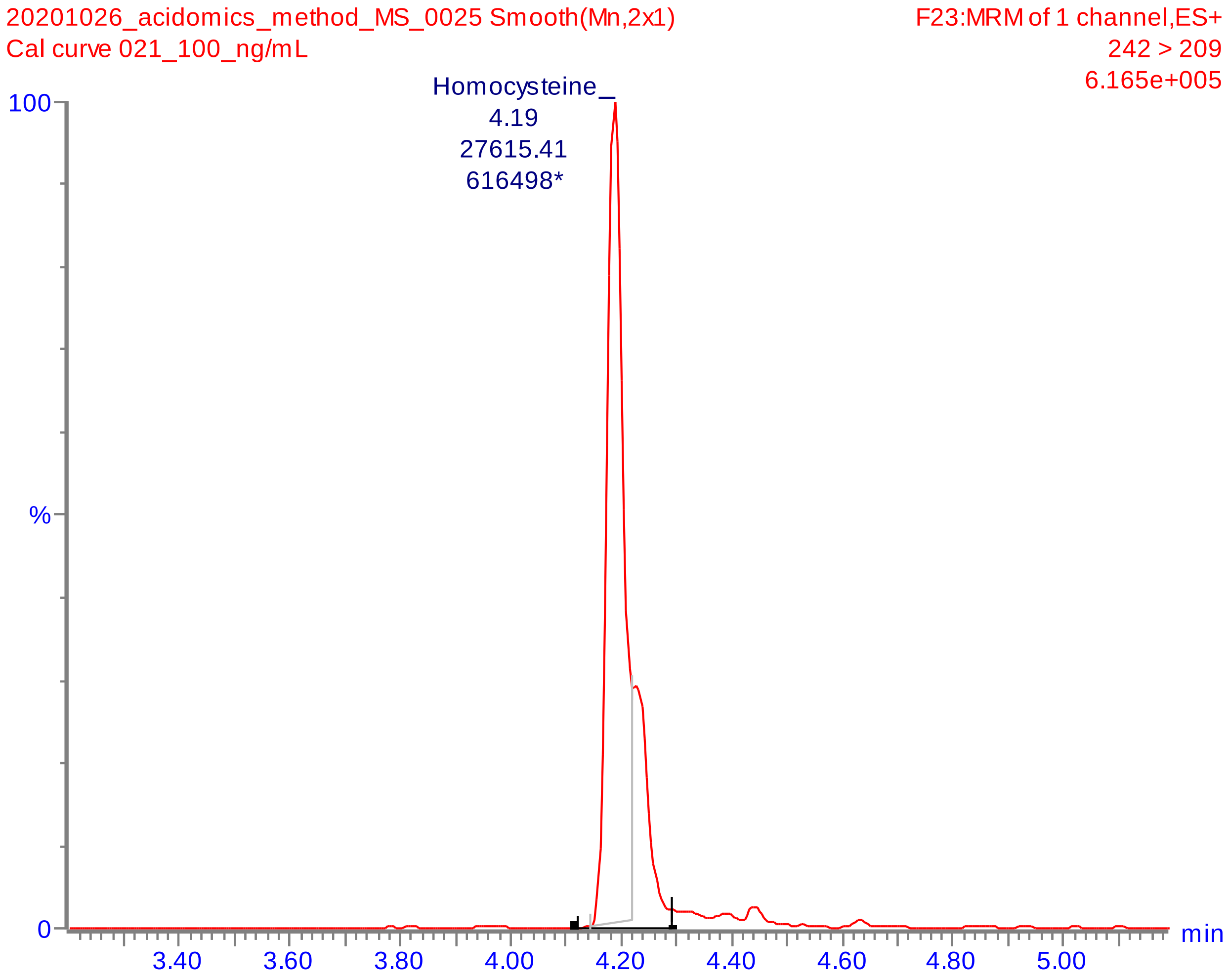

20201026_acidomics_method_MS_0025 Smooth(Mn,2x1)
Cal curve 021_100_ng/mL
F23:MRM of 1 channel,ES+
242 > 209
6.165e+005
Homocysteine_
4.19
27615.41
616498*
100
%
0
3.40
3.60
3.80
4.00
4.20
4.40
4.60
4.80
5.00
min

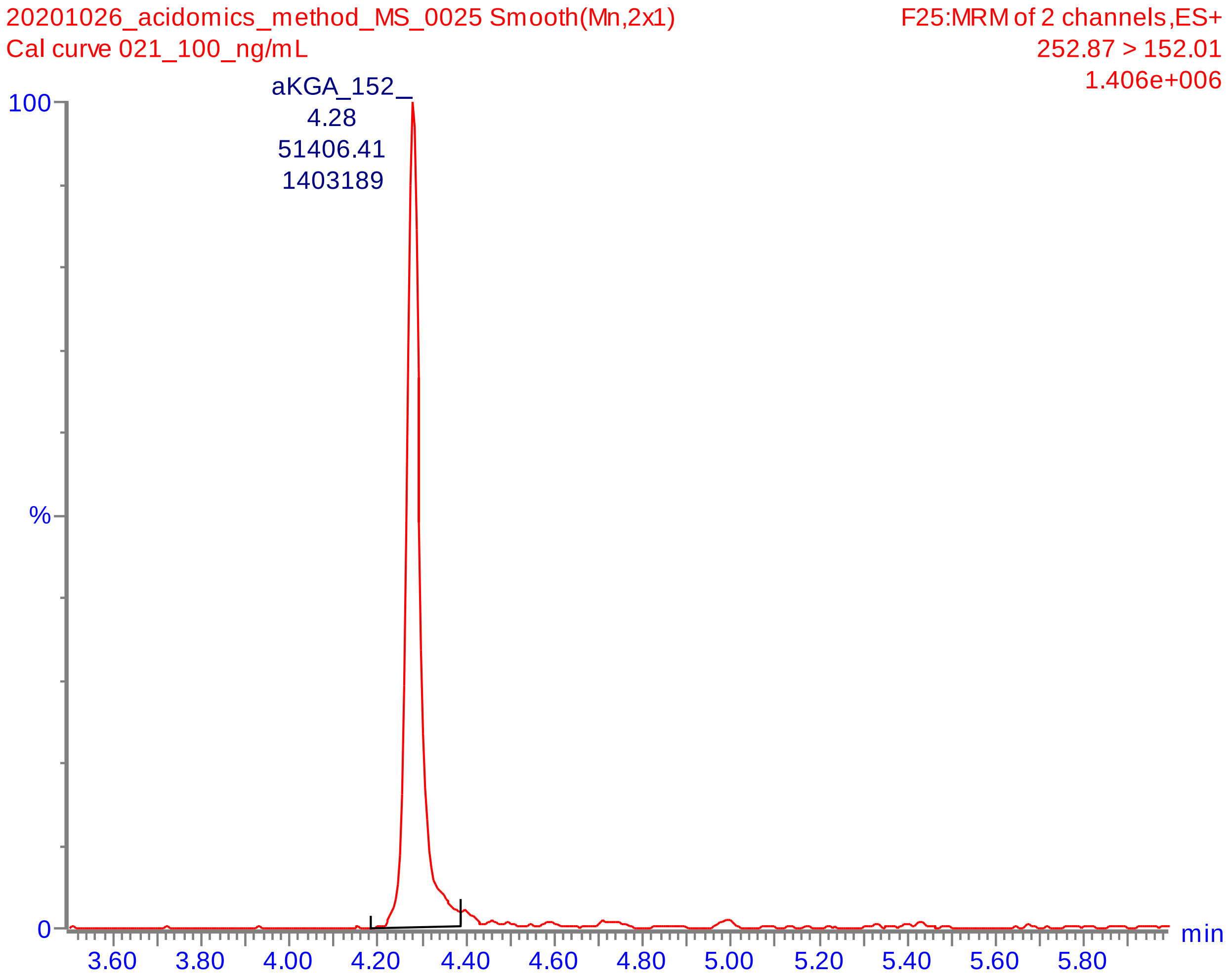

20201026_acidomics_method_MS_0025 Smooth(Mn,2x1)
Cal curve 021_100_ng/mL
F25:MRM of 2 channels,ES+
252.87 > 152.01
1.406e+006
aKGA_152_
4.28
51406.41
1403189
100
%
0
3.60
3.80
4.00
4.20
4.40
4.60
4.80
5.00
5.20
5.40
5.60
5.80
min

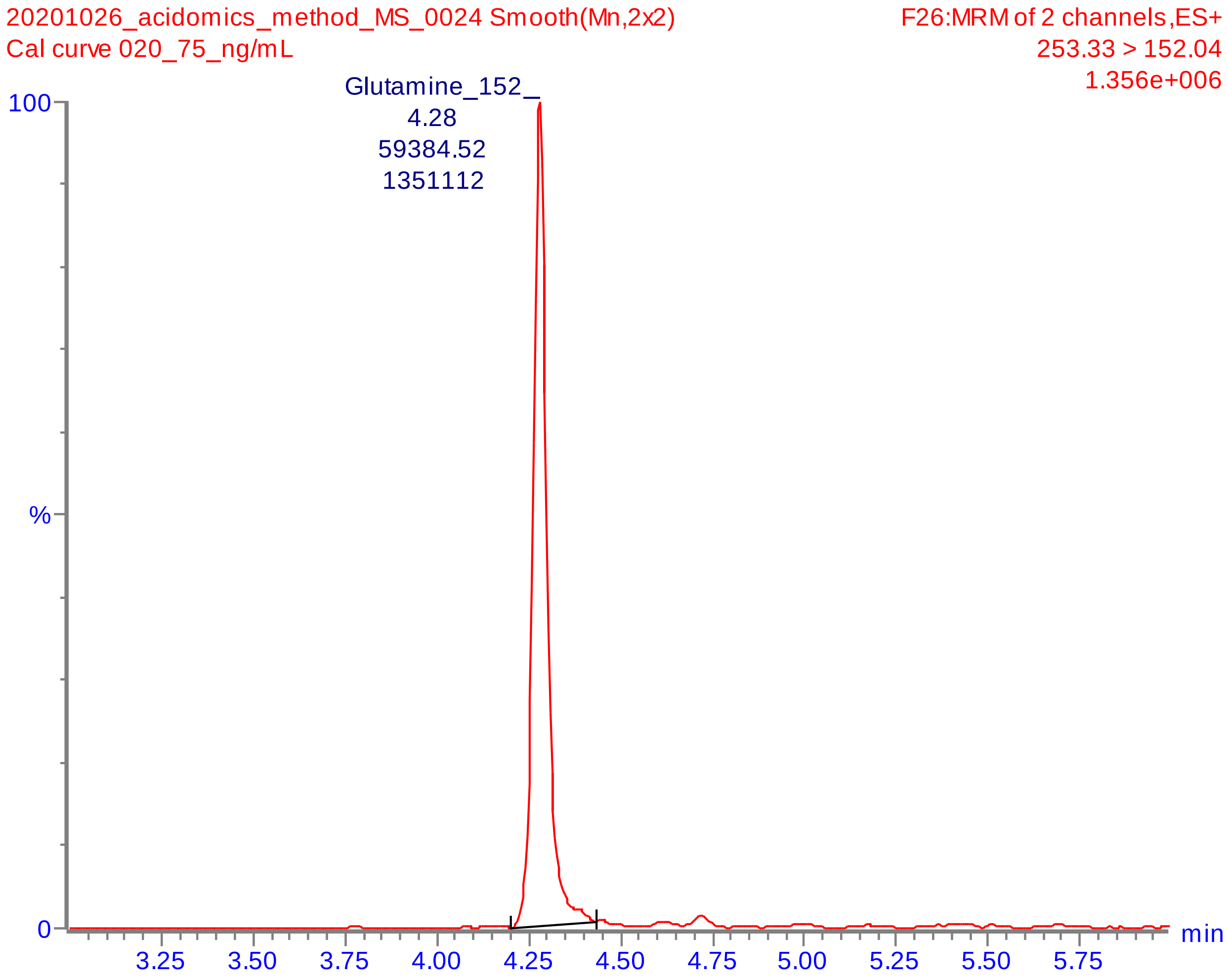

20201026_acidomics_method_MS_0024 Smooth(Mn,2x2)
Cal curve 020_75_ng/mL
F26:MRM of 2 channels,ES+
253.33 > 152.04
1.356e+006
Glutamine_152_
4.28
59384.52
1351112
100
%
0
3.25
3.50
3.75
4.00
4.25
4.50
4.75
5.00
5.25
5.50
5.75
min

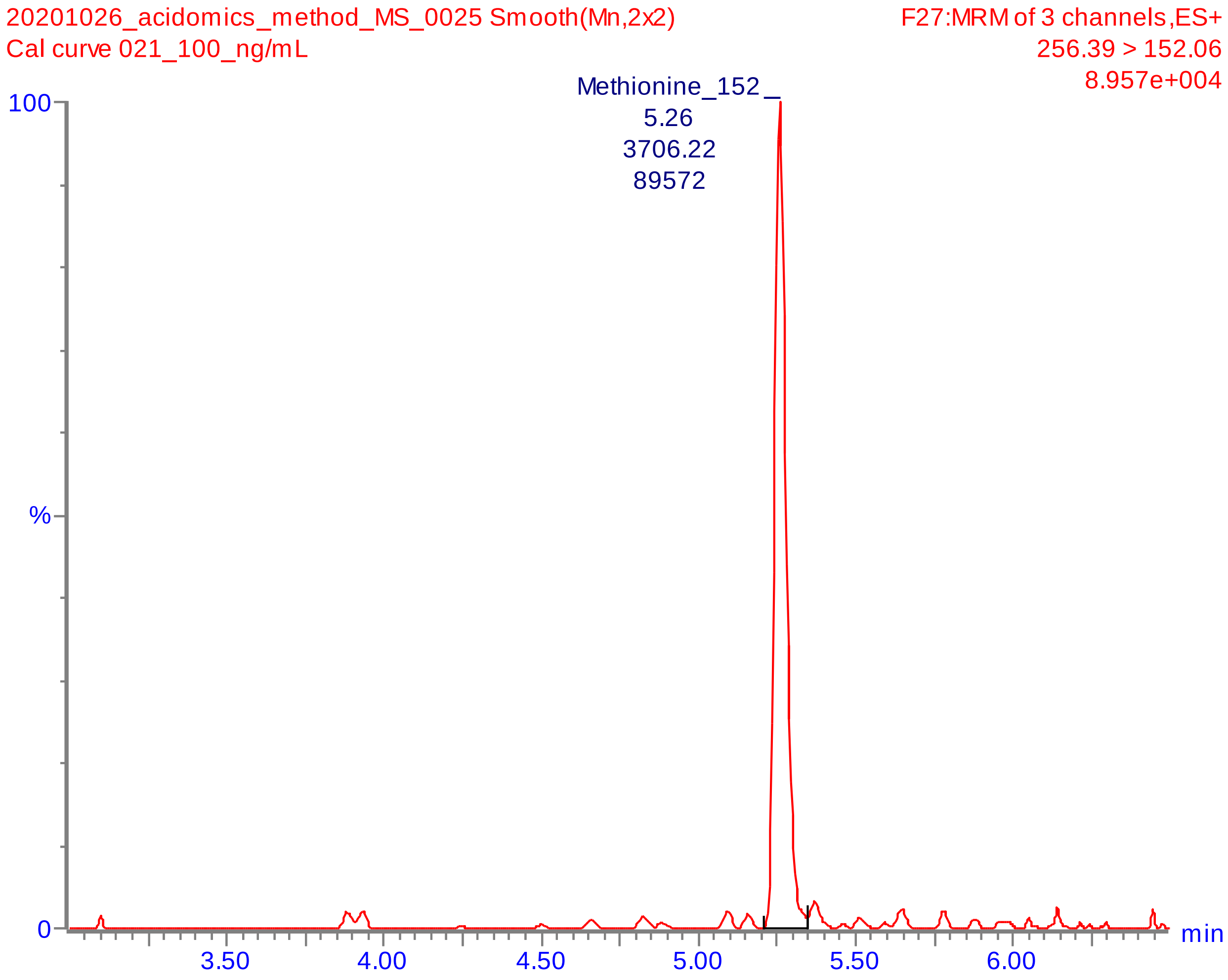
20201026_acidomics_method_MS_0025 Smooth(Mn,2x2)
Cal curve 021_100_ng/mL
F27:MRM of 3 channels,ES+
256.39 > 152.06
8.957e+004
Methionine_152
5.26
3706.22
89572
100
%
0
3.50
4.00
4.50
5.00
5.50
6.00
min

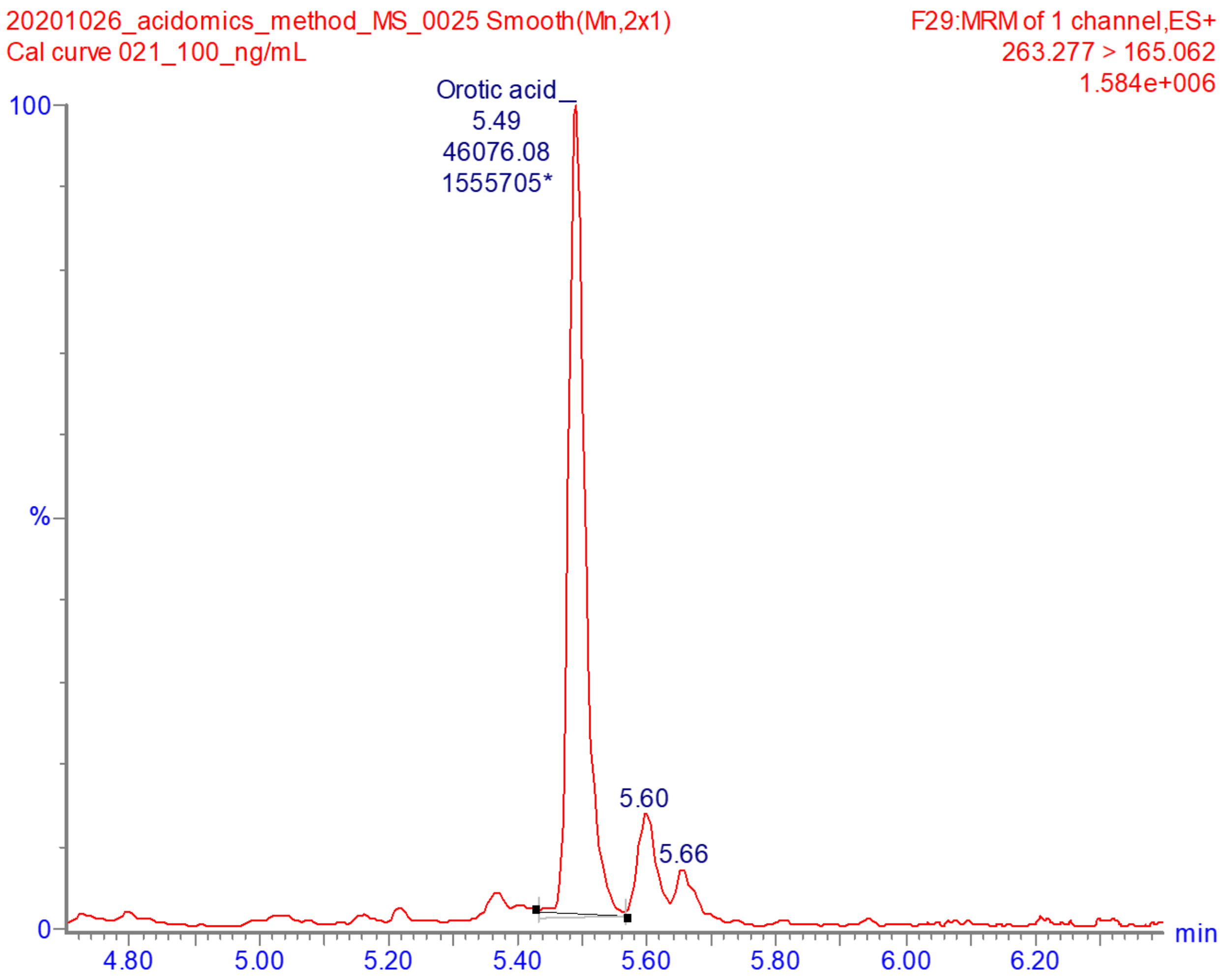
20201026_acidomics_method_MS_0025 Smooth(Mn,2x1)
Cal curve 021_100_ng/mL
F29:MRM of 1 channel,ES+
263.277 > 165.062
1.584e+006
Orotic acid
5.49
46076.08
1555705*
5.60
5.66
100
%
0
4.80
5.00
5.20
5.40
5.60
5.80
6.00
6.20
min

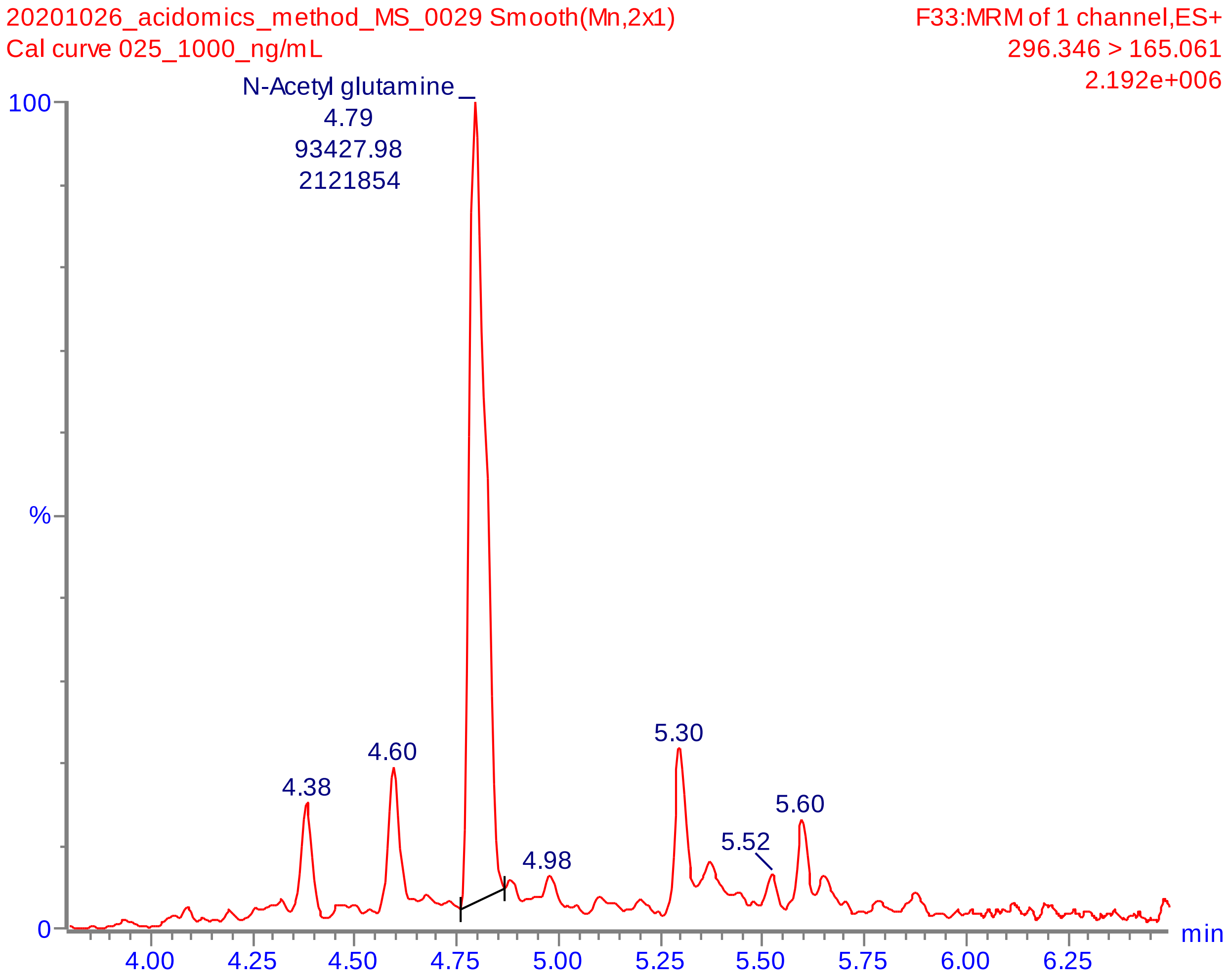

20201026_acidomics_method_MS_0029 Smooth(Mn,2x1)
Cal curve 025_1000_ng/mL
F33:MRM of 1 channel,ES+
296.346 > 165.061
2.192e+006
N-Acetyl glutamine
4.79
93427.98
2121854
100
%
0
4.38
4.60
4.98
5.30
5.52
5.60
4.00
4.25
4.50
4.75
5.00
5.25
5.50
5.75
6.00
6.25
min

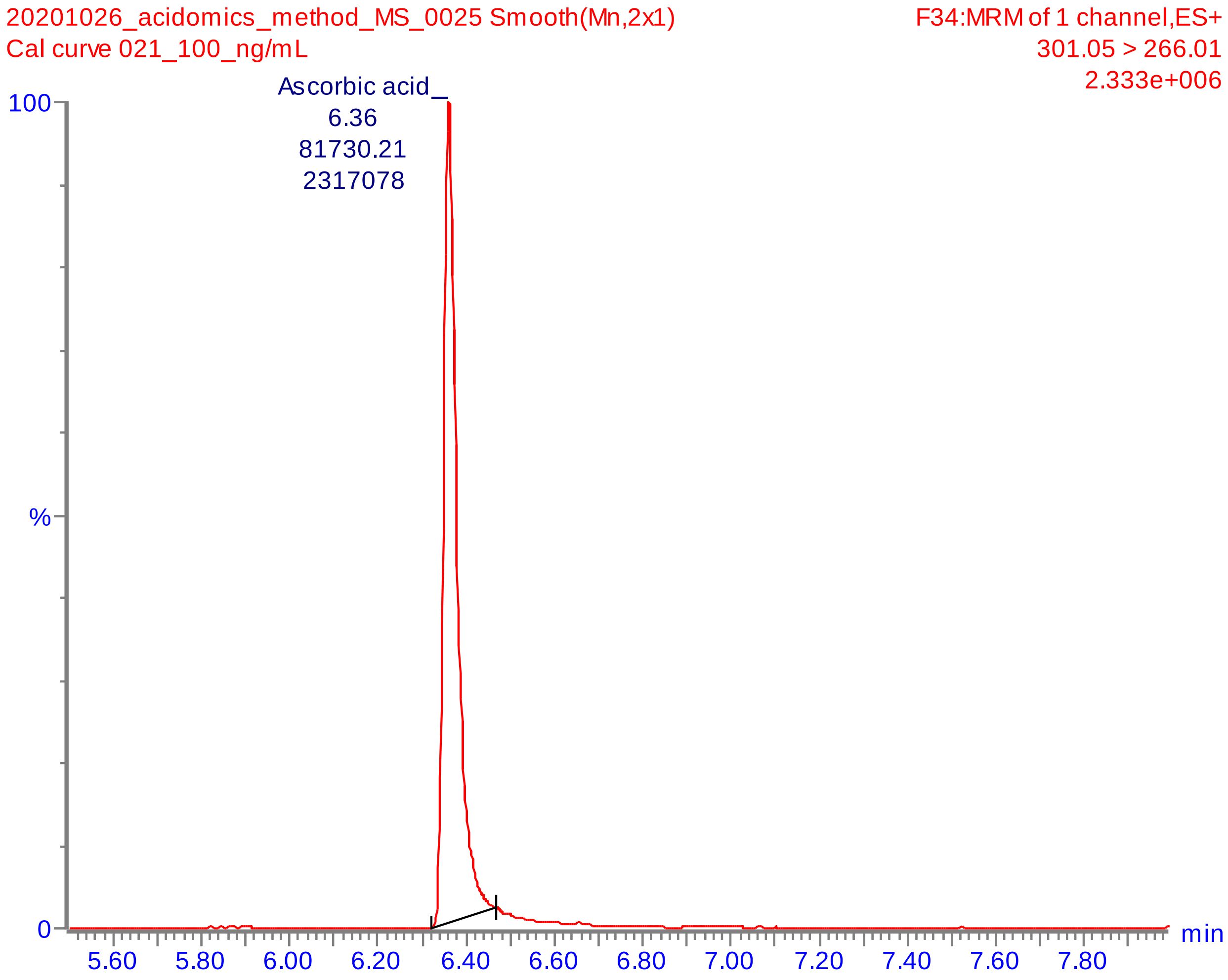
20201026_acidomics_method_MS_0025 Smooth(Mn,2x1)
Cal curve 021_100_ng/mL
F34:MRM of 1 channel,ES+
301.05 > 266.01
2.333e+006
Ascorbic acid_
6.36
81730.21
2317078
100
%
0
min
5.60
5.80
6.00
6.20
6.40
6.60
6.80
7.00
7.20
7.40
7.60
7.80

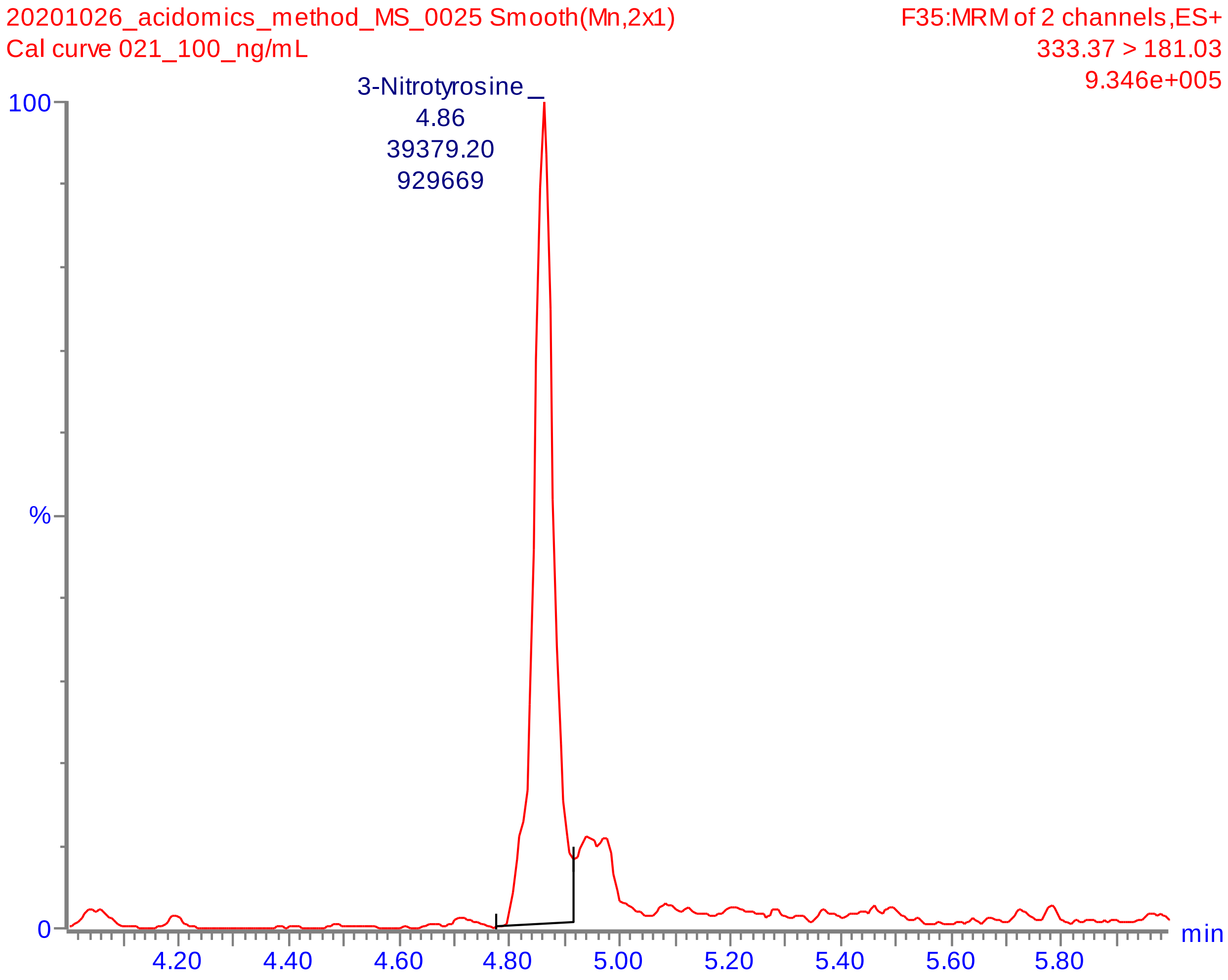
20201026_acidomics_method_MS_0025 Smooth(Mn,2x1)
Cal curve 021_100_ng/mL
F35:MRM of 2 channels,ES+
333.37 > 181.03
9.346e+005
3-Nitrotyrosine
4.86
39379.20
929669
100
%
0
4.20
4.40
4.60
4.80
5.00
5.20
5.40
5.60
5.80
min

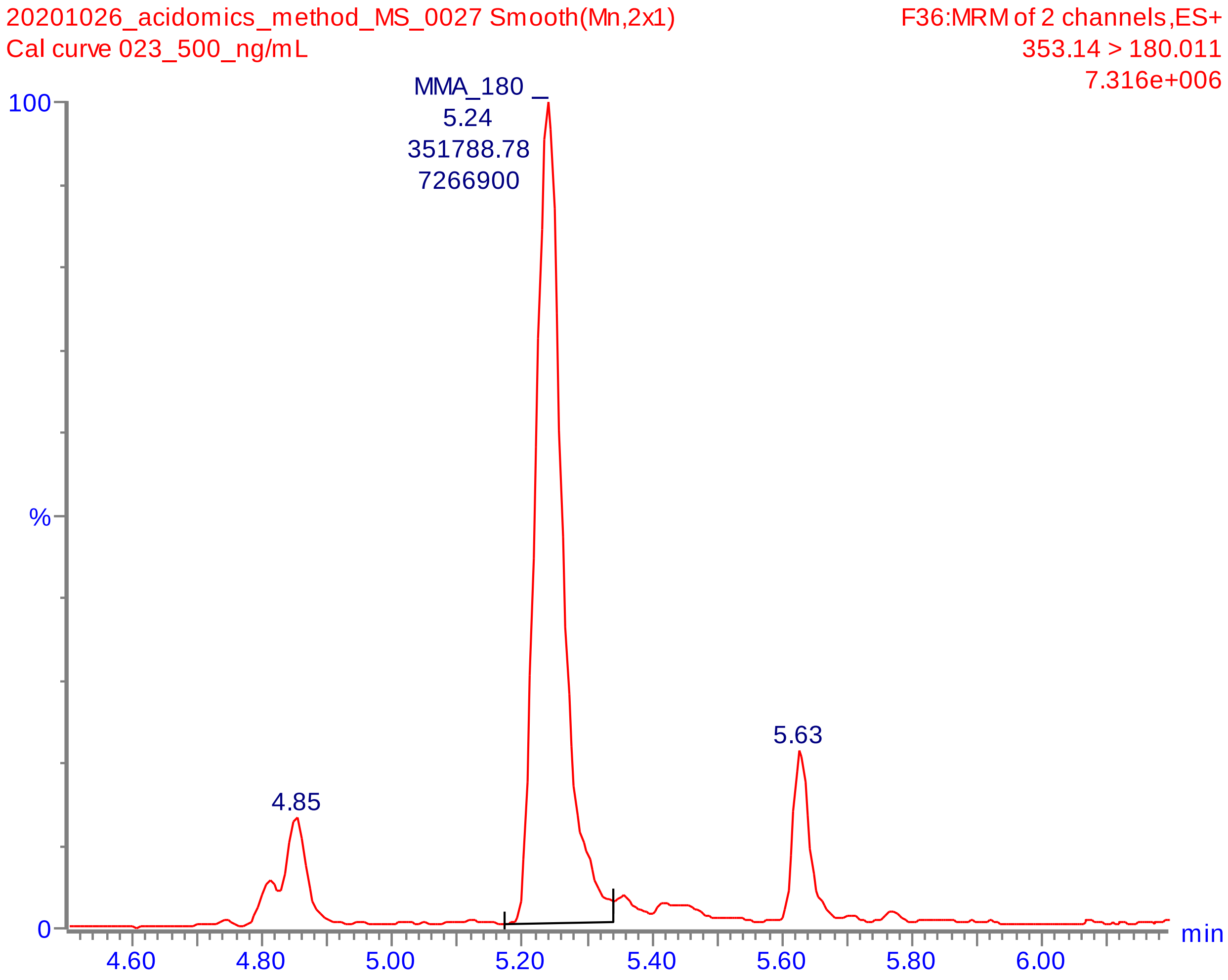

20201026_acidomics_method_MS_0027 Smooth(Mn,2x1)
Cal curve 023_500_ng/mL
F36:MRM of 2 channels,ES+
353.14 > 180.011
7.316e+006
MMA_180
5.24
351788.78
7266900
4.85
5.63
100
%
0
4.60
4.80
5.00
5.20
5.40
5.60
5.80
6.00
min

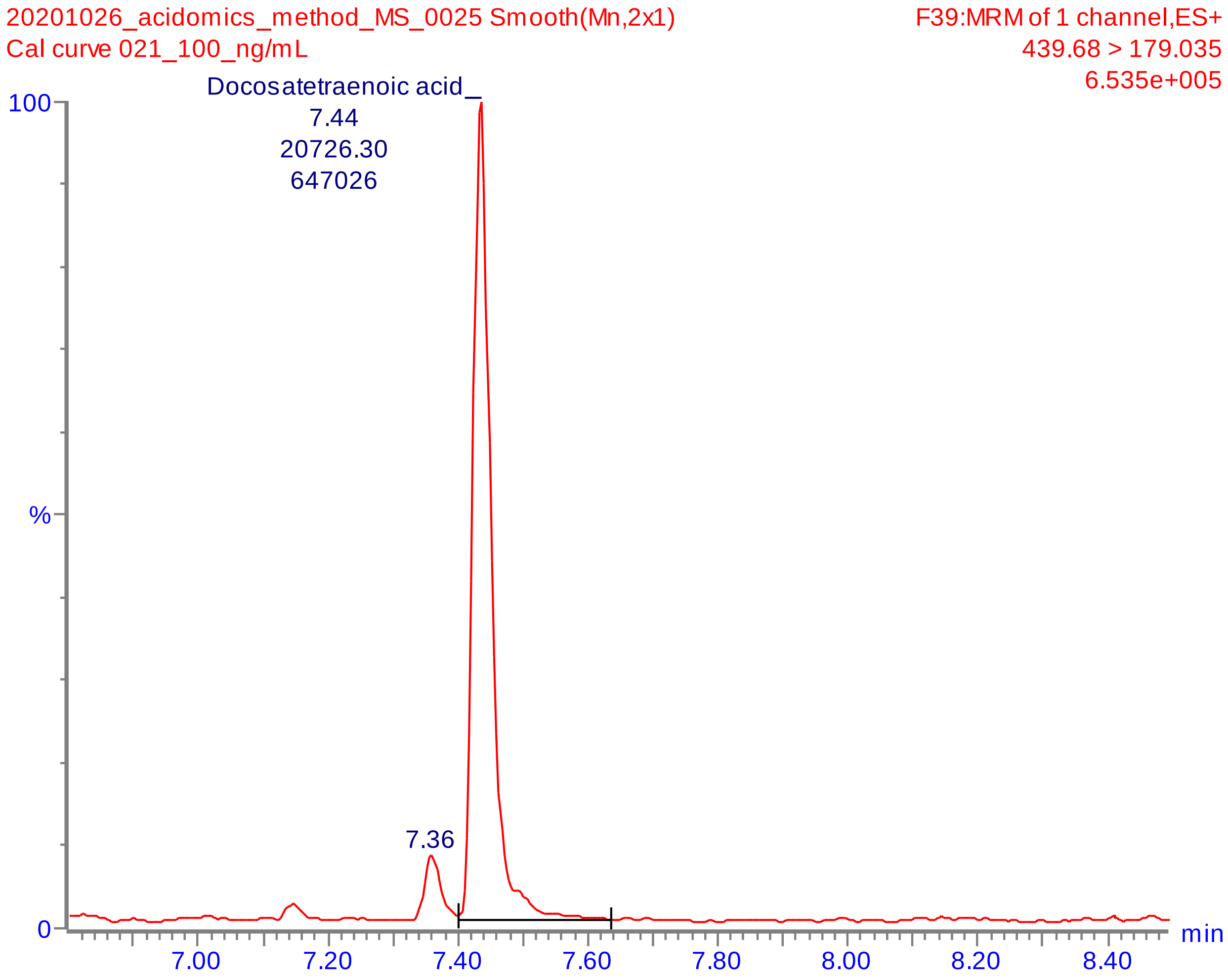

20201026_acidomics_method_MS_0025 Smooth(Mn,2x1)
Cal curve 021_100_ng/mL
F39:MRM of 1 channel,ES+
439.68 > 179.035
6.535e+005
Docosatetraenoic acid_
7.44
20726.30
647026
7.36
100
%
0
7.00
7.20
7.40
7.60
7.80
8.00
8.20
8.40
min

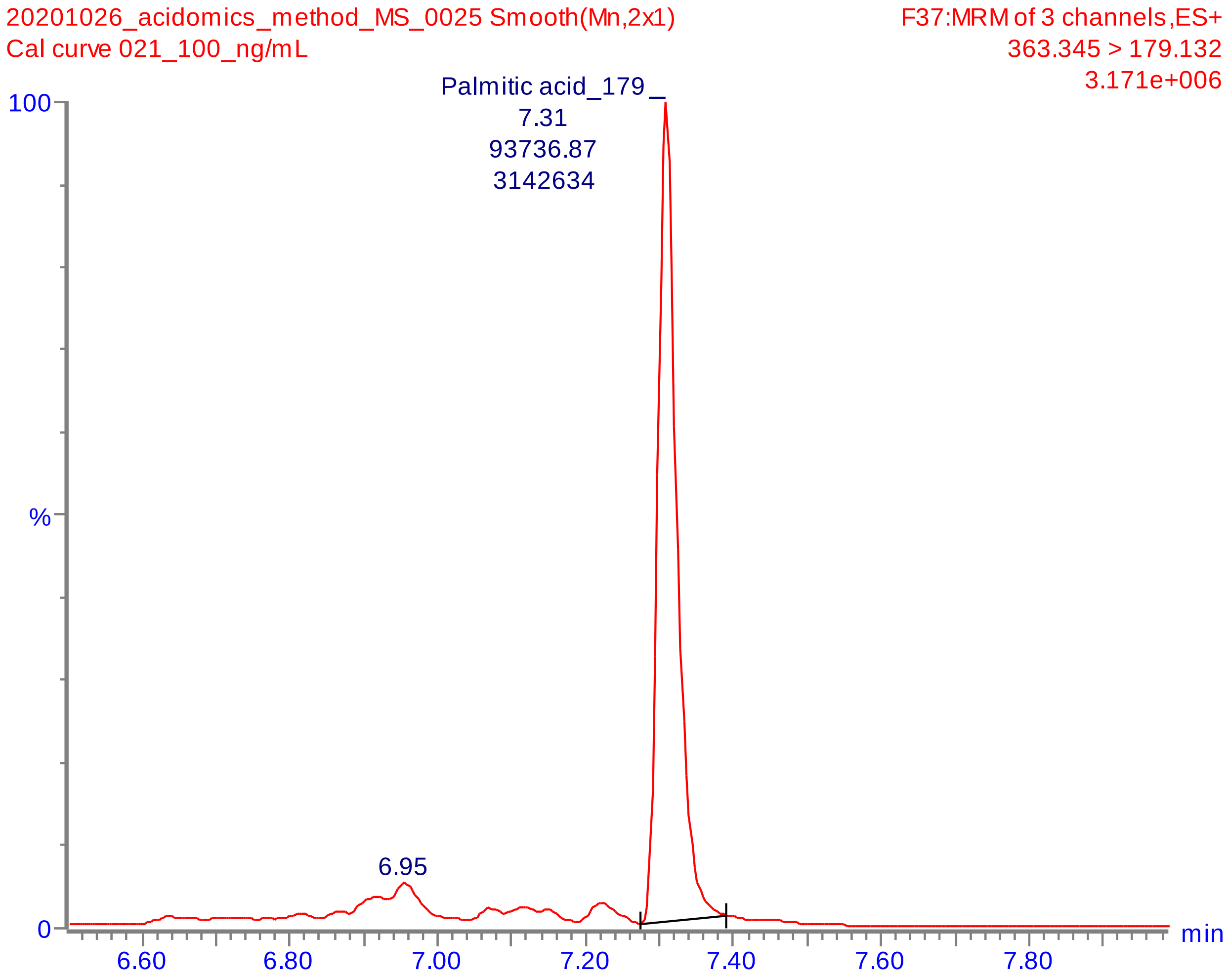

20201026_acidomics_method_MS_0025 Smooth(Mn,2x1)
Cal curve 021_100_ng/mL
F37:MRM of 3 channels,ES+
363.345 > 179.132
3.171e+006
Palmitic acid_179
7.31
93736.87
3142634
100
%
0
6.95
6.60
6.80
7.00
7.20
7.40
7.60
7.80
min

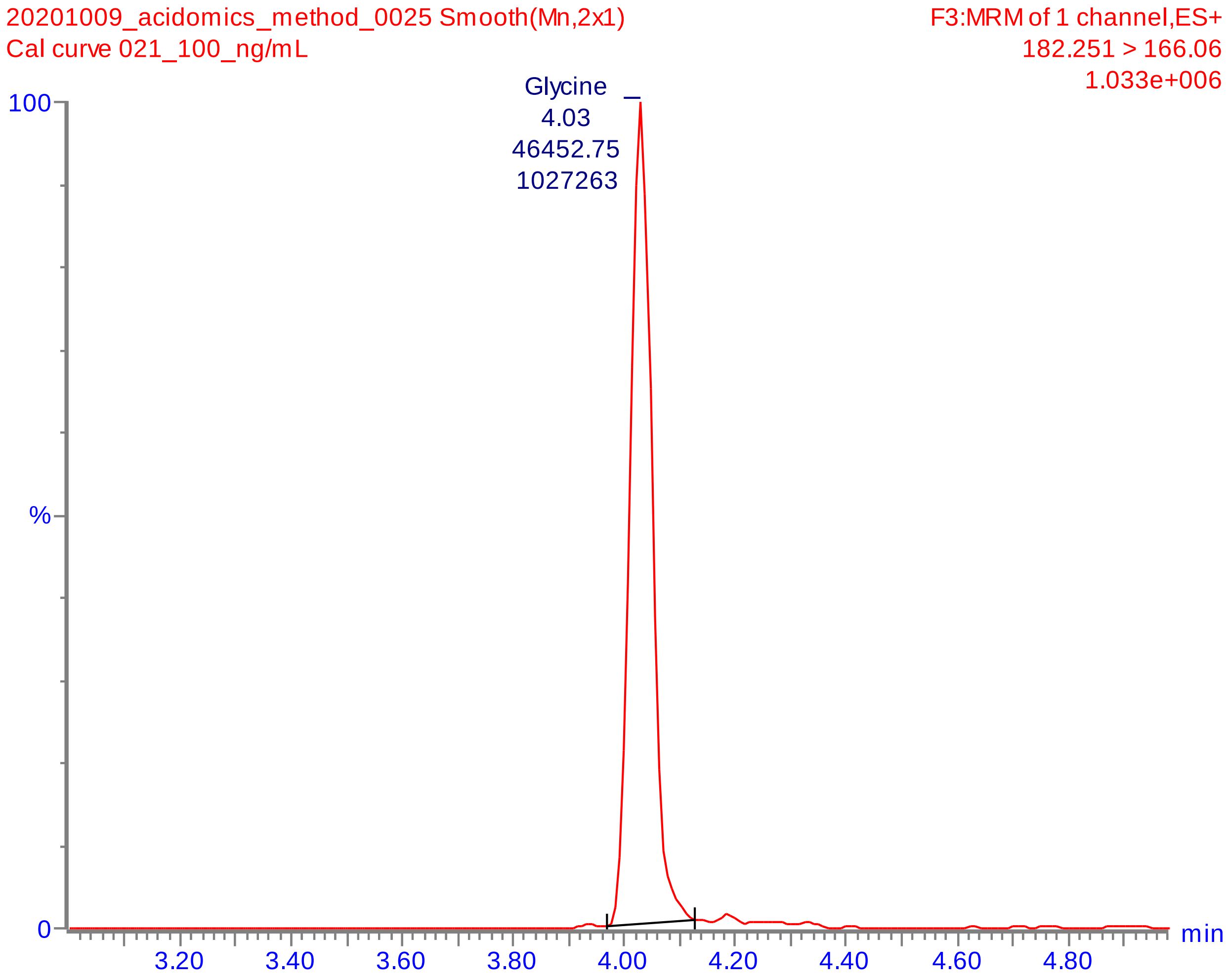

20201009_acidomics_method_0025 Smooth(Mn,2x1)
Cal curve 021_100_ng/mL
F3:MRM of 1 channel,ES+
182.251 > 166.06
1.033e+006
Glycine
4.03
46452.75
1027263
100
%
0
3.20
3.40
3.60
3.80
4.00
4.20
4.40
4.60
4.80
min

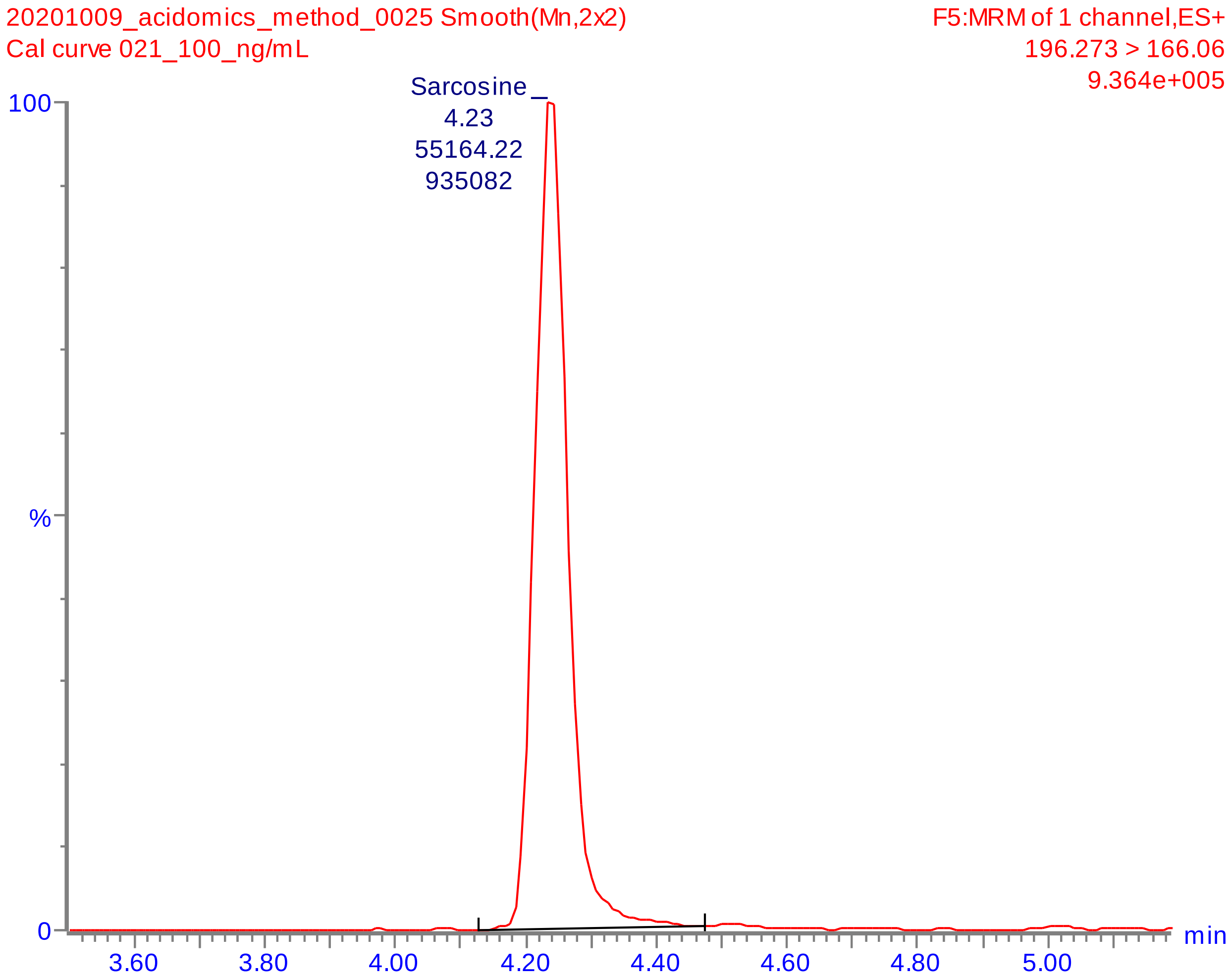

20201009_acidomics_method_0025 Smooth(Mn,2x2)
Cal curve 021_100_ng/mL
F5:MRM of 1 channel,ES+
196.273 > 166.06
9.364e+005
Sarcosine
4.23
55164.22
935082
100
%
0
3.60
3.80
4.00
4.20
4.40
4.60
4.80
5.00
min

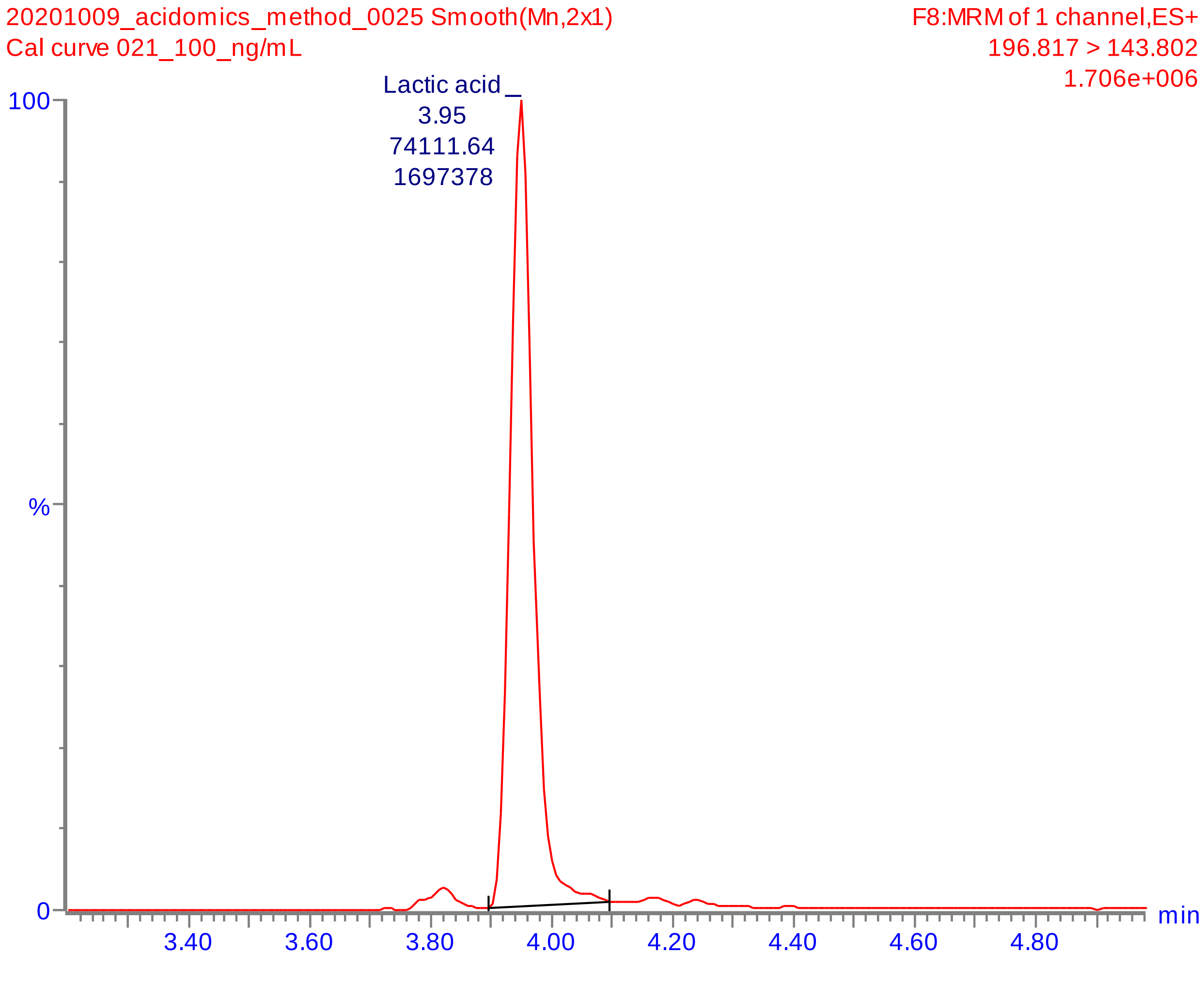

20201009_acidomics_method_0025 Smooth(Mn,2x1)
Cal curve 021_100_ng/mL
F8:MRM of 1 channel,ES+
196.817 > 143.802
1.706e+006
Lactic acid
3.95
74111.64
1697378
100
%
0
3.40
3.60
3.80
4.00
4.20
4.40
4.60
4.80
min

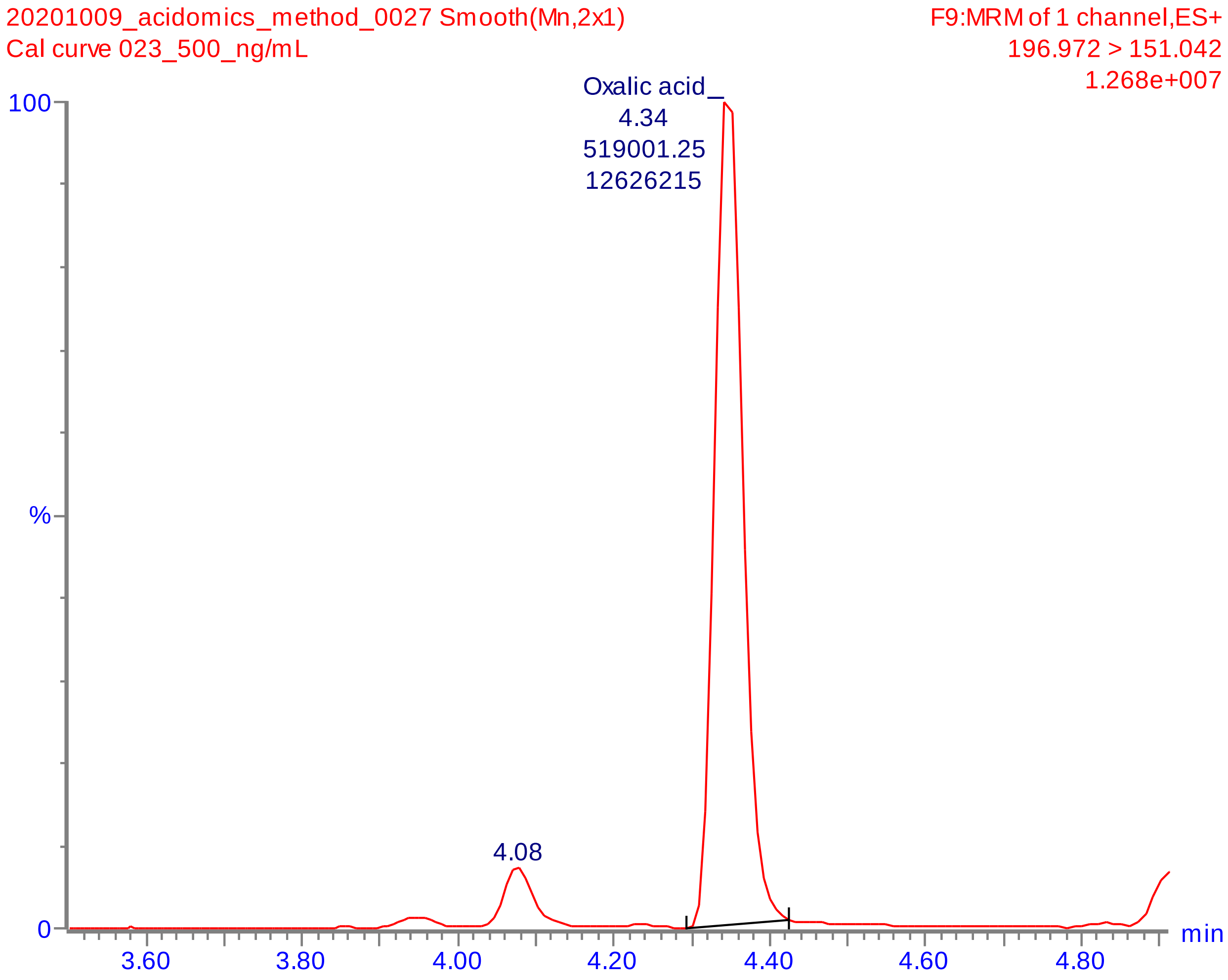

20201009_acidomics_method_0027 Smooth(Mn,2x1)
Cal curve 023_500_ng/mL
F9:MRM of 1 channel,ES+
196.972 > 151.042
1.268e+007
Oxalic acid_
4.34
519001.25
12626215
4.08
100
%
0
3.60
3.80
4.00
4.20
4.40
4.60
4.80
min

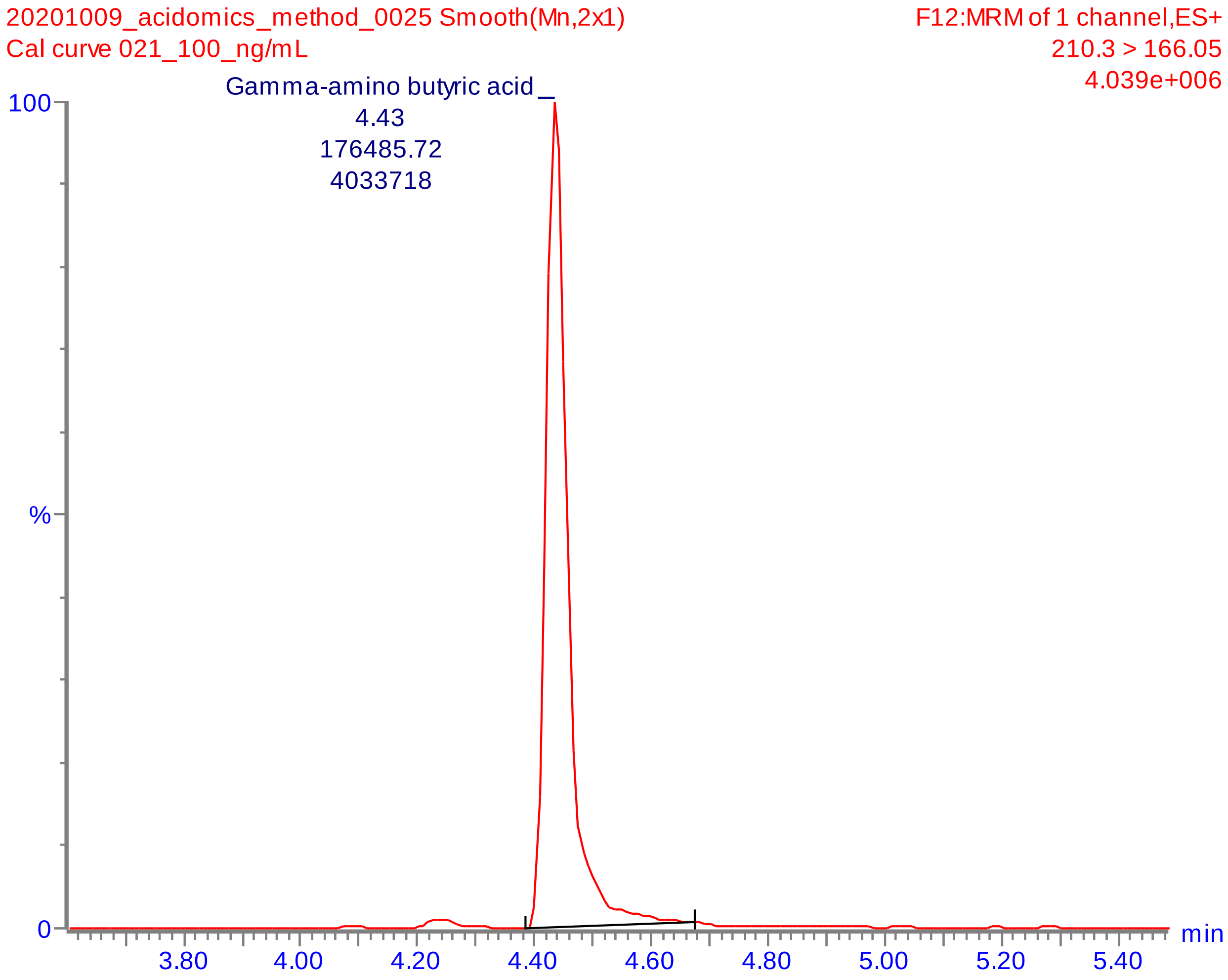

20201009_acidomics_method_0025 Smooth(Mn,2x1)
Cal curve 021_100_ng/mL
F12:MRM of 1 channel,ES+
210.3 > 166.05
4.039e+006
Gamma-amino butyric acid
4.43
176485.72
4033718
100
%
0
3.80
4.00
4.20
4.40
4.60
4.80
5.00
5.20
5.40
min

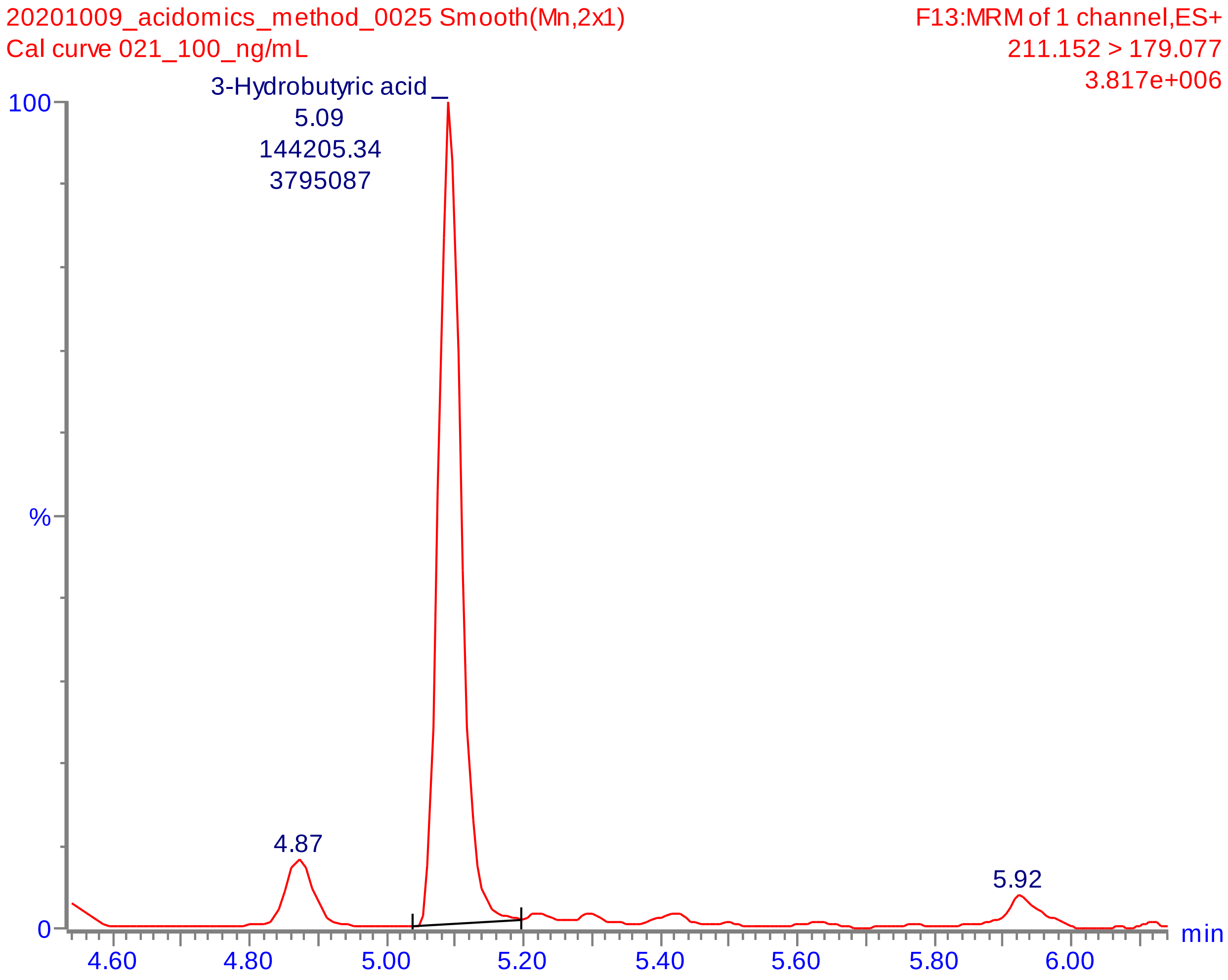

20201009_acidomics_method_0025 Smooth(Mn,2x1)
Cal curve 021_100_ng/mL
F13:MRM of 1 channel,ES+
211.152 > 179.077
3.817e+006
3-Hydrobutyric acid
5.09
144205.34
3795087
4.87
5.92
100
%
0
4.60
4.80
5.00
5.20
5.40
5.60
5.80
6.00
min

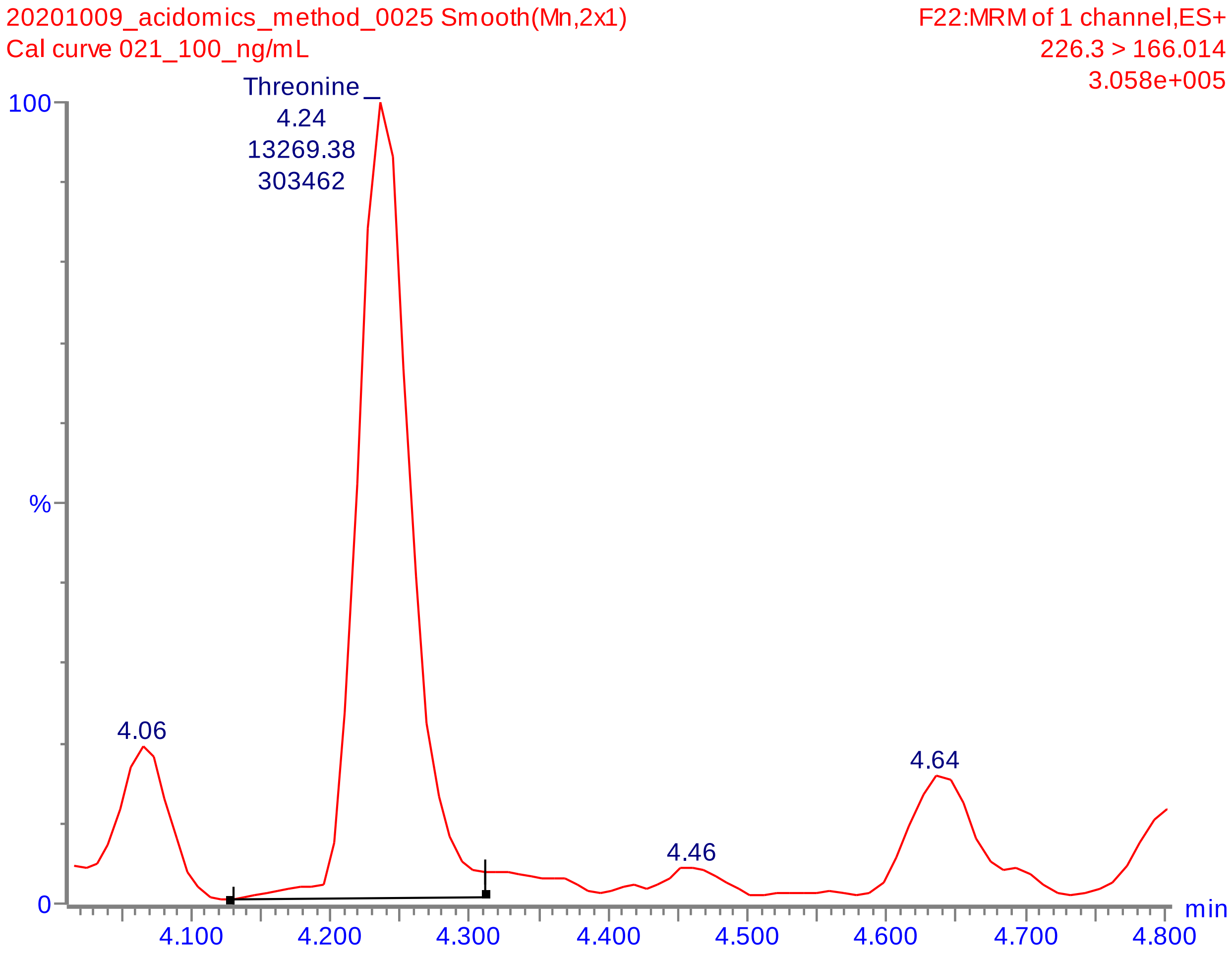
20201009_acidomics_method_0025 Smooth(Mn,2x1)
Cal curve 021_100_ng/mL
F22:MRM of 1 channel,ES+
226.3 > 166.014
3.058e+005
Threonine
4.24
13269.38
303462
4.06
4.46
4.64
100
%
0
4.100
4.200
4.300
4.400
4.500
4.600
4.700
4.800
min

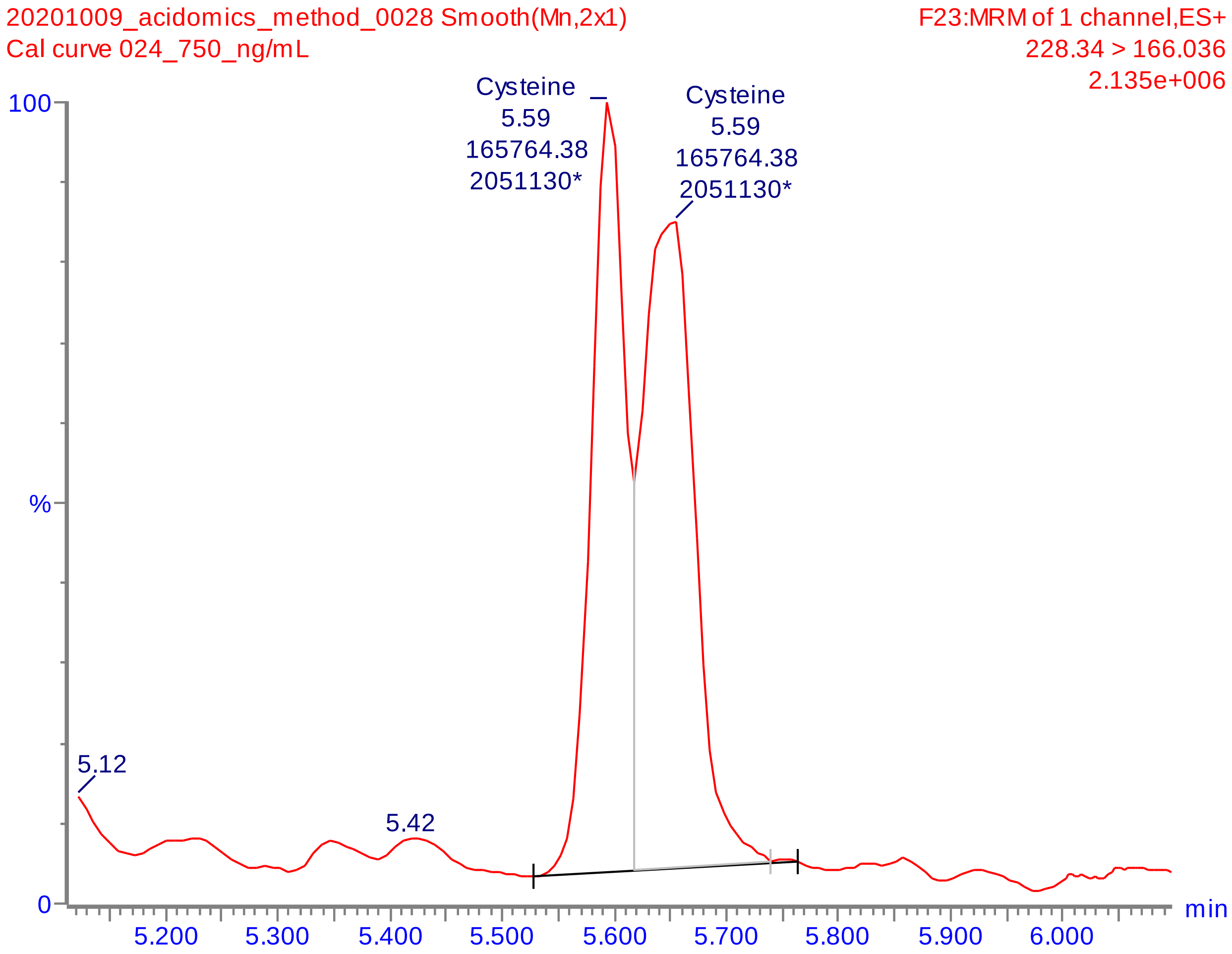

20201009_acidomics_method_0028 Smooth(Mn,2x1)
Cal curve 024_750_ng/mL
F23:MRM of 1 channel,ES+
228.34 > 166.036
2.135e+006
Cysteine
5.59
165764.38
2051130*
Cysteine
5.59
165764.38
2051130*
5.12
5.42
100
%
0
5.200
5.300
5.400
5.500
5.600
5.700
5.800
5.900
6.000
min

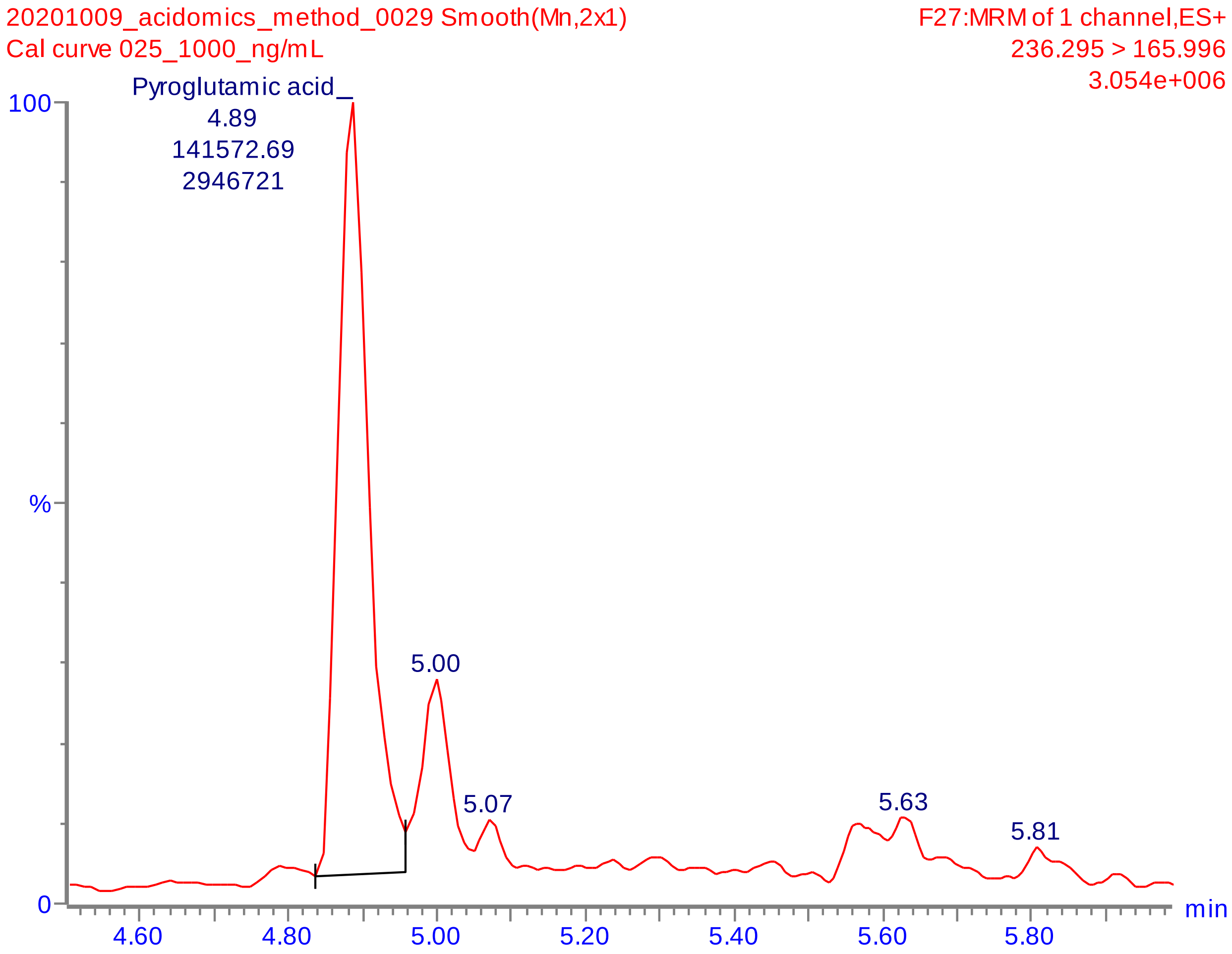
20201009_acidomics_method_0029 Smooth(Mn,2x1)
Cal curve 025_1000_ng/mL
F27:MRM of 1 channel,ES+
236.295 > 165.996
3.054e+006
Pyroglutamic acid
4.89
141572.69
2946721
5.00
5.07
5.63
5.81
100
%
0
4.60
4.80
5.00
5.20
5.40
5.60
5.80
min

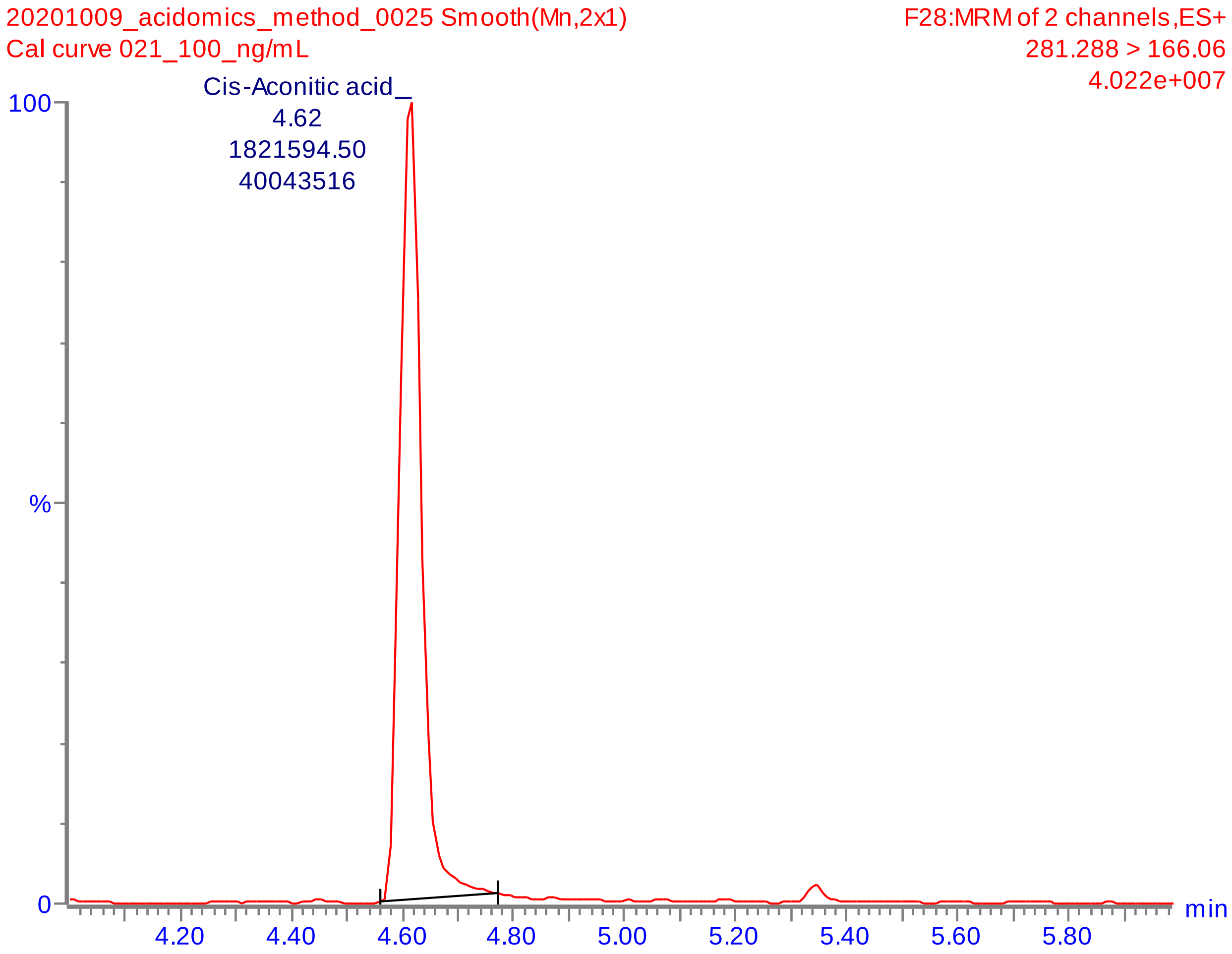
20201009_acidomics_method_0025 Smooth(Mn,2x1)
Cal curve 021_100_ng/mL
F28:MRM of 2 channels,ES+
281.288 > 166.06
4.022e+007
Cis-Aconitic acid
4.62
1821594.50
40043516
100
%
0
4.20
4.40
4.60
4.80
5.00
5.20
5.40
5.60
5.80
min

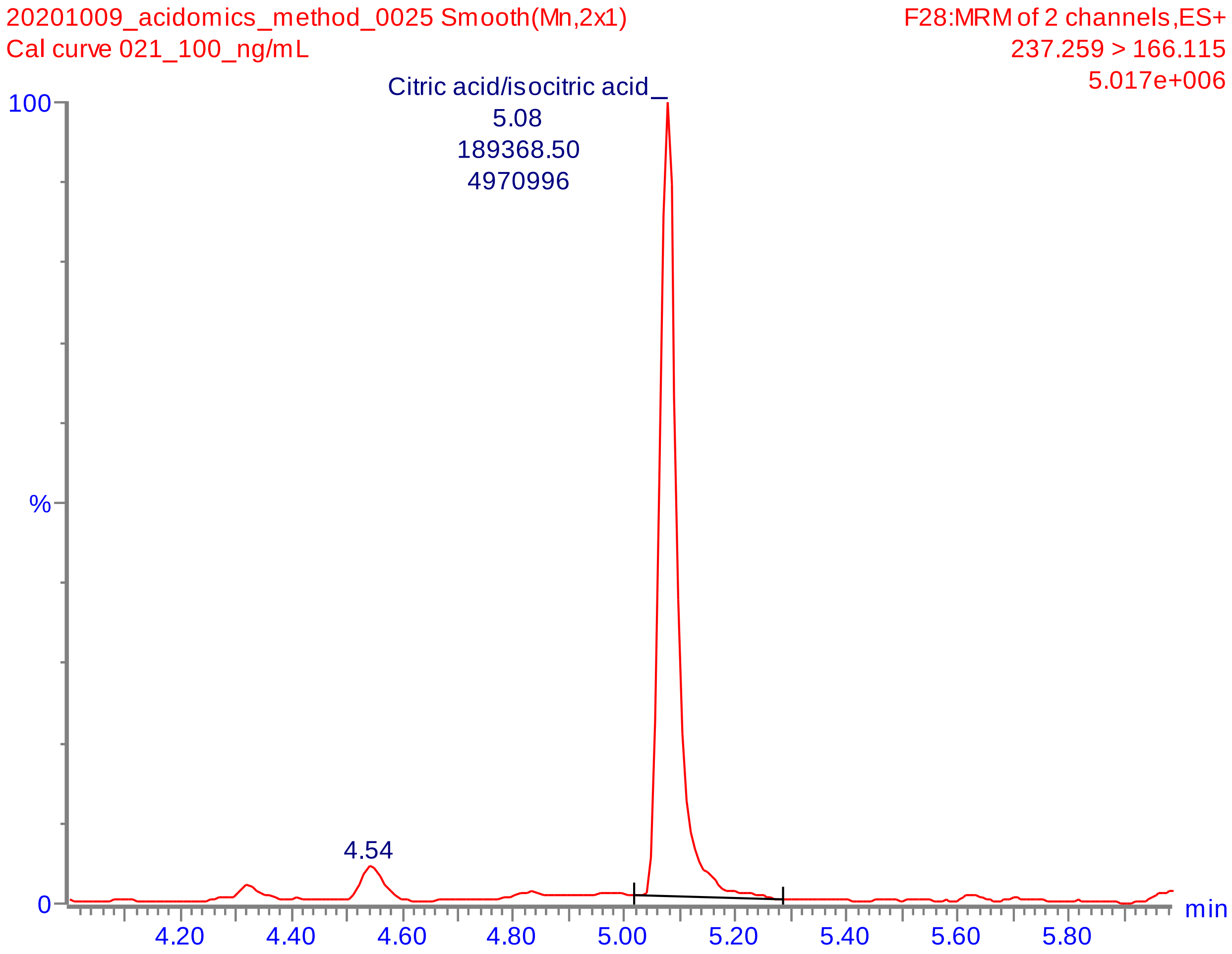

20201009_acidomics_method_0025 Smooth(Mn,2x1)
Cal curve 021_100_ng/mL
F28:MRM of 2 channels,ES+
237.259 > 166.115
5.017e+006
Citric acid/isocitric acid
5.08
189368.50
4970996
4.54
100
%
0
4.20
4.40
4.60
4.80
5.00
5.20
5.40
5.60
5.80
min

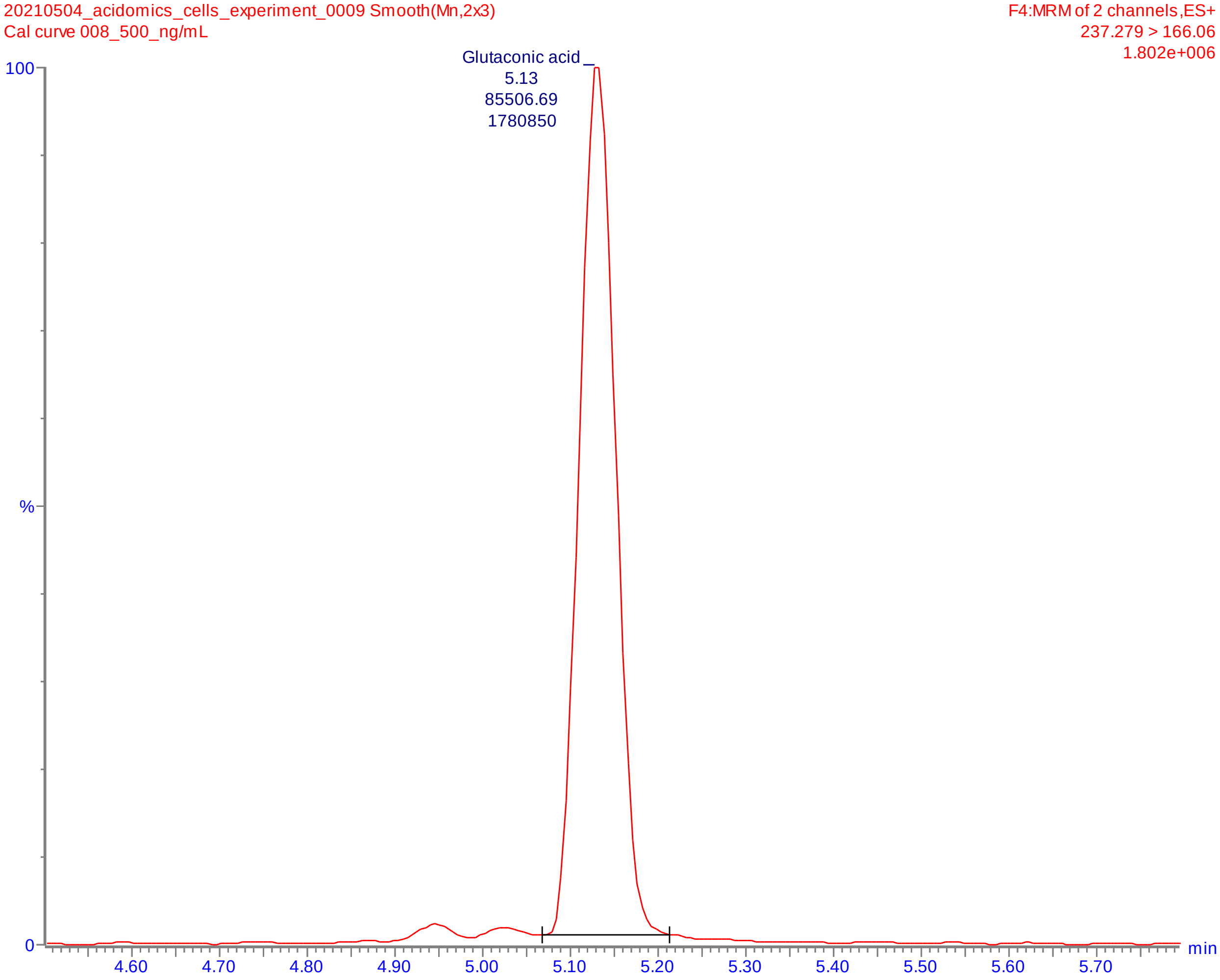

20210504_acidomics_cells_experiment_0009 Smooth(Mn,2x3)
Cal curve 008_500_ng/mL
F4:MRM of 2 channels,ES+
237.279 > 166.06
1.802e+006
Glutaconic acid
5.13
85506.69
1780850
100
%
0
4.60
4.70
4.80
4.90
5.00
5.10
5.20
5.30
5.40
5.50
5.60
5.70
min

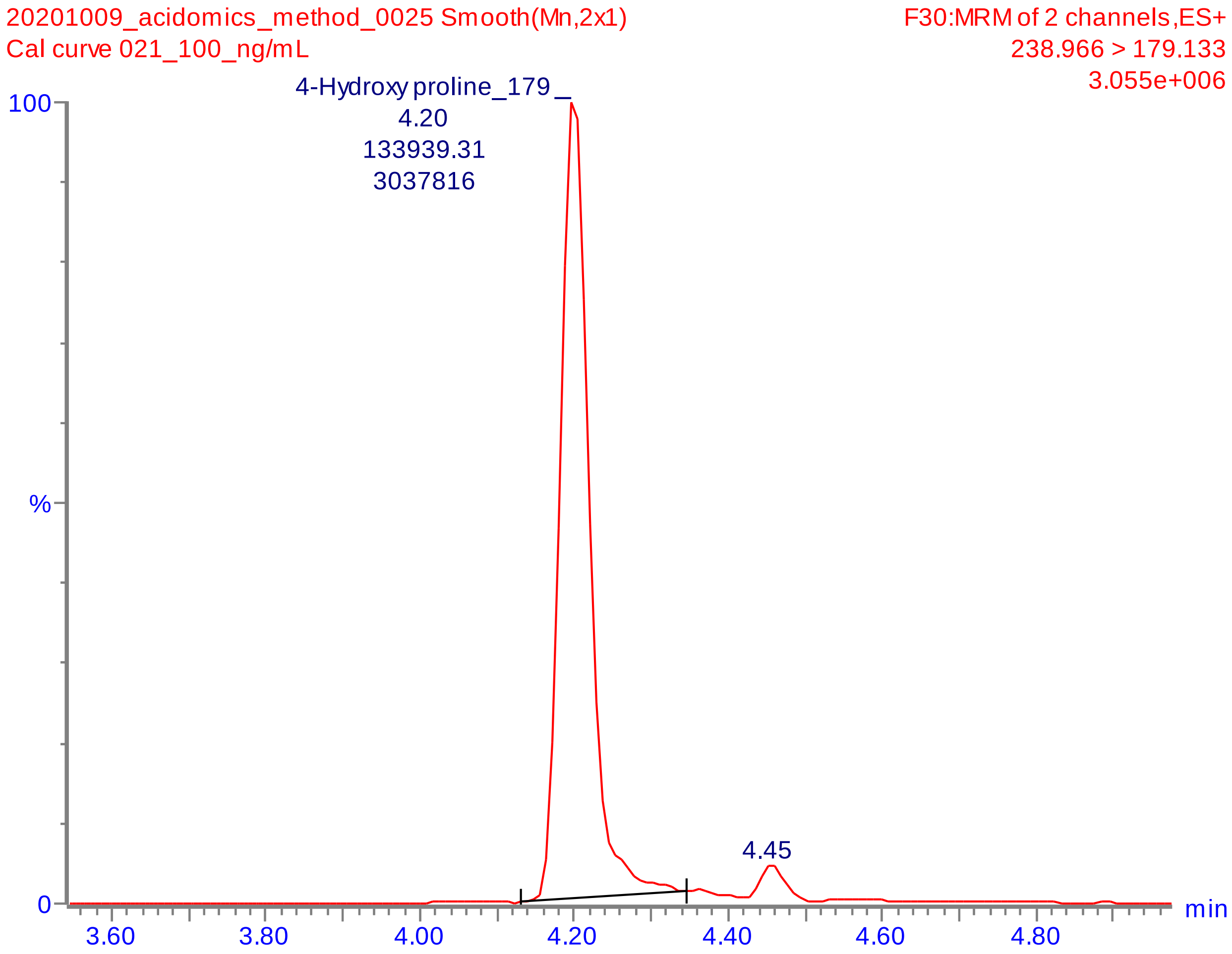

20201009_acidomics_method_0025 Smooth(Mn,2x1)
Cal curve 021_100_ng/mL
F30:MRM of 2 channels,ES+
238.966 > 179.133
3.055e+006
4-Hydroxy proline_179
4.20
133939.31
3037816
4.45
100
%
0
3.60
3.80
4.00
4.20
4.40
4.60
4.80
min

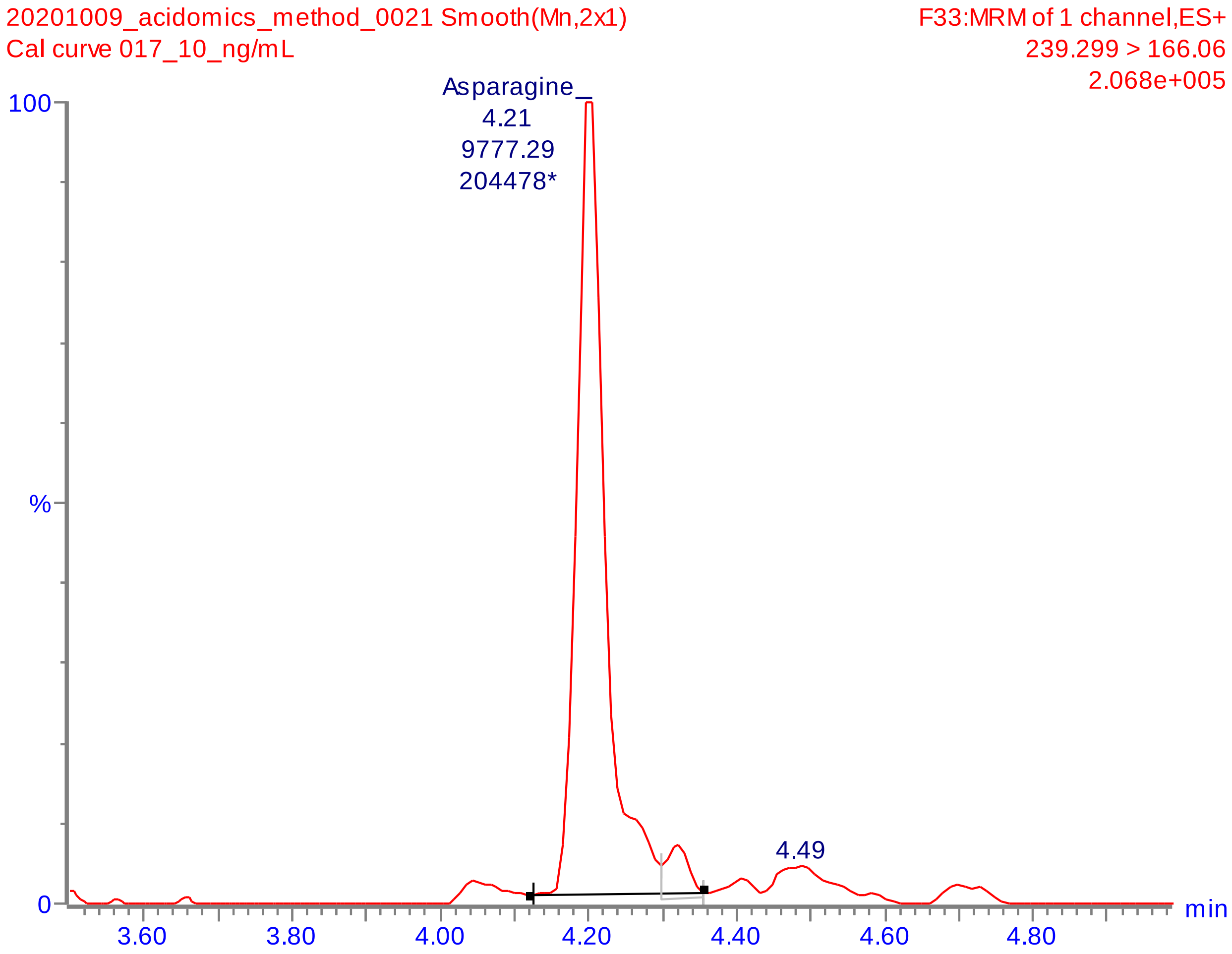

20201009_acidomics_method_0021 Smooth(Mn,2x1)
Cal curve 017_10_ng/mL
F33:MRM of 1 channel,ES+
239.299 > 166.06
2.068e+005
Asparagine_
4.21
9777.29
204478*
4.49
100
%
0
3.60
3.80
4.00
4.20
4.40
4.60
4.80
min

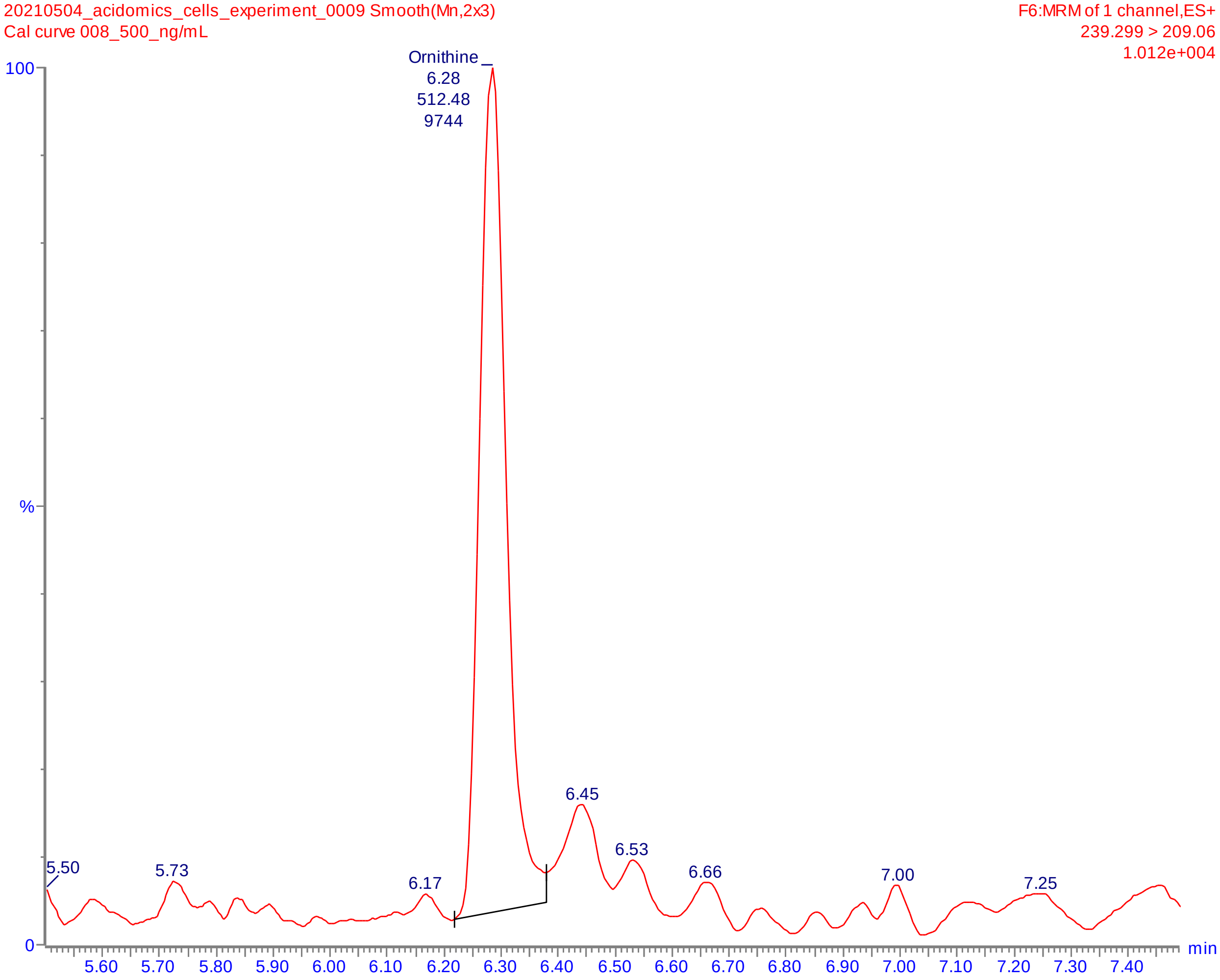

20210504_acidomics_cells_experiment_0009 Smooth(Mn,2x3)
Cal curve 008_500_ng/mL
F6:MRM of 1 channel,ES+
239.299 > 209.06
1.012e+004
Ornithine
6.28
512.48
9744
100
%
0
5.50
5.73
6.17
6.45
6.53
6.66
7.00
7.25
5.60
5.70
5.80
5.90
6.00
6.10
6.20
6.30
6.40
6.50
6.60
6.70
6.80
6.90
7.00
7.10
7.20
7.30
7.40
min

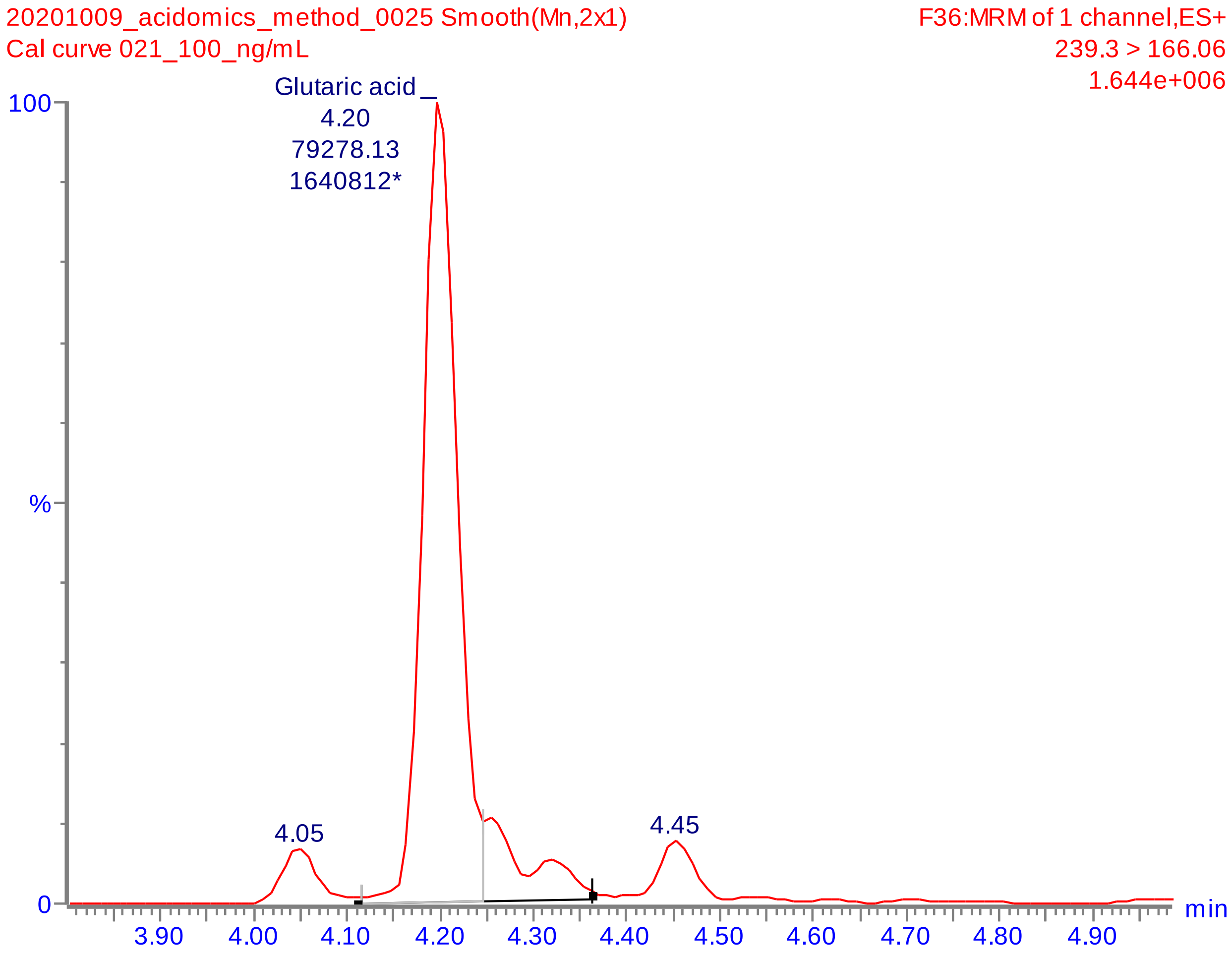
20201009_acidomics_method_0025 Smooth(Mn,2x1)
Cal curve 021_100_ng/mL
F36:MRM of 1 channel,ES+
239.3 > 166.06
1.644e+006
Glutaric acid
4.20
79278.13
1640812*
100
%
0
4.05
4.45
3.90
4.00
4.10
4.20
4.30
4.40
4.50
4.60
4.70
4.80
4.90
min

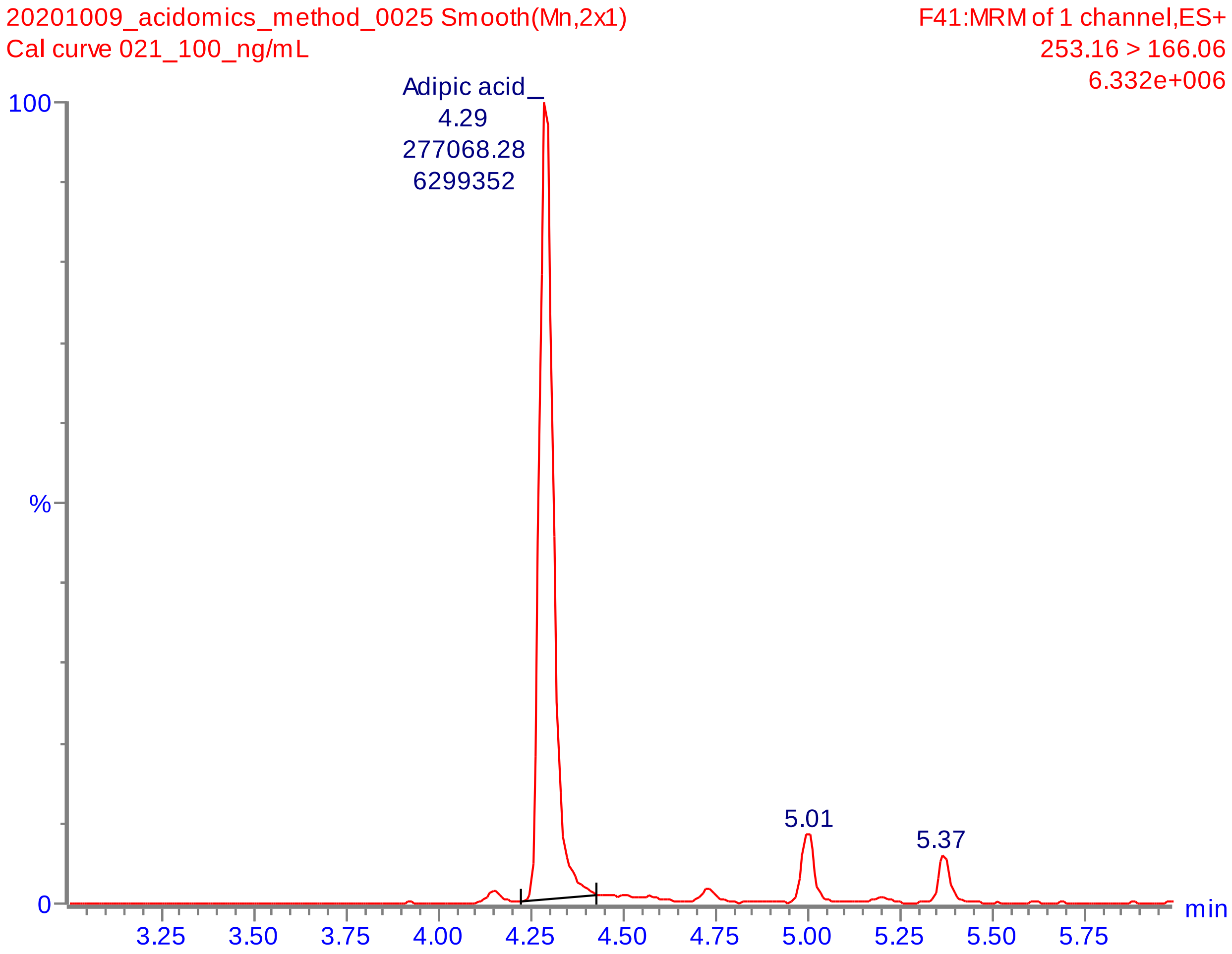

20201009_acidomics_method_0025 Smooth(Mn,2x1)
Cal curve 021_100_ng/mL
F41:MRM of 1 channel,ES+
253.16 > 166.06
6.332e+006
Adipic acid
4.29
277068.28
6299352
100
%
0
5.01
5.37
3.25
3.50
3.75
4.00
4.25
4.50
4.75
5.00
5.25
5.50
5.75
min

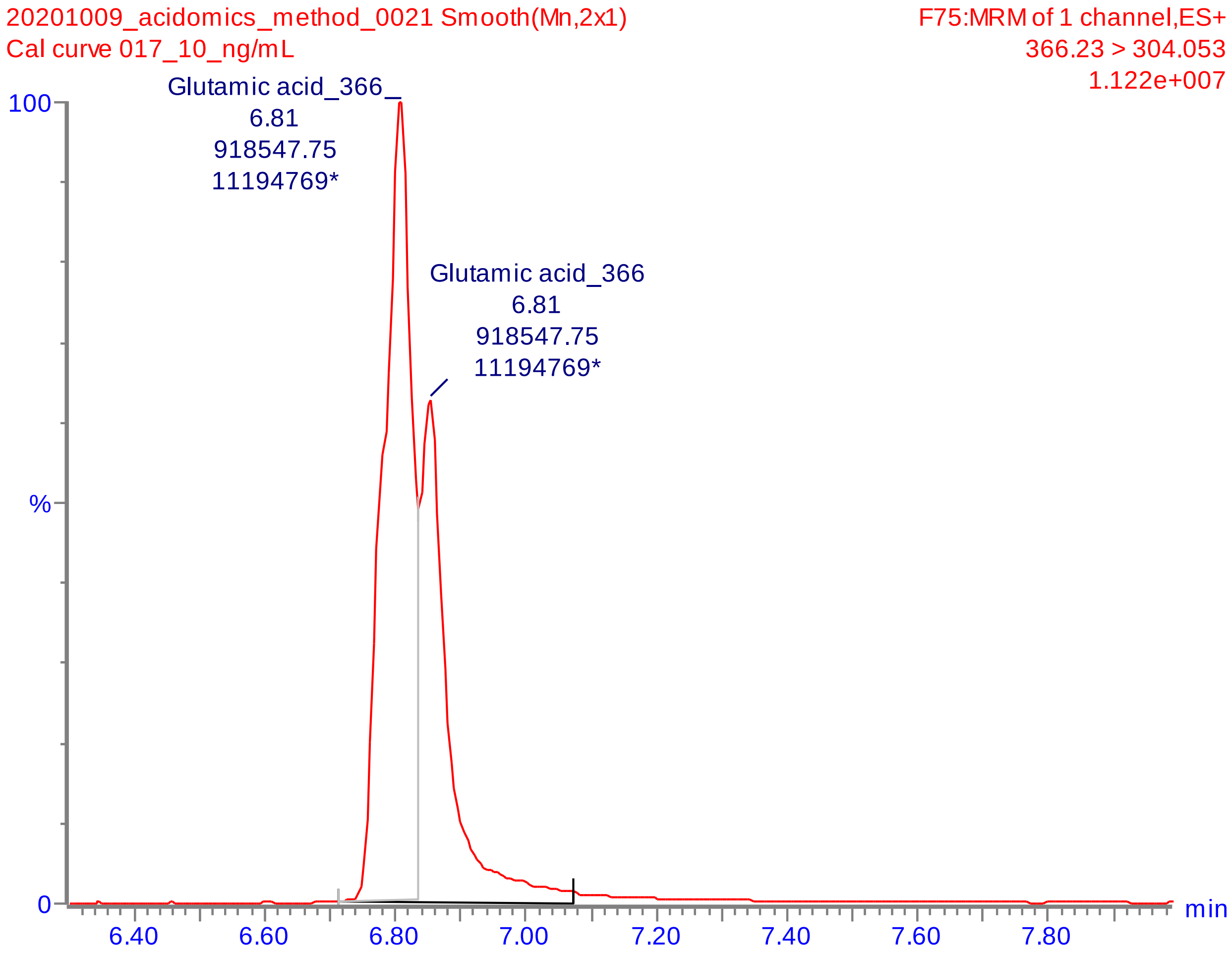

20201009_acidomics_method_0021 Smooth(Mn,2x1)
Cal curve 017_10_ng/mL
F75:MRM of 1 channel,ES+
366.23 > 304.053
1.122e+007
Glutamic acid_366
6.81
918547.75
11194769*
Glutamic acid_366
6.81
918547.75
11194769*
100
%
0
6.40
6.60
6.80
7.00
7.20
7.40
7.60
7.80
min

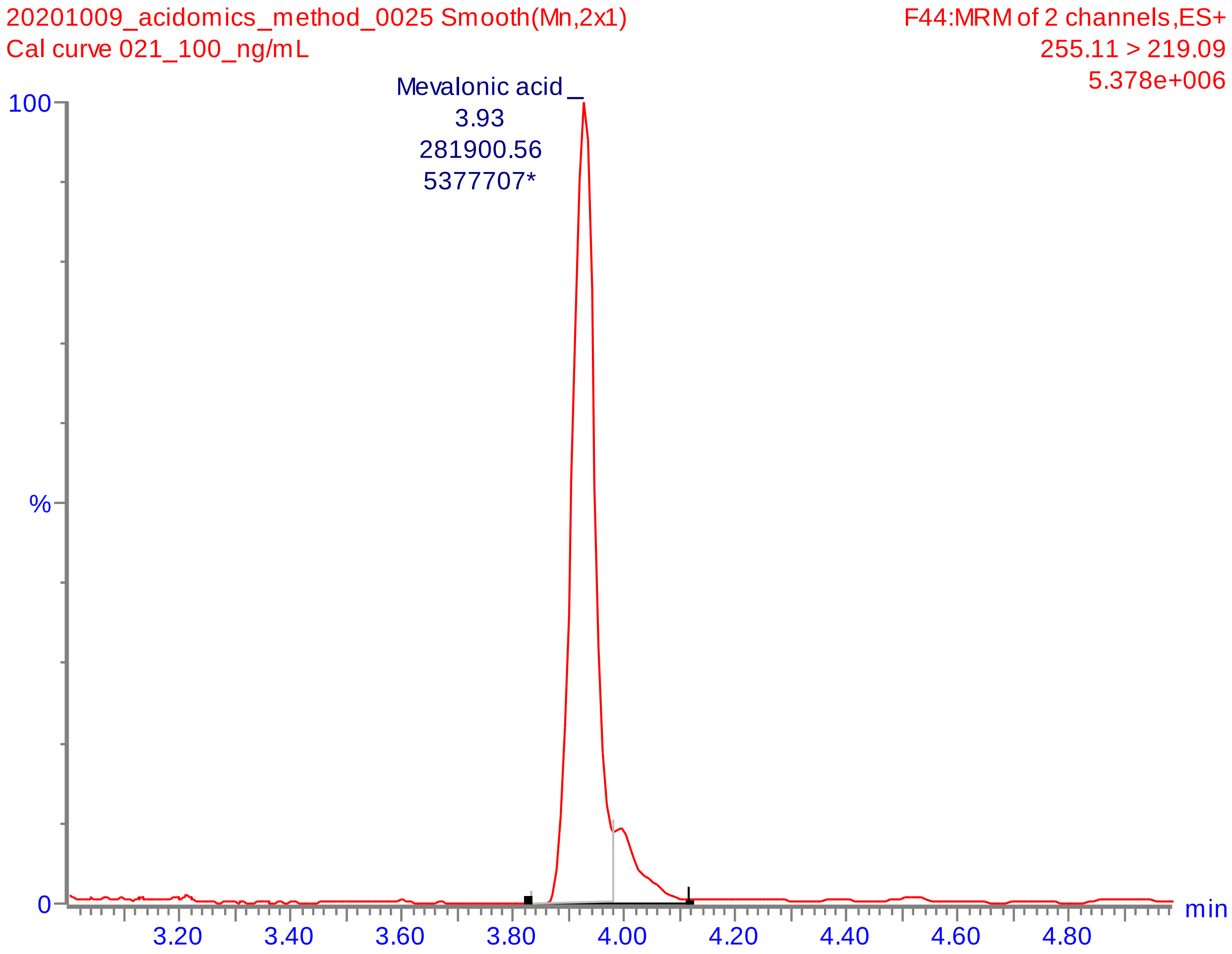

20201009_acidomics_method_0025 Smooth(Mn,2x1)
Cal curve 021_100_ng/mL
F44:MRM of 2 channels,ES+
255.11 > 219.09
5.378e+006
Mevalonic acid
3.93
281900.56
5377707*
100
%
0
3.20
3.40
3.60
3.80
4.00
4.20
4.40
4.60
4.80
min

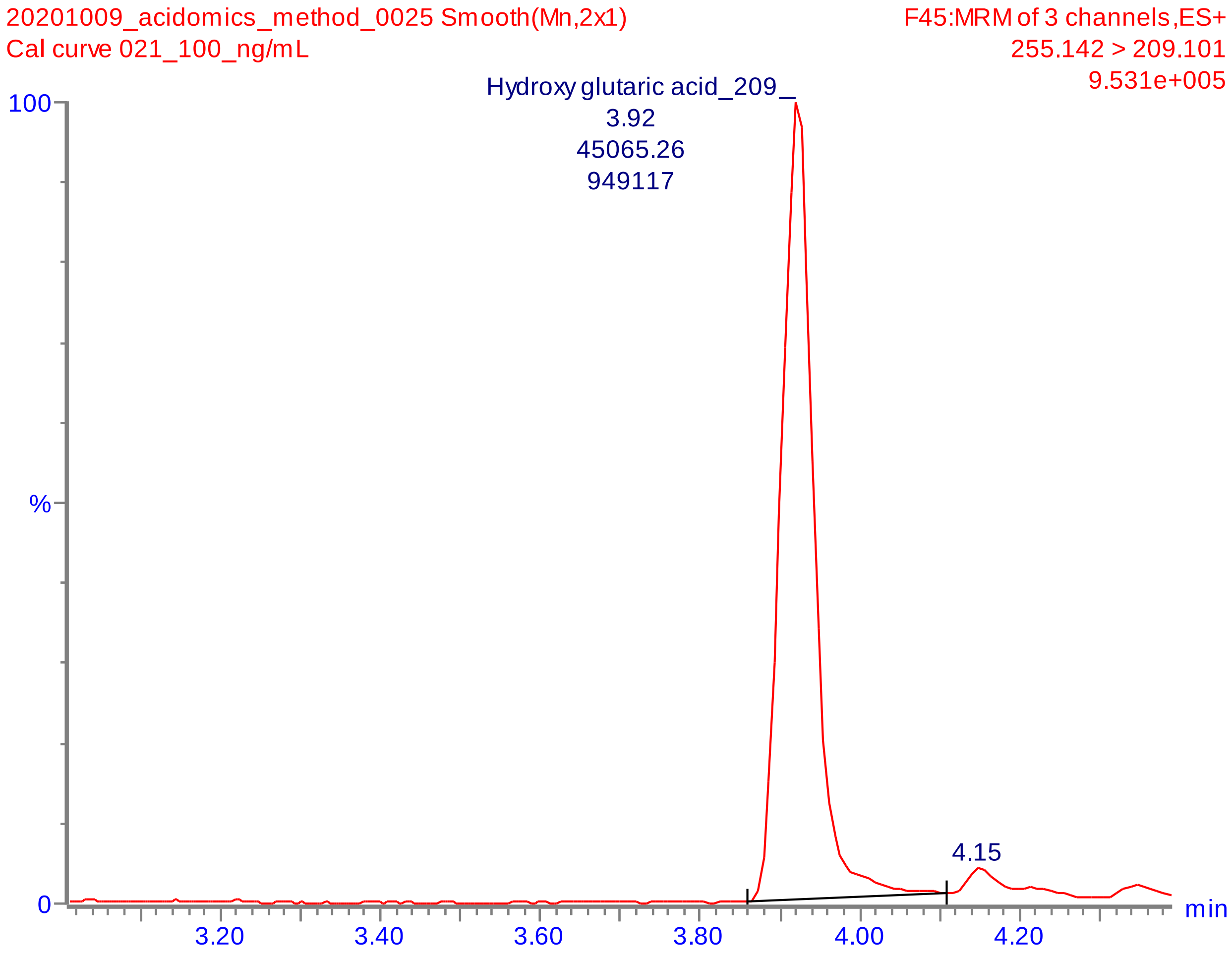

20201009_acidomics_method_0025 Smooth(Mn,2x1)
Cal curve 021_100_ng/mL
F45:MRM of 3 channels,ES+
255.142 > 209.101
9.531e+005
Hydroxy glutaric acid_209_
3.92
45065.26
949117
4.15
100
%
0
3.20
3.40
3.60
3.80
4.00
4.20
min

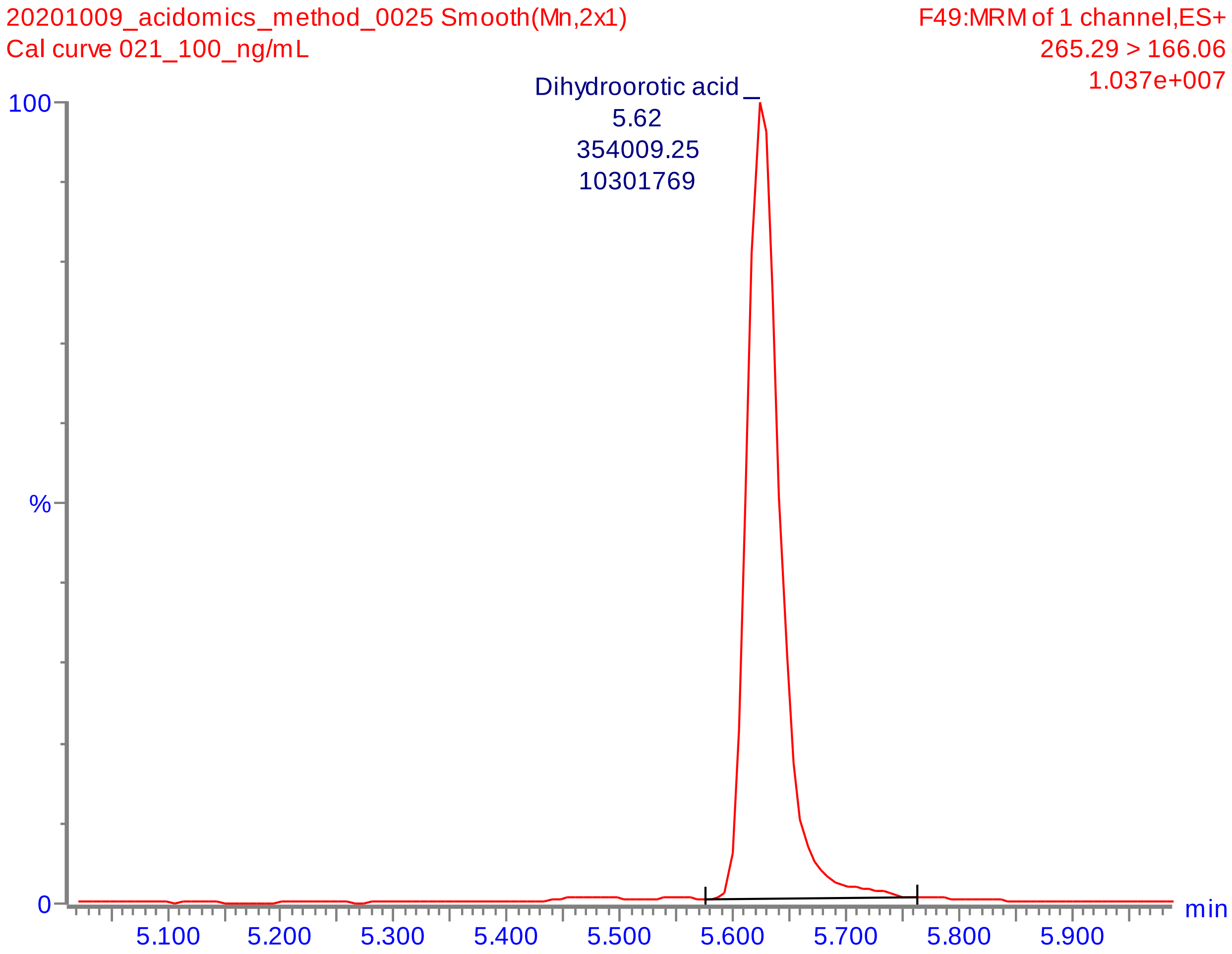

20201009_acidomics_method_0025 Smooth(Mn,2x1)
Cal curve 021_100_ng/mL
F49:MRM of 1 channel,ES+
265.29 > 166.06
1.037e+007
Dihydroorotic acid
5.62
354009.25
10301769
100
%
0
5.100
5.200
5.300
5.400
5.500
5.600
5.700
5.800
5.900
min

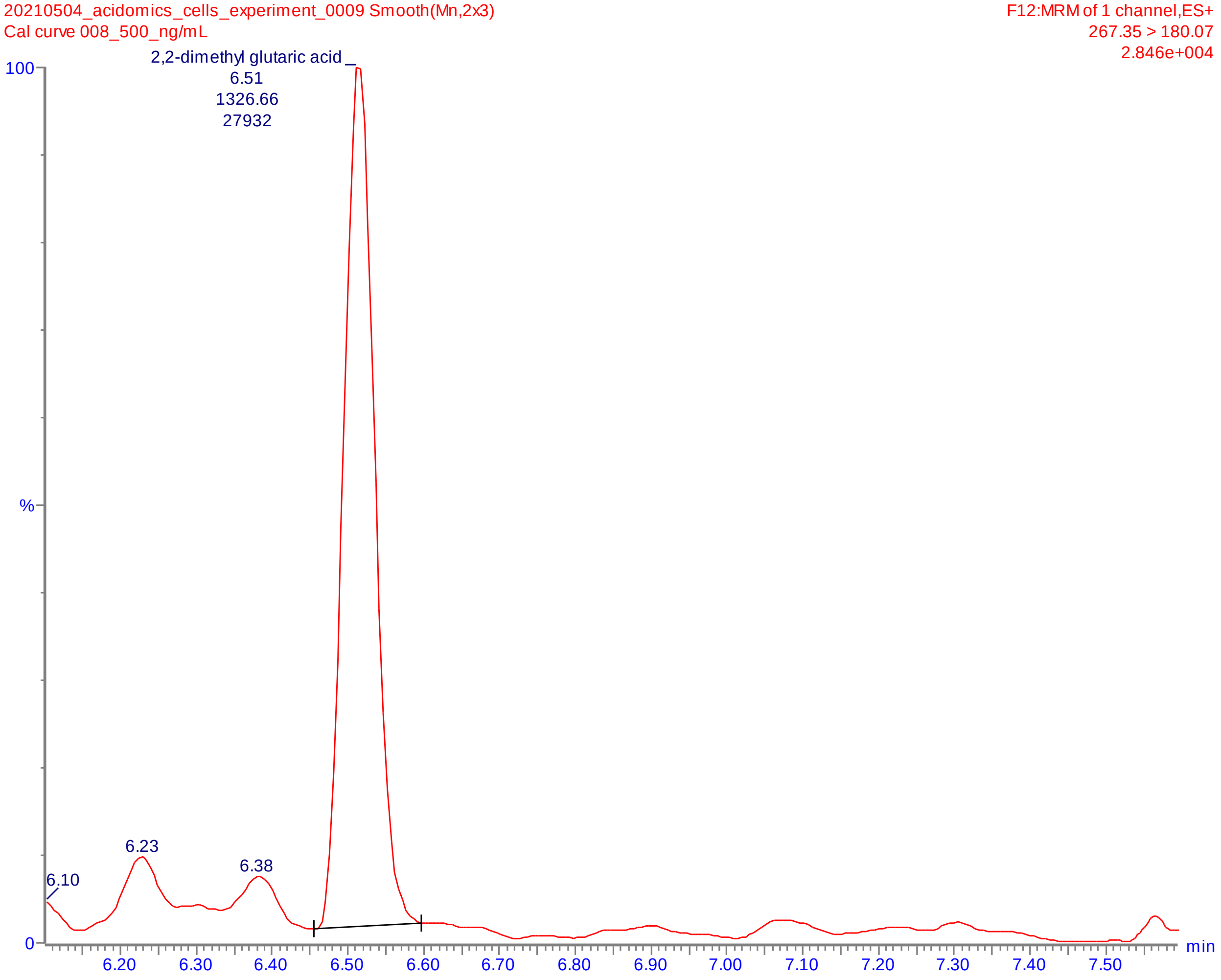
20210504_acidomics_cells_experiment_0009 Smooth(Mn,2x3)
Cal curve 008_500_ng/mL
F12:MRM of 1 channel,ES+
267.35 > 180.07
2.846e+004
2,2-dimethyl glutaric acid
6.51
1326.66
27932
100
%
0
6.10
6.23
6.38
6.20
6.30
6.40
6.50
6.60
6.70
6.80
6.90
7.00
7.10
7.20
7.30
7.40
7.50
min

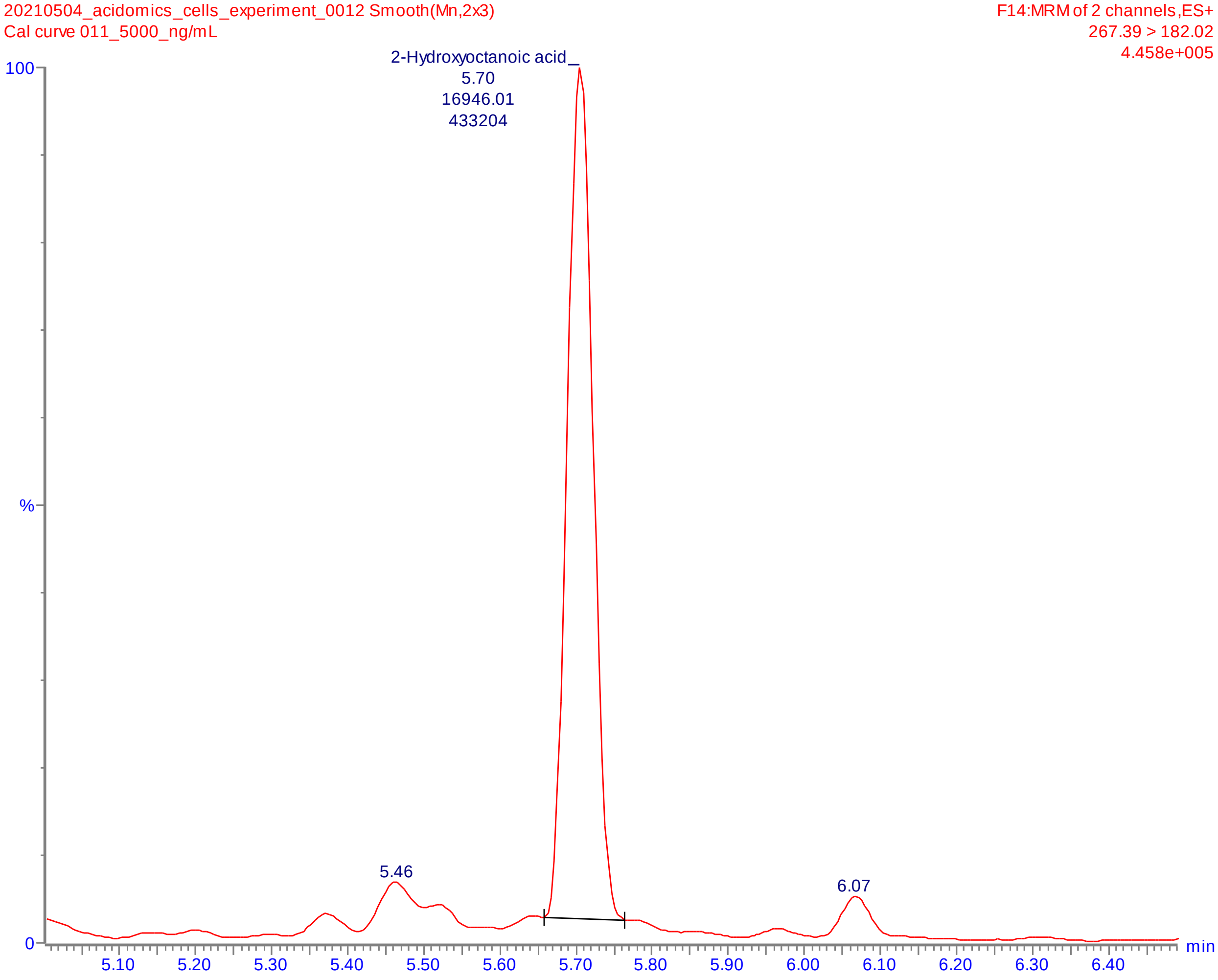

20210504_acidomics_cells_experiment_0012 Smooth(Mn,2x3)
Cal curve 011_5000_ng/mL
F14:MRM of 2 channels,ES+
267.39 > 182.02
4.458e+005
2-Hydroxyoctanoic acid_
5.70
16946.01
433204
100
%
0
5.46
6.07
5.10
5.20
5.30
5.40
5.50
5.60
5.70
5.80
5.90
6.00
6.10
6.20
6.30
6.40
min

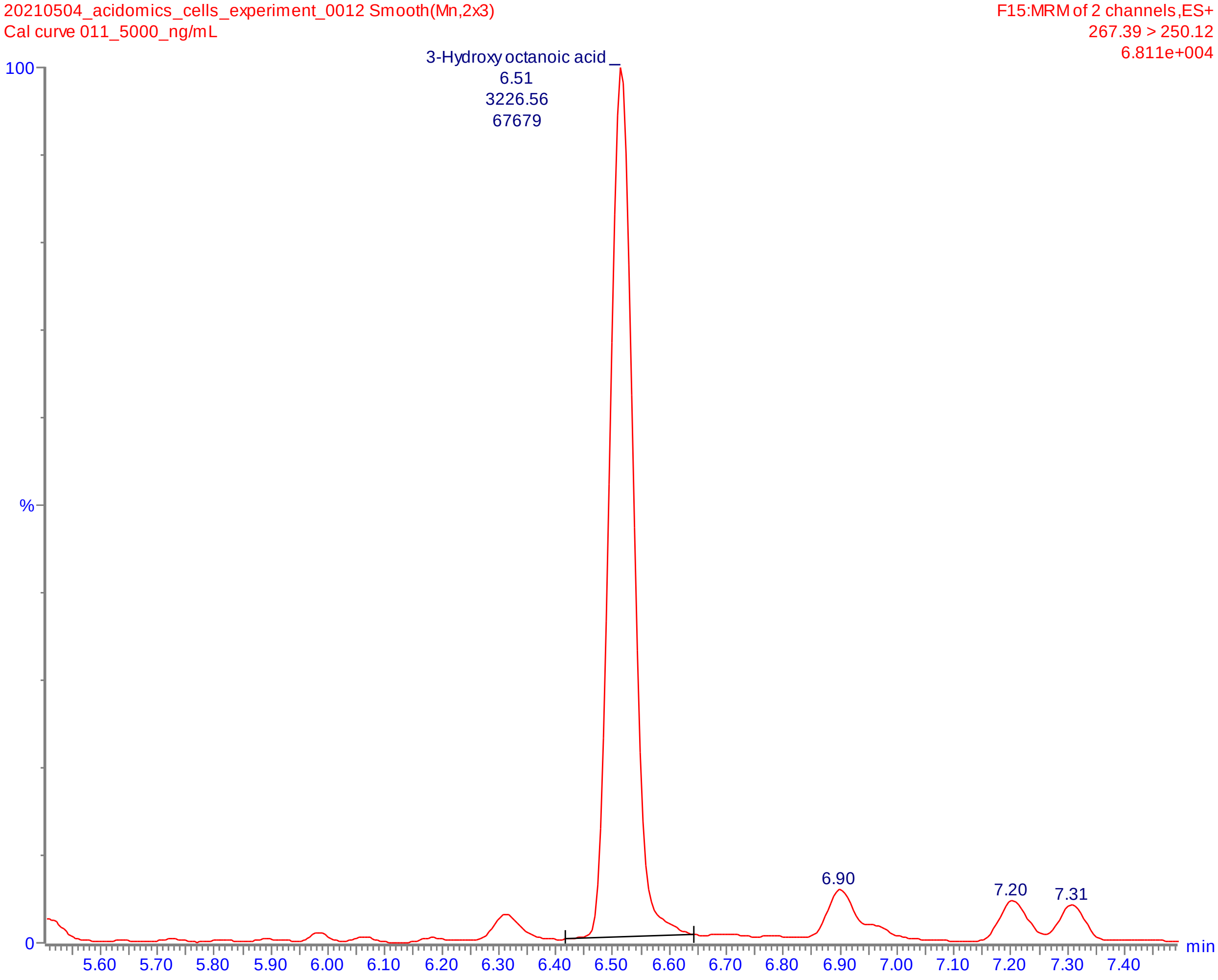

20210504_acidomics_cells_experiment_0012 Smooth(Mn,2x3)
Cal curve 011_5000_ng/mL
F15:MRM of 2 channels,ES+
267.39 > 250.12
6.811e+004
3-Hydroxy octanoic acid
6.51
3226.56
67679
6.90
7.20
7.31
100
%
0
5.60
5.70
5.80
5.90
6.00
6.10
6.20
6.30
6.40
6.50
6.60
6.70
6.80
6.90
7.00
7.10
7.20
7.30
7.40
min

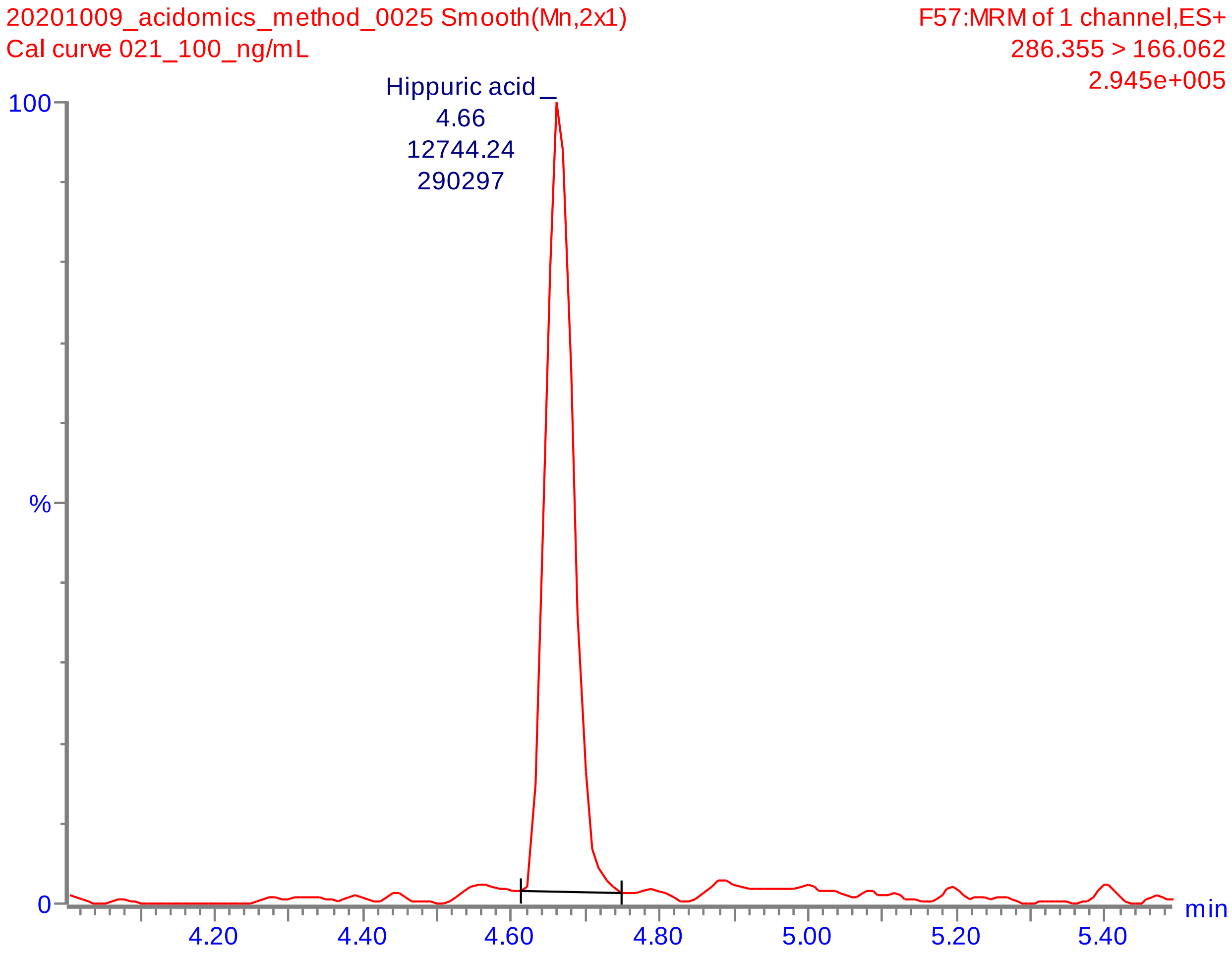

20201009_acidomics_method_0025 Smooth(Mn,2x1)
Cal curve 021_100_ng/mL
F57:MRM of 1 channel,ES+
286.355 > 166.062
2.945e+005
Hippuric acid
4.66
12744.24
290297
100
%
0
4.20
4.40
4.60
4.80
5.00
5.20
5.40
min

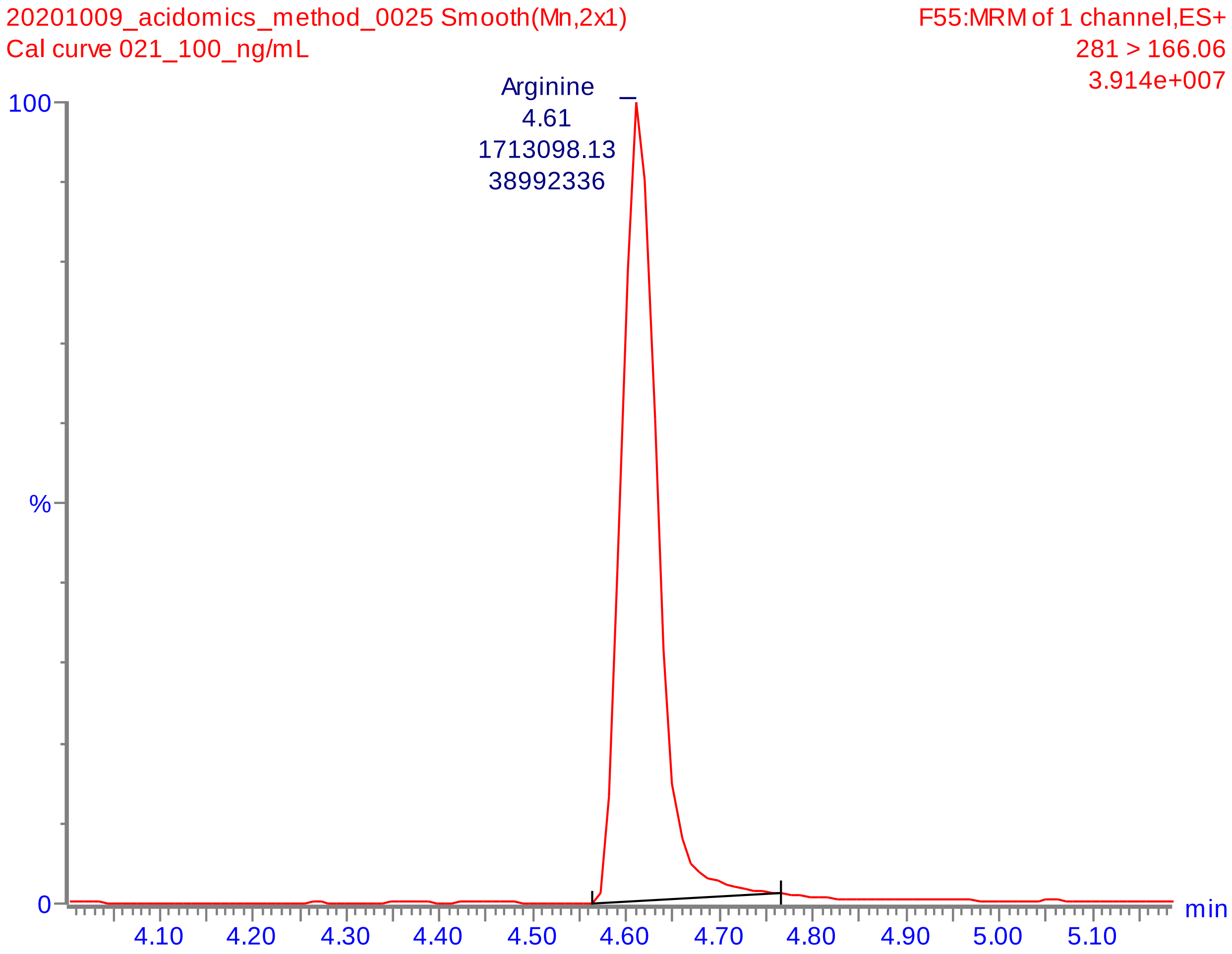

20201009_acidomics_method_0025 Smooth(Mn,2x1)
Cal curve 021_100_ng/mL
F55:MRM of 1 channel,ES+
281 > 166.06
3.914e+007
Arginine
4.61
1713098.13
38992336
100
%
0
4.10
4.20
4.30
4.40
4.50
4.60
4.70
4.80
4.90
5.00
5.10
min

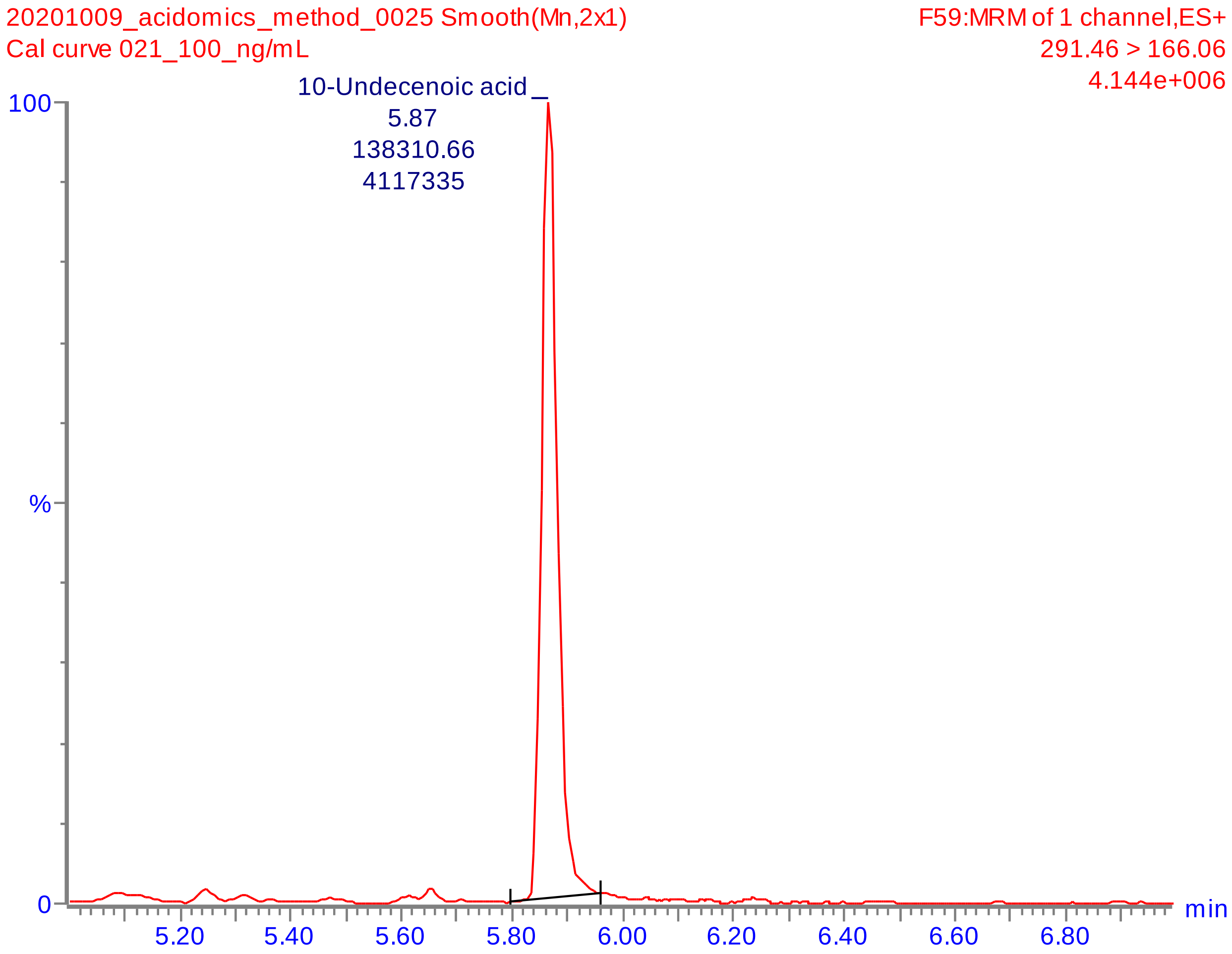
20201009_acidomics_method_0025 Smooth(Mn,2x1)
Cal curve 021_100_ng/mL
F59:MRM of 1 channel,ES+
291.46 > 166.06
4.144e+006
10-Undecenoic acid
5.87
138310.66
4117335
100
%
0
5.20
5.40
5.60
5.80
6.00
6.20
6.40
6.60
6.80
min

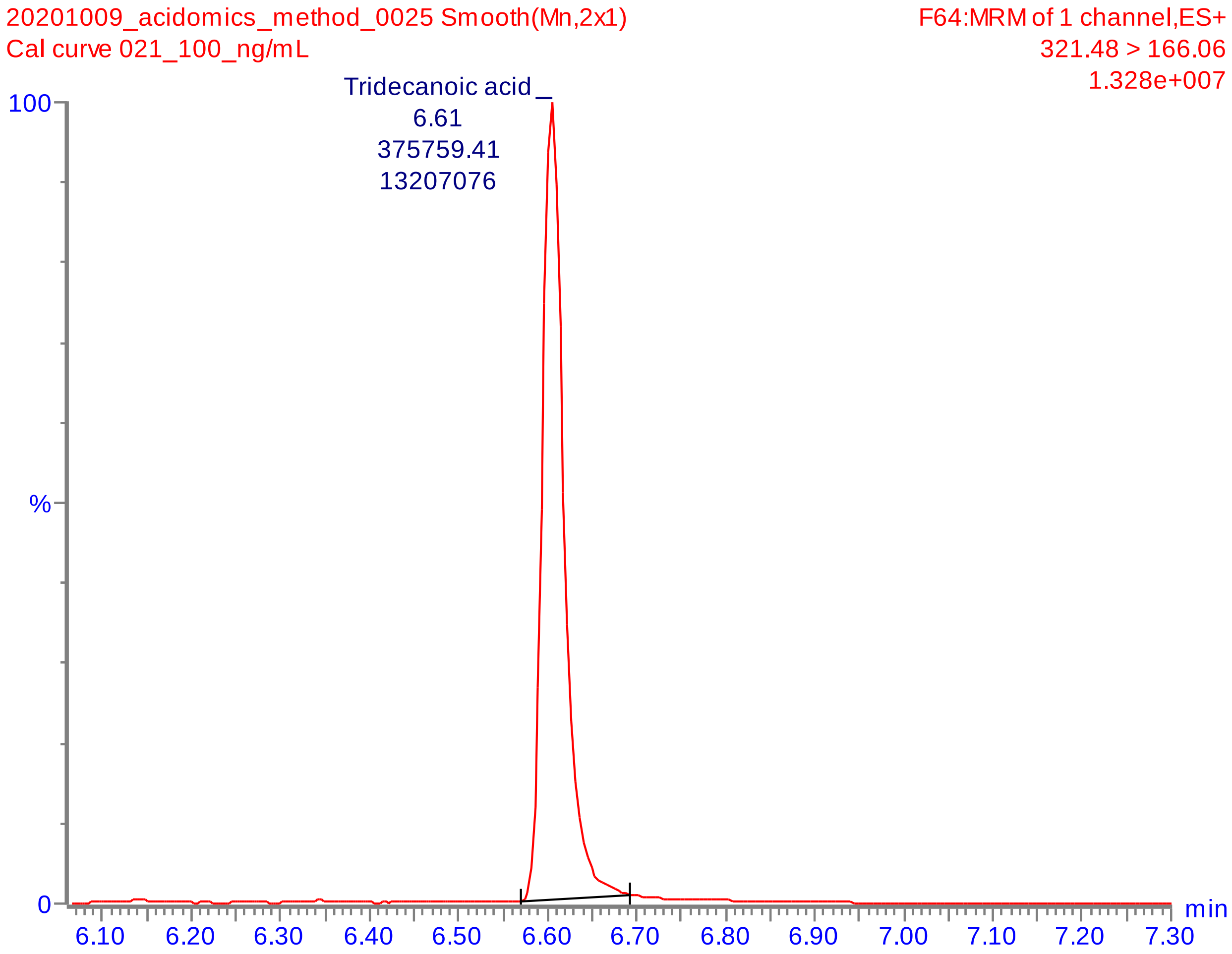

20201009_acidomics_method_0025 Smooth(Mn,2x1)
Cal curve 021_100_ng/mL
F64:MRM of 1 channel,ES+
321.48 > 166.06
1.328e+007
Tridecanoic acid
6.61
375759.41
13207076
100
%
0
6.10
6.20
6.30
6.40
6.50
6.60
6.70
6.80
6.90
7.00
7.10
7.20
7.30
min

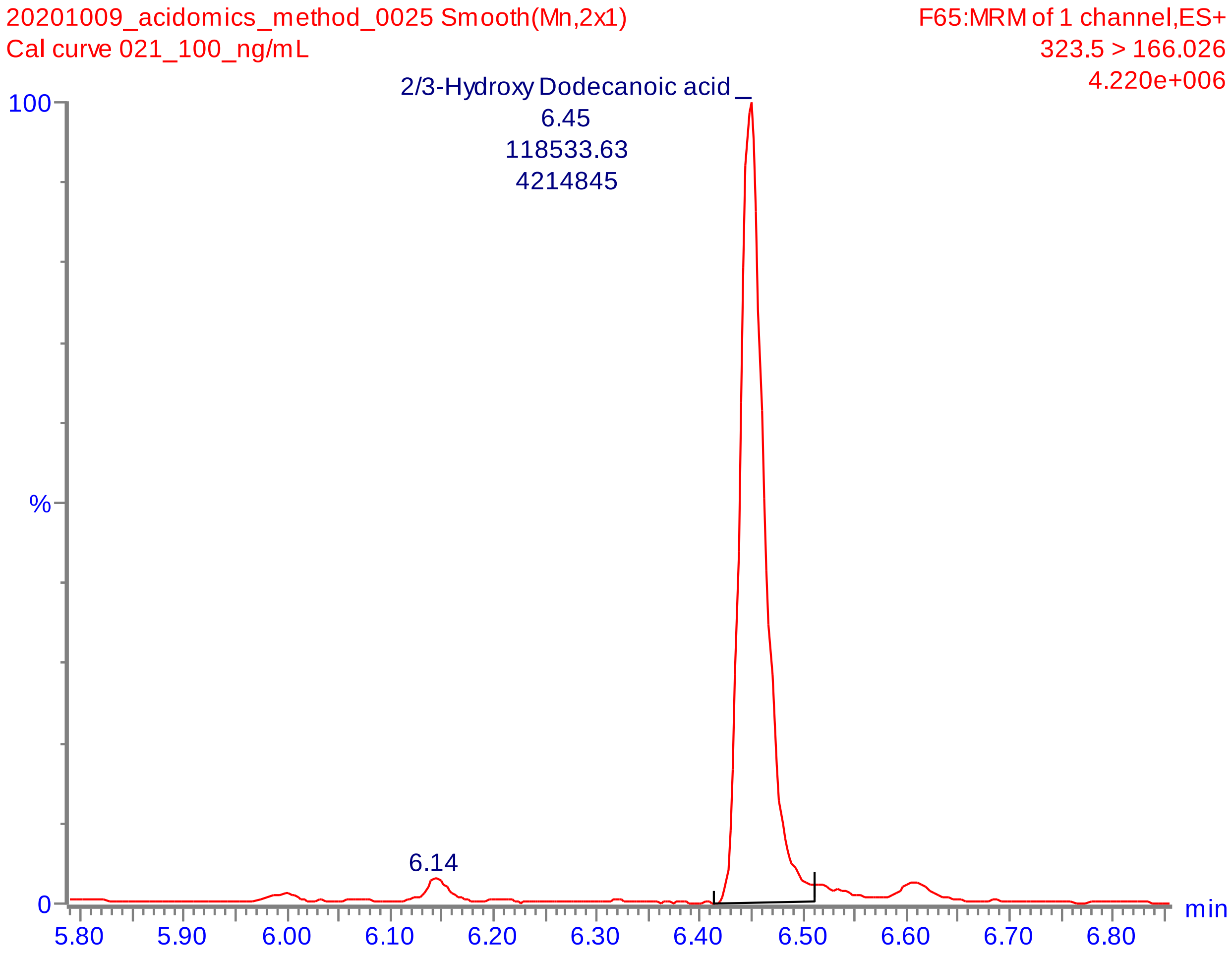

20201009_acidomics_method_0025 Smooth(Mn,2x1)
Cal curve 021_100_ng/mL
F65:MRM of 1 channel,ES+
323.5 > 166.026
4.220e+006
2/3-Hydroxy Dodecanoic acid
6.45
118533.63
4214845
6.14
100
%
0
5.80
5.90
6.00
6.10
6.20
6.30
6.40
6.50
6.60
6.70
6.80
min

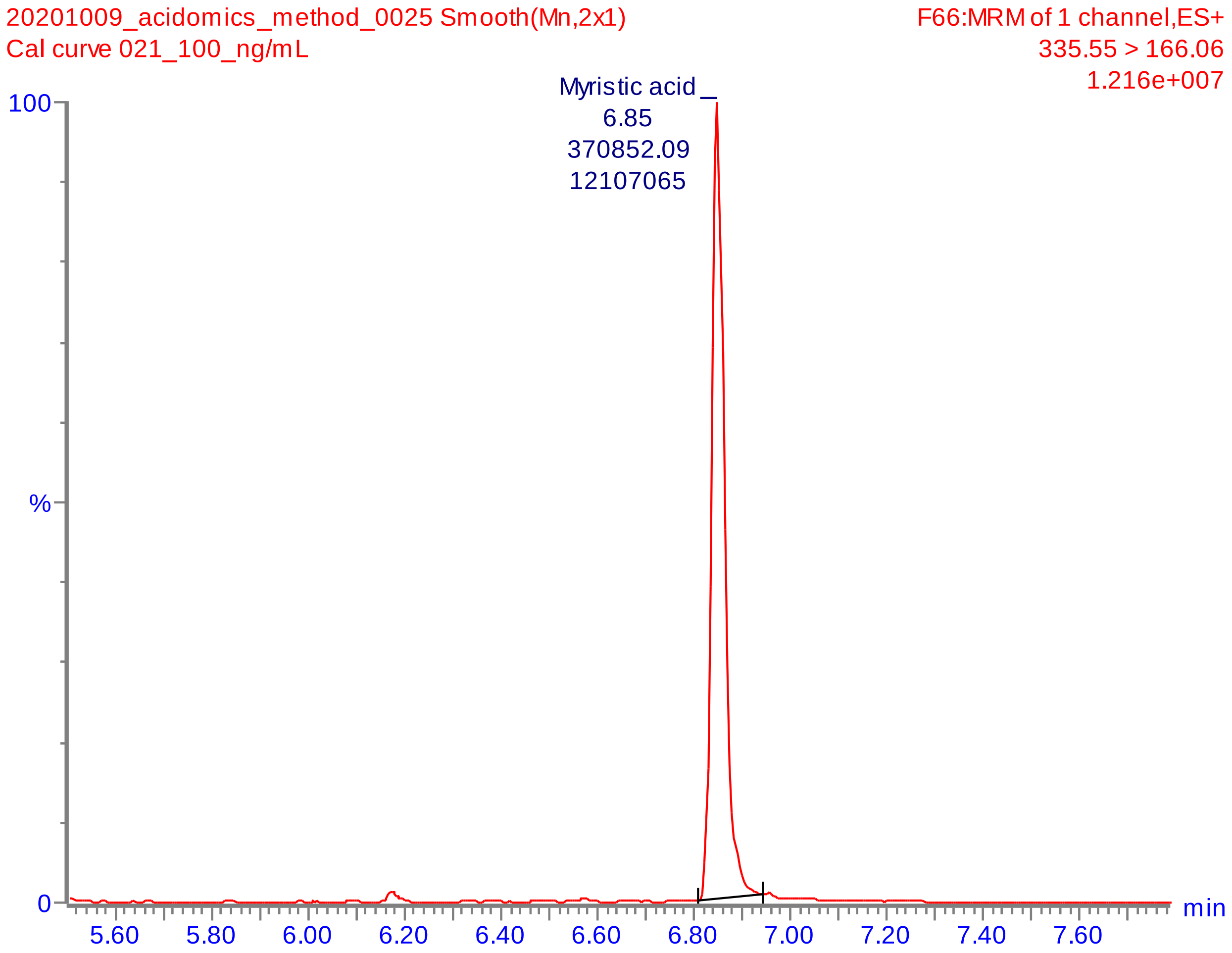

20201009_acidomics_method_0025 Smooth(Mn,2x1)
Cal curve 021_100_ng/mL
F66:MRM of 1 channel,ES+
335.55 > 166.06
1.216e+007
Myristic acid
6.85
370852.09
12107065
100
%
0
5.60
5.80
6.00
6.20
6.40
6.60
6.80
7.00
7.20
7.40
7.60
min

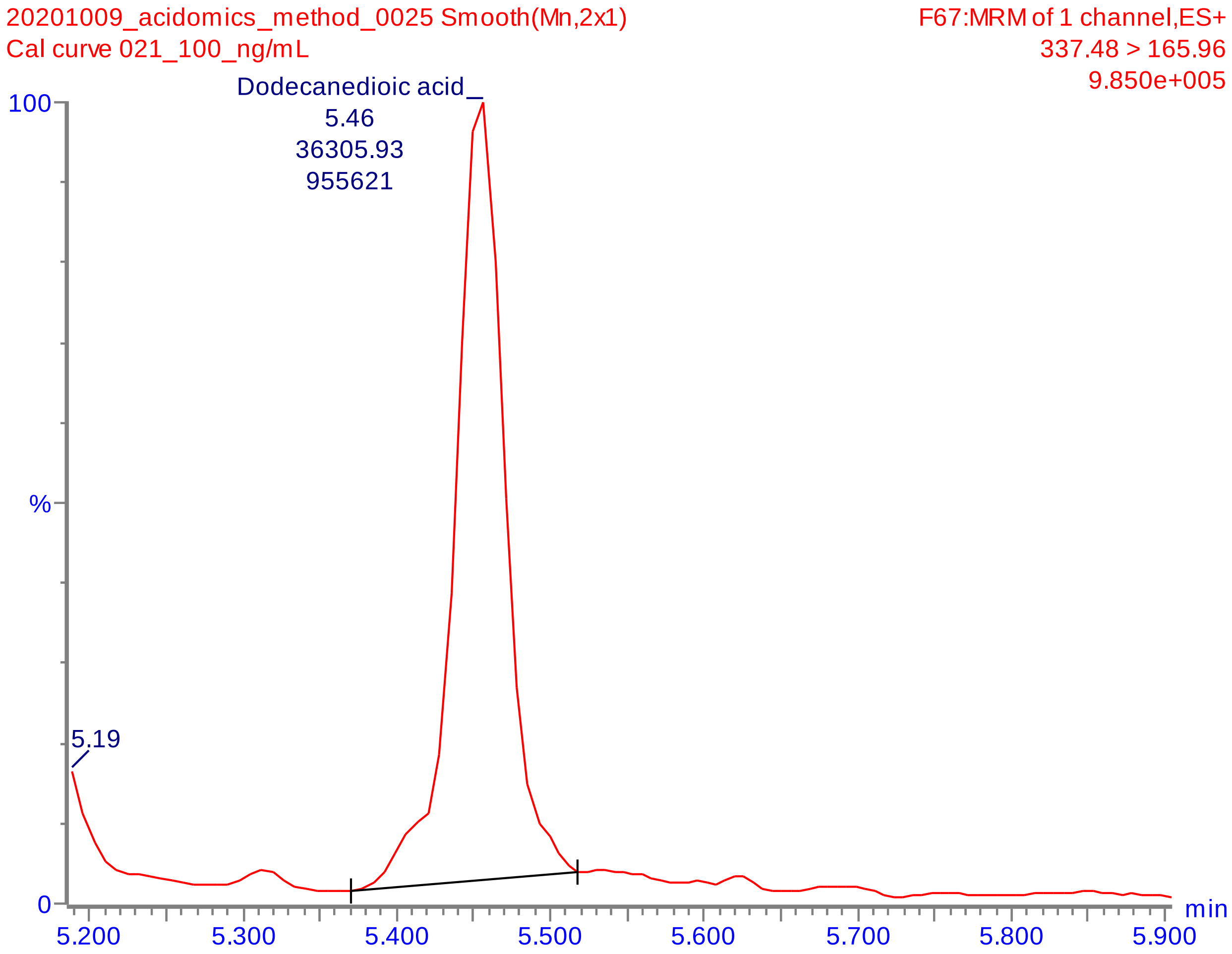
20201009_acidomics_method_0025 Smooth(Mn,2x1)
Cal curve 021_100_ng/mL
F67:MRM of 1 channel,ES+
337.48 > 165.96
9.850e+005
Dodecanedioic acid
5.46
36305.93
955621
5.19
100
%
0
5.200
5.300
5.400
5.500
5.600
5.700
5.800
5.900
min

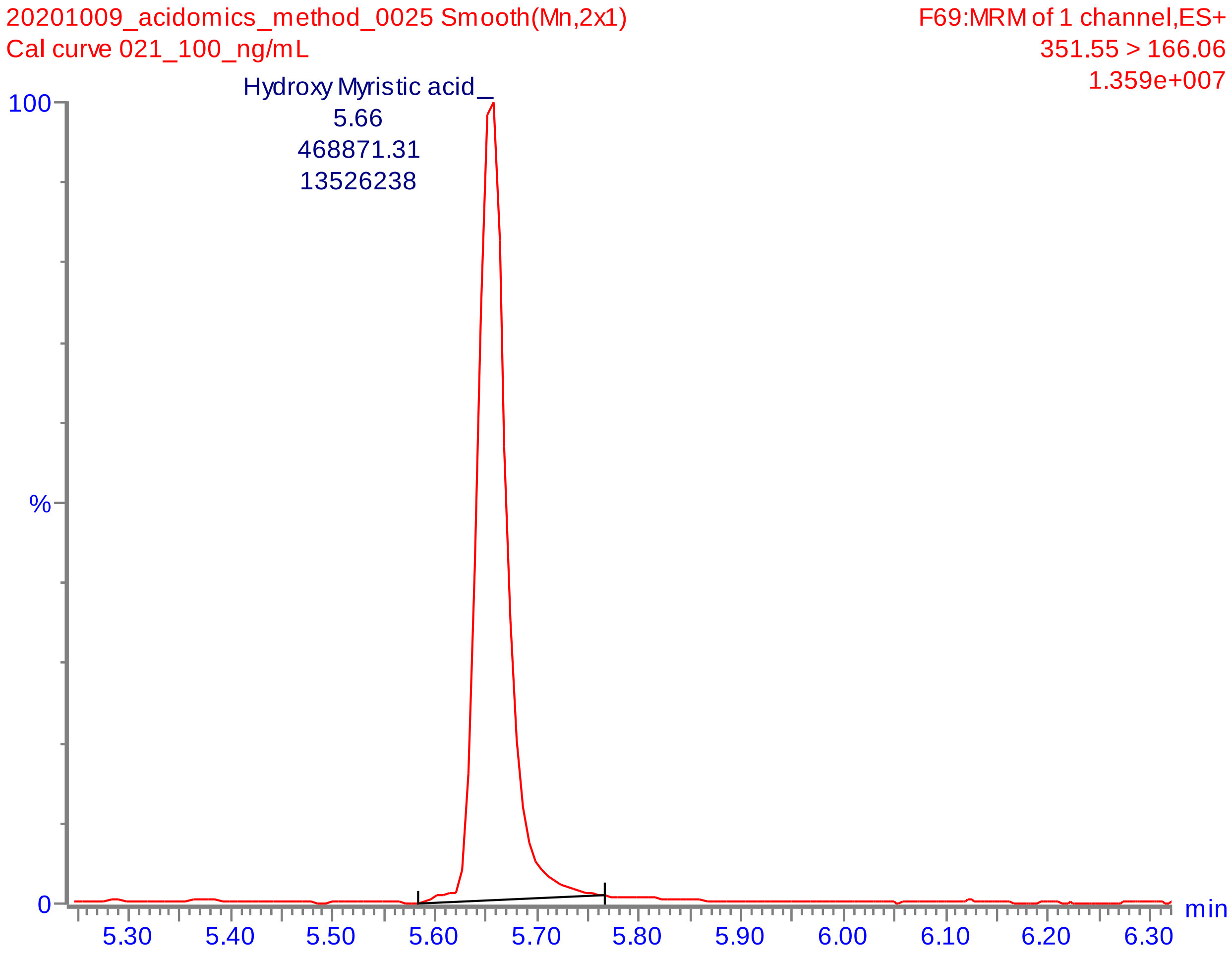

20201009_acidomics_method_0025 Smooth(Mn,2x1)
Cal curve 021_100_ng/mL
F69:MRM of 1 channel,ES+
351.55 > 166.06
1.359e+007
Hydroxy Myristic acid
5.66
468871.31
13526238
100
%
0
5.30
5.40
5.50
5.60
5.70
5.80
5.90
6.00
6.10
6.20
6.30
min

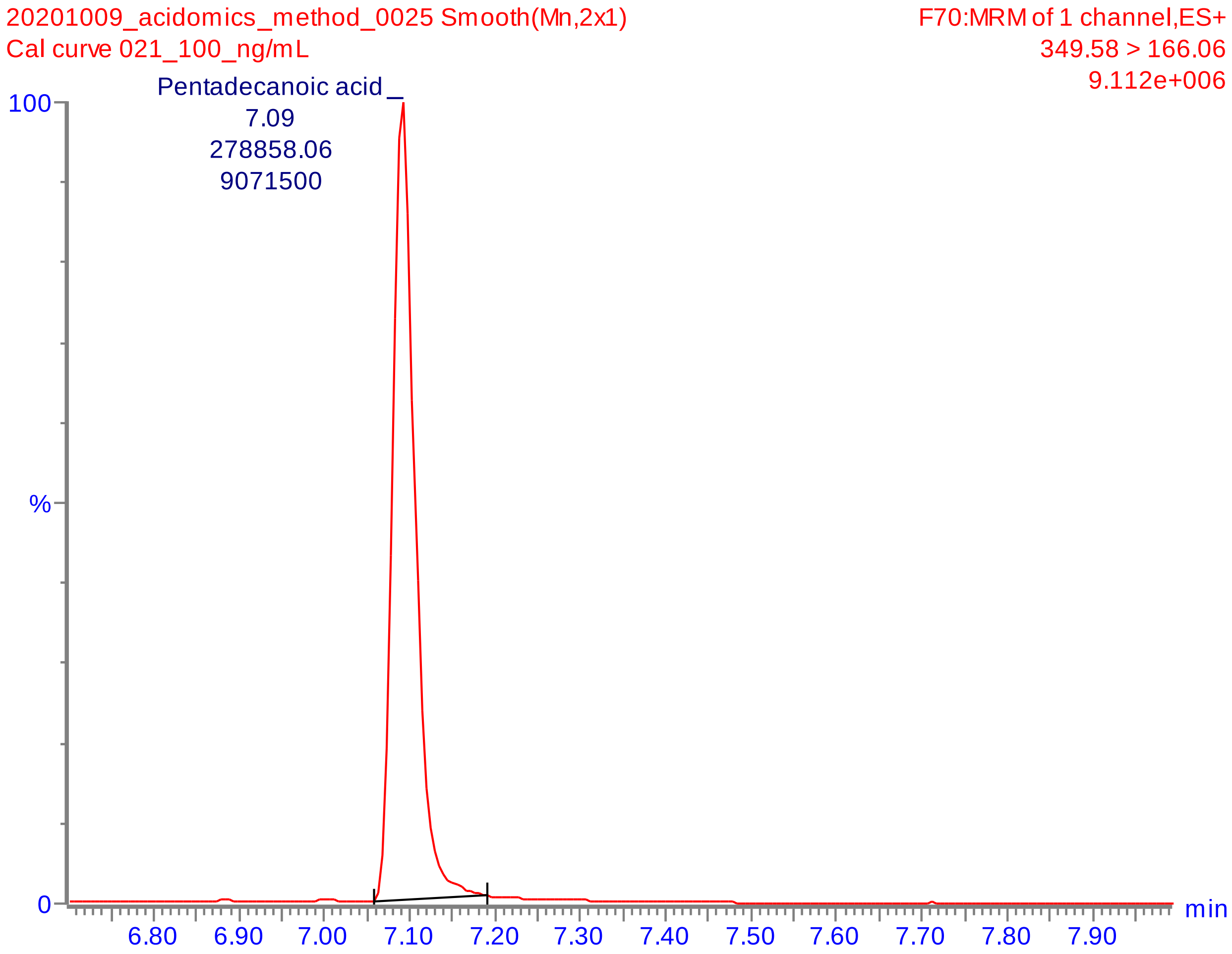

20201009_acidomics_method_0025 Smooth(Mn,2x1)
Cal curve 021_100_ng/mL
F70:MRM of 1 channel,ES+
349.58 > 166.06
9.112e+006
Pentadecanoic acid
7.09
278858.06
9071500
100
%
0
6.80
6.90
7.00
7.10
7.20
7.30
7.40
7.50
7.60
7.70
7.80
7.90
min

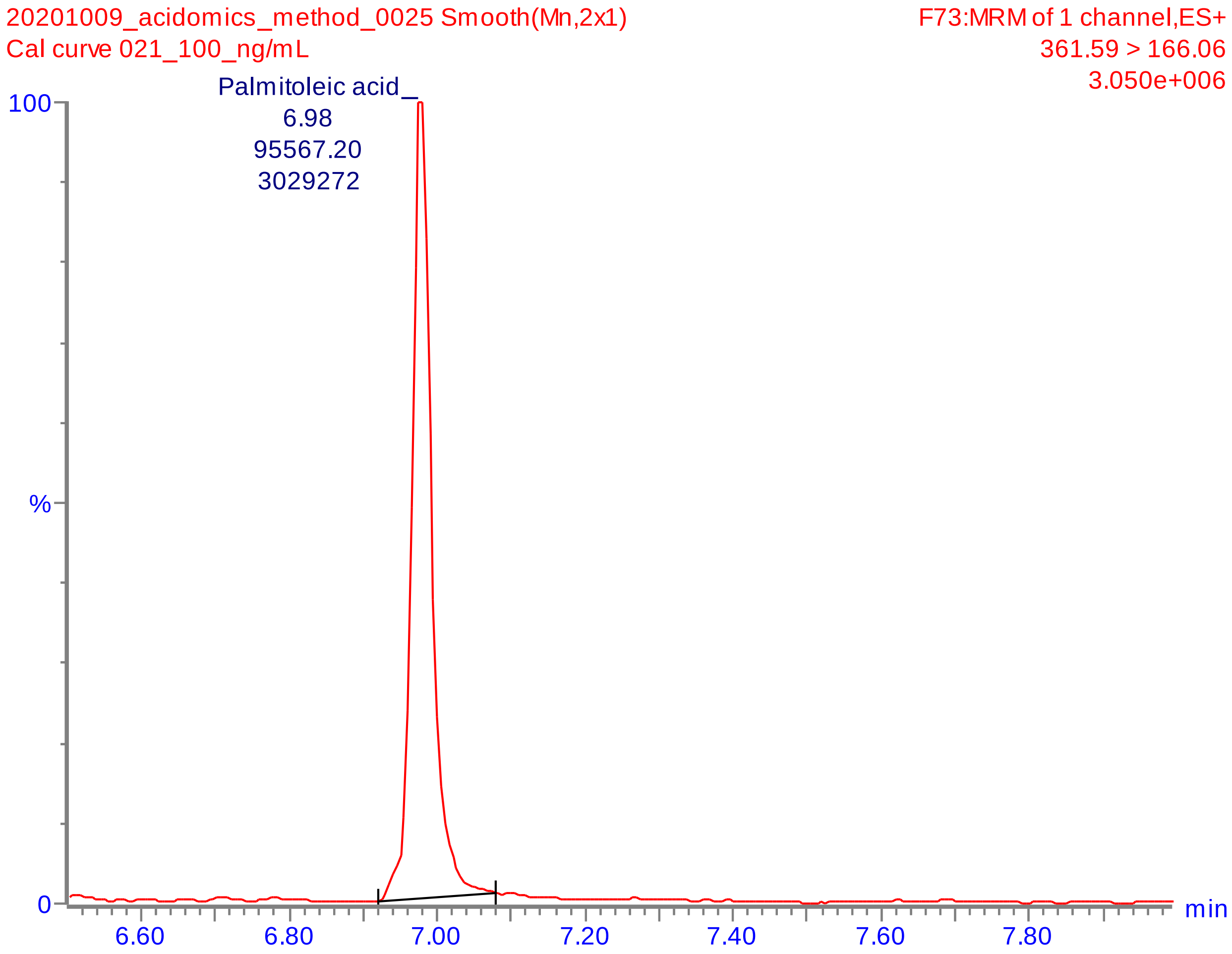
20201009_acidomics_method_0025 Smooth(Mn,2x1)
Cal curve 021_100_ng/mL
F73:MRM of 1 channel,ES+
361.59 > 166.06
3.050e+006
Palmitoleic acid_
6.98
95567.20
3029272
100
%
0
6.60
6.80
7.00
7.20
7.40
7.60
7.80
min

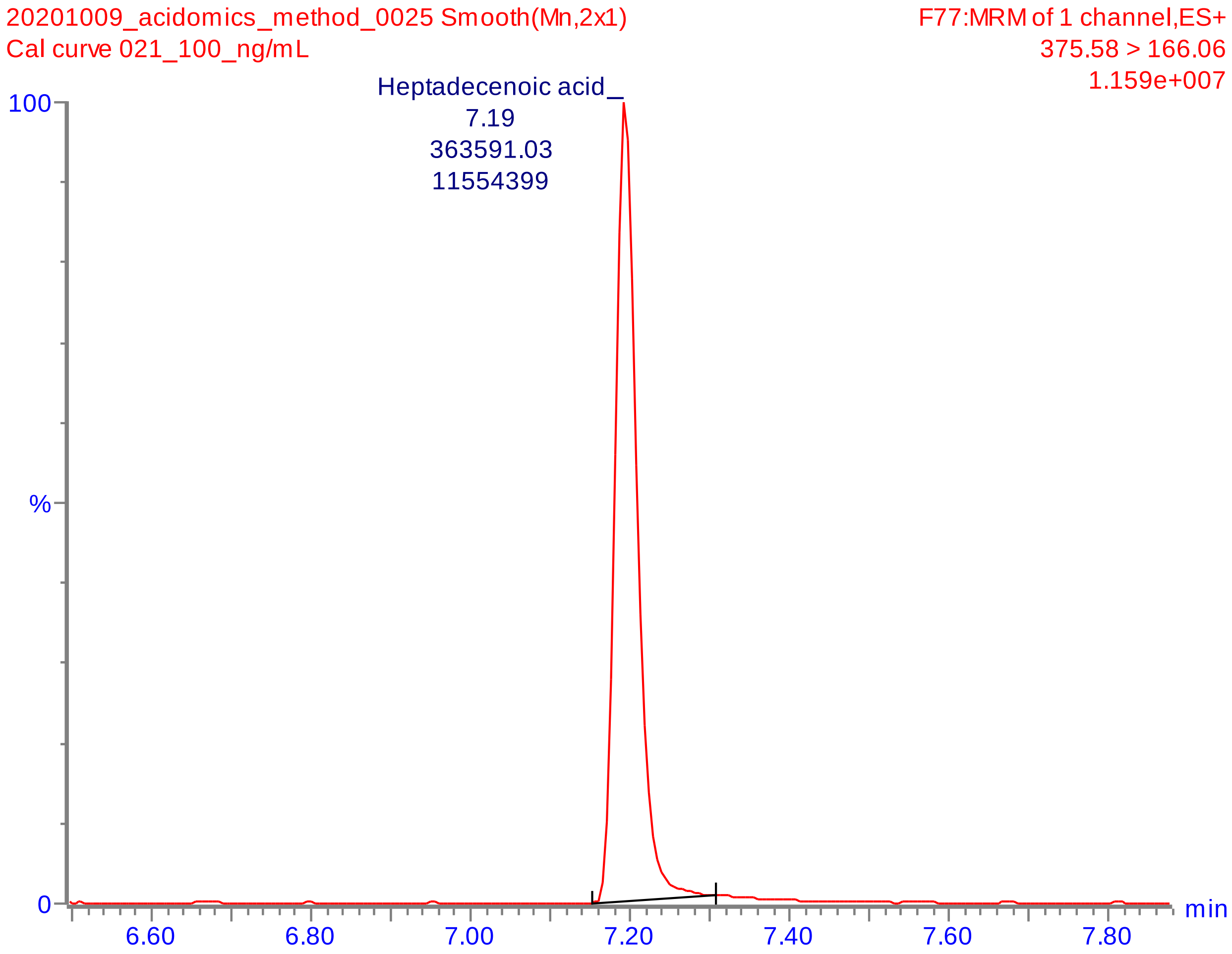

20201009_acidomics_method_0025 Smooth(Mn,2x1)
Cal curve 021_100_ng/mL
F77:MRM of 1 channel,ES+
375.58 > 166.06
1.159e+007
Heptadecenoic acid_
7.19
363591.03
11554399
100
%
0
6.60
6.80
7.00
7.20
7.40
7.60
7.80
min

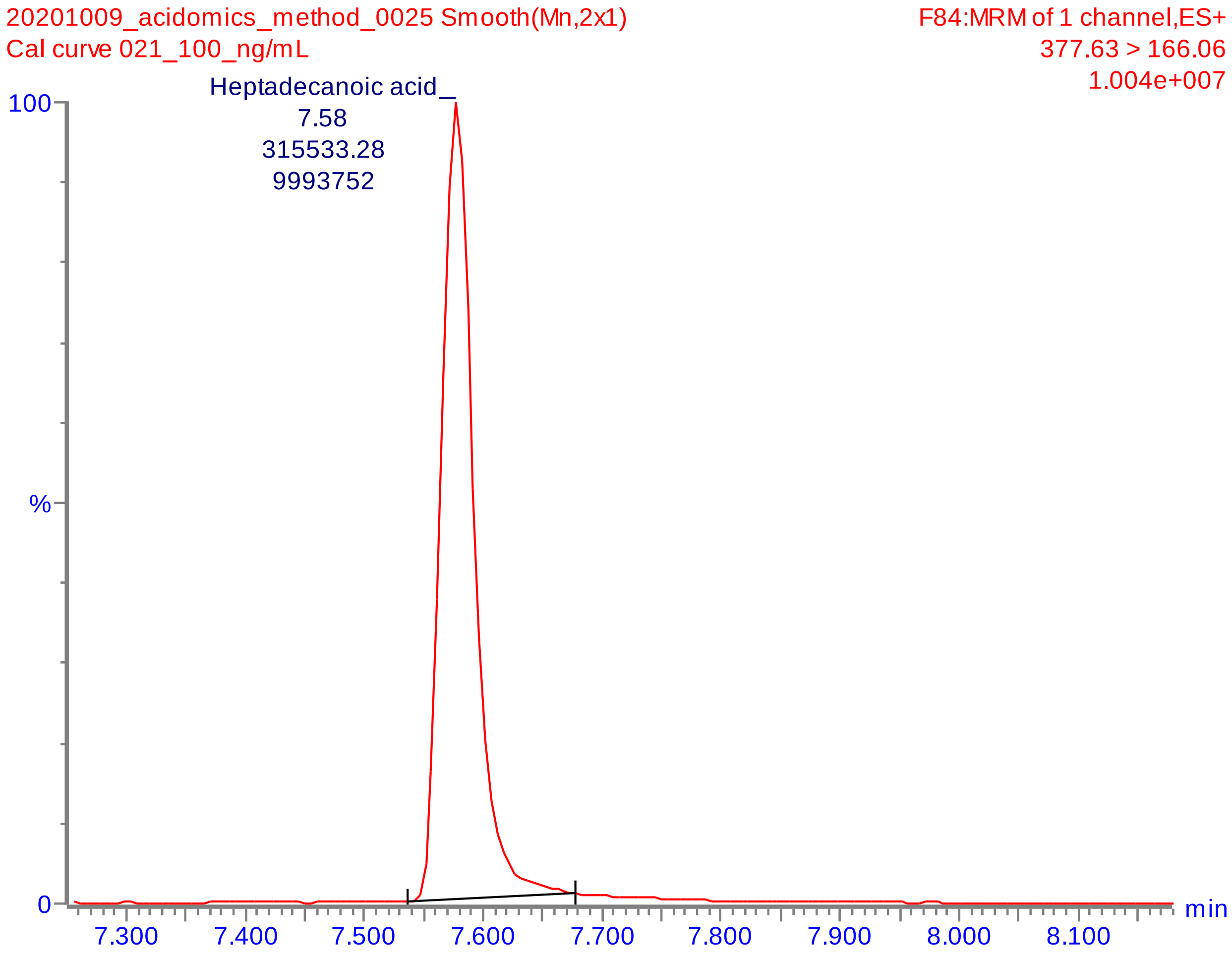

20201009_acidomics_method_0025 Smooth(Mn,2x1)
Cal curve 021_100_ng/mL
F84:MRM of 1 channel,ES+
377.63 > 166.06
1.004e+007
Heptadecanoic acid
7.58
315533.28
9993752
100
%
0
7.300
7.400
7.500
7.600
7.700
7.800
7.900
8.000
8.100
min

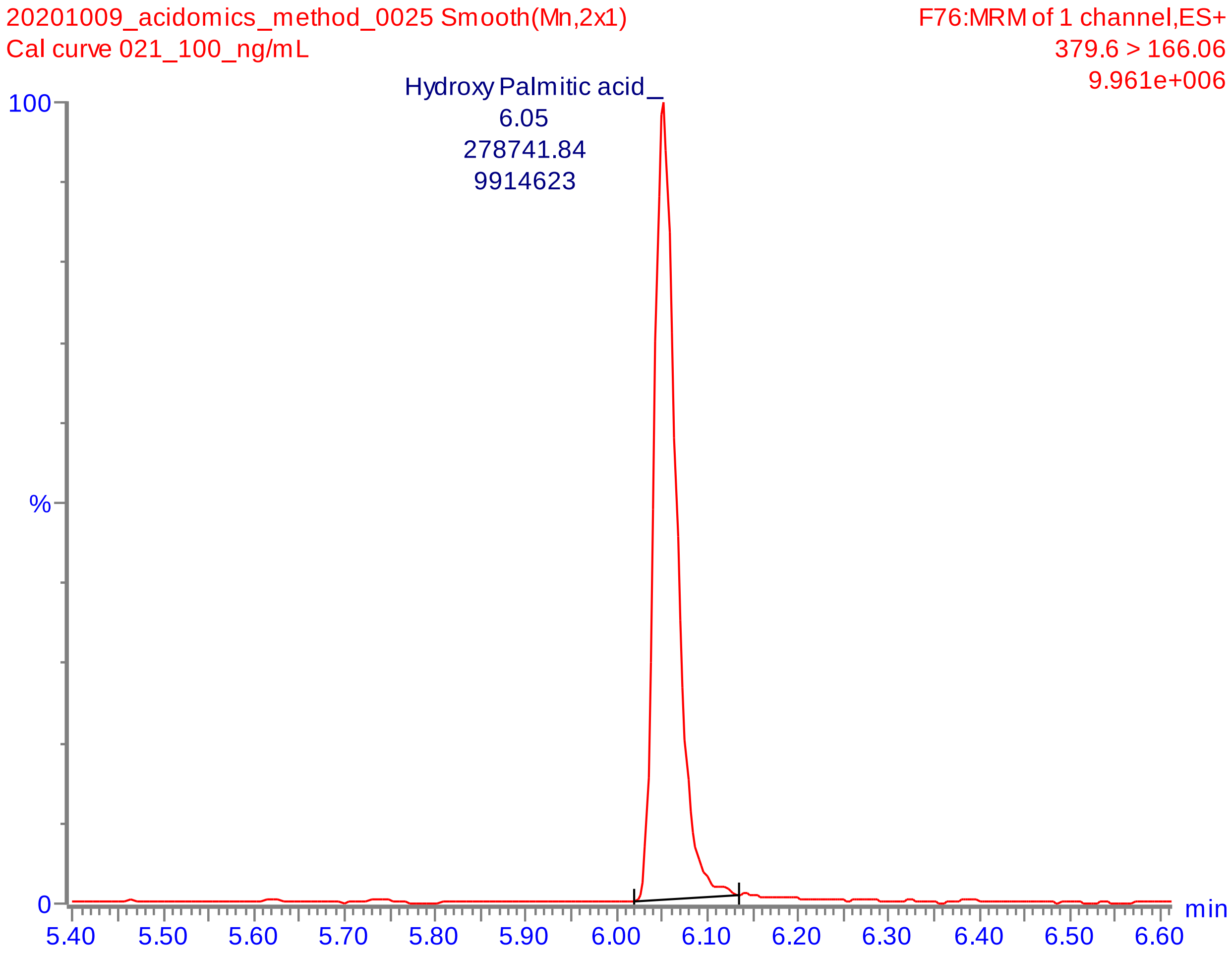

20201009_acidomics_method_0025 Smooth(Mn,2x1)
Cal curve 021_100_ng/mL
F76:MRM of 1 channel,ES+
379.6 > 166.06
9.961e+006
Hydroxy Palmitic acid_
6.05
278741.84
9914623
100
%
0
min
5.40
5.50
5.60
5.70
5.80
5.90
6.00
6.10
6.20
6.30
6.40
6.50
6.60

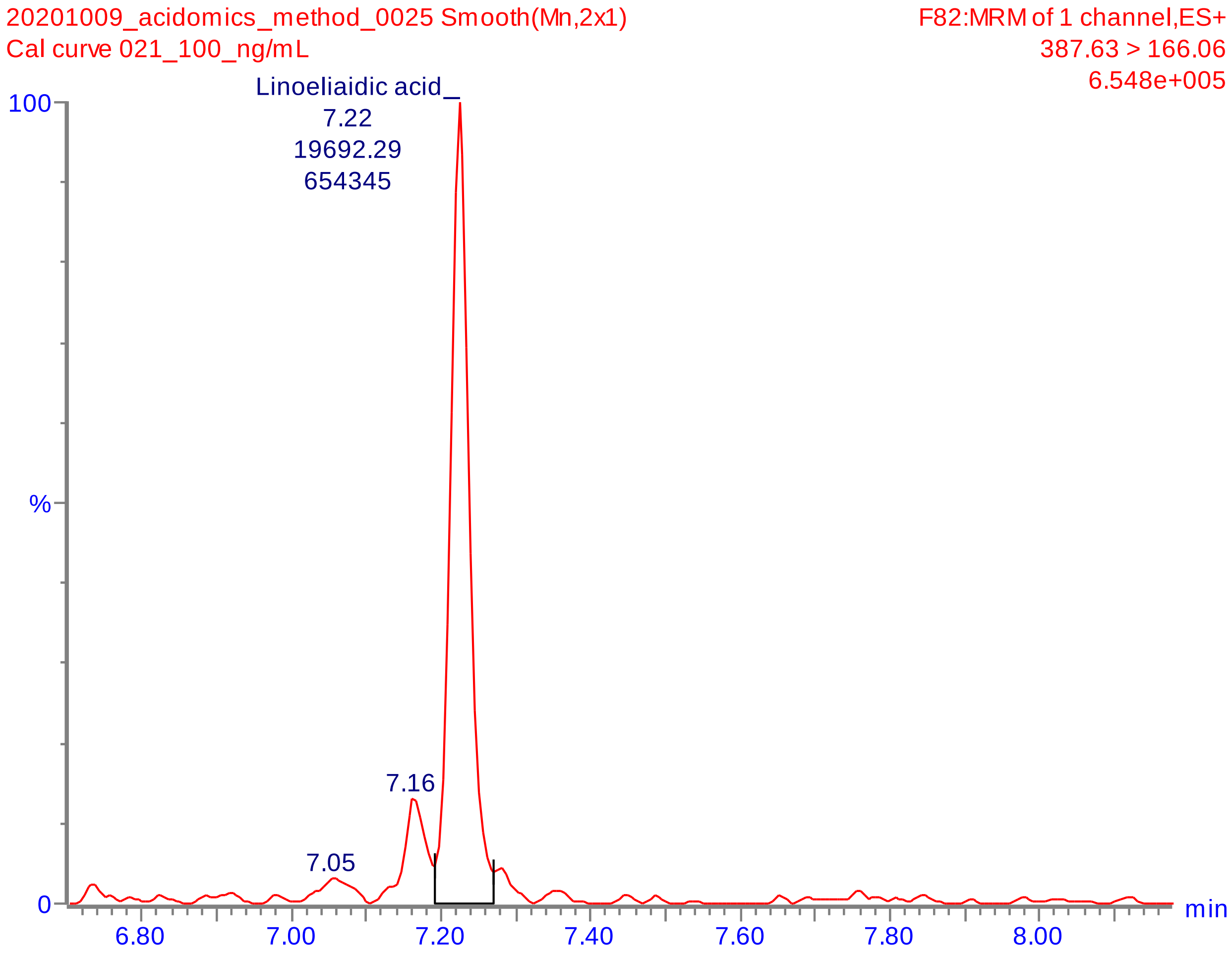

20201009_acidomics_method_0025 Smooth(Mn,2x1)
Cal curve 021_100_ng/mL
F82:MRM of 1 channel,ES+
387.63 > 166.06
6.548e+005
Linoeliaidic acid
7.22
19692.29
654345
7.16
7.05
100
%
0
6.80
7.00
7.20
7.40
7.60
7.80
8.00
min

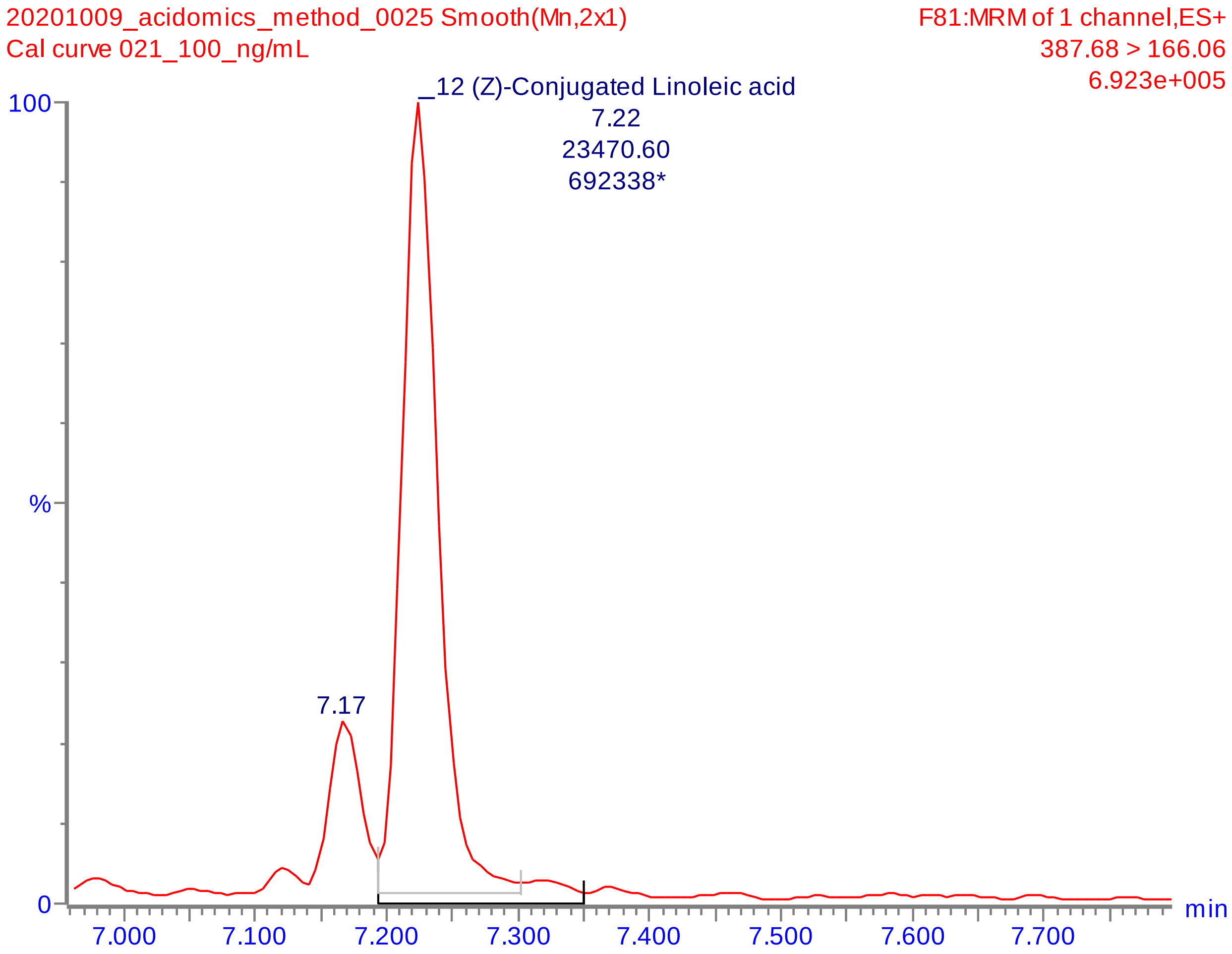
20201009_acidomics_method_0025 Smooth(Mn,2x1)
Cal curve 021_100_ng/mL
F81:MRM of 1 channel,ES+
387.68 > 166.06
6.923e+005
_12 (Z)-Conjugated Linoleic acid
7.22
23470.60
692338*
7.17
100
%
0
7.000
7.100
7.200
7.300
7.400
7.500
7.600
7.700
min

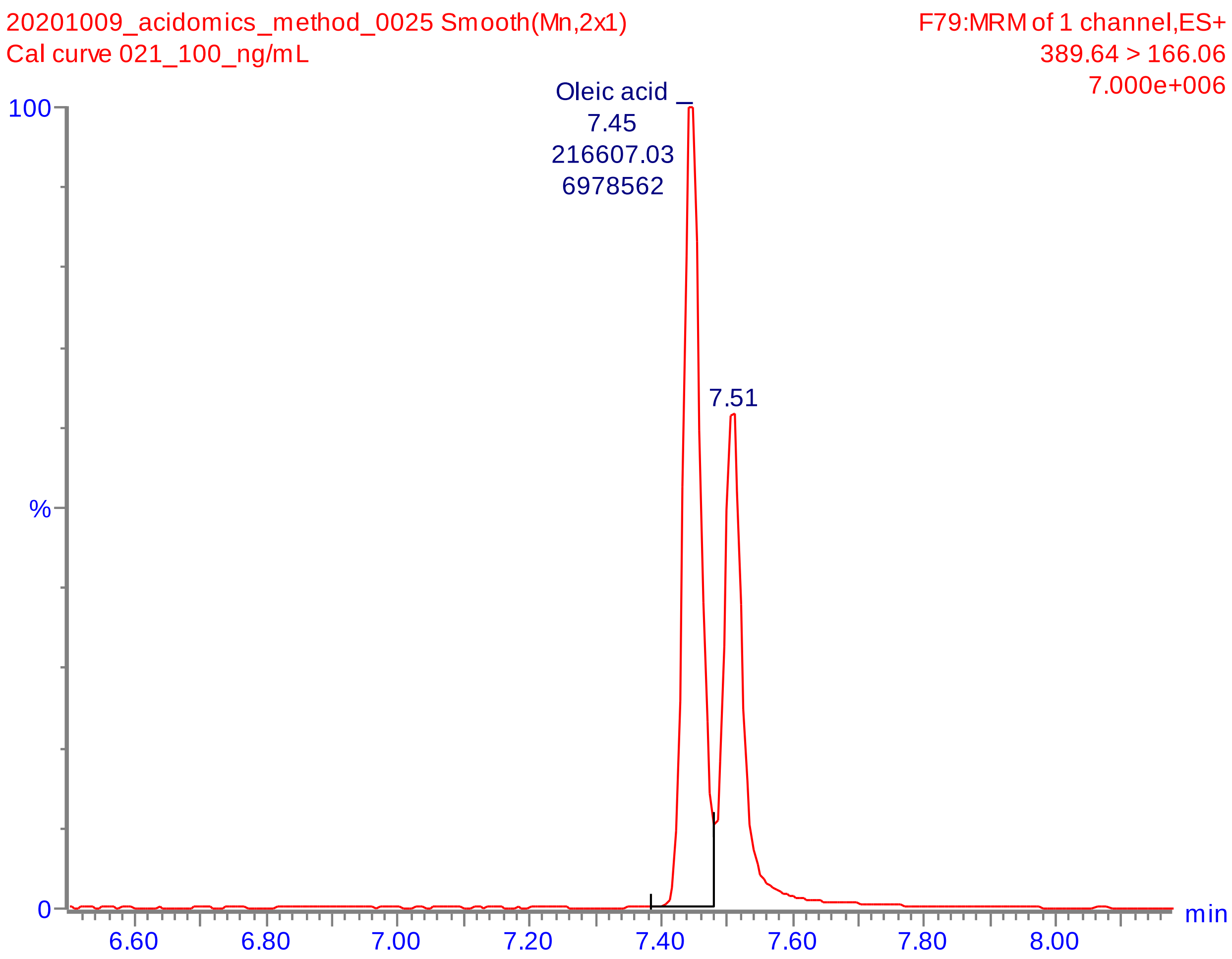

20201009_acidomics_method_0025 Smooth(Mn,2x1)
Cal curve 021_100_ng/mL
F79:MRM of 1 channel,ES+
389.64 > 166.06
7.000e+006
Oleic acid
7.45
216607.03
6978562
7.51
100
%
0
6.60
6.80
7.00
7.20
7.40
7.60
7.80
8.00
min

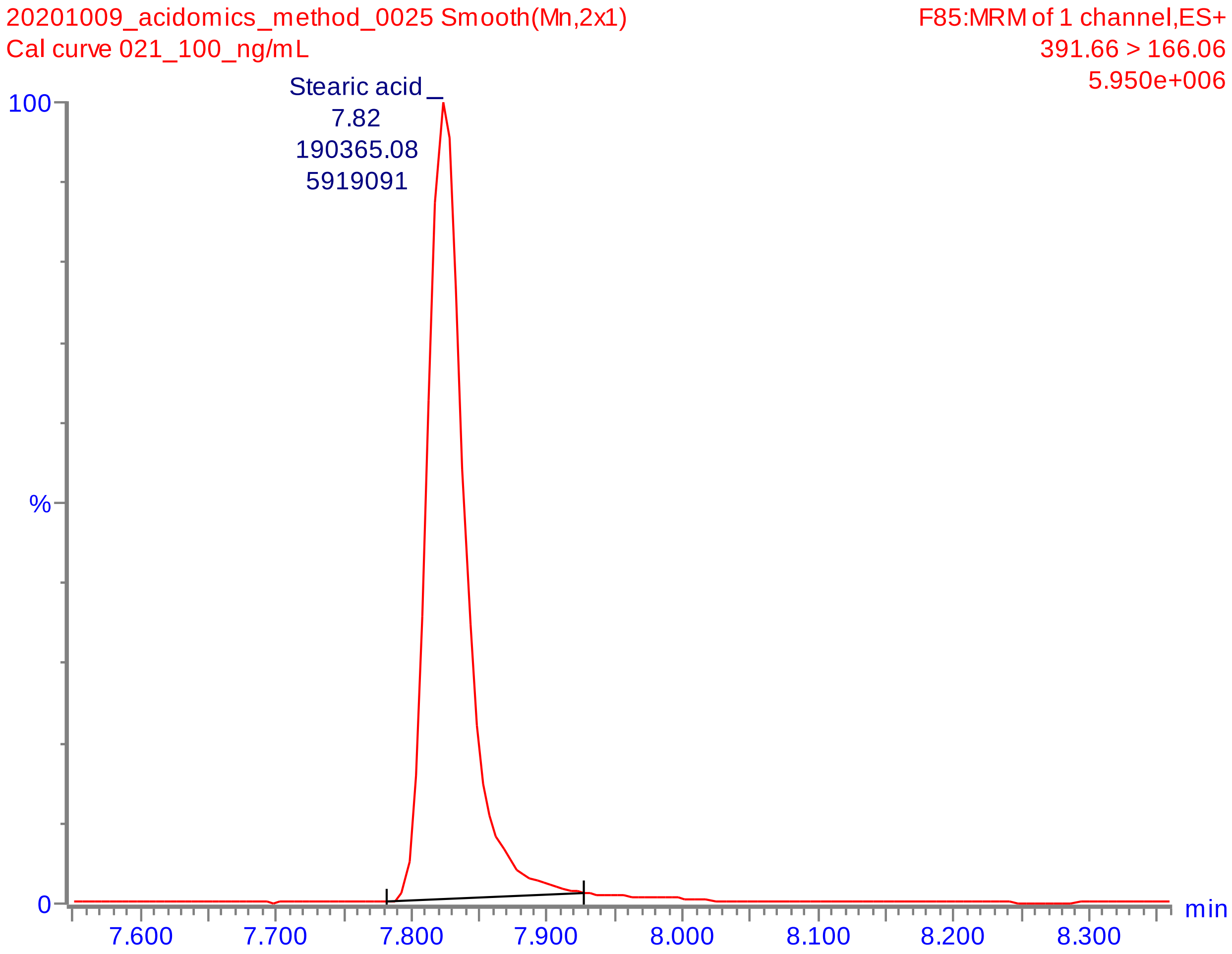

20201009_acidomics_method_0025 Smooth(Mn,2x1)
Cal curve 021_100_ng/mL
F85:MRM of 1 channel,ES+
391.66 > 166.06
5.950e+006
Stearic acid
7.82
190365.08
5919091
100
%
0
7.600
7.700
7.800
7.900
8.000
8.100
8.200
8.300
min

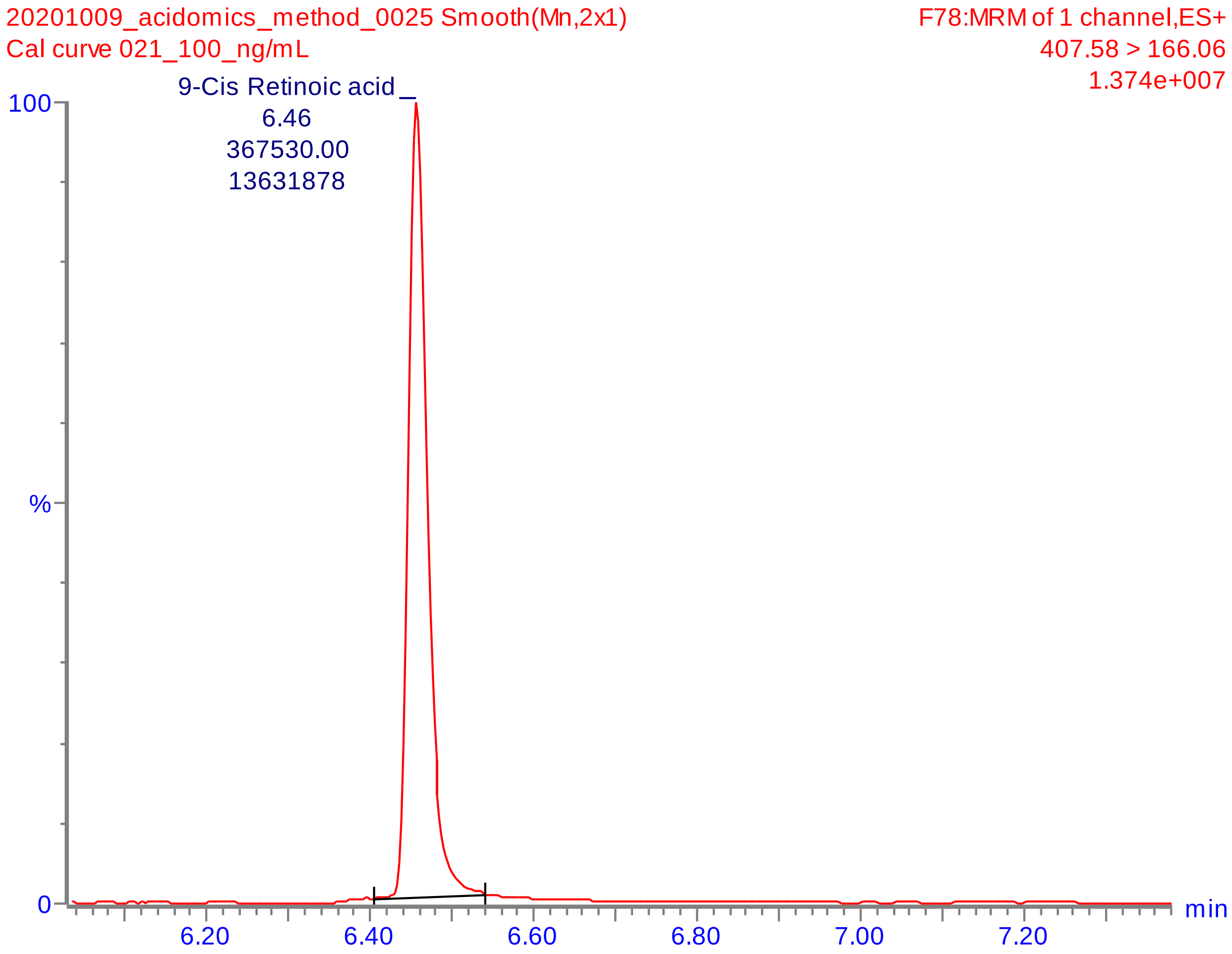

20201009_acidomics_method_0025 Smooth(Mn,2x1)
Cal curve 021_100_ng/mL
F78:MRM of 1 channel,ES+
407.58 > 166.06
1.374e+007
9-Cis Retinoic acid
6.46
367530.00
13631878
100
%
0
6.20
6.40
6.60
6.80
7.00
7.20
min

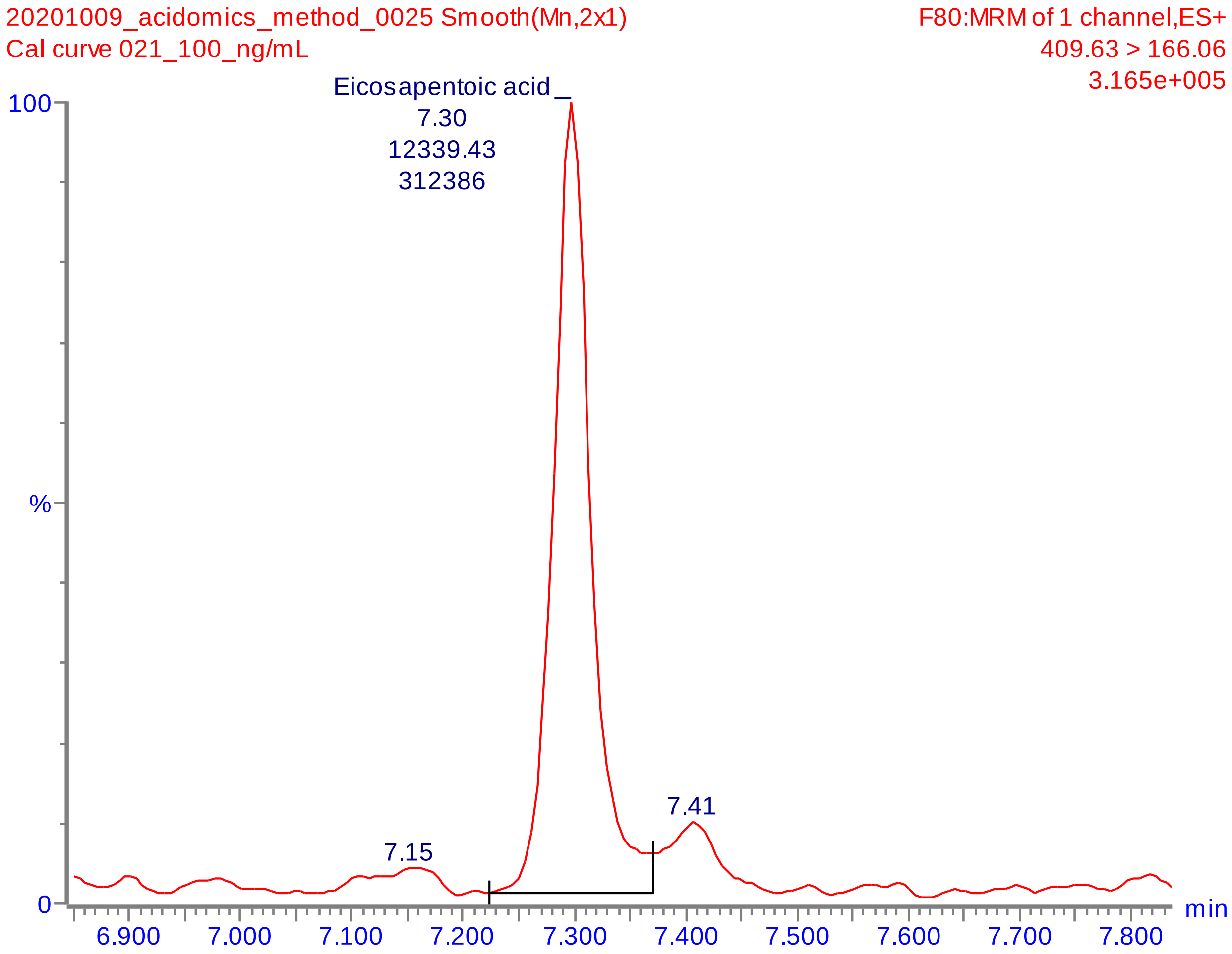
20201009_acidomics_method_0025 Smooth(Mn,2x1)
Cal curve 021_100_ng/mL
F80:MRM of 1 channel,ES+
409.63 > 166.06
3.165e+005
Eicosapentoic acid
7.30
12339.43
312386
7.41
7.15
100
%
0
6.900
7.000
7.100
7.200
7.300
7.400
7.500
7.600
7.700
7.800
min

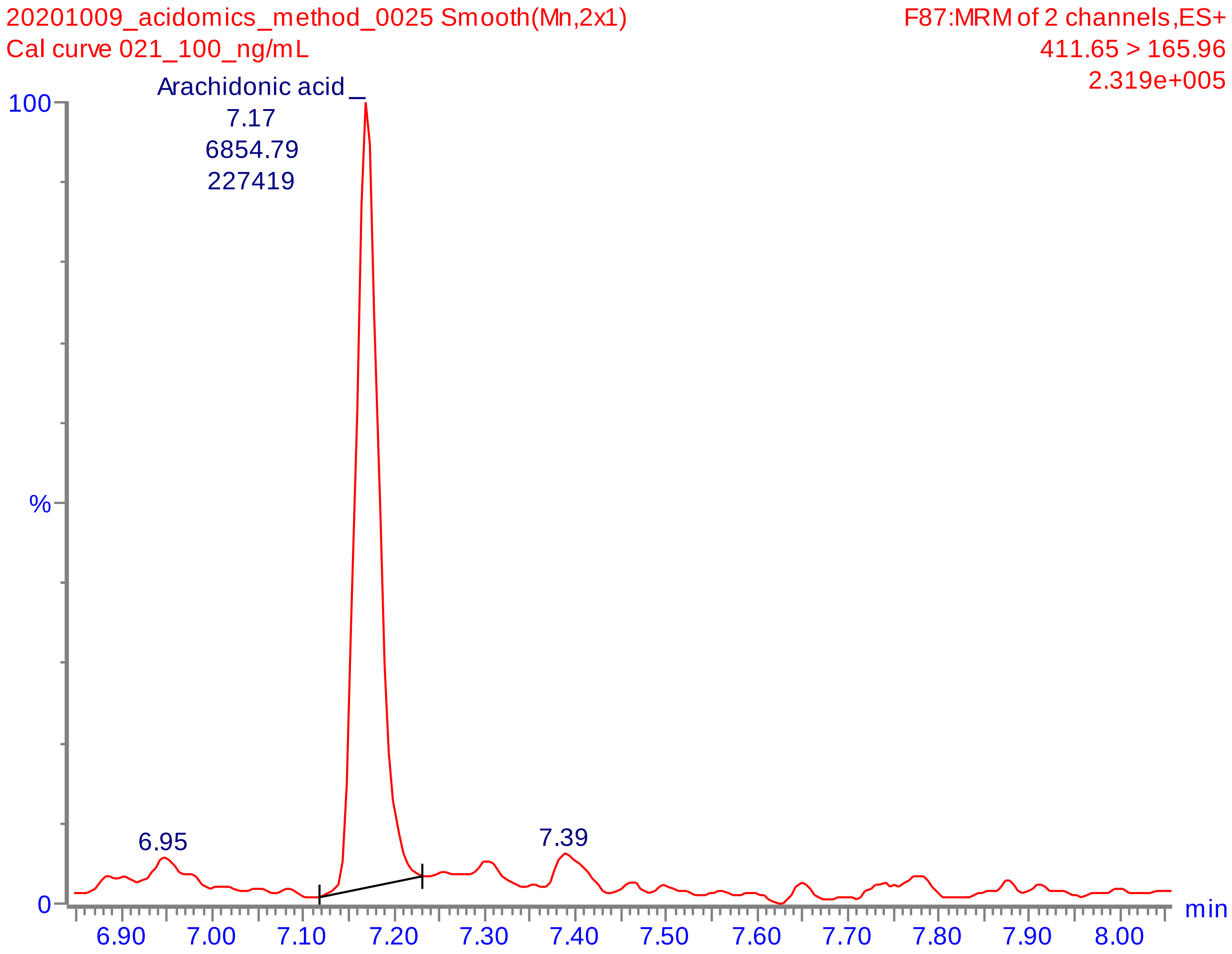

20201009_acidomics_method_0025 Smooth(Mn,2x1)
Cal curve 021_100_ng/mL
F87:MRM of 2 channels,ES+
411.65 > 165.96
2.319e+005
Arachidonic acid
7.17
6854.79
227419
100
%
0
6.95
7.39
6.90
7.00
7.10
7.20
7.30
7.40
7.50
7.60
7.70
7.80
7.90
8.00
min

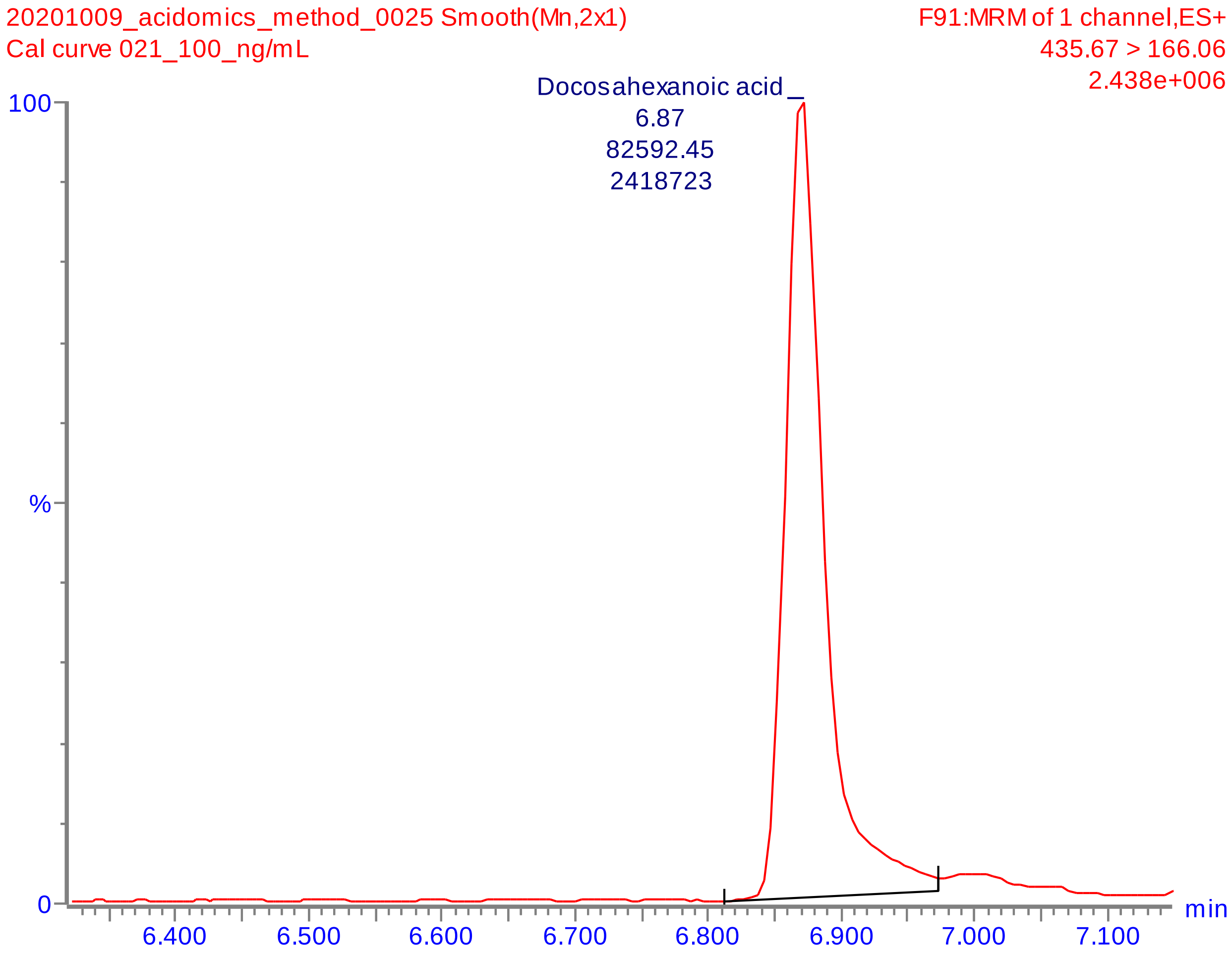

20201009_acidomics_method_0025 Smooth(Mn,2x1)
Cal curve 021_100_ng/mL
F91:MRM of 1 channel,ES+
435.67 > 166.06
2.438e+006
Docosahexanoic acid
6.87
82592.45
2418723
100
%
0
6.400
6.500
6.600
6.700
6.800
6.900
7.000
7.100
min

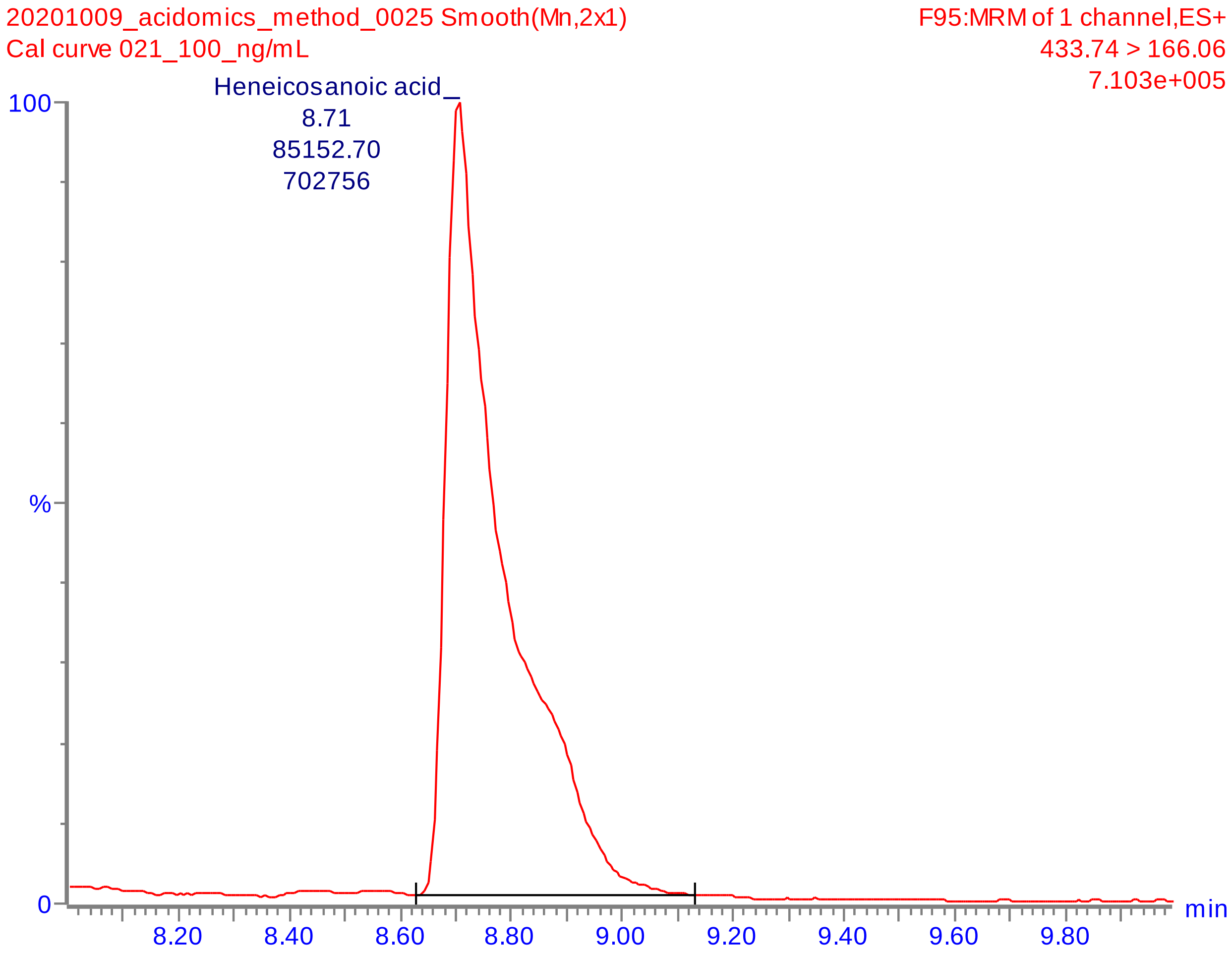
20201009_acidomics_method_0025 Smooth(Mn,2x1)
Cal curve 021_100_ng/mL
F95:MRM of 1 channel,ES+
433.74 > 166.06
7.103e+005
Heneicosanoic acid_
8.71
85152.70
702756
100
%
0
8.20
8.40
8.60
8.80
9.00
9.20
9.40
9.60
9.80
min

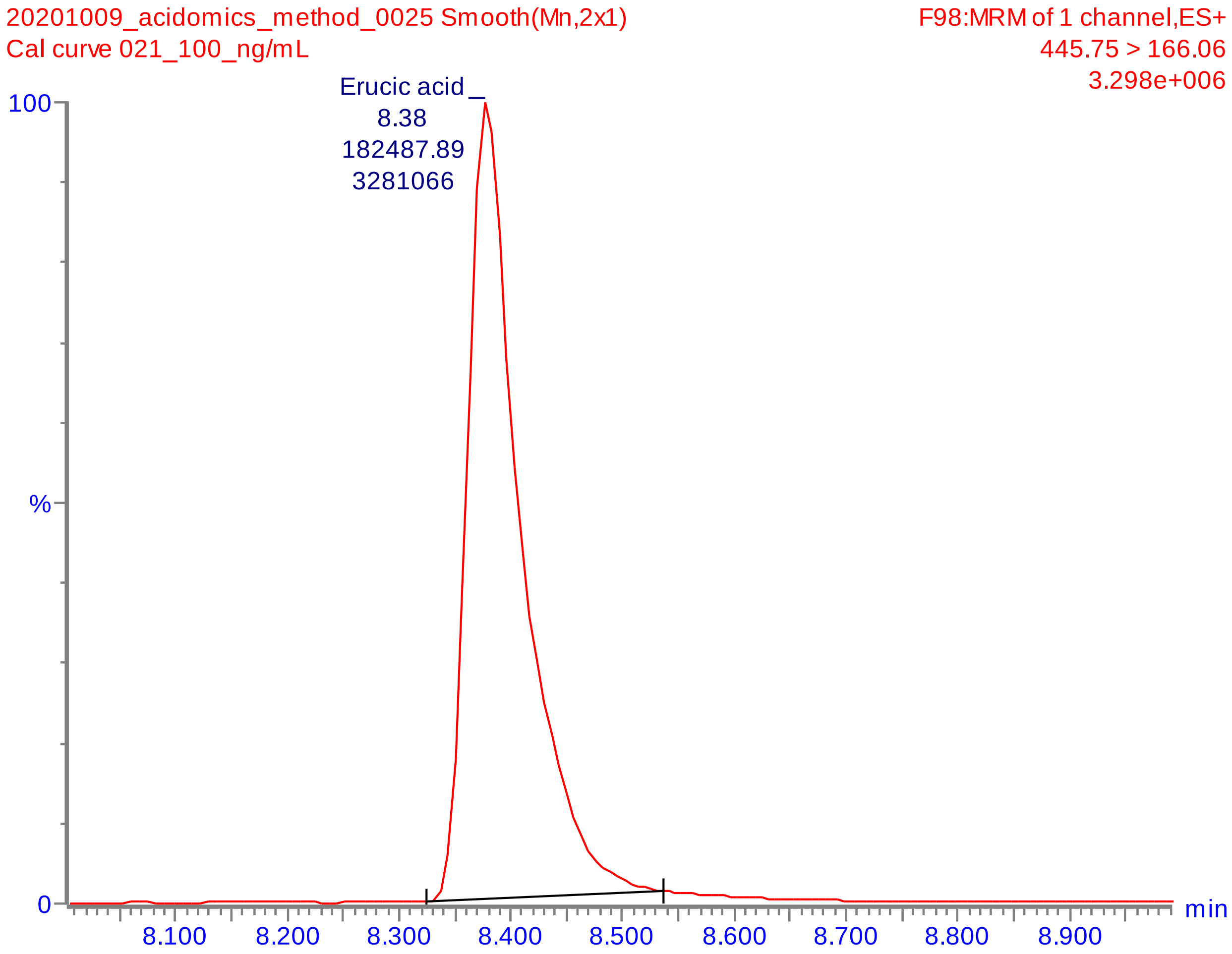

20201009_acidomics_method_0025 Smooth(Mn,2x1)
Cal curve 021_100_ng/mL
F98:MRM of 1 channel,ES+
445.75 > 166.06
3.298e+006
Erucic acid
8.38
182487.89
3281066
100
%
0
8.100
8.200
8.300
8.400
8.500
8.600
8.700
8.800
8.900
min

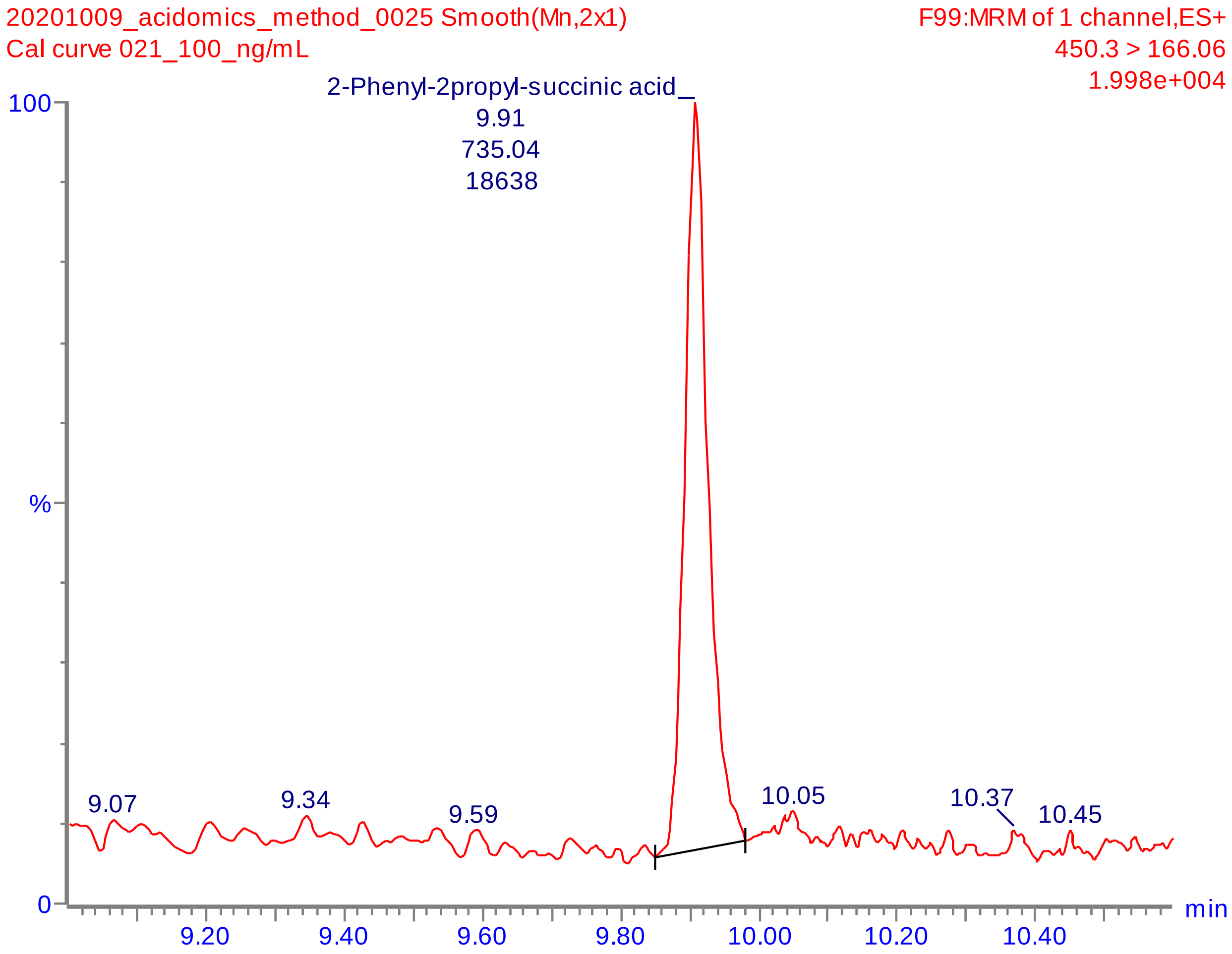

20201009_acidomics_method_0025 Smooth(Mn,2x1)
Cal curve 021_100_ng/mL
F99:MRM of 1 channel,ES+
450.3 > 166.06
1.998e+004
2-Phenyl-2propyl-succinic acid
9.91
735.04
18638
100
%
0
9.07
9.34
9.59
10.05
10.37
10.45
9.20
9.40
9.60
9.80
10.00
10.20
10.40
min

Supplementary Figure S3. Standard curve of selected carboxylic acids

Compound name: Glyceric acid
Correlation coefficient: r = 0.998642, r^2 = 0.997286
Calibration curve: 0.00512451 * x + 0.177688
Response type: Internal Std (Ref 2), Area * (IS Conc. / IS Area)
Curve type: Linear, Origin: Exclude, Weighting: 1/x, Axis trans: None

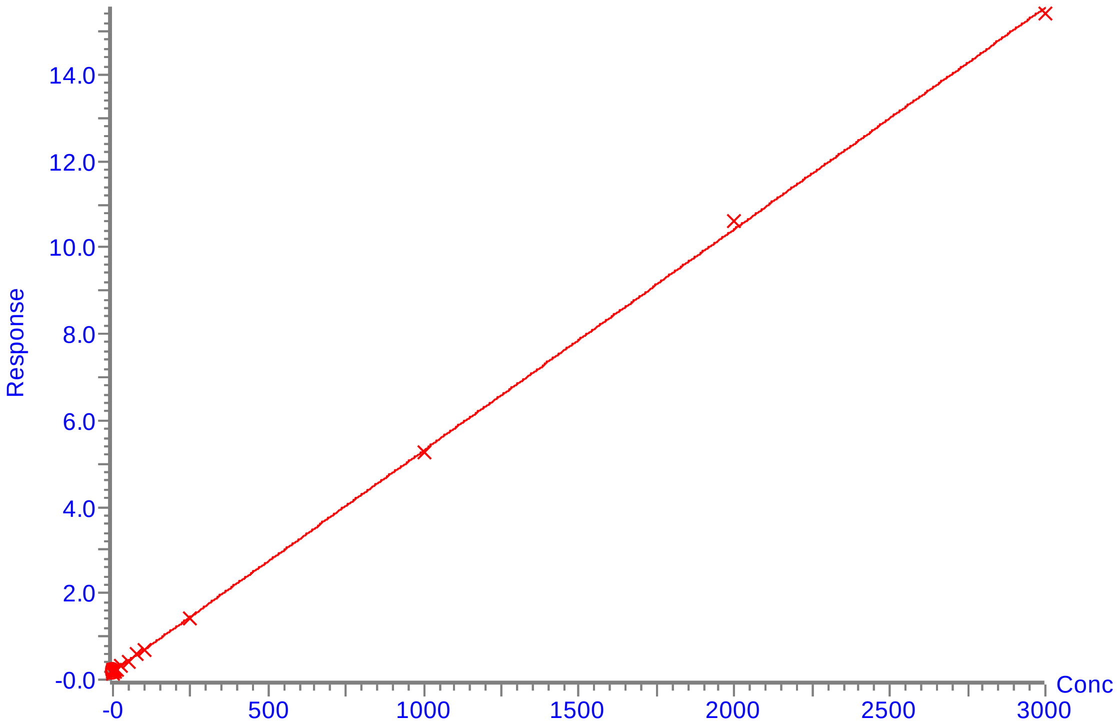

Glyceric acid					
	Std Conc (ng/mL)	Response	Conc.	%Dev	S/N
1	2.5	0.191	2.6	5.5	2189.101
2	5	0.203	5	0.2	1896.329
3	7.5	0.215	7.3	-2.3	2614.522
4	10	0.23	10.2	2	1392.784
5	25	0.309	25.7	2.7	3452.051
6	50	0.429	49.1	-1.8	2412.603
7	75	0.573	77.2	2.9	4630.4
8	100	0.678	97.6	-2.4	3252.067
9	250	1.431	244.6	-2.1	6490.712
10	1000	5.265	992.8	-0.7	2256.179
11	2000	10.615	2036.7	1.8	3613.909
12	3000	15.408	2972	-0.9	6370.232

Compound name: Glyoxalic acid_1
Coefficient of Determination: R^2 = 0.998949
Calibration curve: 4.88297e-010 * x^2 + 0.000124527 * x + 9.31703e-005
Response type: Internal Std (Ref 2), Area * (IS Conc. / IS Area)
Curve type: 2nd Order, Origin: Exclude, Weighting: 1/x, Axis trans: None

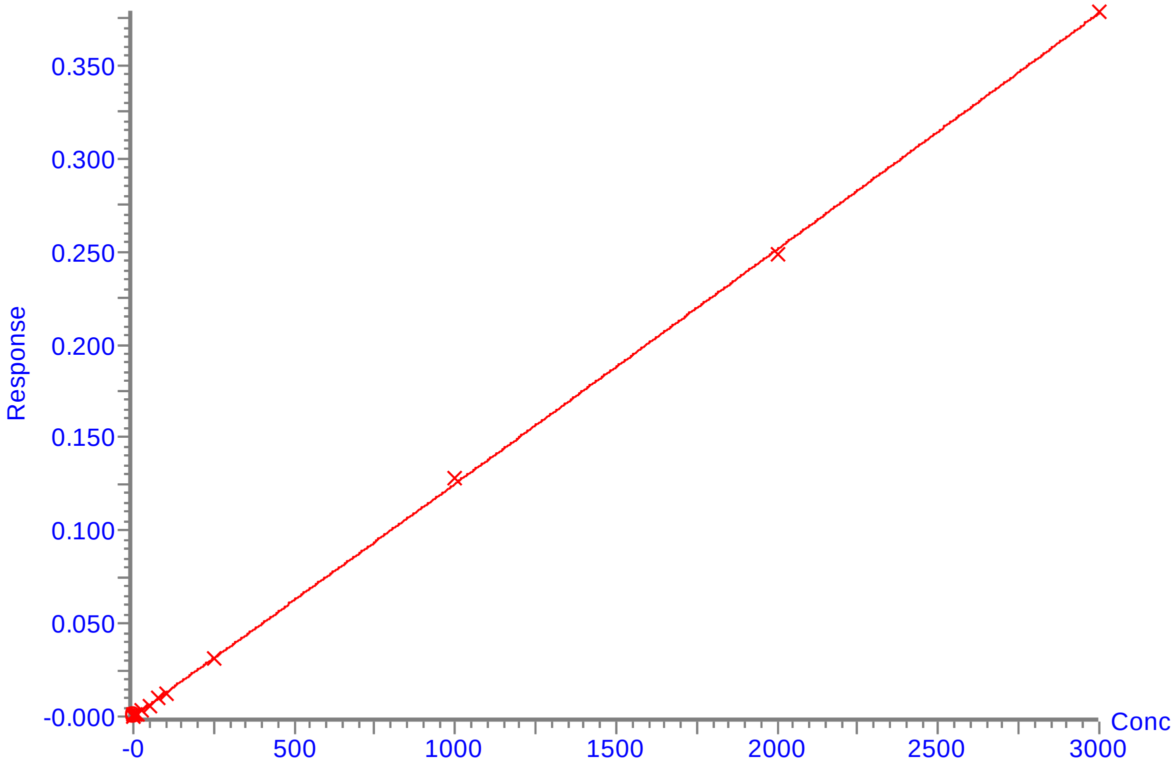

Glyoxalic acid					
	Std Conc (ng/mL)	Response	Conc.	%Dev	S/N
1	5	0.001	5.4	7.9	49.695
2	7.5	0.001	7.4	-1.7	10.554
3	10	0.002	11.6	15.6	9.341
4	25	0.003	23.6	-5.8	16.438
5	50	0.006	47.8	-4.3	69.854
6	75	0.01	75.6	0.8	52.901
7	100	0.012	94.5	-5.5	72.647
8	250	0.031	246.9	-1.2	79.599
9	1000	0.129	1028.1	2.8	133.726
10	2000	0.248	1977.7	-1.1	221.925
11	3000	0.379	3006	0.2	196.583

Compound name: Propionic acid
Correlation coefficient: r = 0.999070, r^2 = 0.998140
Calibration curve: 0.00592889 * x + 0.0184697
Response type: Internal Std (Ref 2), Area * (IS Conc. / IS Area)
Curve type: Linear, Origin: Exclude, Weighting: 1/x, Axis trans: None

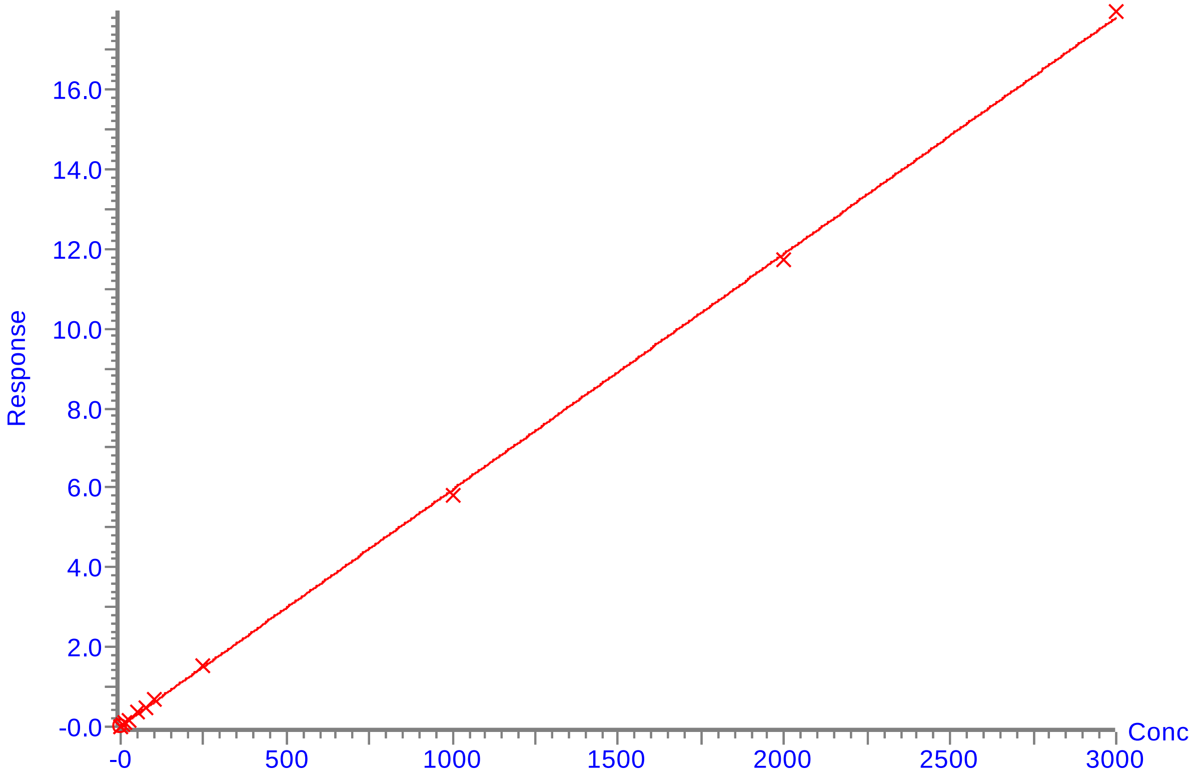

Propionic acid					
	Std Conc (ng/mL)	Response	Conc.	%Dev	S/N
1	5	0.05	5.3	5.4	97.87
2	7.5	0.064	7.7	3	226.611
3	10	0.087	11.6	15.8	139.49
4	25	0.165	24.6	-1.4	202.447
5	50	0.346	55.3	10.6	769.14
6	75	0.468	75.9	1.1	460.501
7	100	0.706	116	16	495.198
8	250	1.508	251.2	0.5	618.883
9	1000	5.832	980.5	-1.9	1632.454
10	2000	11.717	1973.2	-1.3	2998.916

Compound name: Pyruvic acid_179
Correlation coefficient: r = 0.999830, r^2 = 0.999661
Calibration curve: 0.00892922 * x + 1.12112
Response type: Internal Std (Ref 2), Area * (IS Conc. / IS Area)
Curve type: Linear, Origin: Exclude, Weighting: 1/x, Axis trans: None

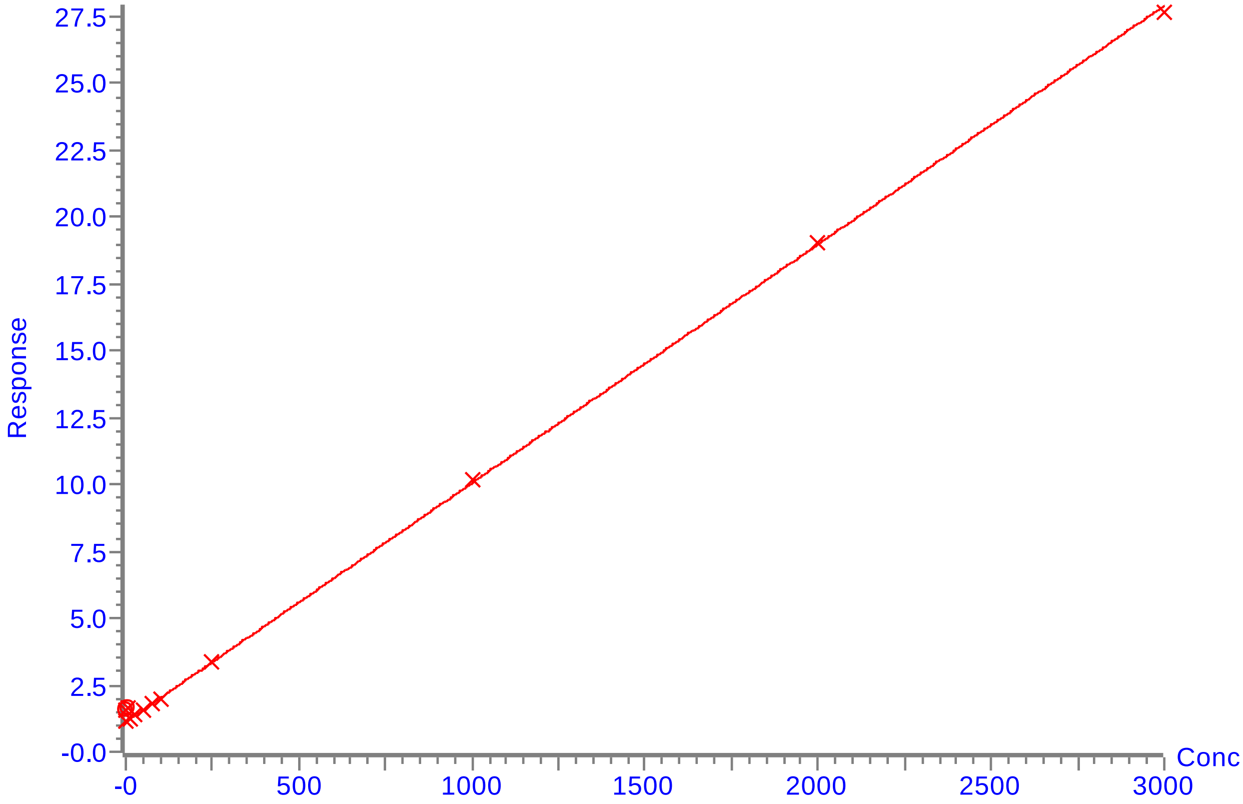

Pyruvic acid					
	Std Conc (ng/mL)	Response	Conc.	%Dev	S/N
1	10	1.235	12.8	28	826.63
2	25	1.368	27.7	10.7	2553.92
3	50	1.596	53.2	6.3	3007.967
4	75	1.791	75.1	0.1	7938.929
5	100	2.007	99.2	-0.8	2185.92
6	250	3.366	251.4	0.6	8542.873
7	1000	10.152	1011.4	1.1	7546.01
8	2000	19.029	2005.5	0.3	10478.77
9	3000	27.685	2975	-0.8	7560.999

Compound name: Alanine
Correlation coefficient: r = 0.999164, r^2 = 0.998328
Calibration curve: 0.000776941 * x + 0.0917322
Response type: Internal Std (Ref 2), Area * (IS Conc. / IS Area)
Curve type: Linear, Origin: Exclude, Weighting: 1/x, Axis trans: None

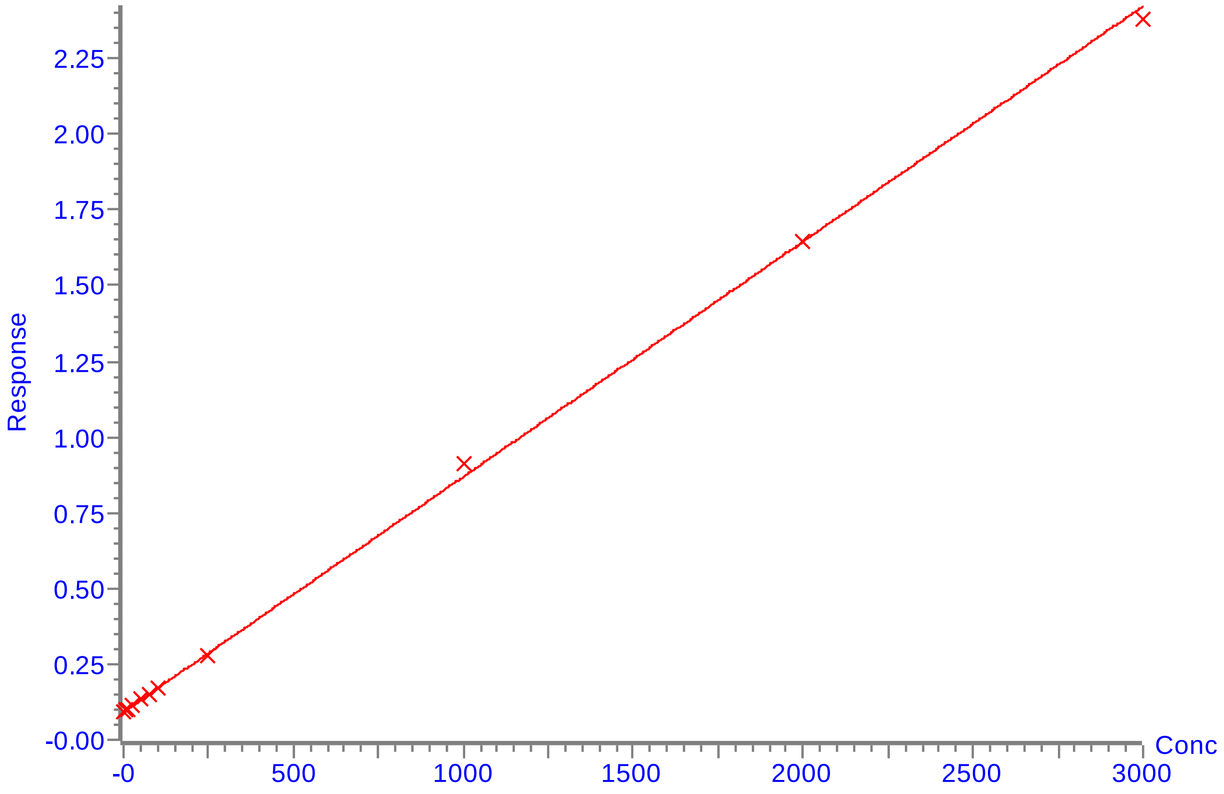

Alanine					
	Std Conc (ng/mL)	Response	Conc.	%Dev	S/N
1	5	0.096	5.9	18.2	750.615
2	7.5	0.097	7.4	-1.1	592.909
3	10	0.101	11.6	15.8	246.039
4	25	0.114	29	16	285.662
5	50	0.135	55.6	11.2	361.211
6	75	0.151	76.6	2.1	504.953
7	100	0.167	97.5	-2.5	339.665
8	250	0.279	241.1	-3.5	469.805
9	1000	0.915	1059.8	6	1901.629
10	2000	1.642	1995.9	-0.2	1245.058
11	3000	2.377	2941.4	-2	484.933

Compound name: 2-oxo-butyric acid
Correlation coefficient: r = 0.996540, r^2 = 0.993091
Calibration curve: 0.00241346 * x + 0.0534511
Response type: Internal Std (Ref 2), Area * (IS Conc. / IS Area)
Curve type: Linear, Origin: Exclude, Weighting: 1/x, Axis trans: None

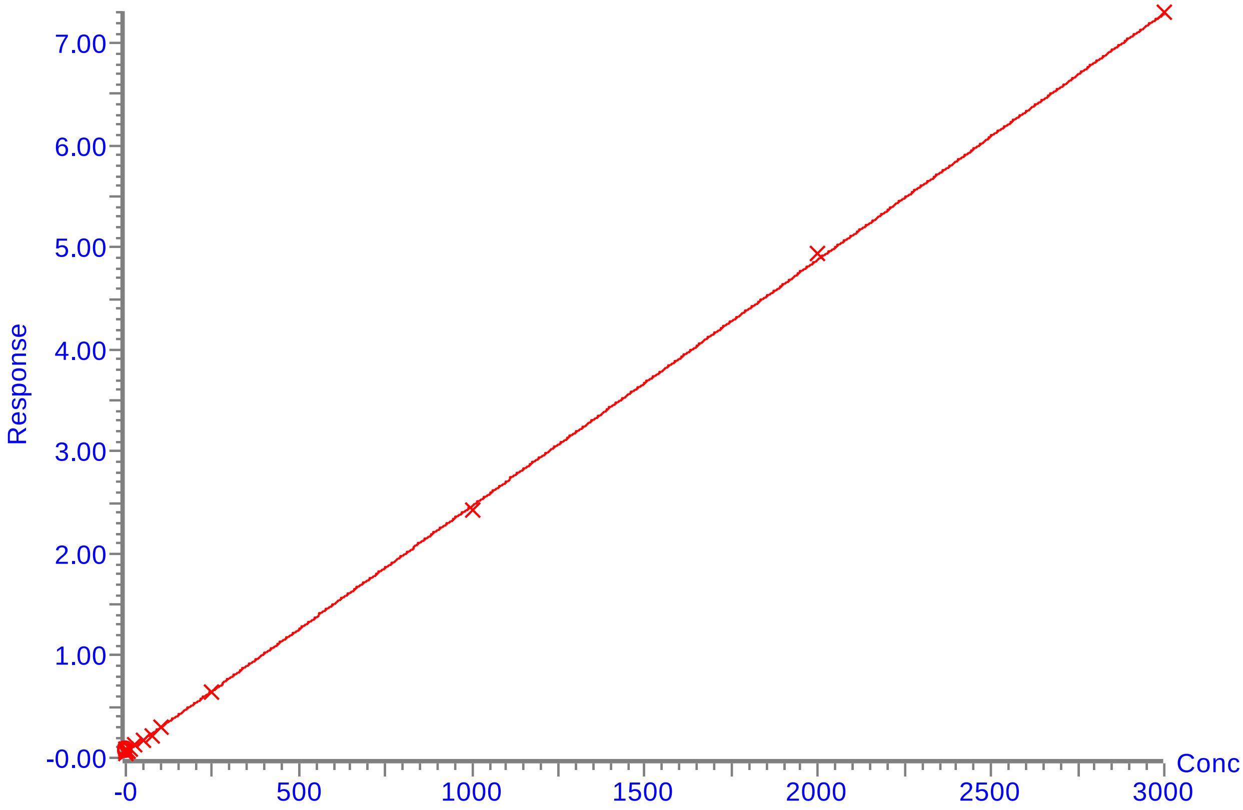

2-oxo-butyric acid					
	Std Conc (ng/mL)	Response	Conc.	%Dev	S/N
1	2.5	0.06	2.7	7.6	146.685
2	5	0.066	5.1	2.4	220.804
3	7.5	0.07	6.9	-8.2	152.087
4	10	0.082	11.8	17.7	237.307
5	50	0.172	49.2	-1.5	306.963
6	75	0.22	69	-8	308.994
7	100	0.296	100.3	0.3	422.116
8	250	0.636	241.5	-3.4	580.161
9	1000	2.429	984.3	-1.6	1786.738
10	2000	4.939	2024.3	1.2	1054.447
11	3000	7.305	3004.7	0.2	753.71

Compound name: Malonic acid_152
Correlation coefficient: r = 0.999713, r^2 = 0.999426
Calibration curve: 0.000576929 * x + 0.0834816
Response type: Internal Std (Ref 2), Area * (IS Conc. / IS Area)
Curve type: Linear, Origin: Exclude, Weighting: 1/x, Axis trans: None

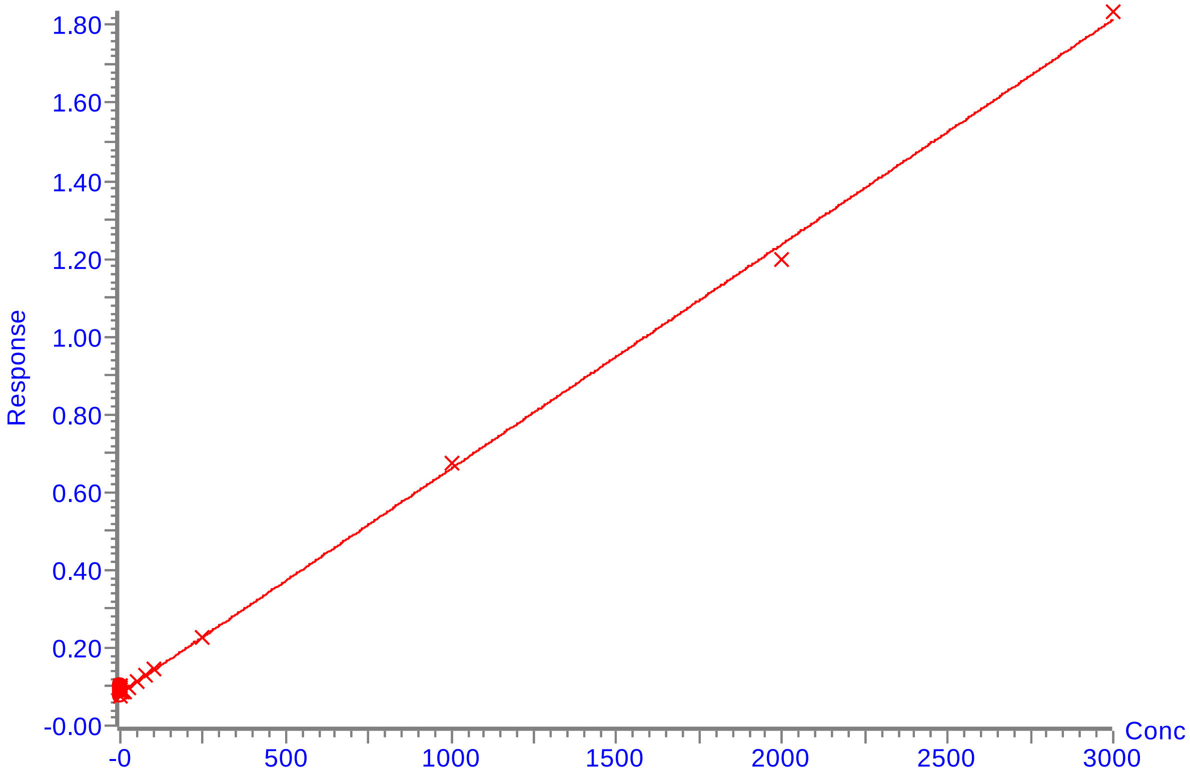

Malonic acid					
	Std Conc (ng/mL)	Response	Conc.	%Dev	S/N
1	1	0.084	1.1	8.8	372.092
2	2.5	0.085	2.2	-13.4	257.139
3	5	0.086	5.1	2.4	563.668
4	7.5	0.088	7.2	-4.6	527.207
5	10	0.089	9.4	-6.5	290.84
6	25	0.099	26.9	7.5	837.124
7	50	0.111	48	-4	377.3
8	75	0.129	78.4	4.6	581.593
9	100	0.144	104.2	4.2	276.342
10	250	0.229	252.3	0.9	445.655
11	1000	0.673	1021.3	2.1	622.213
12	2000	1.2	1934.6	-3.3	1279.919
13	3000	1.835	3035.4	1.2	1215.216

Compound name: Serine
Correlation coefficient: r = 0.999286, r^2 = 0.998573
Calibration curve: 8.93123e-005 * x + 0.0170367
Response type: Internal Std (Ref 2), Area * (IS Conc. / IS Area)
Curve type: Linear, Origin: Exclude, Weighting: 1/x, Axis trans: None

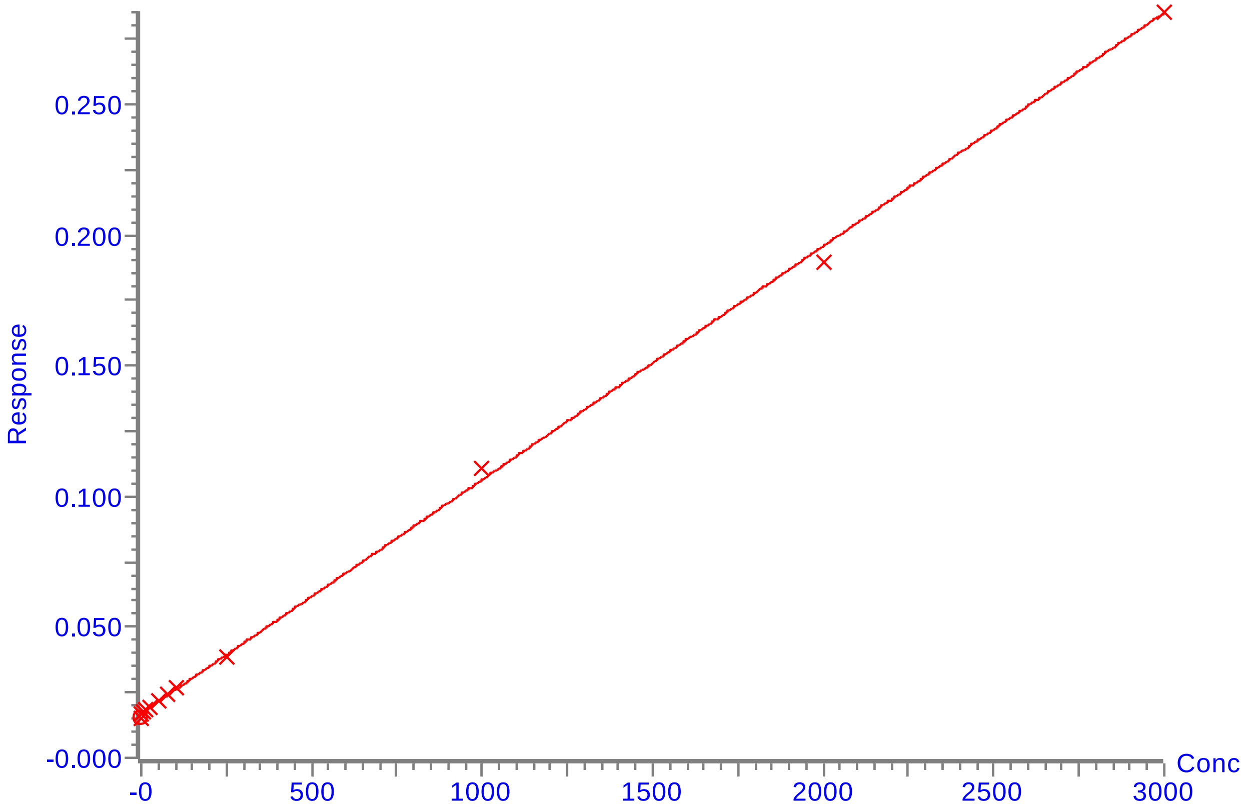

Serine					
	Std Conc (ng/mL)	Response	Conc.	%Dev	S/N
1	5	0.018	5.7	14.1	89.795
2	10	0.018	11.4	13.8	71.837
3	25	0.019	24	-3.9	99.64
4	50	0.022	57	14.1	124.253
5	75	0.024	77.2	2.9	84.875
6	100	0.027	106	6	100.234
7	250	0.039	245.9	-1.6	88.719
8	1000	0.111	1052	5.2	175.052
9	2000	0.19	1933	-3.4	299.31
10	3000	0.285	3002.3	0.1	127.662

Compound name: 4-Me-2-oxo-pentanoic acid
Correlation coefficient: r = 0.998025, r^2 = 0.996054
Calibration curve: 2.44122 * x + -12.8998
Response type: Internal Std (Ref 9), Area * (IS Conc. / IS Area)
Curve type: Linear, Origin: Exclude, Weighting: 1/x, Axis trans: None

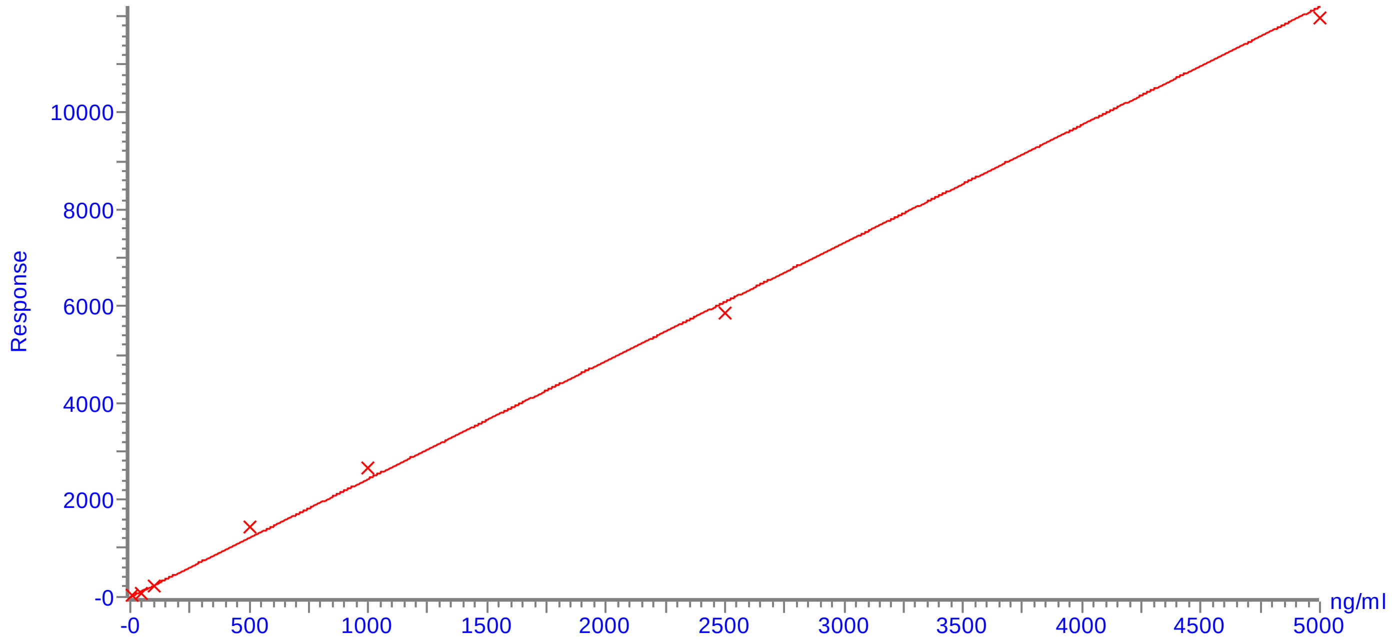

4-methyl-2-oxo-pentanoic acid					
	Std Conc (ng/mL)	Response	Conc.	%Dev	S/N
1	10	11.661132	10.06	0.6	1106.915
2	50	81.006854	38.47	-23.1	2285.011
3	100	229.312603	99.22	-0.8	3319.217
4	500	1437.893426	594.29	18.9	2101.931
5	1000	2666.831949	1097.70	9.8	4439.94
6	2500	5870.959997	2410.22	-3.6	5385.672
7	5000	11973.56943	4910.04	-1.8	6600.557

Compound name: Fumaric acid
Correlation coefficient: r = 0.999419, r^2 = 0.998838
Calibration curve: 13.2882 * x + -139.601
Response type: Internal Std (Ref 9), Area * (IS Conc. / IS Area)
Curve type: Linear, Origin: Exclude, Weighting: 1/x, Axis trans: None

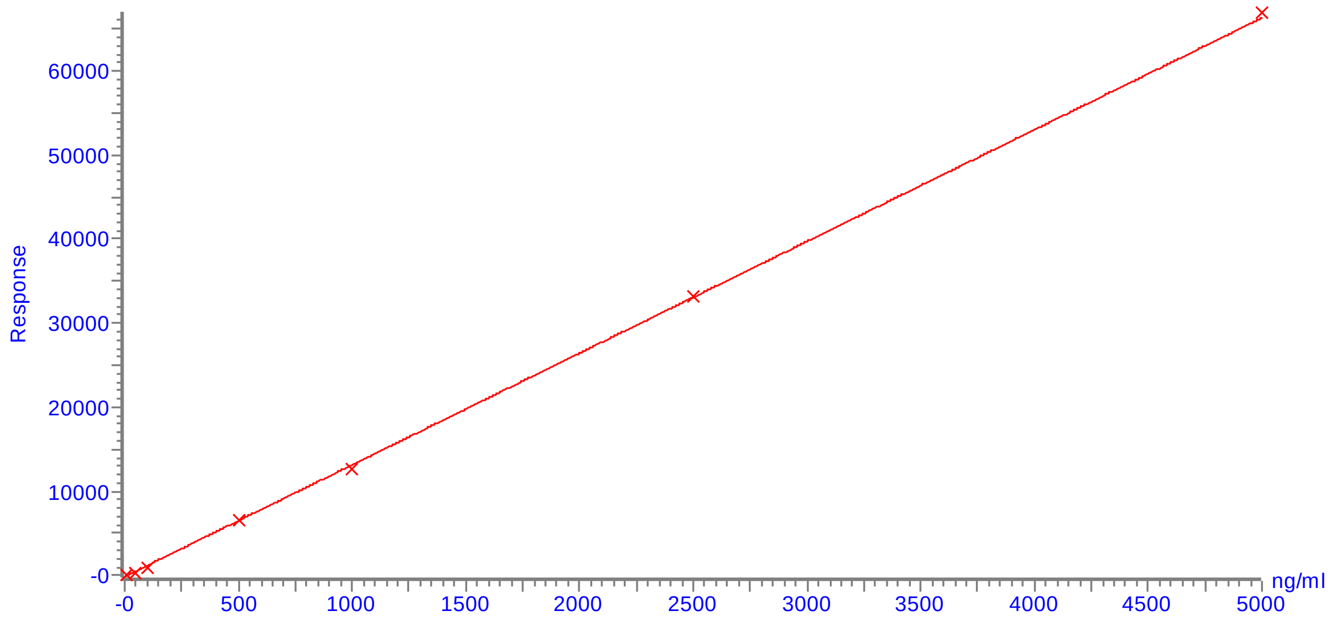

Fumaric acid					
	Std Conc (ng/mL)	Response	Conc.	%Dev	S/N
1	10	51.952646	14.42	44.2	
2	50	349.524635	36.81	-26.4	
3	100	977.47907	84.07	-15.9	
4	500	6553.966778	503.72	0.7	
5	1000	12601.05991	958.80	-4.1	
6	2500	33276.54234	2514.73	0.6	
7	5000	66932.05177	5047.46	0.9	

Compound name: Maleic acid
Correlation coefficient: r = 0.997995, r^2 = 0.995994
Calibration curve: 30.0176 * x + -334.853
Response type: Internal Std (Ref 9), Area * (IS Conc. / IS Area)
Curve type: Linear, Origin: Exclude, Weighting: 1/x, Axis trans: None

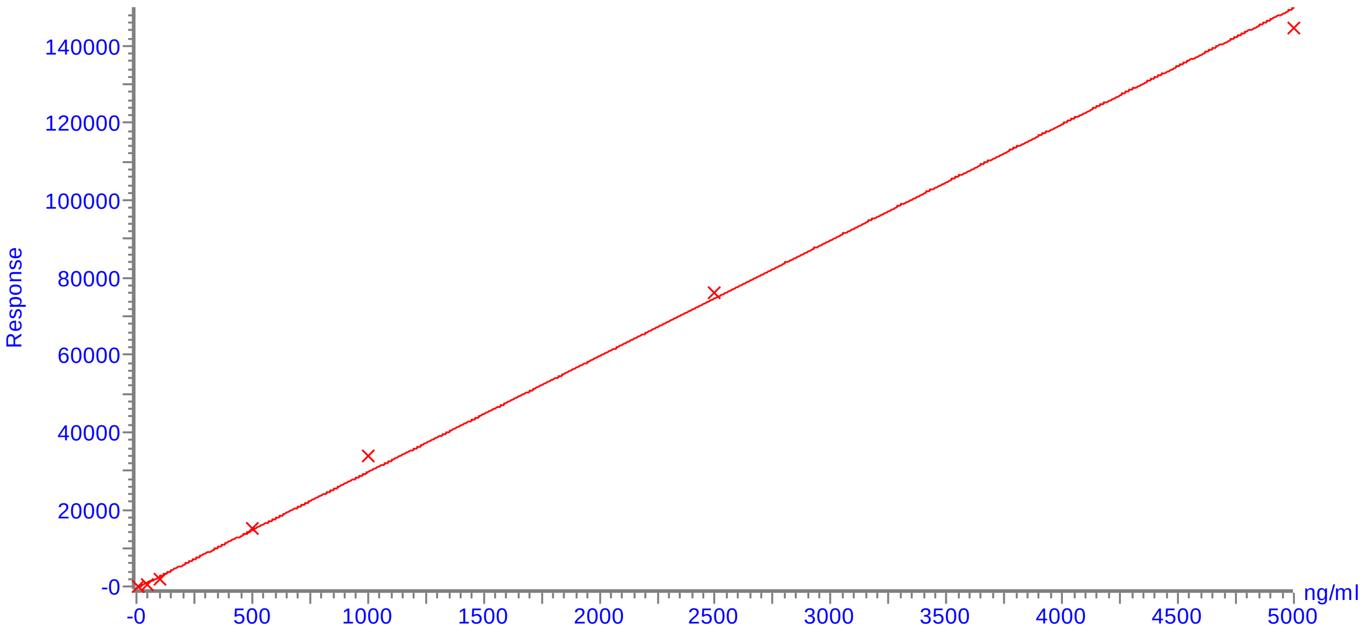

Maleic acid					
	Std Conc (ng/mL)	Response	Conc.	%Dev	S/N
1	10	64.602489	13.31	33.1	7415.358
2	50	724.591949	35.29	-29.4	10348.629
3	100	2064.030258	79.92	-20.1	12235.669
4	500	15358.13931	522.79	4.6	16886.924
5	1000	33723.81409	1134.62	13.5	24748.384
6	2500	76089.10544	2545.97	1.8	34871.894
7	5000	144592.6513	4828.09	-3.4	5480.32

Compound name: Valine
Correlation coefficient: r = 0.998404, r^2 = 0.996812
Calibration curve: 0.00438182 * x + 0.00765242
Response type: Internal Std (Ref 2), Area * (IS Conc. / IS Area)
Curve type: Linear, Origin: Exclude, Weighting: 1/x, Axis trans: None

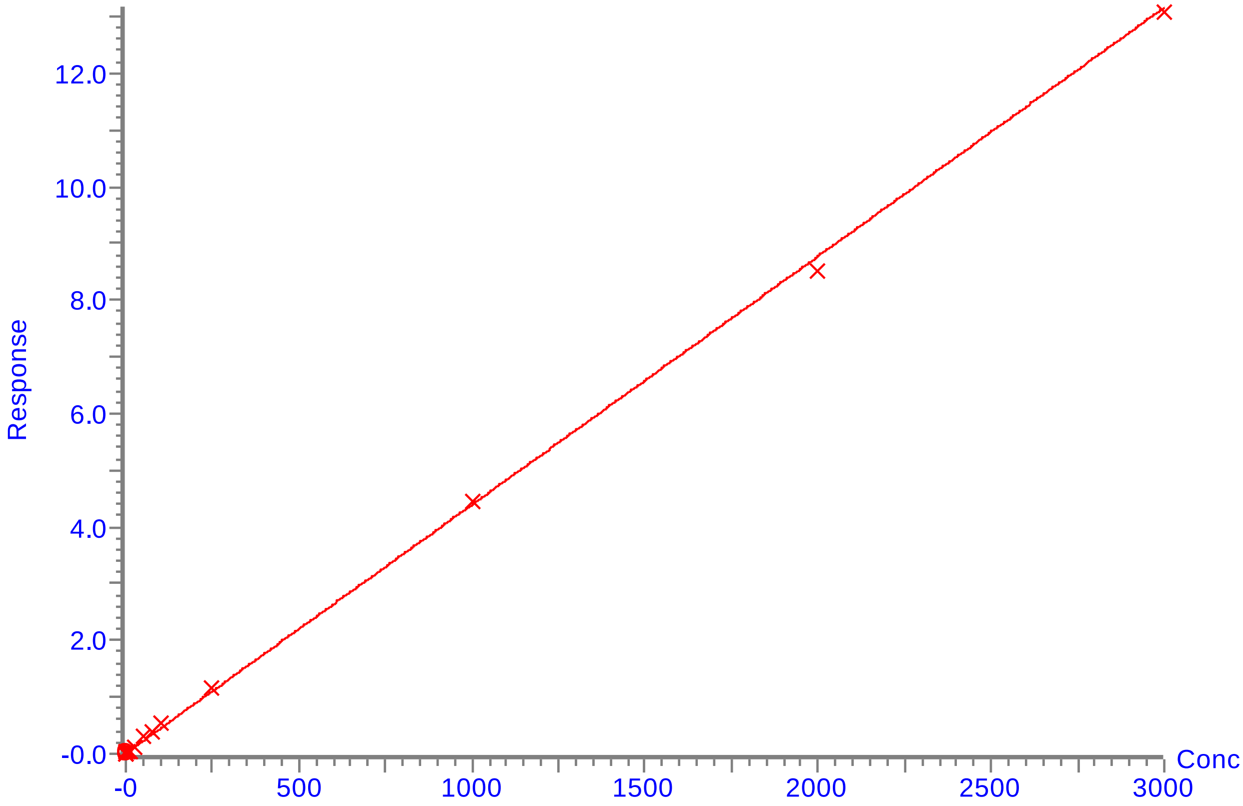

Valine					
	Std Conc (ng/mL)	Response	Conc.	%Dev	S/N
1	5	0.097	5.9	17.7	342.11
2	7.5	0.098	7	-7.1	391.381
3	10	0.1	9.6	-4.2	401.245
4	25	0.113	23.2	-7	324.818
5	50	0.14	54.7	9.4	549.304
6	75	0.164	81.4	8.5	490.369
7	100	0.178	96.8	-3.2	415.159
8	250	0.277	209.7	-16.1	398.88
9	1000	0.986	1011.4	1.1	942.359
10	2000	1.868	2009	0.5	793.978
11	3000	2.756	3013.9	0.5	1046.82

Compound name: Succinic acid
Correlation coefficient: r = 0.999769, r^2 = 0.999538
Calibration curve: 0.000779028 * x + 0.0205476
Response type: Internal Std (Ref 2), Area * (IS Conc. / IS Area)
Curve type: Linear, Origin: Exclude, Weighting: 1/x, Axis trans: None

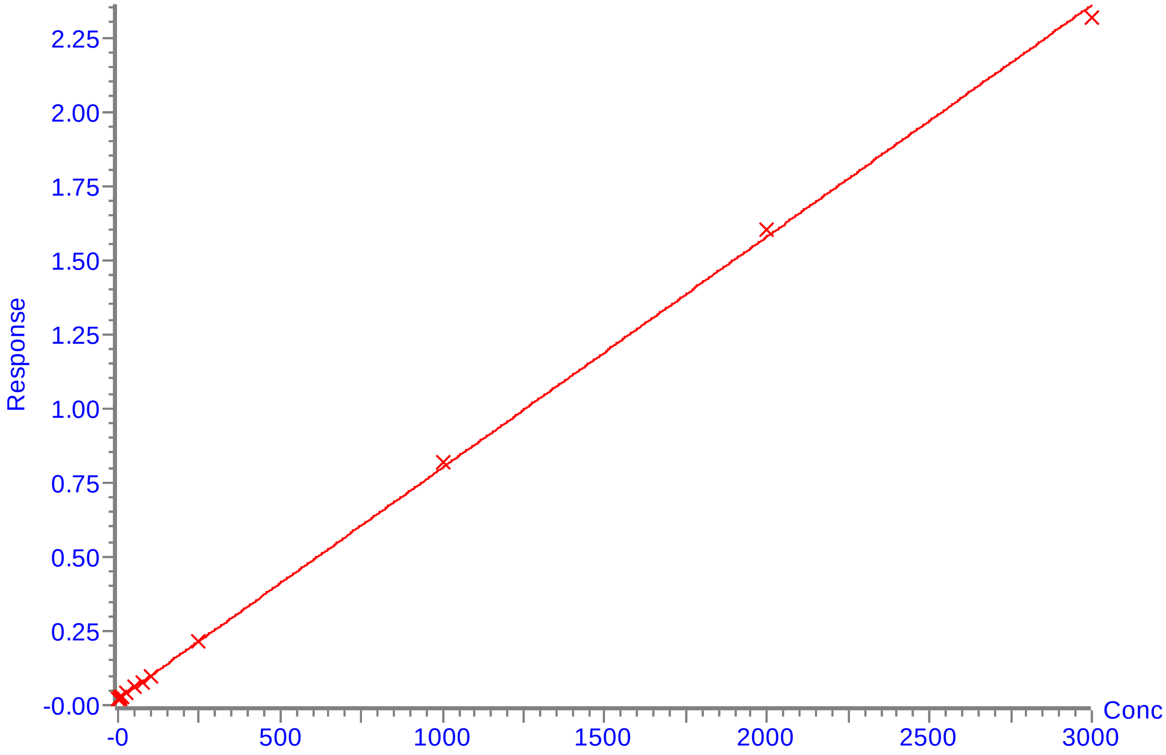

Succinic acid					
	Std Conc (ng/mL)	Response	Conc.	%Dev	S/N
1	1	0.021	1	-0.8	105.902
2	2.5	0.022	2.2	-13	24.337
3	5	0.024	4.4	-12.8	59.842
4	7.5	0.027	7.8	3.4	31.761
5	10	0.03	11.6	15.6	40.139
6	25	0.041	26.7	6.9	42.057
7	50	0.063	54.2	8.4	110.749
8	75	0.075	70.2	-6.4	203.725
9	100	0.097	97.7	-2.3	116.438
10	250	0.213	247.6	-1	260.912
11	1000	0.817	1022.3	2.2	554.023
12	2000	1.602	2029.8	1.5	319.115
13	3000	2.319	2950.7	-1.6	402.72

Compound name: Ureidopropionic acid
Correlation coefficient: r = 0.999824, r^2 = 0.999648
Calibration curve: 0.000281963 * x + -0.000330475
Response type: Internal Std (Ref 2), Area * (IS Conc. / IS Area)
Curve type: Linear, Origin: Exclude, Weighting: 1/x, Axis trans: None

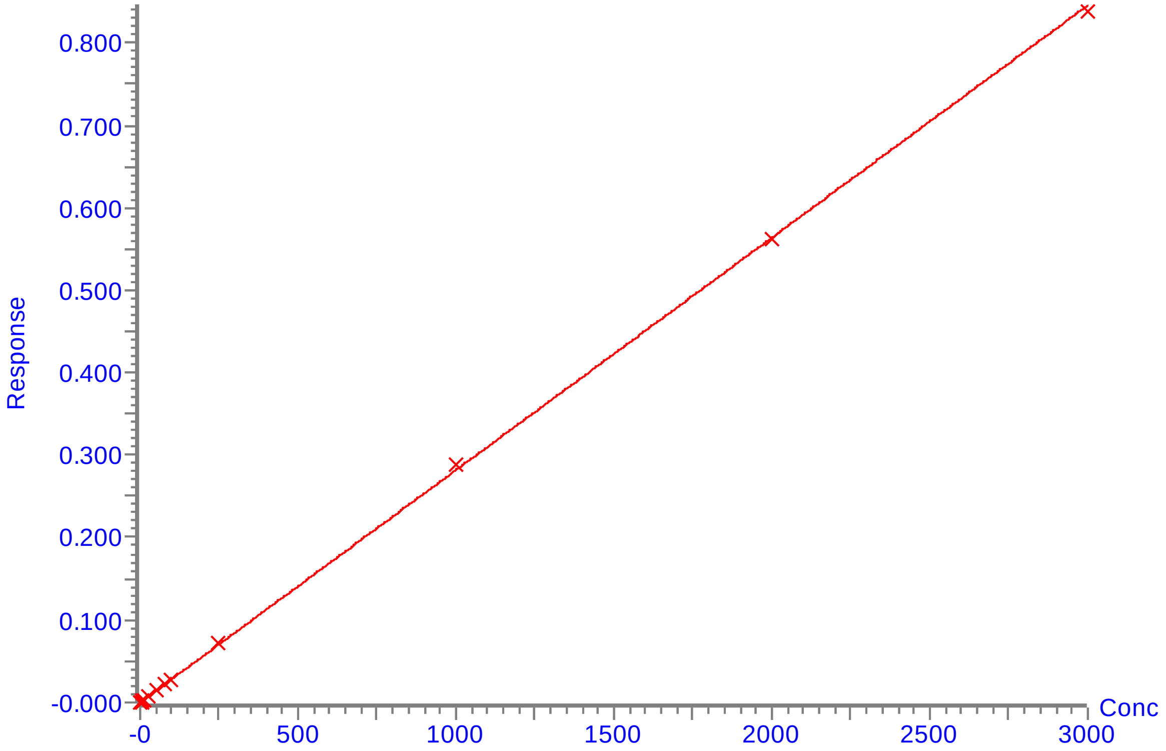

Ureidopropionic acid					
	Std Conc (ng/mL)	Response	Conc.	%Dev	S/N
1	5	0.001	4.1	-18.1	51.052
2	7.5	0.002	7	-6.3	67.003
3	10	0.002	9.6	-4	34.592
4	25	0.007	24.3	-2.6	113.066
5	50	0.015	54.7	9.3	357.734
6	75	0.022	78.1	4.1	481.159
7	100	0.026	94.9	-5.1	385.451
8	250	0.072	256.8	2.7	1390.741
9	1000	0.288	1024.4	2.4	3804.177
10	2000	0.561	1990.8	-0.5	3057.401
11	3000	0.839	2977.9	-0.7	2793.254

Compound name: Oxaloacetic acid
Correlation coefficient: r = 0.999627, r^2 = 0.999254
Calibration curve: 5.70966e-005 * x + 0.0012498
Response type: Internal Std (Ref 2), Area * (IS Conc. / IS Area)
Curve type: Linear, Origin: Exclude, Weighting: 1/x, Axis trans: None

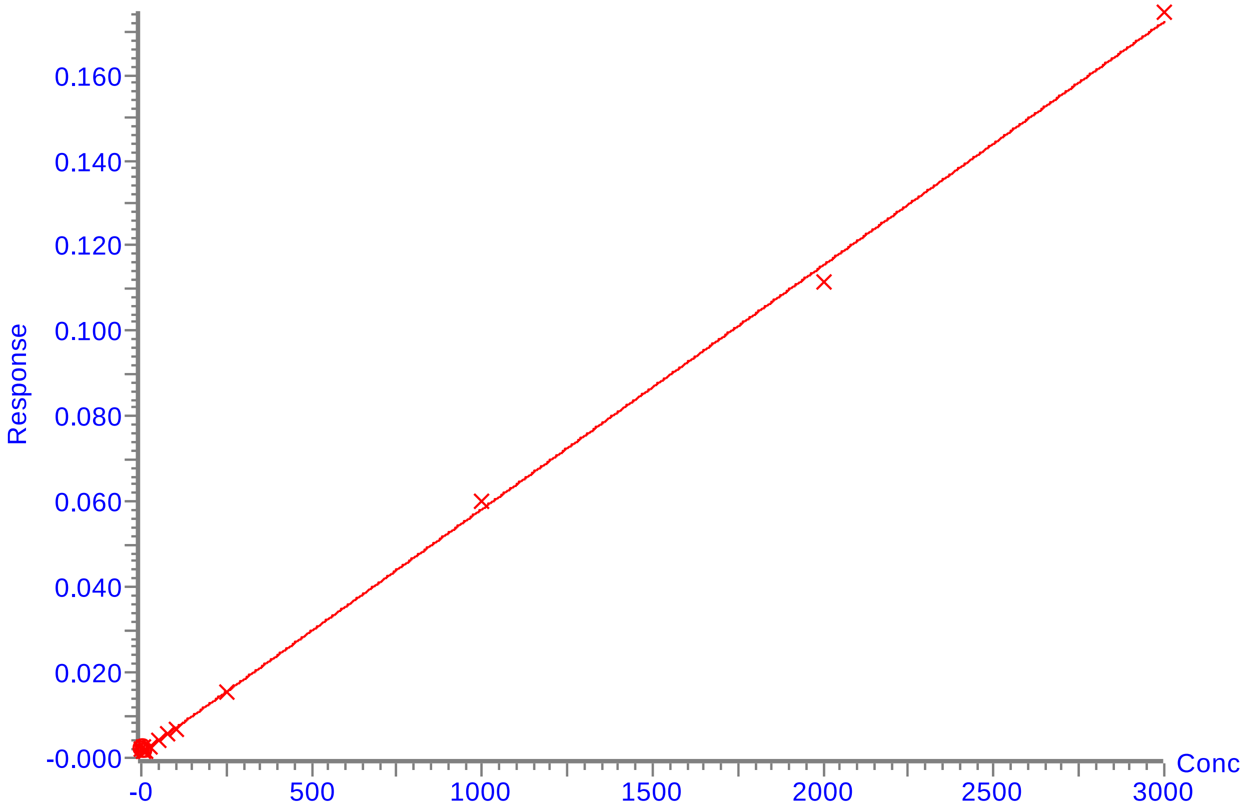

Oxaloacetic acid					
	Std Conc (ng/mL)	Response	Conc.	%Dev	S/N
1	50	0.004	47.5	-4.9	13.59
2	75	0.006	76.1	1.5	15.99
3	100	0.007	98.4	-1.6	15.839
4	250	0.015	248.3	-0.7	39.467
5	1000	0.06	1035.1	3.5	181.353
6	2000	0.111	1930.9	-3.5	231.574
7	3000	0.175	3037.6	1.3	157.504

Compound name: Aspartic acid
Correlation coefficient: r = 0.999638, r^2 = 0.999276
Calibration curve: 0.0029226 * x + 0.007553
Response type: Internal Std (Ref 2), Area * (IS Conc. / IS Area)
Curve type: Linear, Origin: Exclude, Weighting: 1/x, Axis trans: None

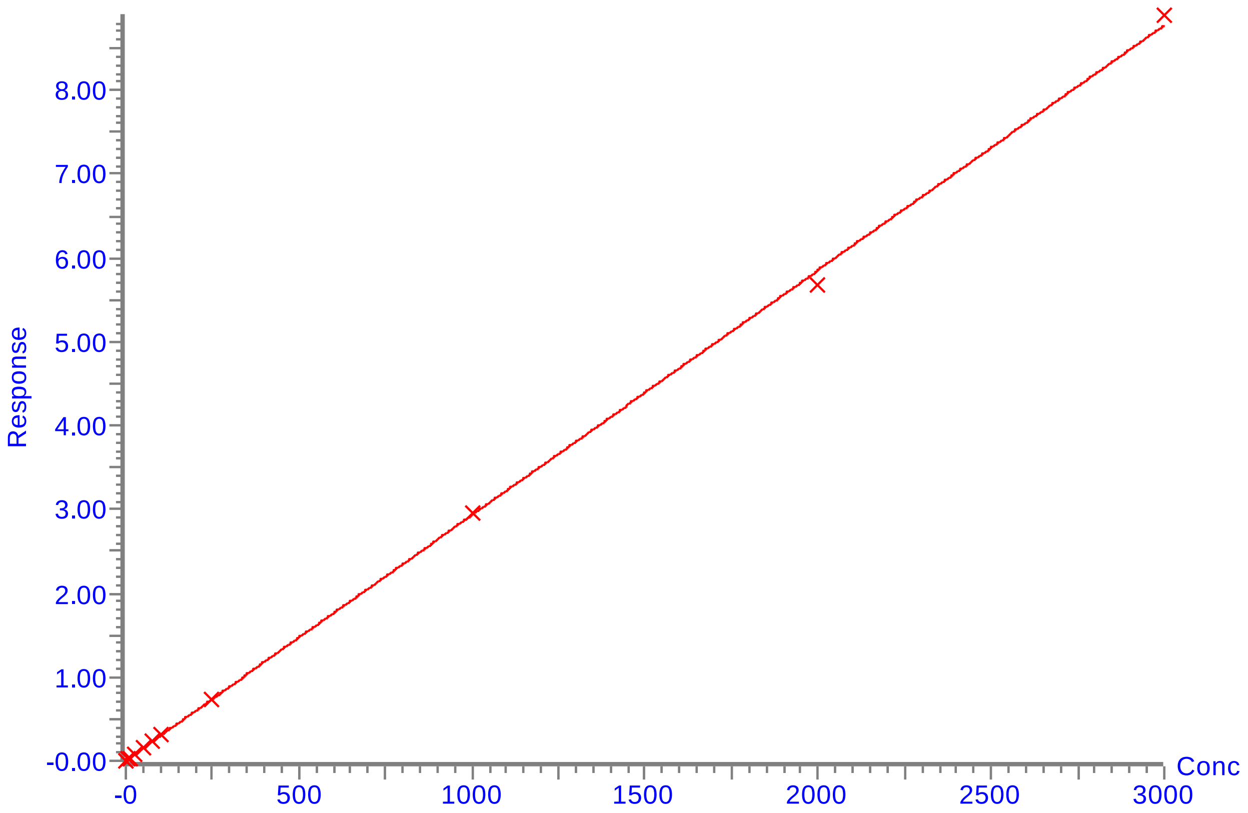

Aspartic acid					
	Std Conc (ng/mL)	Response	Conc.	%Dev	S/N
1	1	0.011	1.2	21.9	58.739
2	5	0.024	5.5	9.6	315.545
3	7.5	0.031	7.9	4.8	209.108
4	10	0.039	10.8	7.7	287.046
5	25	0.089	27.8	11.3	312.138
6	50	0.151	49.1	-1.7	678.733
7	75	0.237	78.6	4.8	1267.253
8	100	0.325	108.5	8.5	655.415
9	250	0.731	247.4	-1	1350.748
10	1000	2.943	1004.4	0.4	3680.649
11	2000	5.686	1943.1	-2.8	3146.528
12	3000	8.895	3040.8	1.4	4304.487

Compound name: Malic acid
Correlation coefficient: r = 0.998995, r^2 = 0.997992
Calibration curve: 0.00041664 * x + -2.08854e-005
Response type: Internal Std (Ref 2), Area * (IS Conc. / IS Area)
Curve type: Linear, Origin: Exclude, Weighting: 1/x, Axis trans: None

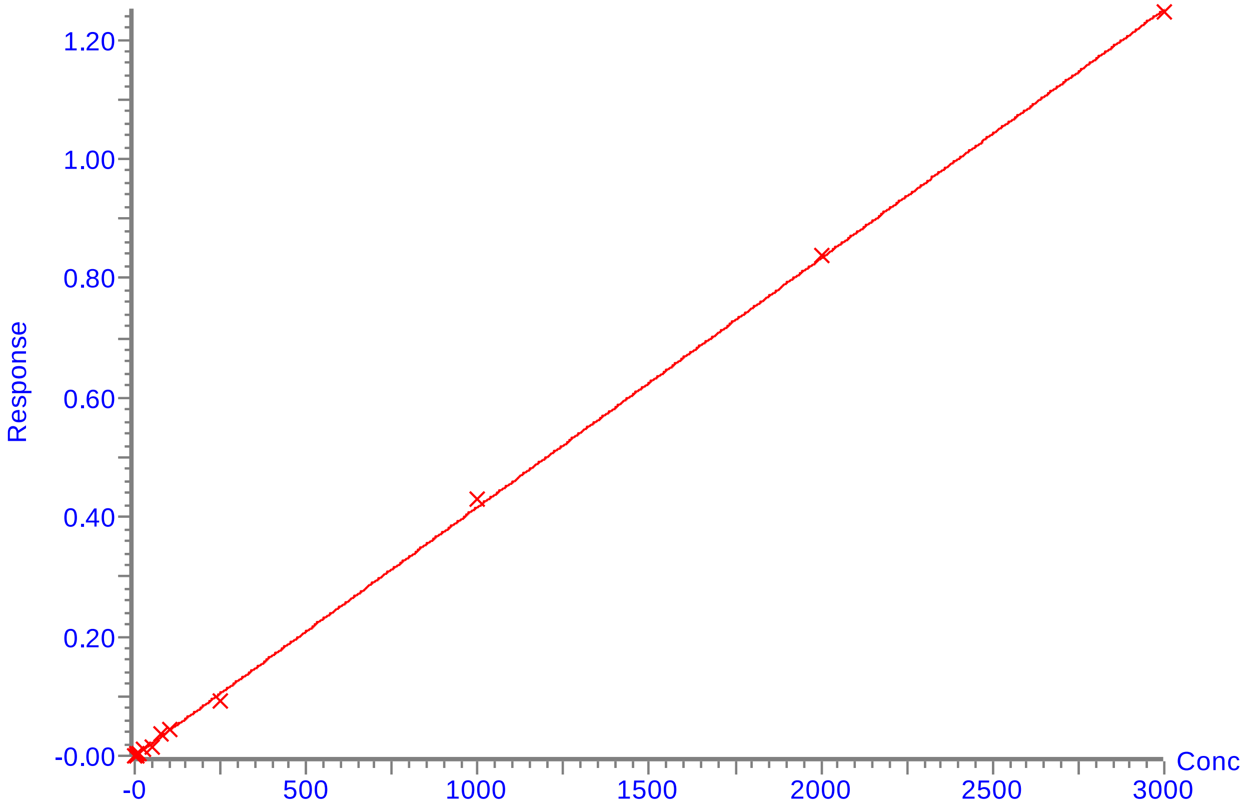

Malic acid					
	Std Conc (ng/mL)	Response	Conc.	%Dev	S/N
1	1	0.001	1.3	33.7	8.419
2	2.5	0.001	2.7	6.3	5.725
3	5	0.002	4.4	-12.9	23.439
4	7.5	0.003	7	-6.2	40.698
5	10	0.005	11	10.3	25.411
6	25	0.01	23.8	-4.8	43.998
7	75	0.035	84.8	13.1	139.548
8	100	0.043	103.6	3.6	111.923
9	250	0.091	219.5	-12.2	420.434
10	1000	0.432	1036.4	3.6	1768.697
11	2000	0.837	2009.4	0.5	3162.574
12	3000	1.245	2989.3	-0.4	1826.383

Compound name: Homocysteine
Correlation coefficient: r = 0.999688, r^2 = 0.999376
Calibration curve: 0.0004124 * x + 0.00427699
Response type: Internal Std (Ref 2), Area * (IS Conc. / IS Area)
Curve type: Linear, Origin: Exclude, Weighting: 1/x, Axis trans: None

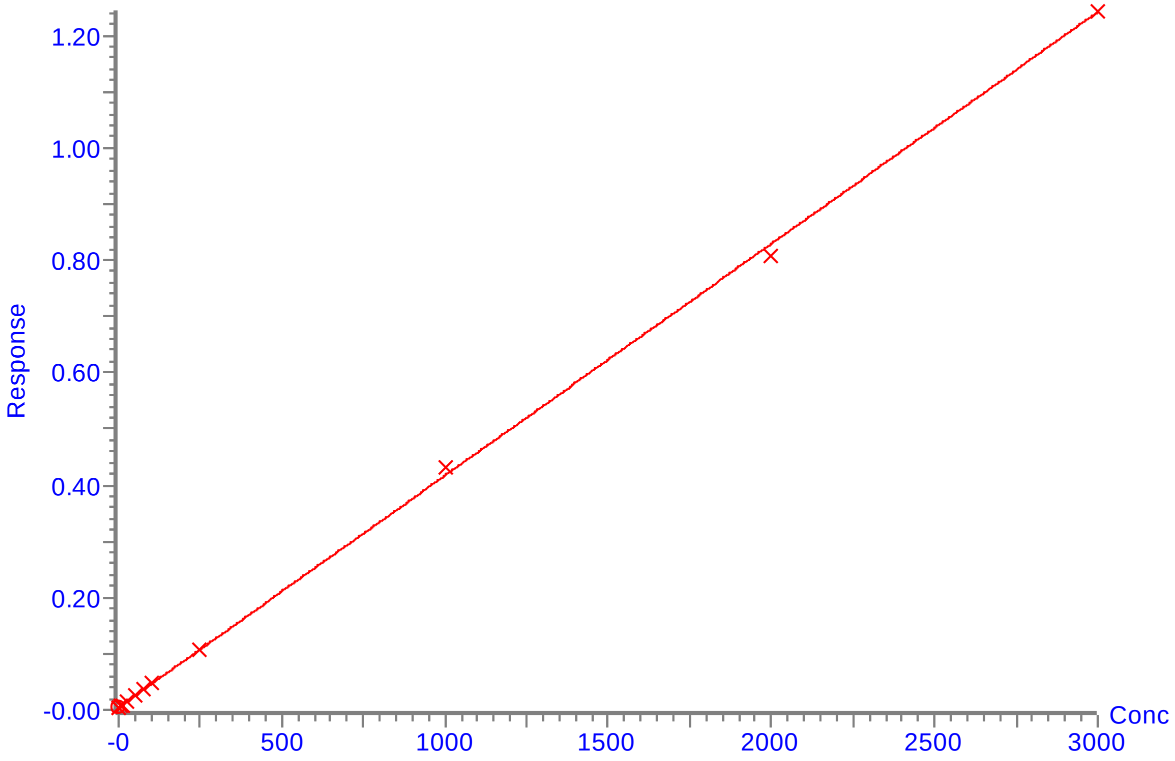

Homocysteine					
	Std Conc (ng/mL)	Response	Conc.	%Dev	S/N
1	1	0.005	0.7	-33.7	146.988
2	5	0.006	4.8	-3.3	403.054
3	7.5	0.007	6.6	-11.6	188.437
4	10	0.009	11.5	15.5	220.005
5	25	0.016	28.9	15.4	345.835
6	50	0.027	55.1	10.1	1224.045
7	75	0.036	77.6	3.4	1478.894
8	100	0.047	102.5	2.5	299.599
9	250	0.108	252.3	0.9	2376.68
10	1000	0.43	1031.4	3.1	4210.038
11	2000	0.808	1949.2	-2.5	5012.416
12	3000	1.243	3002.9	0.1	3845.646

Compound name: aKGA_152
Correlation coefficient: r = 0.998891, r^2 = 0.997783
Calibration curve: 0.000814376 * x + 0.0087788
Response type: Internal Std (Ref 2), Area * (IS Conc. / IS Area)
Curve type: Linear, Origin: Exclude, Weighting: 1/x, Axis trans: None

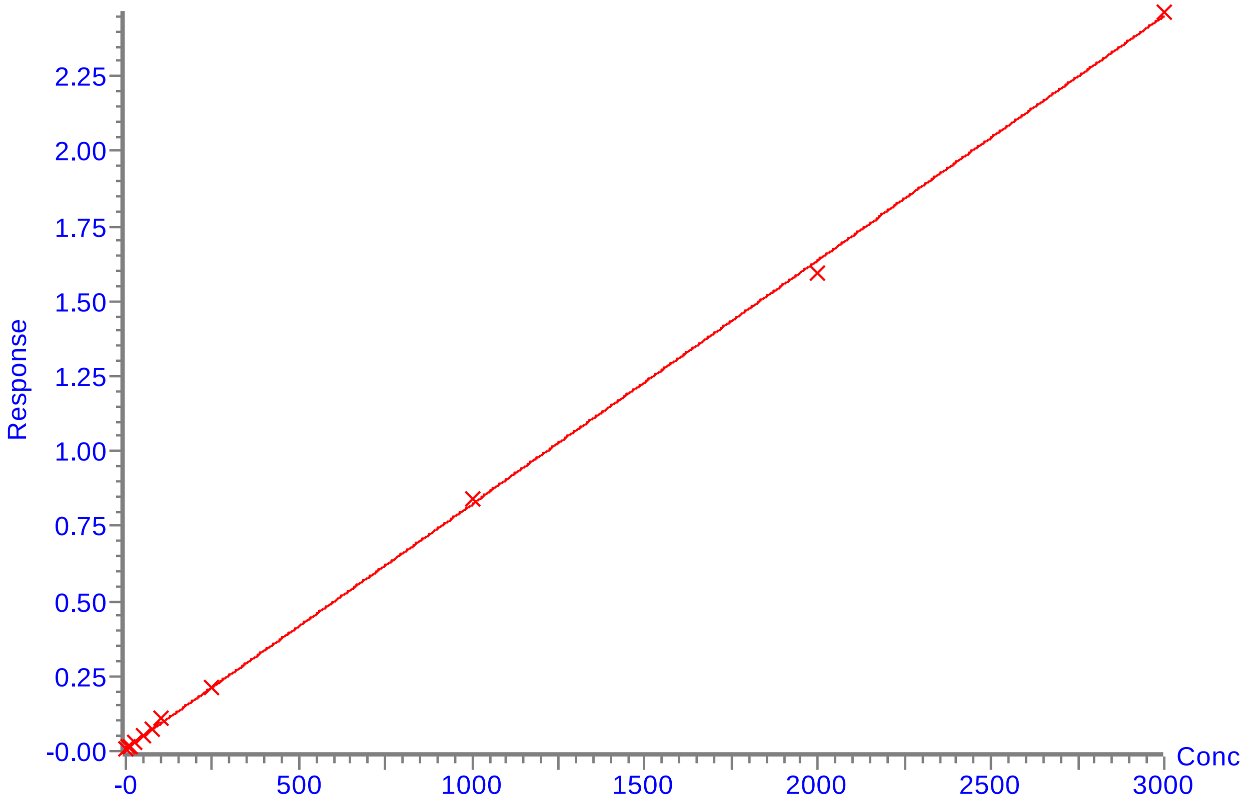

Alpha-keto-glutaric acid					
	Std Conc (ng/mL)	Response	Conc.	%Dev	S/N
1	5	0.013	5.4	7.7	124.262
2	7.5	0.015	7.1	-4.9	102.656
3	10	0.016	9.2	-7.8	71.14
4	25	0.029	25.1	0.2	102.525
5	50	0.054	55.1	10.1	336.385
6	75	0.07	75.1	0.1	287.371
7	100	0.108	122.4	22.4	444.491
8	250	0.213	250.2	0.1	843.036
9	1000	0.838	1018.2	1.8	1458.003
10	2000	1.591	1942.9	-2.9	1510.92
11	3000	2.464	3014.3	0.5	1086.407

Compound name: Glutamine_152
Correlation coefficient: r = 0.999356, r^2 = 0.998712
Calibration curve: 0.00100782 * x + 0.00943305
Response type: Internal Std (Ref 2), Area * (IS Conc. / IS Area)
Curve type: Linear, Origin: Exclude, Weighting: 1/x, Axis trans: None

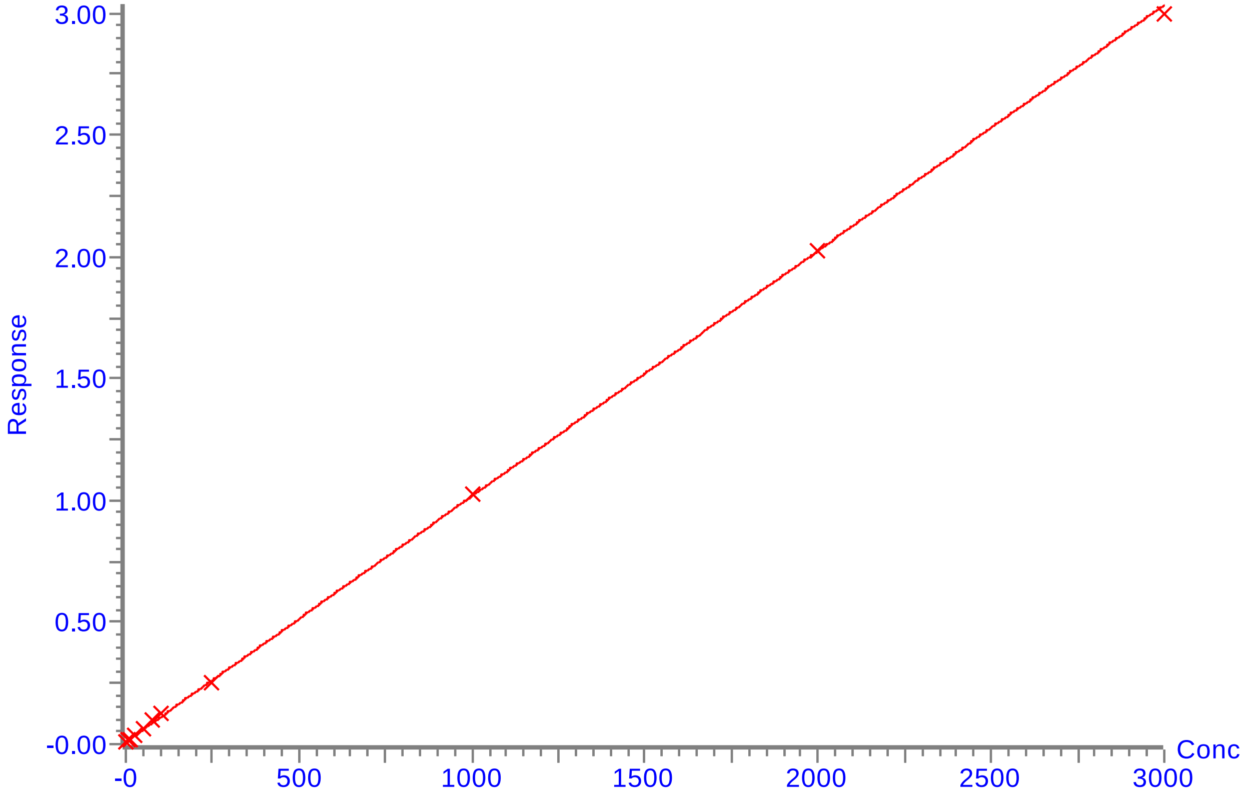

Glutamine					
	Std Conc (ng/mL)	Response	Conc.	%Dev	S/N
1	5	0.014	4.9	-2.8	139.098
2	7.5	0.017	7.2	-4	101.091
3	10	0.02	10.8	8	63.539
4	25	0.036	26.2	4.8	137.775
5	50	0.062	52.5	5	250.312
6	75	0.097	86.6	15.5	633.039
7	100	0.123	113	13	490.594
8	250	0.253	241.6	-3.4	746.382
9	1000	1.03	1012.4	1.2	1621.754
10	2000	2.029	2004	0.2	1932.759
11	3000	2.998	2965.4	-1.2	1474.488

Compound name: Methionine_152
Correlation coefficient: r = 0.999559, r^2 = 0.999119
Calibration curve: 4.62315e-005 * x + 0.00268953
Response type: Internal Std (Ref 2), Area * (IS Conc. / IS Area)
Curve type: Linear, Origin: Exclude, Weighting: 1/x, Axis trans: None

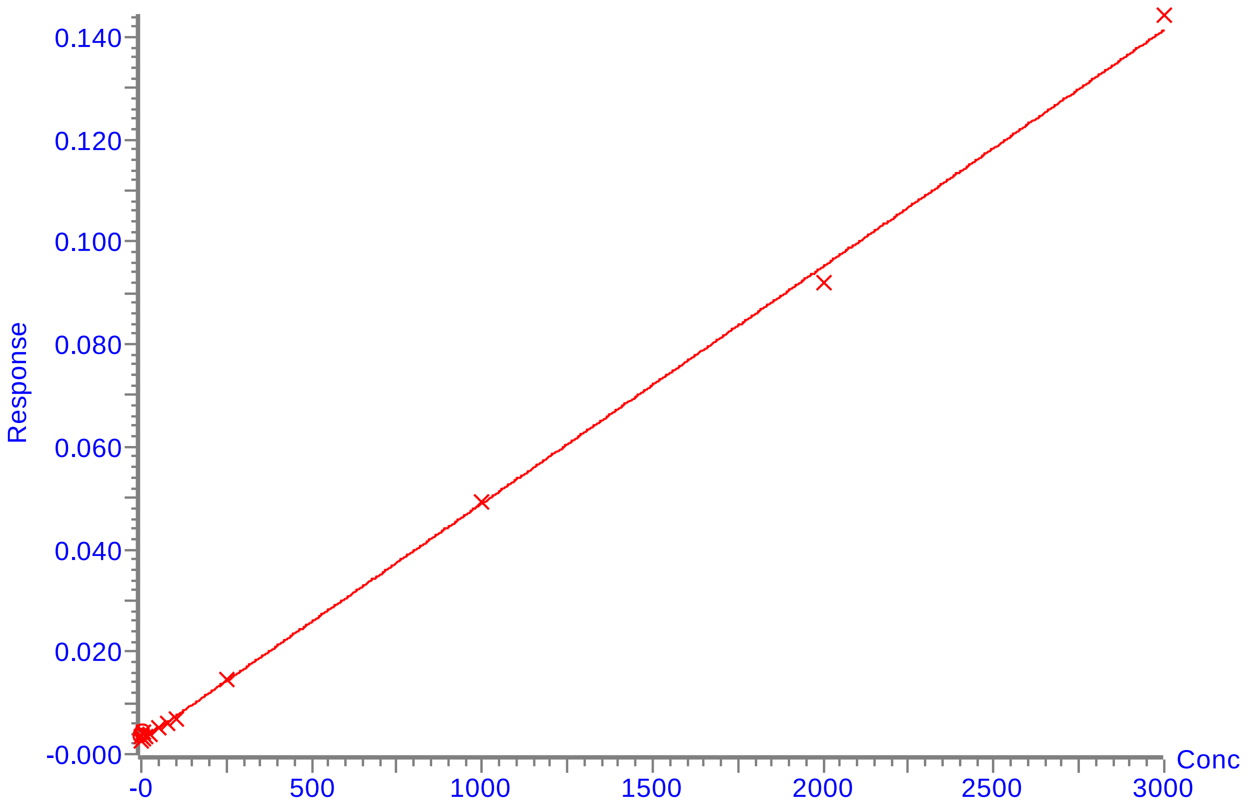

Methionine					
	Std Conc (ng/mL)	Response	Conc.	%Dev	S/N
1	7.5	0.003	7.8	4.4	34.616
2	10	0.003	11.9	19.3	41.675
3	25	0.004	27	8	61.381
4	50	0.005	50	-0.1	40.626
5	75	0.006	75.2	0.3	39.201
6	100	0.007	91.4	-8.6	65.268
7	250	0.015	258	3.2	50.736
8	1000	0.049	1006.5	0.7	198.578
9	2000	0.092	1928.3	-3.6	119.426
10	3000	0.144	3062	2.1	327.797

Compound name: Orotic acid
Correlation coefficient: r = 0.999248, r^2 = 0.998497
Calibration curve: 0.000893395 * x + 0.032125
Response type: Internal Std (Ref 2), Area * (IS Conc. / IS Area)
Curve type: Linear, Origin: Exclude, Weighting: 1/x, Axis trans: None

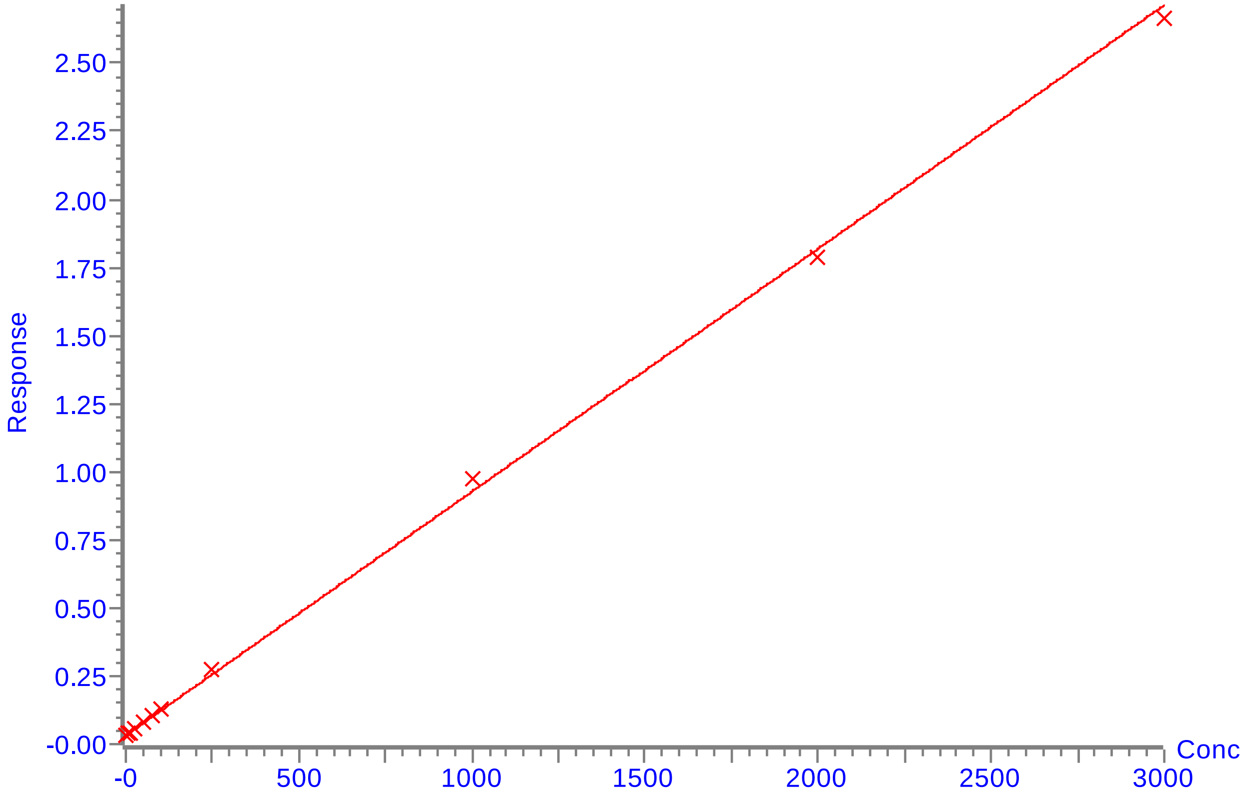

Orotic acid					
	Std Conc (ng/mL)	Response	Conc.	%Dev	S/N
1	1	0.033	1.1	7.4	130.403
2	5	0.037	5.8	16.5	163.758
3	7.5	0.04	8.5	12.7	171.48
4	10	0.043	12.6	25.7	109.247
5	25	0.055	26.1	4.3	95.644
6	50	0.08	54.1	8.3	325.824
7	75	0.103	79.5	6	267.907
8	100	0.125	104.3	4.3	236.986
9	250	0.271	266.8	6.7	353.954
10	1000	0.975	1055.1	5.5	770.713
11	2000	1.79	1967.1	-1.6	602.851
12	3000	2.663	2944.8	-1.8	829.658

Compound name: Methyl adipic acid
Correlation coefficient: r = 0.999696, r^2 = 0.999392
Calibration curve: 0.00899636 * x + 0.246154
Response type: Internal Std (Ref 2), Area * (IS Conc. / IS Area)
Curve type: Linear, Origin: Exclude, Weighting: 1/x, Axis trans: None

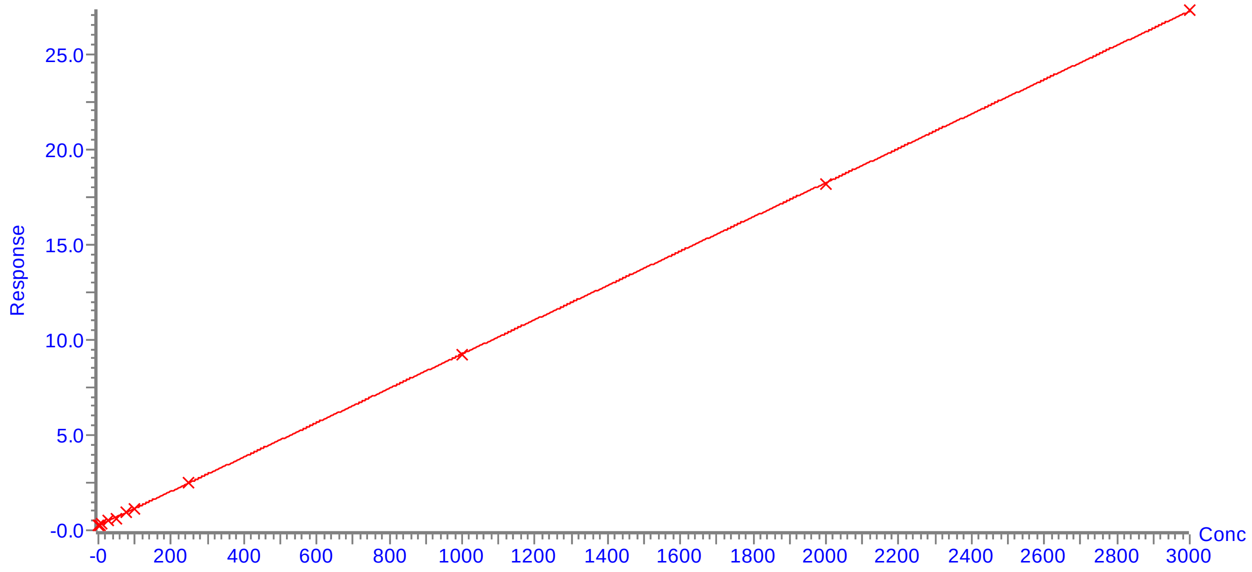

3-Methyl adipic acid					
	Std Conc (ng/mL)	Response	Conc.	%Dev	S/N
1	1	0.254	0.9	-7.5	2950.632
2	5	0.298	5.8	15.5	4485.552
3	7.5	0.332	9.6	27.8	3264.924
4	10	0.369	13.6	36	1839.697
5	25	0.486	26.6	6.5	2328.618
6	50	0.644	44.2	-11.6	2955.949
7	75	0.915	74.4	-0.8	3138.629
8	100	1.151	100.6	0.6	3744.729
9	250	2.488	249.2	-0.3	7354.977
10	1000	9.242	1000	0	11577.701
11	2000	18.189	1994.4	-0.3	14753.171
12	3000	27.287	3005.8	0.2	17835.707

Compound name: N-Acetyl glutamine
Correlation coefficient: r = 0.999387, r^2 = 0.998775
Calibration curve: 0.000168394 * x + 0.000398661
Response type: Internal Std (Ref 2), Area * (IS Conc. / IS Area)
Curve type: Linear, Origin: Exclude, Weighting: 1/x, Axis trans: None

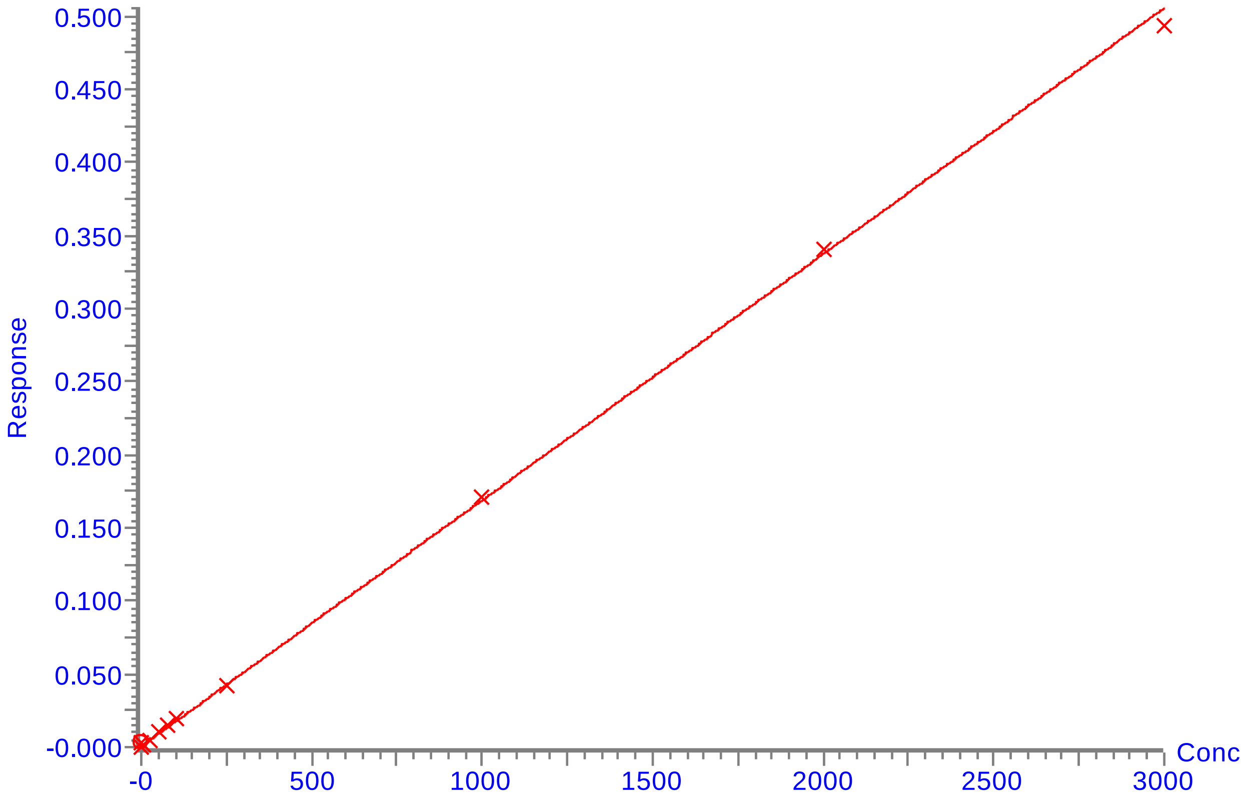

N-acetyl glutamine					
	Std Conc (ng/mL)	Response	Conc.	%Dev	S/N
1	5	0.001	5	-0.6	11.369
2	7.5	0.002	9.5	26.7	8.383
3	25	0.006	32	28.1	10.855
4	50	0.011	59.2	18.5	24.198
5	75	0.015	85.5	14	30.721
6	100	0.019	108.4	8.4	35.322
7	250	0.042	248	-0.8	66.517
8	1000	0.171	1014.7	1.5	218.669
9	2000	0.341	2023.3	1.2	154.767
10	3000	0.494	2929.3	-2.4	138.155

Compound name: Ascorbic acid
Correlation coefficient: r = 0.999671, r^2 = 0.999342
Calibration curve: 0.000394784 * x + 0.19762
Response type: Internal Std (Ref 2), Area * (IS Conc. / IS Area)
Curve type: Linear, Origin: Exclude, Weighting: 1/x, Axis trans: None

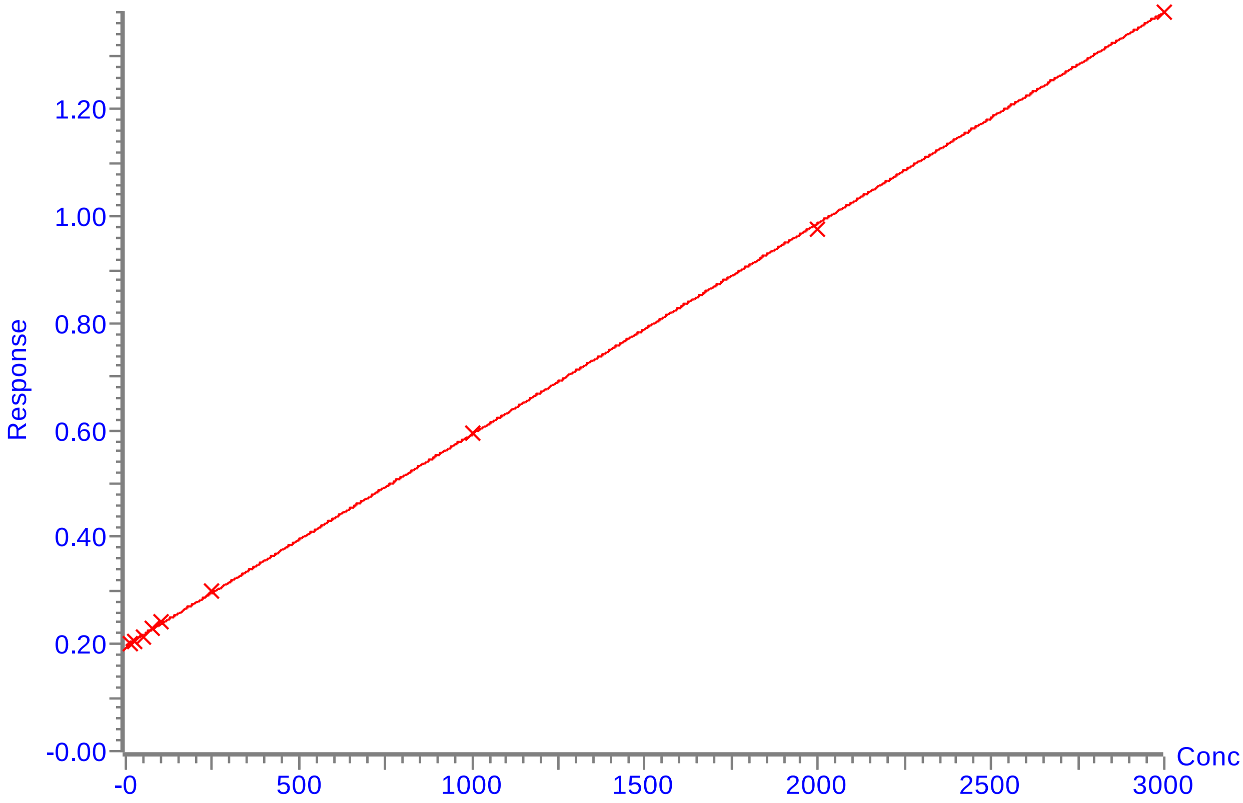

Ascorbic acid					
	Std Conc (ng/mL)	Response	Conc.	%Dev	S/N
1	10	0.202	9.9	-1.4	1484.186
2	25	0.206	22.4	-10.4	1535.161
3	50	0.215	44.7	-10.6	1476.083
4	75	0.229	78.6	4.8	1674.704
5	100	0.243	115.8	15.8	1810.019
6	250	0.298	255.5	2.2	1753.098
7	1000	0.596	1008.6	0.9	3433.815
8	2000	0.978	1975.9	-1.2	3453.523
9	3000	1.381	2998.6	0	4531.584

Compound name: 3-Nitrotyrosine
Correlation coefficient: r = 0.999897, r^2 = 0.999794
Calibration curve: 0.000510407 * x + 0.106181
Response type: Internal Std (Ref 2), Area * (IS Conc. / IS Area)
Curve type: Linear, Origin: Exclude, Weighting: 1/x, Axis trans: None

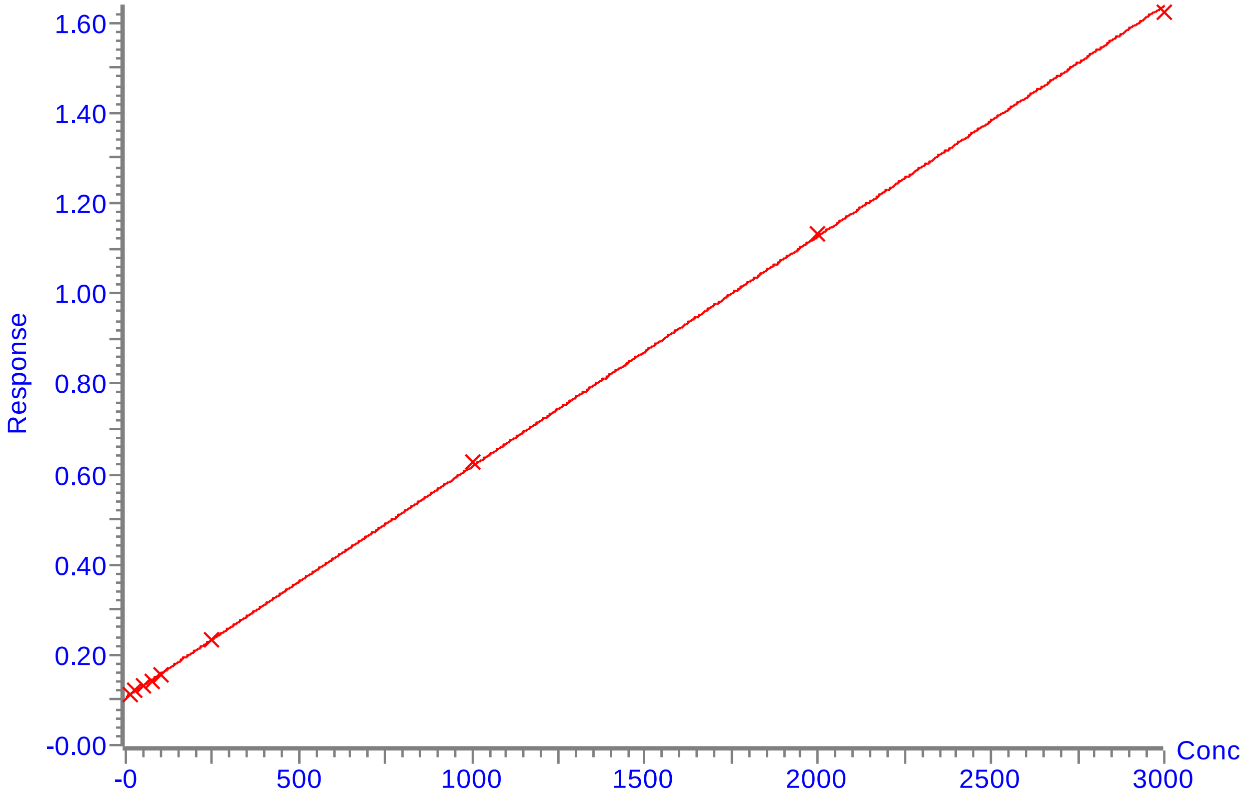

3-Nitrotyrosine					
	Std Conc (ng/mL)	Response	Conc.	%Dev	S/N
1	10	0.111	10.2	2.1	279.203
2	25	0.119	25.6	2.4	193.576
3	50	0.131	48.4	-3.2	274.018
4	75	0.142	71	-5.4	235.879
5	100	0.158	101	1	172.438
6	250	0.236	253.4	1.4	316.201
7	1000	0.628	1023.3	2.3	241.99
8	2000	1.131	2008.5	0.4	550.553
9	3000	1.621	2968.5	-1	282.509

Compound name: MMA_180
Correlation coefficient: r = 0.999480, r^2 = 0.998960
Calibration curve: 0.000254513 * x + 0.0458174
Response type: Internal Std (Ref 2), Area * (IS Conc. / IS Area)
Curve type: Linear, Origin: Exclude, Weighting: 1/x, Axis trans: None

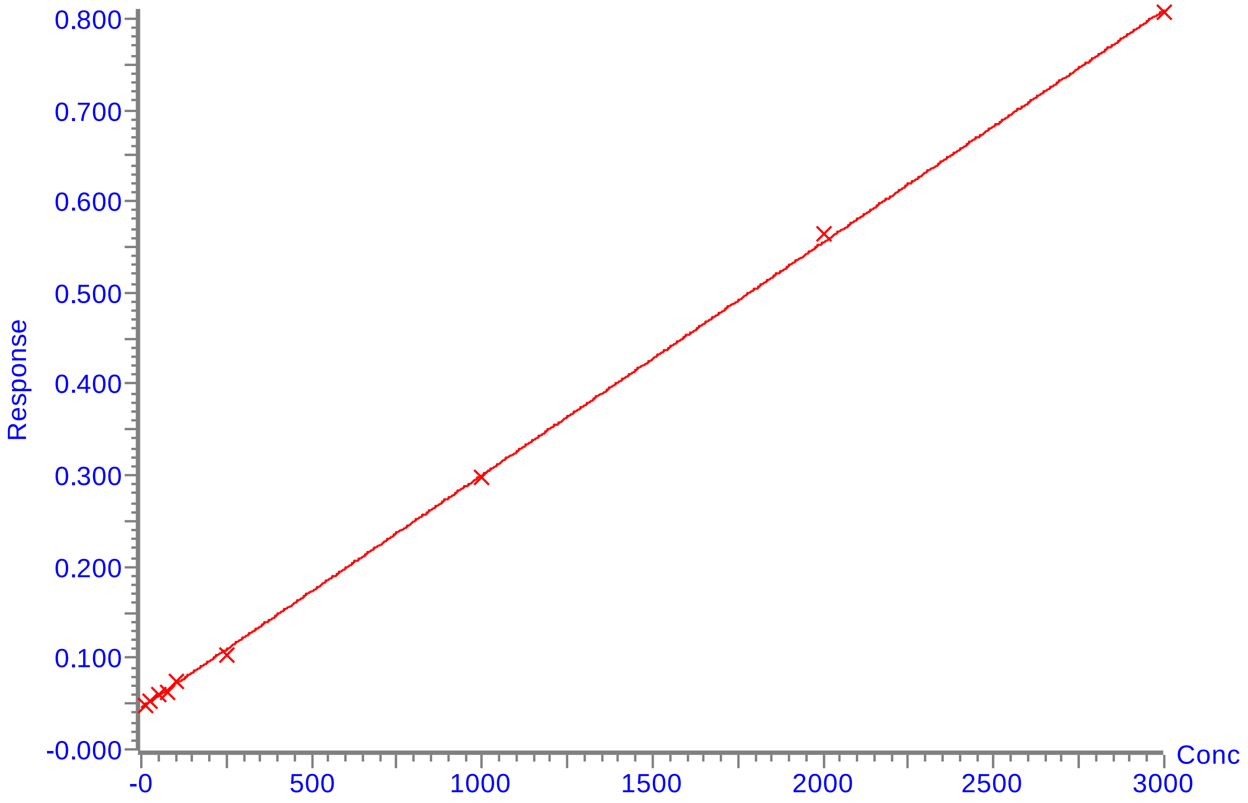

Methyl malonic acid					
	Std Conc (ng/mL)	Response	Conc.	%Dev	S/N
1	10	0.049	10.8	7.7	72.804
2	25	0.052	24.3	-2.9	124.989
3	50	0.06	56	12.1	71.765
4	75	0.062	62.2	-17	30.926
5	100	0.073	108	8	95.107
6	250	0.104	228.5	-8.6	184.353
7	1000	0.298	991.8	-0.8	312.34
8	2000	0.565	2039	2	235.456
9	3000	0.807	2989.3	-0.4	157.563

Compound name: Docosatetraenoic acid
Correlation coefficient: r = 0.999542, r^2 = 0.999083
Calibration curve: 0.000364727 * x + 0.0186498
Response type: Internal Std (Ref 2), Area * (IS Conc. / IS Area)
Curve type: Linear, Origin: Exclude, Weighting: 1/x, Axis trans: None

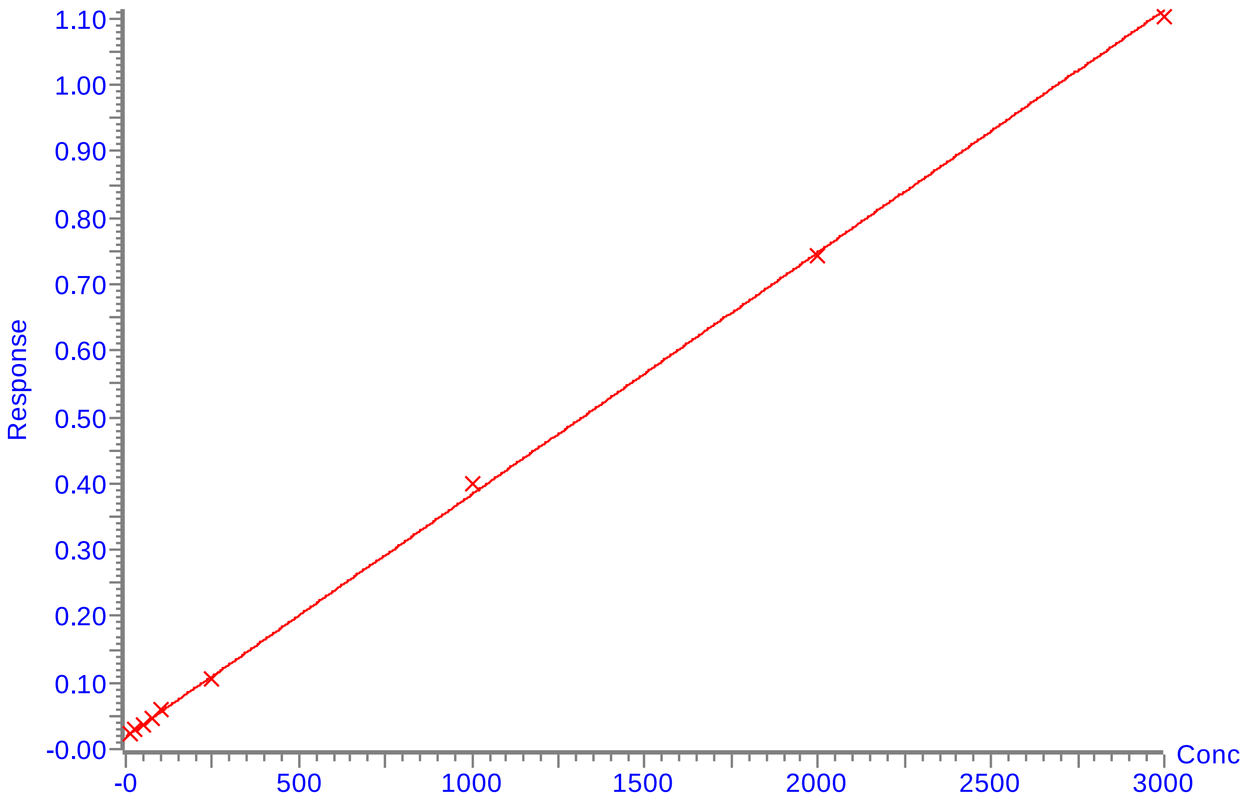

Docosatetraenoic acid					
	Std Conc (ng/mL)	Response	Conc.	%Dev	S/N
1	10	0.022	8.8	-11.9	127.435
2	25	0.029	28.6	14.6	184.379
3	50	0.035	45.3	-9.5	355.49
4	75	0.045	71.4	-4.9	496.697
5	100	0.06	112.1	12.1	530.818
6	250	0.107	241.7	-3.3	878.552
7	1000	0.4	1046.3	4.6	1543.57
8	2000	0.743	1985.7	-0.7	1072.081
9	3000	1.102	2970.2	-1	1561.537

Compound name: Palmitic acid_179
Correlation coefficient: r = 0.999425, r^2 = 0.998851
Calibration curve: 0.00486967 * x + 0.34432
Response type: Internal Std (Ref 2), Area * (IS Conc. / IS Area)
Curve type: Linear, Origin: Exclude, Weighting: 1/x, Axis trans: None

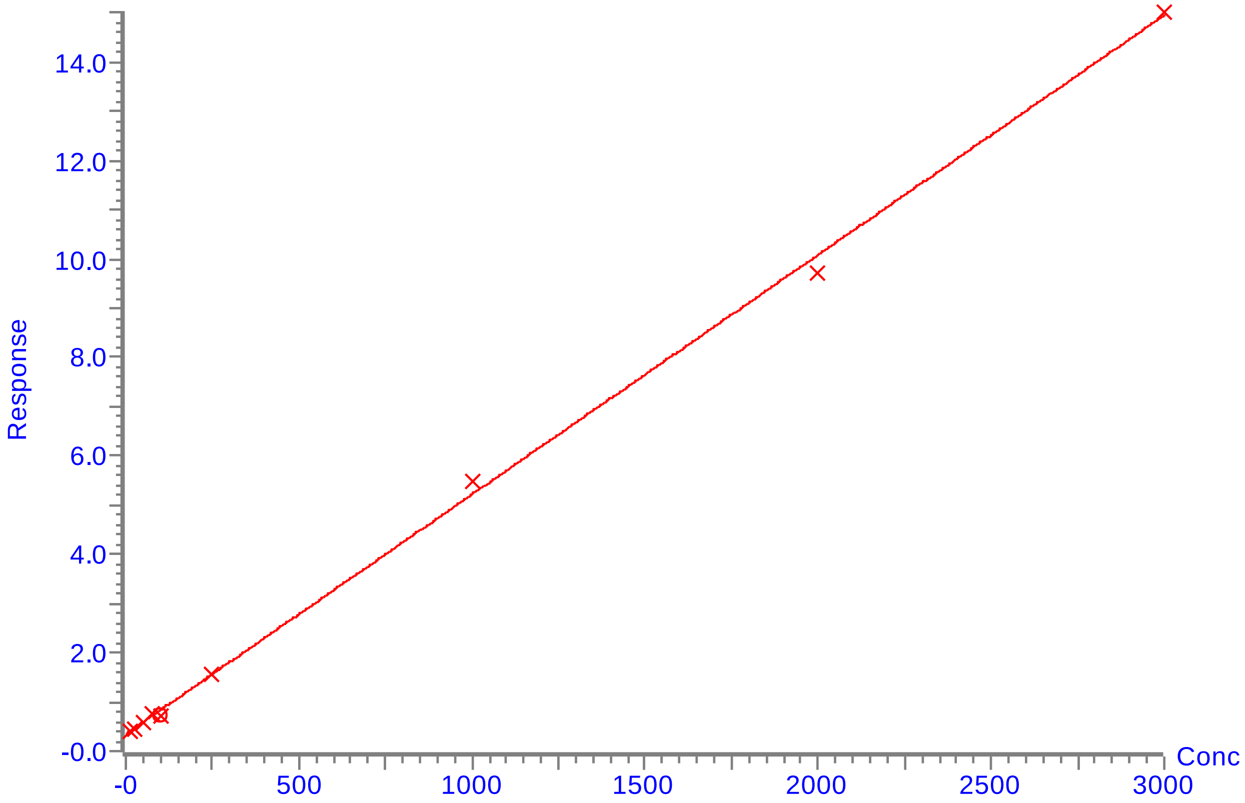

Palmitic acid					
	Std Conc (ng/mL)	Response	Conc.	%Dev	S/N
1	10	0.391	9.7	-3.2	1113.135
2	25	0.462	24.1	-3.4	3946.547
3	50	0.575	47.4	-5.1	6028.302
4	75	0.741	81.5	8.6	3645.023
5	100	0.73	79.2	-20.8	923.577
6	250	1.57	251.8	0.7	9527.806
7	1000	5.495	1057.7	5.8	15306.201
8	2000	9.717	1924.7	-3.8	12760.493
9	3000	15.017	3013.1	0.4	6947.416

Compound name: Glycine
Correlation coefficient: r = 0.999805, r^2 = 0.999611
Calibration curve: 0.00218867 * x + -0.000390896
Response type: Internal Std (Ref 2), Area * (IS Conc. / IS Area)
Curve type: Linear, Origin: Exclude, Weighting: 1/x, Axis trans: None

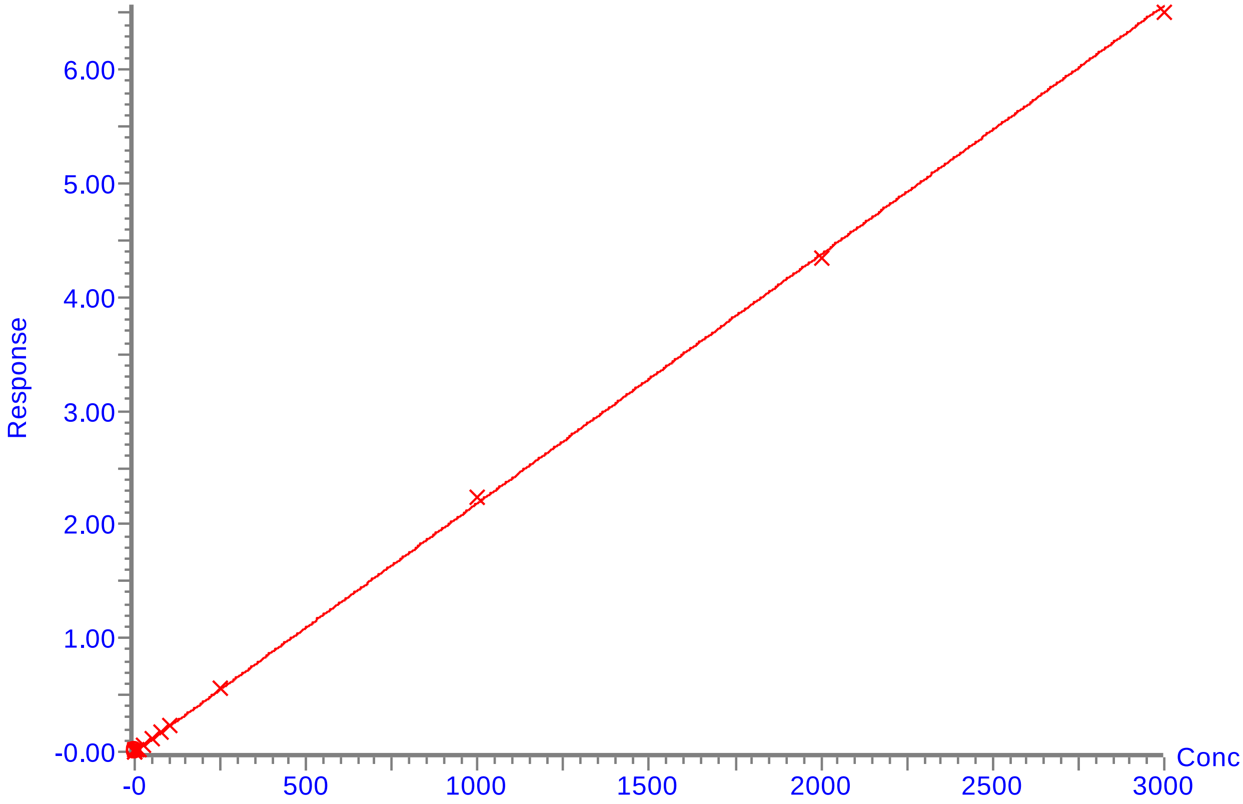

Glycine					
	Std Conc (ng/mL)	Response	Conc.	%Dev	S/N
1	1	0.002	0.9	-9.9	2.592
2	5	0.011	5	0.8	50.838
3	7.5	0.014	6.7	-11	41.95
4	10	0.023	10.5	4.6	71.915
5	25	0.049	22.7	-9.3	88.262
6	50	0.124	57	14.1	144.869
7	75	0.169	77.6	3.5	109.329
8	100	0.23	105.4	5.4	505.257
9	250	0.552	252.4	1	381.049
10	1000	2.237	1022.1	2.2	768.538
11	2000	4.353	1988.9	-0.6	577.137
12	3000	6.509	2974.2	-0.9	816.833

Compound name: Sarcosine
Correlation coefficient: r = 0.999686, r^2 = 0.999372
Calibration curve: 0.00104959 * x + 0.110184
Response type: Internal Std (Ref 2), Area * (IS Conc. / IS Area)
Curve type: Linear, Origin: Exclude, Weighting: 1/x, Axis trans: None

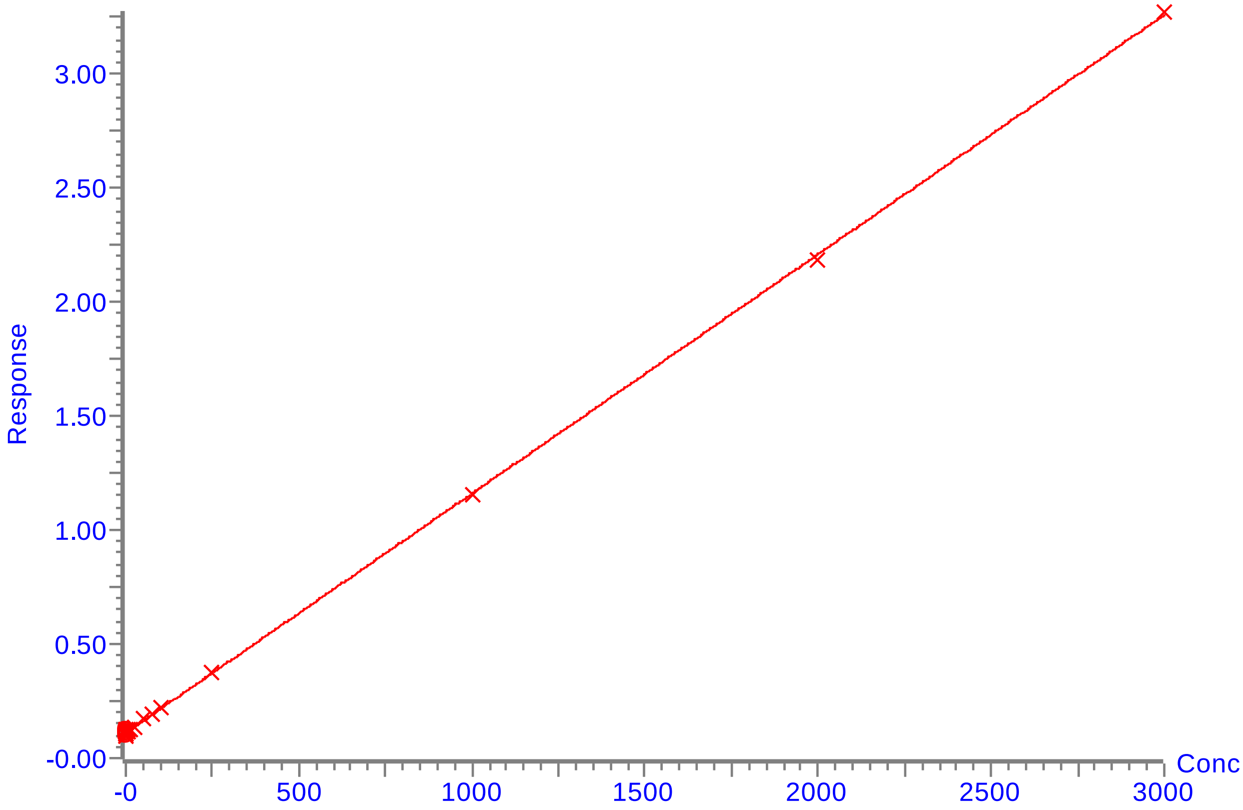

Sarcosine					
	Std Conc (ng/mL)	Response	Conc.	%Dev	S/N
1	2.5	0.113	2.7	9.2	214.917
2	5	0.115	5	0.2	401.774
3	10	0.122	11.4	13.5	106.901
4	25	0.137	25.3	1.4	473.746
5	50	0.172	58.8	17.6	372.6
6	75	0.19	75.7	0.9	419.328
7	100	0.22	104.7	4.7	270.337
8	250	0.375	252.6	1	788.64
9	1000	1.15	990.8	-0.9	1201.022
10	2000	2.185	1976.4	-1.2	2941.195
11	3000	3.272	3012.2	0.4	2572.065

Compound name: Lactic acid
Correlation coefficient: r = 0.999628, r^2 = 0.999256
Calibration curve: 0.00345725 * x + 0.00238623
Response type: Internal Std (Ref 2), Area * (IS Conc. / IS Area)
Curve type: Linear, Origin: Exclude, Weighting: 1/x, Axis trans: None

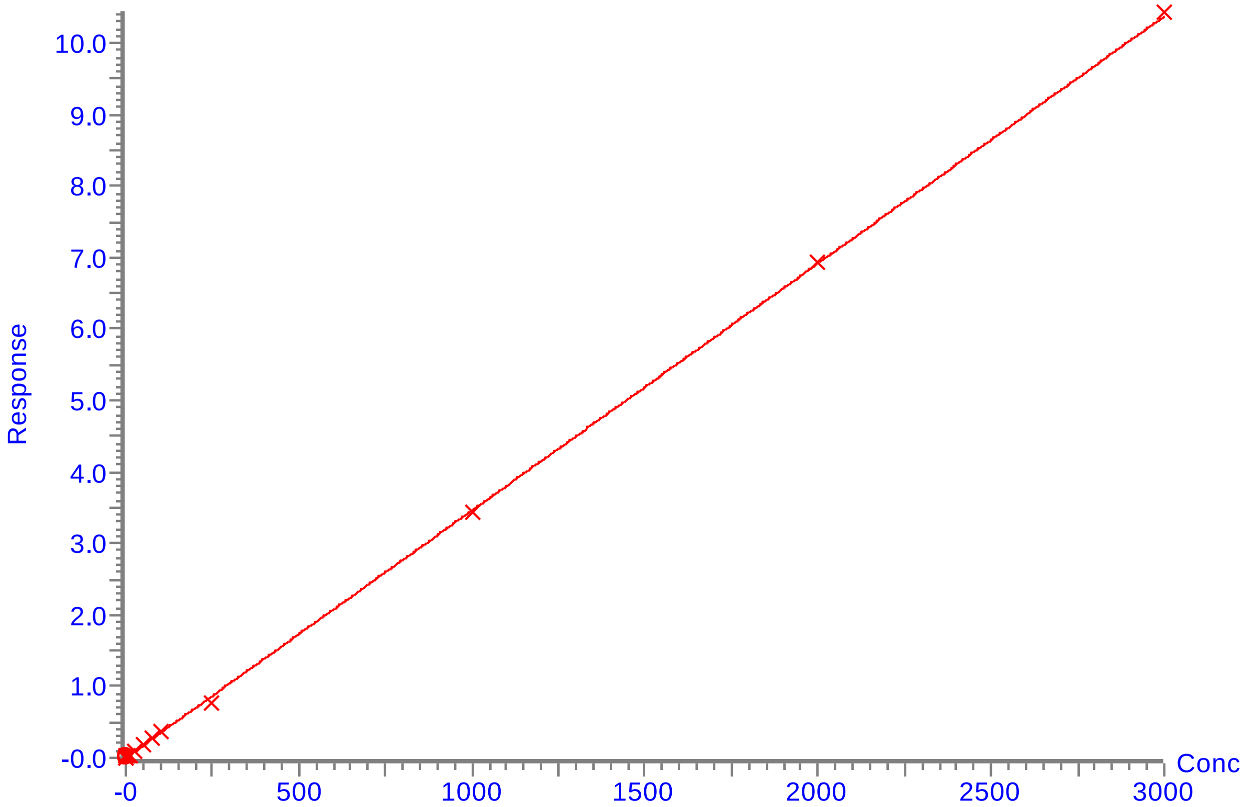

Lactic acid					
	Std Conc (ng/mL)	Response	Conc.	%Dev	S/N
1	0.1	0.003	0.1	-30.4	4.955
2	0.5	0.004	0.5	-6.8	16.851
3	0.75	0.005	0.7	-9.6	18.828
4	1	0.006	1	-3.3	9.017
5	5	0.02	5.2	4.2	130.329
6	10	0.041	11.2	12.2	249.28
7	50	0.189	54	8.1	837.488
8	100	0.356	102.4	2.4	1204.368
9	250	0.769	221.9	-11.3	1872.788
10	1000	3.434	992.6	-0.7	4005.933
11	2000	6.921	2001.3	0.1	3333.84
12	3000	10.431	3016.6	0.6	2976.899

Compound name: Oxalic acid
Correlation coefficient: r = 0.998438, r^2 = 0.996878
Calibration curve: 0.000671286 * x + 0.0197405
Response type: Internal Std (Ref 2), Area * (IS Conc. / IS Area)
Curve type: Linear, Origin: Exclude, Weighting: 1/x, Axis trans: None

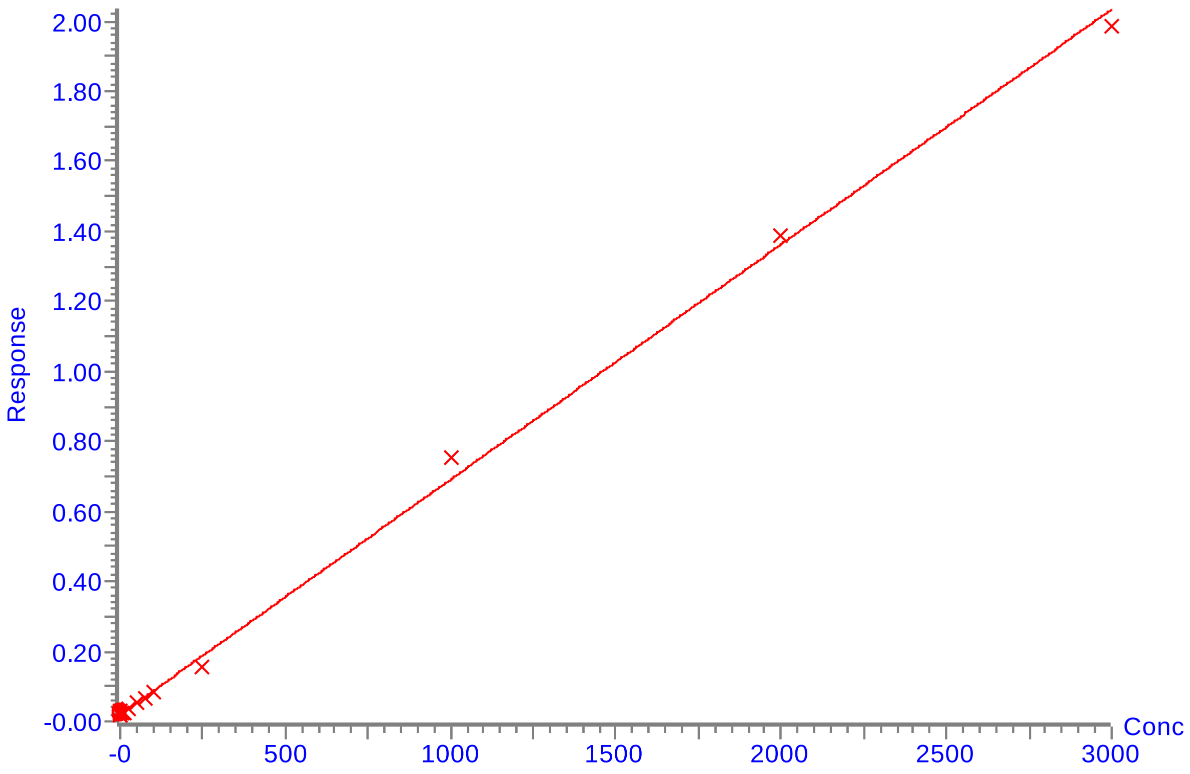

Oxalic acid					
	Std Conc (ng/mL)	Response	Conc.	%Dev	S/N
1	1	0.02	1	-2.5	47.26
2	2.5	0.022	2.7	8.5	17.042
3	5	0.024	5.7	14.9	39.351
4	7.5	0.025	7.5	-0.5	21.895
5	10	0.027	10.5	5	45.693
6	25	0.036	24.6	-1.7	35.305
7	50	0.053	50.2	0.5	42.794
8	100	0.085	96.8	-3.2	62.893
9	250	0.156	203.5	-18.6	119.053
10	1000	0.752	1090.5	9.1	855.561
11	2000	1.386	2034.7	1.7	558.196
12	3000	1.988	2931.4	-2.3	501.966

Compound name: Gamma-amino butyric acid
Correlation coefficient: r = 0.998691, r^2 = 0.997383
Calibration curve: 0.00544558 * x + 0.00430084
Response type: Internal Std (Ref 2), Area * (IS Conc. / IS Area)
Curve type: Linear, Origin: Exclude, Weighting: 1/x, Axis trans: None

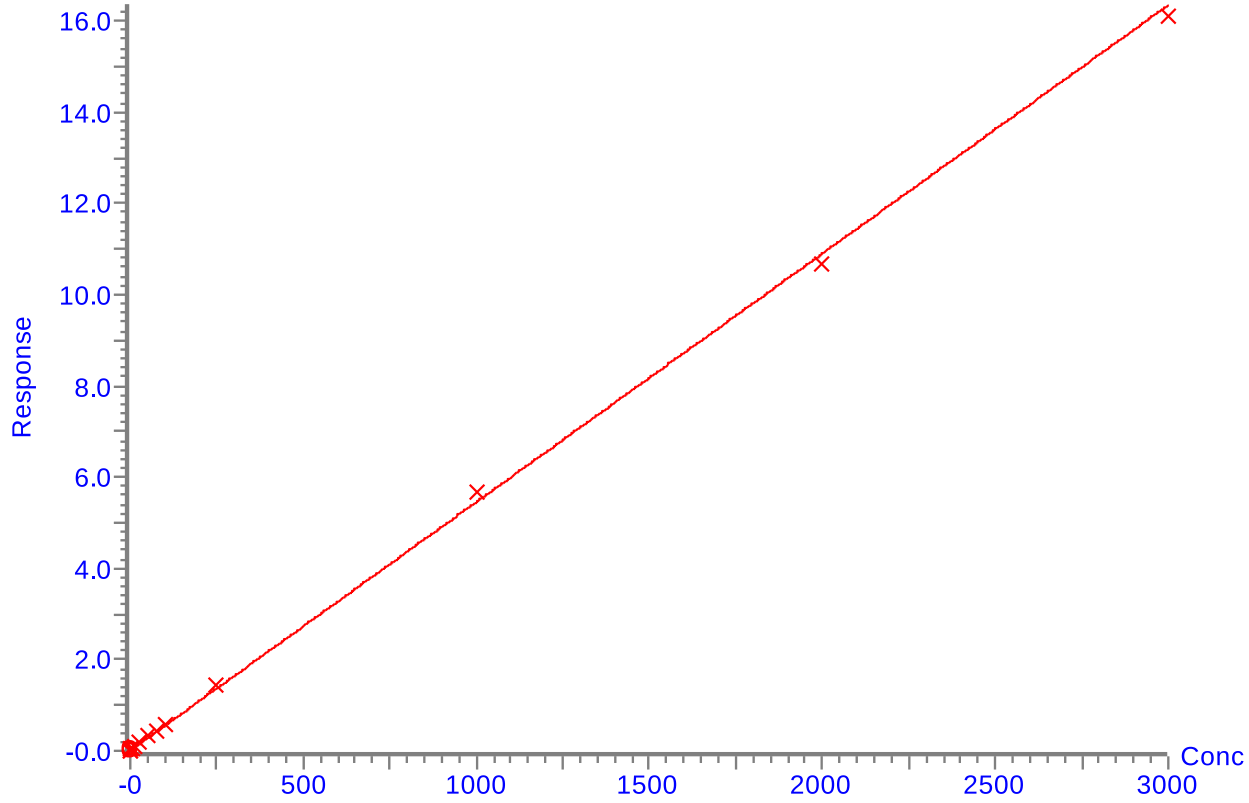

GABA					
	Std Conc (ng/mL)	Response	Conc.	%Dev	S/N
1	0.5	0.007	0.5	-7.7	10.439
2	5	0.031	4.9	-2.1	198.295
3	7.5	0.05	8.4	11.7	137.144
4	10	0.073	12.6	26	537.495
5	50	0.322	58.4	16.8	1193.15
6	75	0.452	82.2	9.6	456.198
7	100	0.567	103.3	3.3	1616.193
8	250	1.465	268.2	7.3	432.797
9	1000	5.65	1036.7	3.7	1310.672
10	2000	10.691	1962.4	-1.9	4118.542
11	3000	16.108	2957.2	-1.4	3097.238

Compound name: 3-Hydrobutyric acid
Correlation coefficient: r = 0.999336, r^2 = 0.998673
Calibration curve: 0.00524439 * x + 0.209765
Response type: Internal Std (Ref 2), Area * (IS Conc. / IS Area)
Curve type: Linear, Origin: Exclude, Weighting: 1/x, Axis trans: None

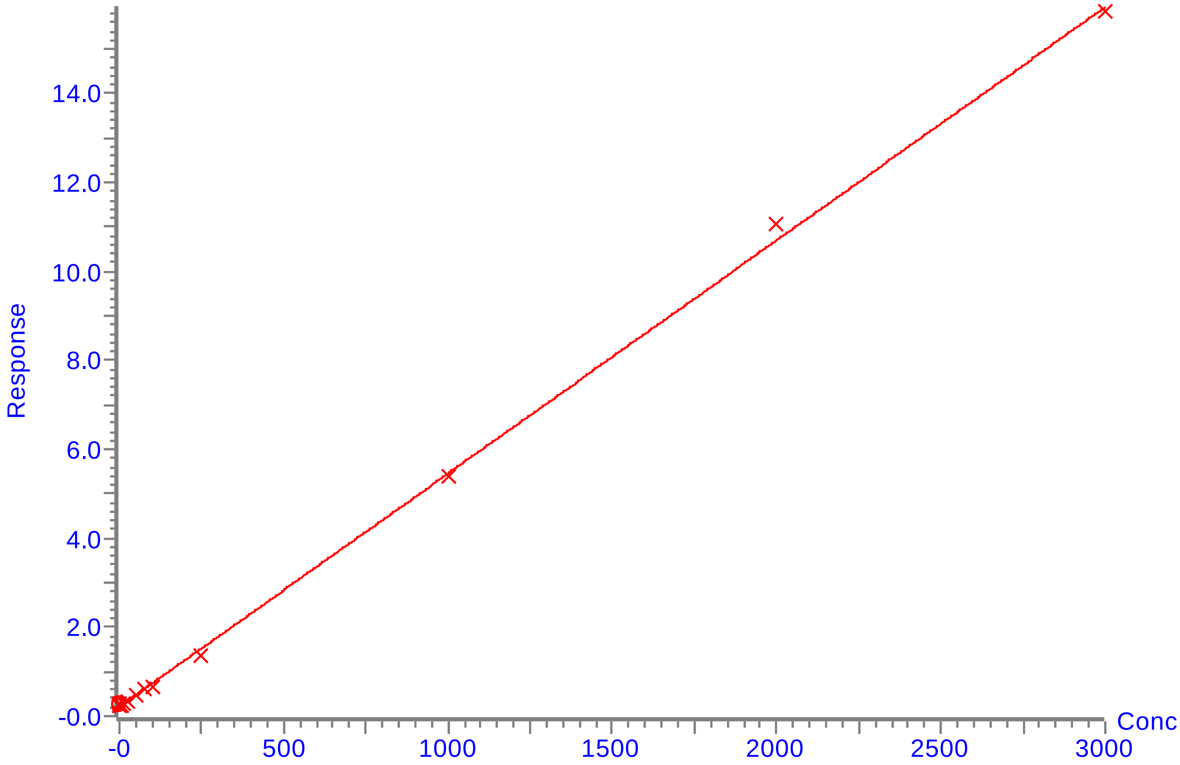

3-Hydroxy Butyric acid					
	Std Conc (ng/mL)	Response	Conc.	%Dev	S/N
1	1	0.215	1.1	7.9	714.481
2	2.5	0.224	2.8	10.7	804.11
3	5	0.237	5.2	3.6	450.592
4	7.5	0.251	7.8	4	683.855
5	10	0.264	10.3	2.9	441.343
6	25	0.34	24.9	-0.4	625.788
7	50	0.46	47.7	-4.6	509.187
8	75	0.605	75.3	0.4	468.197
9	100	0.658	85.4	-14.6	780.848
10	250	1.366	220.4	-11.8	637.171
11	1000	5.402	990	-1	1083.468
12	2000	11.075	2071.8	3.6	1454.768

Compound name: Threonine
Correlation coefficient: r = 0.998979, r^2 = 0.997959
Calibration curve: 0.000456408 * x + 0.00514902
Response type: Internal Std (Ref 2), Area * (IS Conc. / IS Area)
Curve type: Linear, Origin: Exclude, Weighting: 1/x, Axis trans: None

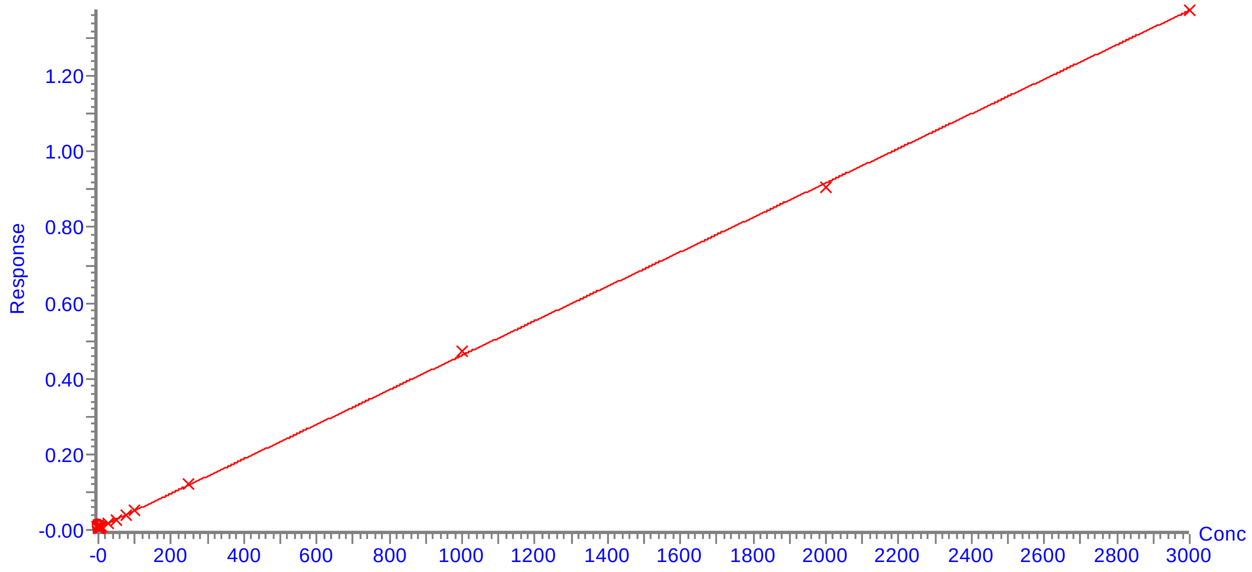

Threonine					
	Std Conc (ng/mL)	Response	Conc.	%Dev	S/N
1	1	0.006	1.1	7.4	13.718
2	2.5	0.006	2.8	12.4	20.776
3	5	0.007	4.7	-5.9	17.717
4	7.5	0.009	8.1	8	34.287
5	25	0.017	26	4.1	25.663
6	50	0.026	46.6	-6.9	15.781
7	75	0.037	69.8	-6.9	81.237
8	100	0.054	106.6	6.6	48.212
9	250	0.123	258.5	3.4	68.792
10	1000	0.472	1023.8	2.4	170.589
11	2000	0.904	1969.6	-1.5	92.007
12	3000	1.373	2996.5	-0.1	185.266

Compound name: Cysteine
Correlation coefficient: r = 0.999712, r^2 = 0.999425
Calibration curve: 0.000130827 * x + 0.000121304
Response type: Internal Std (Ref 2), Area * (IS Conc. / IS Area)
Curve type: Linear, Origin: Exclude, Weighting: 1/x, Axis trans: None

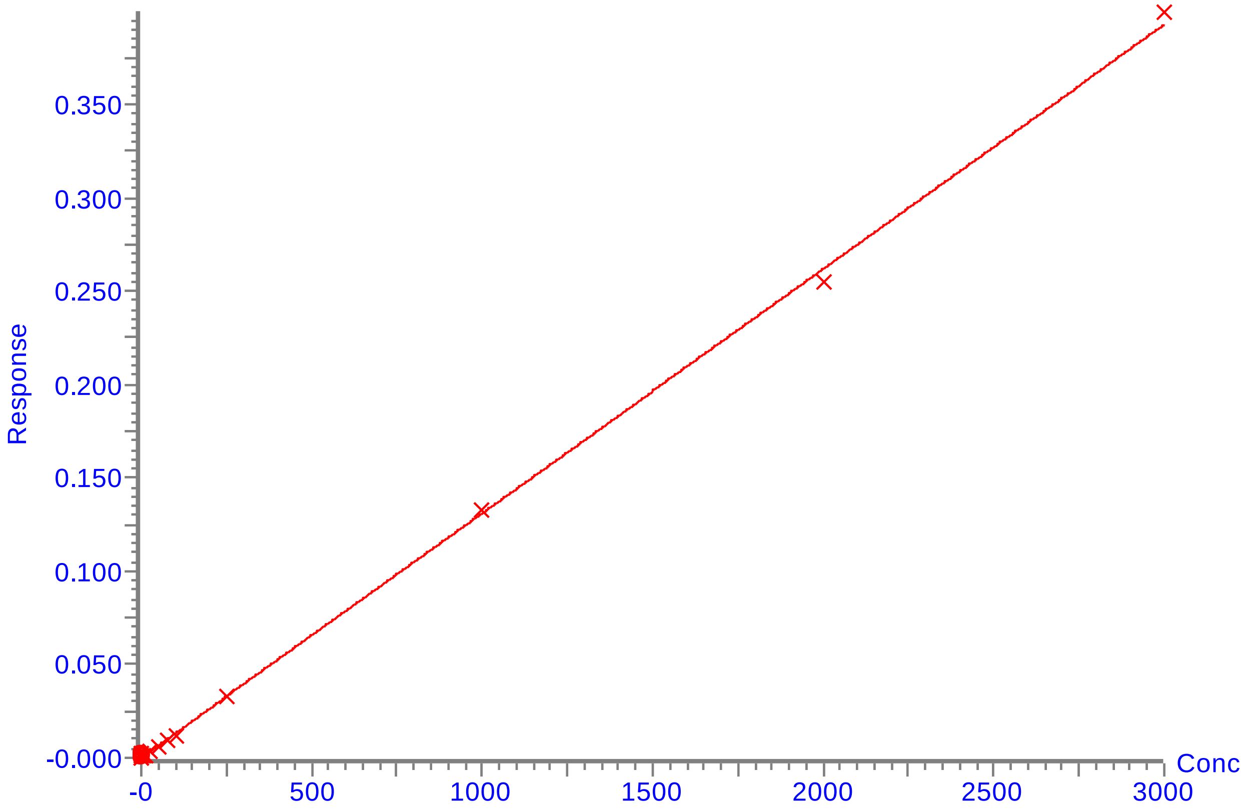

Cysteine					
	Std Conc (ng/mL)	Response	Conc.	%Dev	S/N
1	1	0	1.3	28	1.753
2	2.5	0	2.8	11.3	1.833
3	10	0.001	9.2	-7.7	5.696
4	25	0.003	22.7	-9.1	5.706
5	50	0.006	46	-8	5.855
6	75	0.01	71.9	-4.1	14.601
7	100	0.012	92.1	-7.9	21.387
8	250	0.033	253.9	1.6	32.943
9	1000	0.132	1011.2	1.1	53.516
10	2000	0.255	1950.5	-2.5	54.127
11	3000	0.399	3051.8	1.7	41.875

Compound name: Pyroglutamic acid
Correlation coefficient: r = 0.999522, r^2 = 0.999044
Calibration curve: 0.000244797 * x + 0.00139121
Response type: Internal Std (Ref 2), Area * (IS Conc. / IS Area)
Curve type: Linear, Origin: Exclude, Weighting: 1/x, Axis trans: None

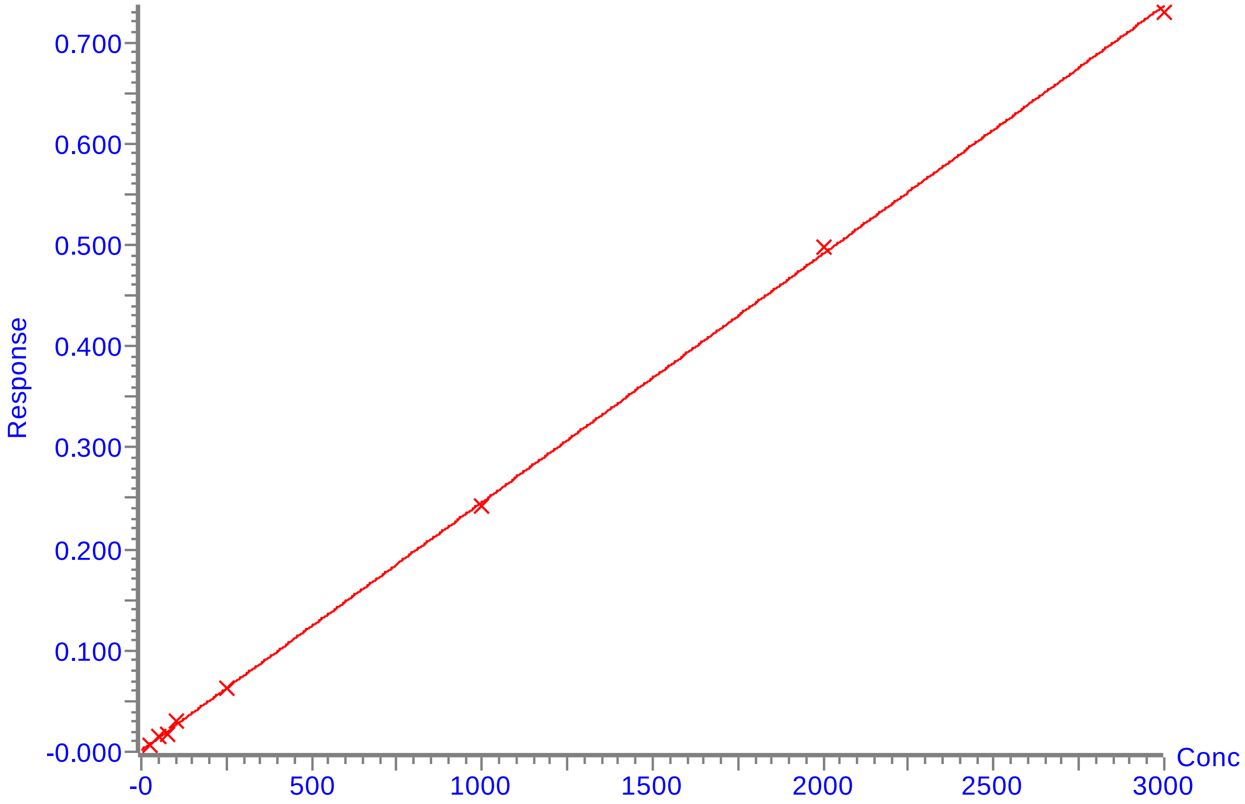

Pyroglutamic acid					
	Std Conc (ng/mL)	Response	Conc.	%Dev	S/N
1	25	0.007	20.9	-16.3	11.172
2	50	0.015	55.4	10.9	11.085
3	75	0.018	67.1	-10.5	14.136
4	100	0.03	115.6	15.6	41.615
5	250	0.063	253.5	1.4	17.663
6	1000	0.242	983.8	-1.6	129.024
7	2000	0.499	2030.9	1.5	167.783
8	3000	0.729	2972.9	-0.9	141.764

Compound name: Cis-Aconitic acid
Correlation coefficient: r = 0.999762, r^2 = 0.999525
Calibration curve: 0.071893 * x + 0.328922
Response type: Internal Std (Ref 2), Area * (IS Conc. / IS Area)
Curve type: Linear, Origin: Exclude, Weighting: 1/x, Axis trans: None

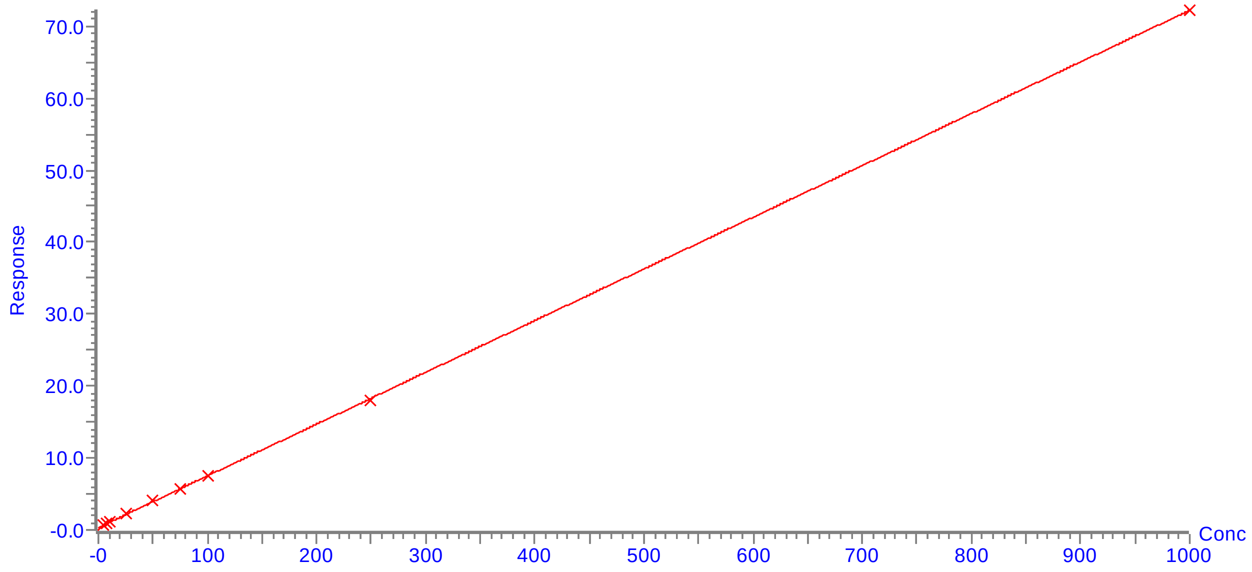

Cis-aconitic acid					
	Std Conc (ng/mL)	Response	Conc.	%Dev	S/N
1	5	0.618	4	-19.5	262.676
2	7.5	0.877	7.6	1.6	421.364
3	10	1.101	10.7	7.4	347.242
4	25	2.212	26.2	4.8	1174.904
5	50	4.194	53.8	7.5	1201.202
6	75	5.662	74.2	-1.1	2392.026
7	100	7.578	100.8	0.8	2716.852
8	250	18.058	246.6	-1.4	2669.967
9	1000	72.118	998.5	-0.1	1408.557

Compound name: Citric acid/isocitric acid
Correlation coefficient: r = 0.999128, r^2 = 0.998257
Calibration curve: 0.00944203 * x + 0.0834325
Response type: Internal Std (Ref 2), Area * (IS Conc. / IS Area)
Curve type: Linear, Origin: Exclude, Weighting: 1/x, Axis trans: None

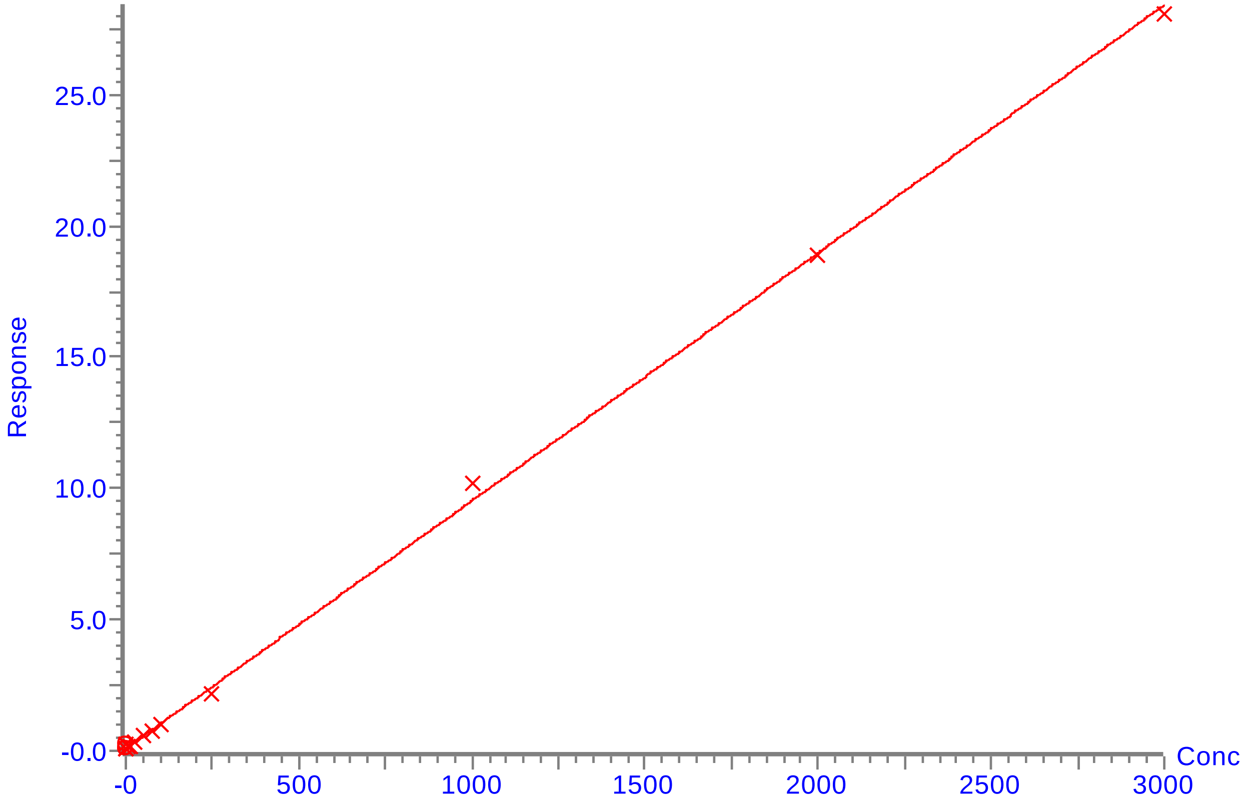

Citric acid/Isocitric acid					
	Std Conc (ng/mL)	Response	Conc.	%Dev	S/N
1	5	0.136	5.6	12.1	131.562
2	7.5	0.158	7.9	6	302.151
3	10	0.184	10.7	6.8	193.272
4	25	0.307	23.7	-5.1	152.824
5	50	0.555	49.9	-0.2	309.744
6	75	0.791	75	0	662.182
7	100	1.016	98.8	-1.2	658.932
8	250	2.198	223.9	-10.4	851.186
9	1000	10.162	1067.4	6.7	1996.178
10	2000	18.889	1991.7	-0.4	3148.883
11	3000	28.106	2967.9	-1.1	1552.699

Compound name: Glutaconic acid
Correlation coefficient: r = 0.997375, r^2 = 0.994756
Calibration curve: 2.34841 * x + -24.7825
Response type: Internal Std (Ref 9), Area * (IS Conc. / IS Area)
Curve type: Linear, Origin: Exclude, Weighting: 1/x, Axis trans: None

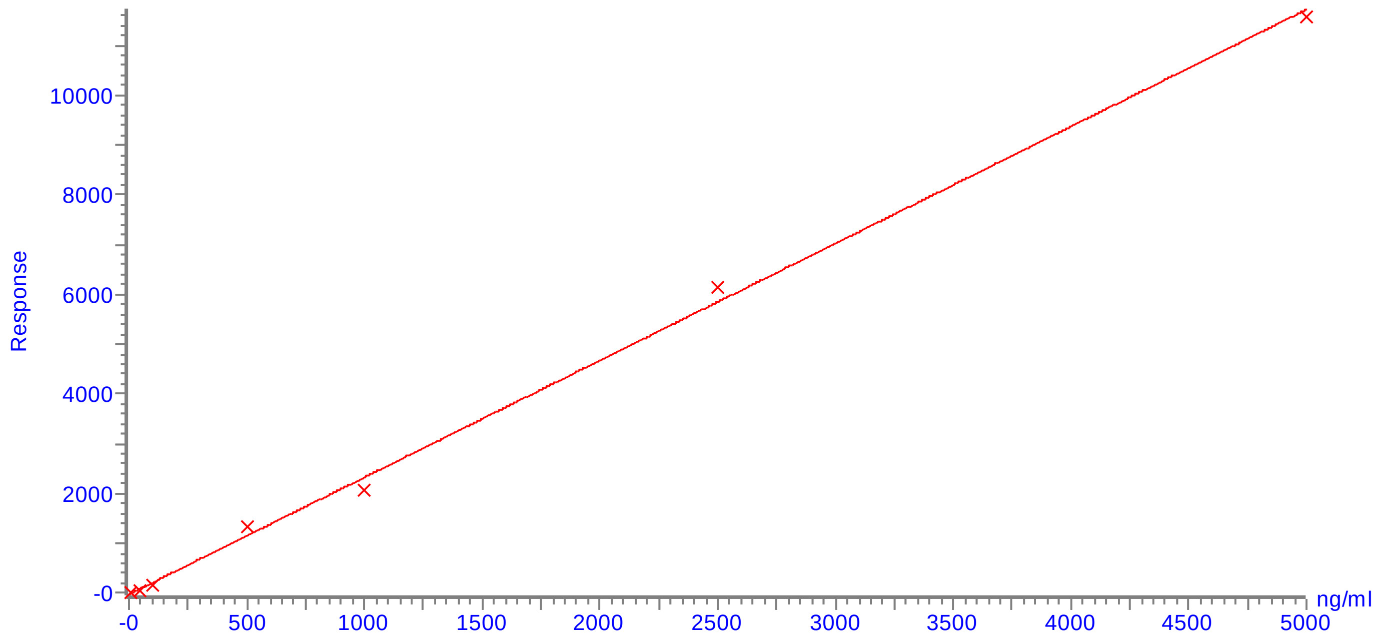

Glutaconic acid					
	Std Conc (ng/mL)	Response	Conc.	%Dev	S/N
1	10	10.540981	15.04	50.4	2564.754
2	50	48.524753	31.22	-37.6	3055.709
3	100	159.631071	78.53	-21.5	1530.47
4	500	1336.1956	579.53	15.9	889.113
5	1000	2061.873038	888.54	-11.1	1412.777
6	2500	6142.626038	2626.20	5	3393.81
7	5000	11578.58322	4940.94	-1.2	3629.895

Compound name: 4-Hydroxy proline_1
Correlation coefficient: r = 0.995351, r^2 = 0.990724
Calibration curve: 0.00443723 * x + 0.0118856
Response type: Internal Std (Ref 2), Area * (IS Conc. / IS Area)
Curve type: Linear, Origin: Exclude, Weighting: 1/x, Axis trans: None

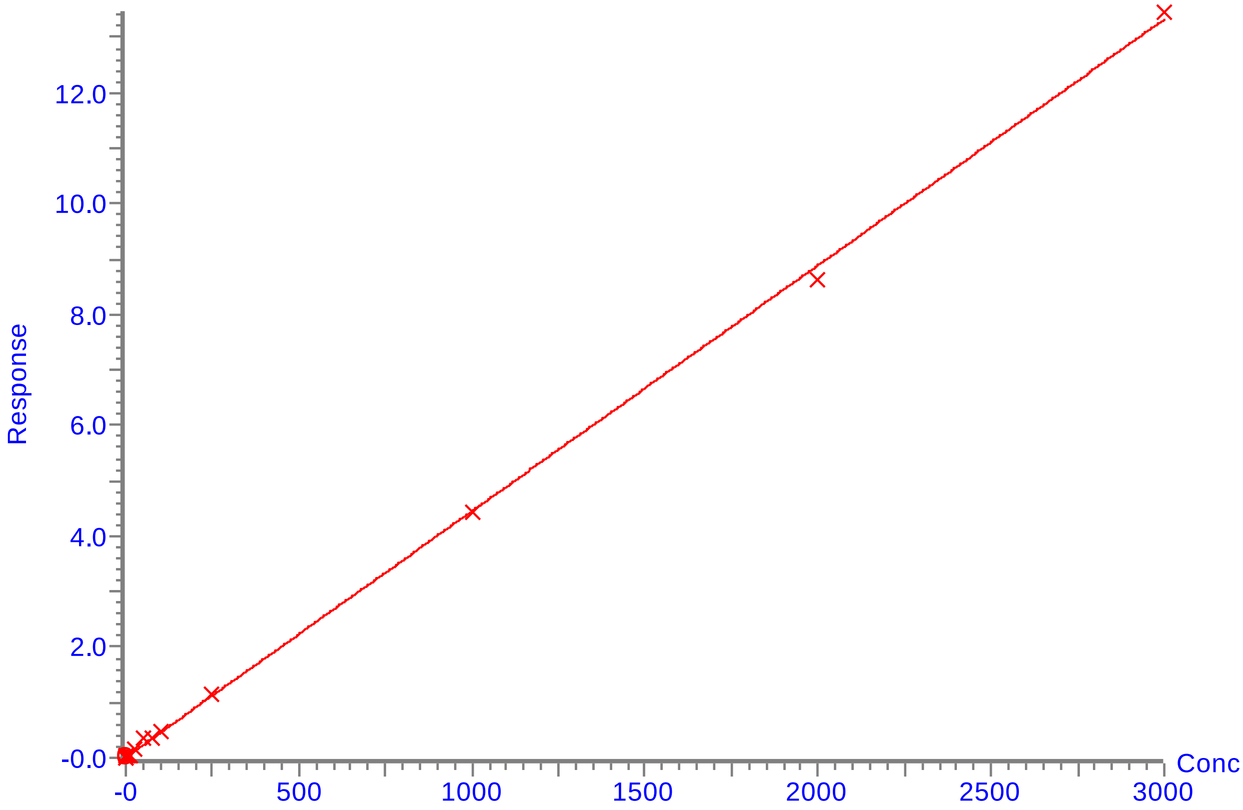

4-Hydroxy proline					
	Std Conc (ng/mL)	Response	Conc.	%Dev	S/N
1	0.5	0.014	0.5	4.4	11.07
2	5	0.035	5.2	3.4	78.973
3	7.5	0.048	8.1	7.4	66.68
4	10	0.057	10.1	1.2	126.872
5	75	0.363	79.1	5.5	669.205
6	100	0.461	101.2	1.2	462.217
7	250	1.141	254.5	1.8	1350.353
8	1000	4.412	991.7	-0.8	2251.292
9	2000	8.639	1944.3	-2.8	1613.748
10	3000	13.453	3029.3	1	1397.954

Compound name: Asparagine
Correlation coefficient: r = 0.997806, r^2 = 0.995618
Calibration curve: 0.00278259 * x + 0.00729158
Response type: Internal Std (Ref 2), Area * (IS Conc. / IS Area)
Curve type: Linear, Origin: Exclude, Weighting: 1/x, Axis trans: None

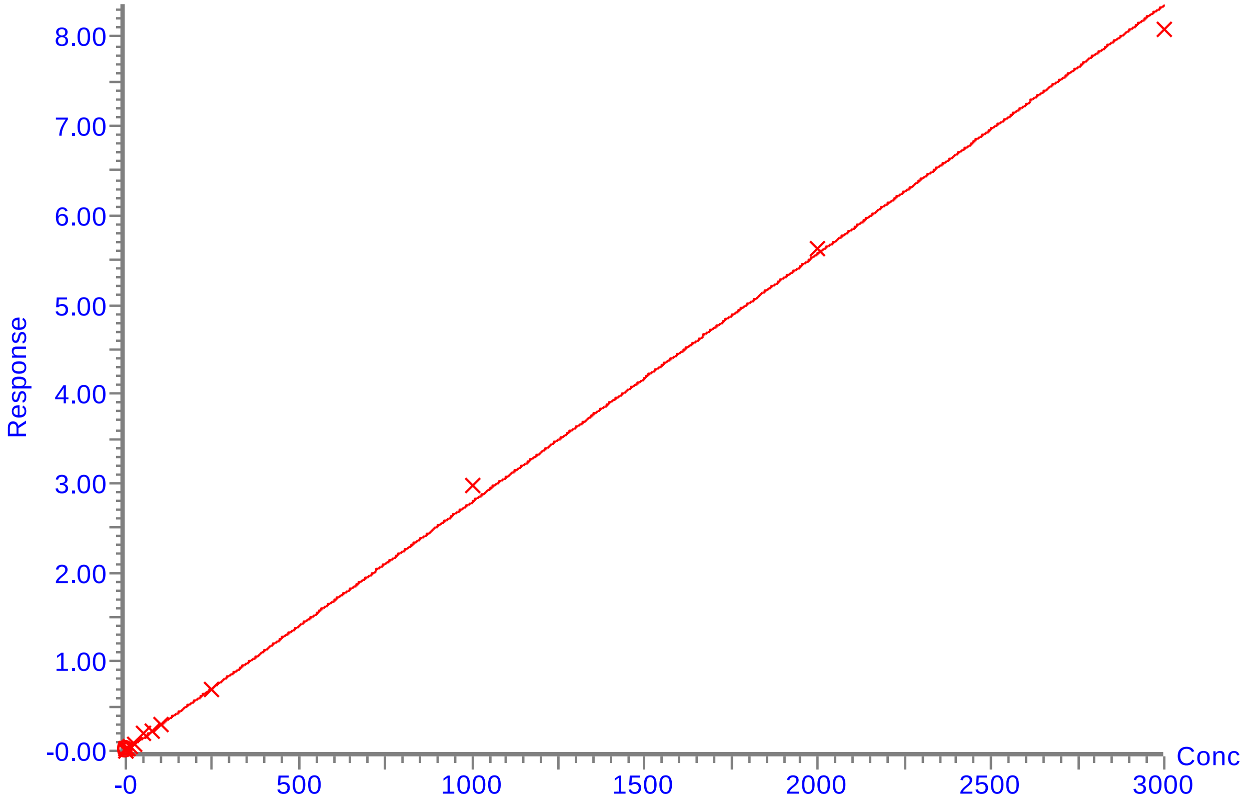

Asparagine					
	Std Conc (ng/mL)	Response	Conc.	%Dev	S/N
1	1	0.01	0.9	-8.3	16.588
2	5	0.022	5.2	3.7	18.472
3	7.5	0.026	6.9	-8.1	140.86
4	10	0.038	11	10.4	57.269
5	25	0.082	26.8	7.3	186.195
6	50	0.193	66.7	33.4	413.956
7	75	0.229	79.6	6.1	325.61
8	100	0.292	102.4	2.4	271.221
9	250	0.681	242.2	-3.1	686.278
10	1000	2.981	1068.6	6.9	1691.112
11	2000	5.621	2017.3	0.9	1242.195
12	3000	8.081	2901.4	-3.3	1112.973

Compound name: Ornithine
Correlation coefficient: r = 0.998451, r^2 = 0.996905
Calibration curve: 0.00703109 * x + 0.0208763
Response type: Internal Std (Ref 9), Area * (IS Conc. / IS Area)
Curve type: Linear, Origin: Exclude, Weighting: 1/x, Axis trans: None

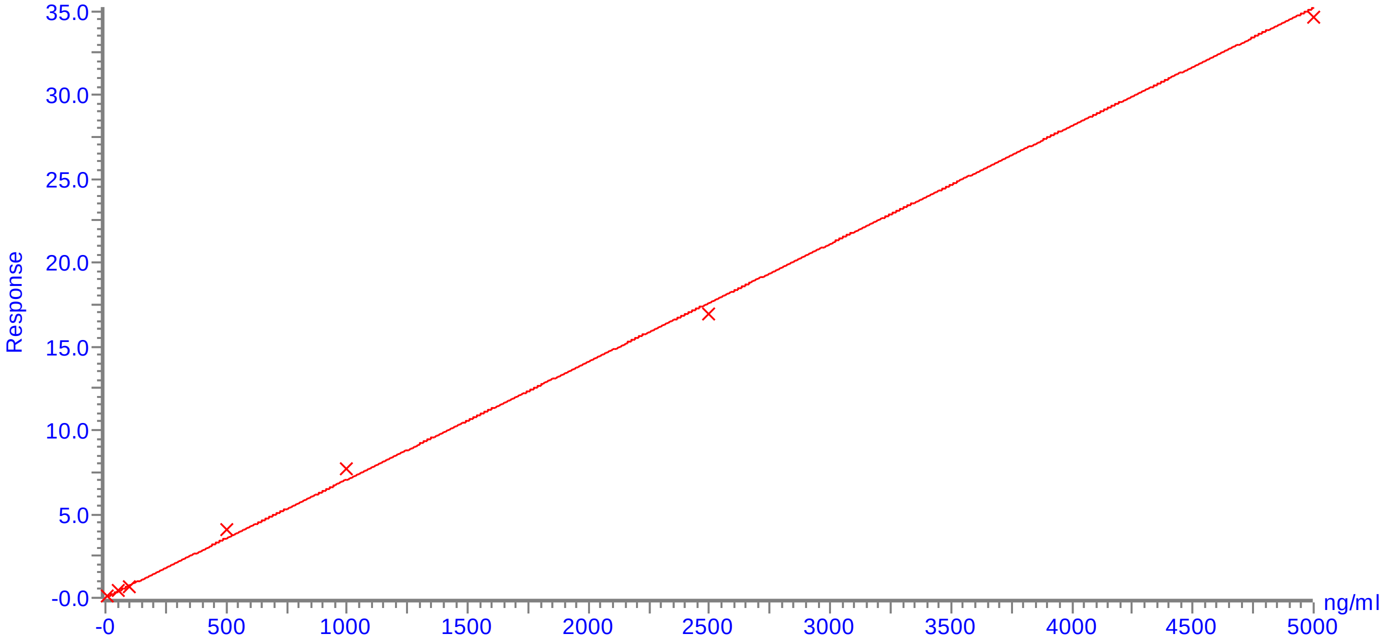

Ornithine					
	Std Conc (ng/mL)	Response	Conc.	%Dev	S/N
1	10	0.078005	8.13	-18.7	81.35
2	50	0.395586	53.29	6.6	65.633
3	100	0.667839	92.01	-8	49.484
4	500	4.079116	577.18	15.4	65.398
5	1000	7.745784	1098.68	9.9	55.301
6	2500	16.976087	2411.46	-3.5	59.303
7	5000	34.608548	4919.24	-1.6	156.764

Compound name: Glutaric acid
Correlation coefficient: r = 0.999083, r^2 = 0.998166
Calibration curve: 0.00278281 * x + 0.00400399
Response type: Internal Std (Ref 2), Area * (IS Conc. / IS Area)
Curve type: Linear, Origin: Exclude, Weighting: 1/x, Axis trans: None

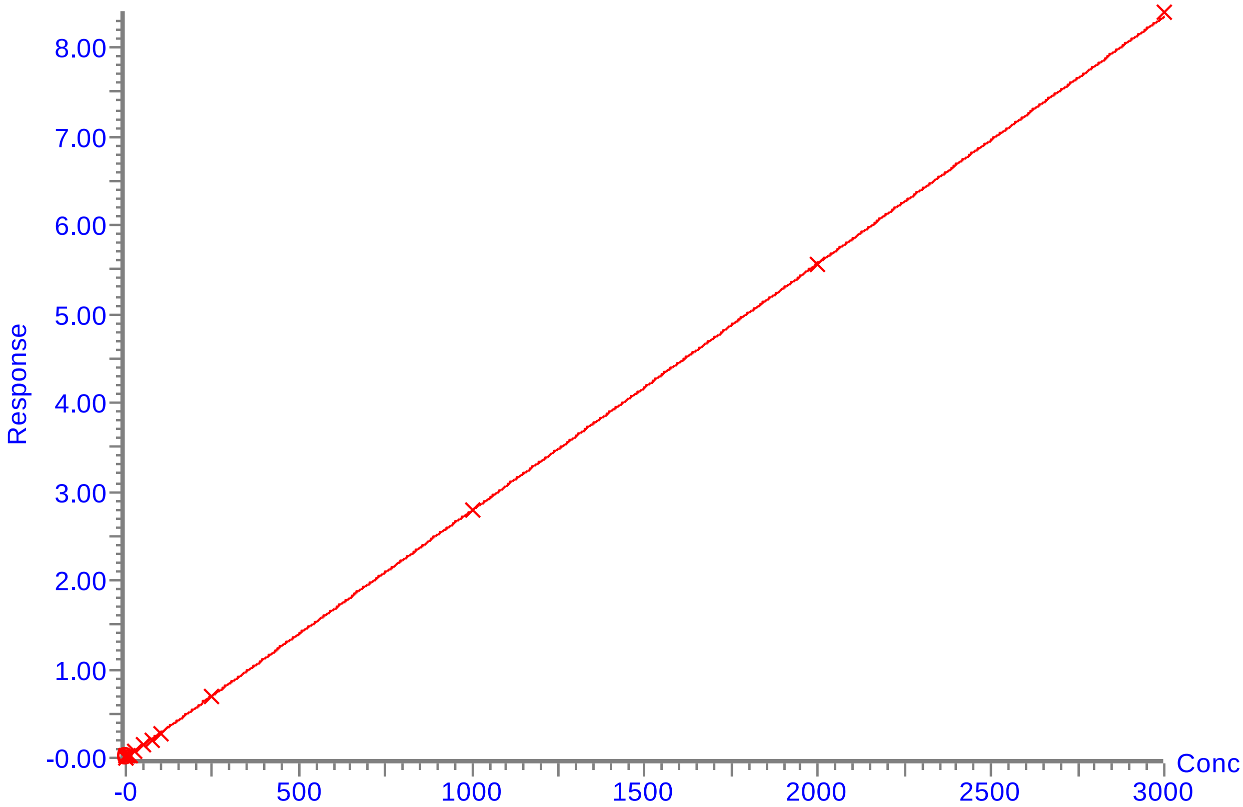

Glutaric acid					
	Std Conc (ng/mL)	Response	Conc.	%Dev	S/N
1	0.5	0.005	0.4	-14.2	18.001
2	1	0.007	1.2	20.3	21.331
3	5	0.02	5.7	13.7	29.349
4	7.5	0.025	7.7	2.5	39.328
5	10	0.036	11.4	13.8	95.076
6	25	0.071	24.2	-3.3	147.115
7	50	0.137	47.9	-4.1	101.843
8	100	0.279	98.6	-1.4	271.273
9	250	0.682	243.7	-2.5	588.313
10	1000	2.793	1002.2	0.2	1175.049
11	2000	5.552	1993.8	-0.3	589.023
12	3000	8.399	3016.9	0.6	862.082

Compound name: Adipic acid
Correlation coefficient: r = 0.998075, r^2 = 0.996154
Calibration curve: 0.0093574 * x + 0.036464
Response type: Internal Std (Ref 2), Area * (IS Conc. / IS Area)
Curve type: Linear, Origin: Exclude, Weighting: 1/x, Axis trans: None

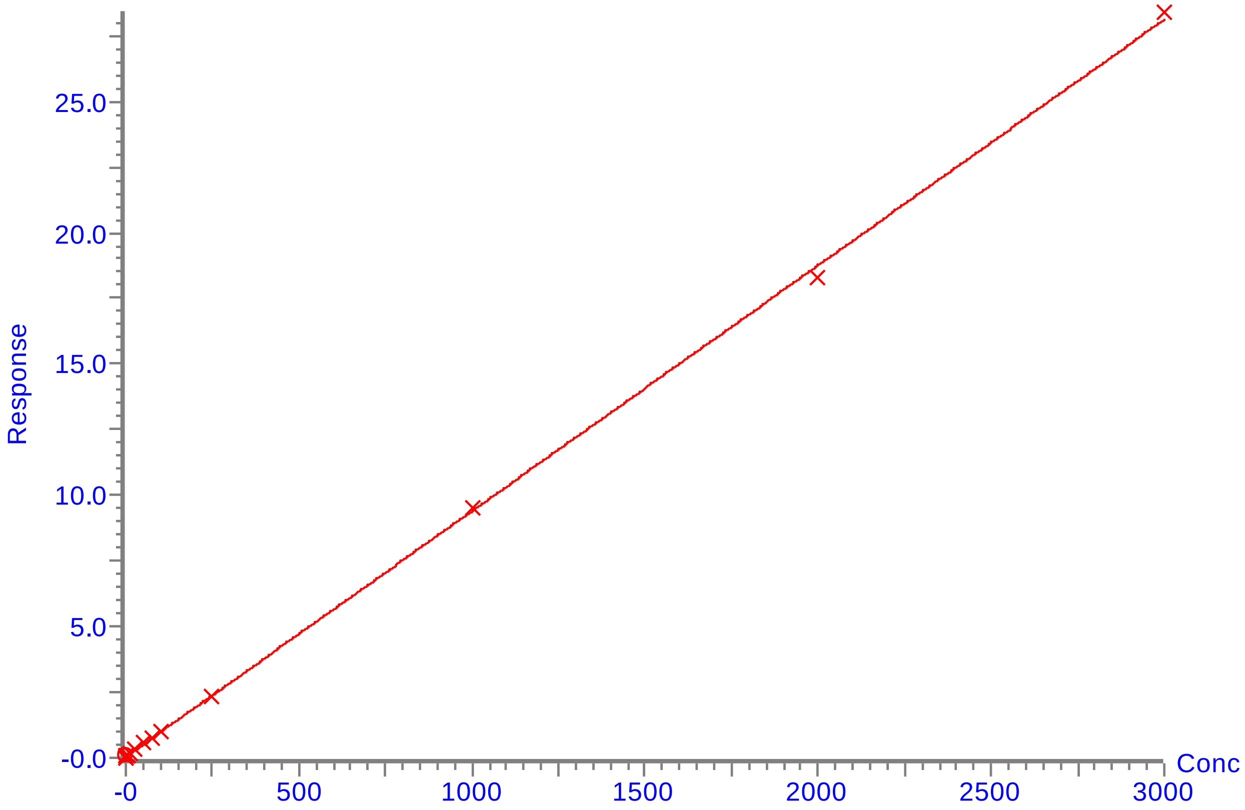

Adipic acid					
	Std Conc (ng/mL)	Response	Conc.	%Dev	S/N
1	5	0.087	5.4	8.1	165.821
2	7.5	0.109	7.8	3.8	180.741
3	10	0.135	10.6	5.6	348.563
4	25	0.298	28	11.9	608.109
5	50	0.551	55	10	1766.288
6	75	0.719	73	-2.7	1308.082
7	100	1.027	105.8	5.8	1050.058
8	250	2.36	248.3	-0.7	3313.35
9	1000	9.511	1012.5	1.3	5591.138
10	2000	18.303	1952.1	-2.4	3789.475
11	3000	28.394	3030.5	1	9399.621

Compound name: Glutamic acid_366
Correlation coefficient: r = 0.995877, r^2 = 0.991772
Calibration curve: 0.0132351 * x + 2.4606
Response type: Internal Std (Ref 2), Area * (IS Conc. / IS Area)
Curve type: Linear, Origin: Exclude, Weighting: 1/x, Axis trans: None

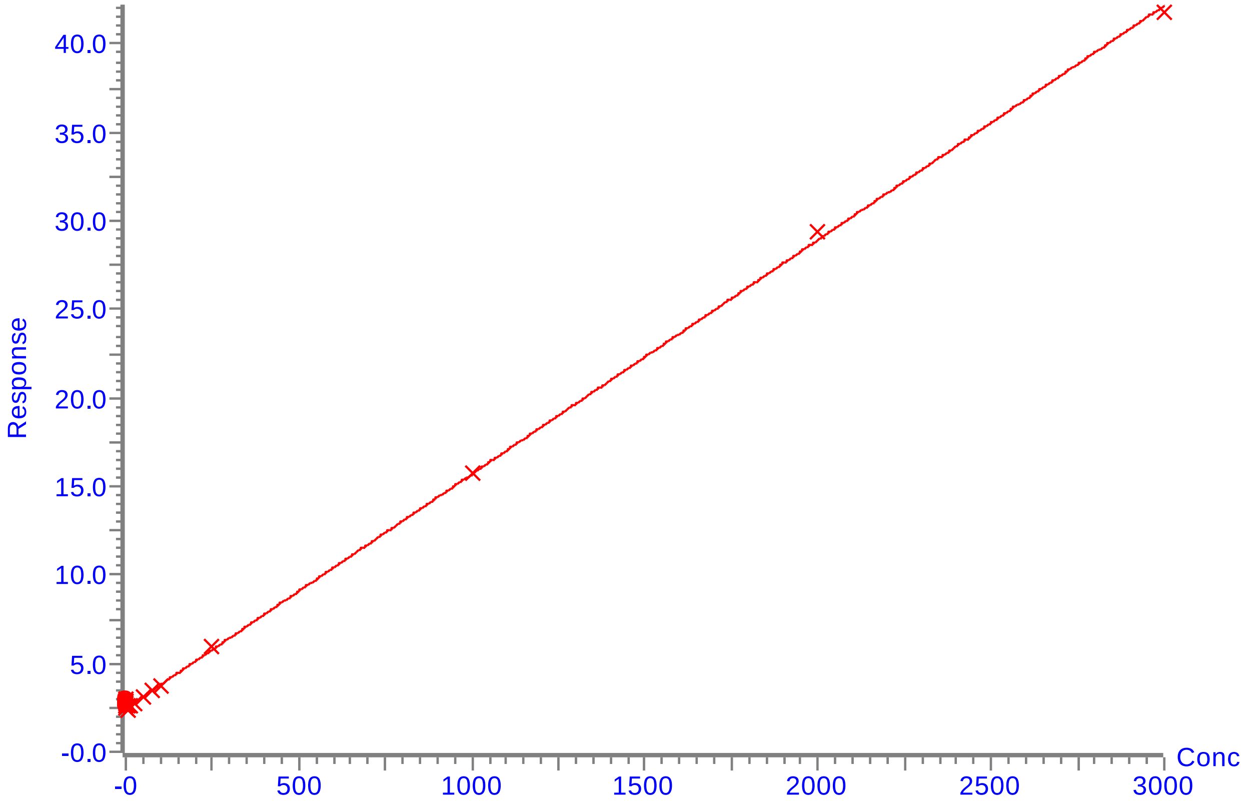

Glutamic acid					
	Std Conc (ng/mL)	Response	Conc.	%Dev	S/N
1	7.5	2.572	8.4	11.9	2351.609
2	10	2.616	11.7	17.3	2674.701
3	25	2.789	24.8	-0.9	3141.115
4	50	3.074	46.3	-7.3	2203.972
5	75	3.455	75.1	0.2	2953.287
6	100	3.752	97.6	-2.4	3152.474
7	250	5.971	265.2	6.1	3196.995
8	1000	15.728	1002.4	0.2	5718.103
9	2000	29.385	2034.3	1.7	5983.388
10	3000	41.75	2968.6	-1	5562.169

Compound name: Mevalonic acid
Correlation coefficient: r = 0.999014, r^2 = 0.998030
Calibration curve: 0.00937525 * x + 0.00491091
Response type: Internal Std (Ref 2), Area * (IS Conc. / IS Area)
Curve type: Linear, Origin: Exclude, Weighting: 1/x, Axis trans: None

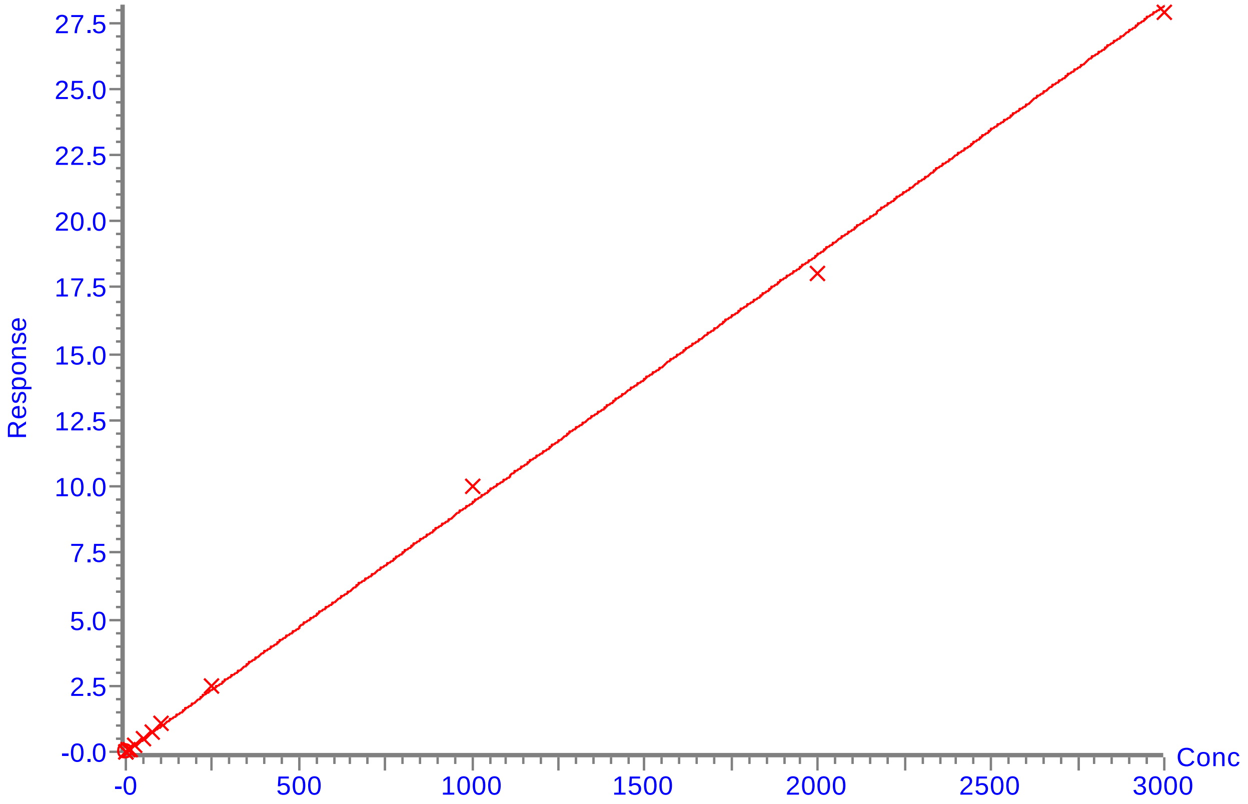

Mevalonic acid					
	Std Conc (ng/mL)	Response	Conc.	%Dev	S/N
1	5	0.053	5.2	3.1	58.476
2	7.5	0.083	8.3	11.2	77.254
3	10	0.101	10.2	2.4	85.062
4	25	0.255	26.7	6.7	224.564
5	50	0.474	50.1	0.1	395.52
6	75	0.727	77	2.7	855.387
7	100	1.045	110.9	10.9	595.855
8	250	2.5	266.2	6.5	980.161
9	1000	10.002	1066.4	6.6	5317.859
10	2000	18.051	1924.8	-3.8	4089.659
11	3000	27.912	2976.6	-0.8	5951.023

Compound name: Hydroxy glutaric acid_209
Correlation coefficient: r = 0.999213, r^2 = 0.998427
Calibration curve: 0.00432507 * x + 0.00487617
Response type: Internal Std (Ref 2), Area * (IS Conc. / IS Area)
Curve type: Linear, Origin: Exclude, Weighting: 1/x, Axis trans: None

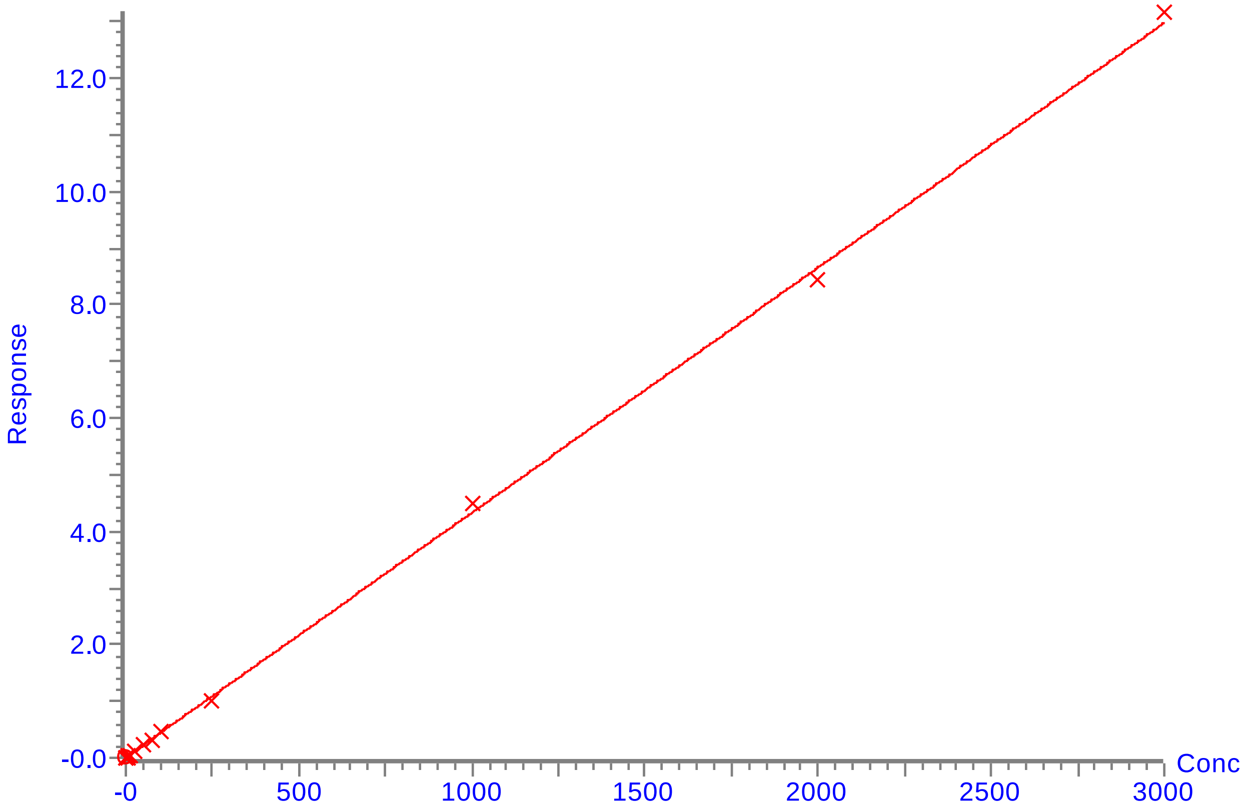

2-Hydroxy glutaric acid					
	Std Conc (ng/mL)	Response	Conc.	%Dev	S/N
1	0.1	0.005	0.1	-12.1	21.875
2	1	0.009	1	0.9	39.404
3	2.5	0.014	2.2	-11.1	34.338
4	7.5	0.035	7.1	-5.9	49.336
5	10	0.043	8.8	-11.7	62.991
6	50	0.22	49.7	-0.7	454.623
7	75	0.3	68.2	-9.1	654.408
8	100	0.466	106.5	6.5	879.274
9	250	1.013	233	-6.8	2217.616
10	1000	4.493	1037.6	3.8	21505.275
11	2000	8.424	1946.5	-2.7	32870.682
12	3000	13.167	3043.2	1.4	17852.673

Compound name: Dihydroorotic acid
Correlation coefficient: r = 0.999571, r^2 = 0.999142
Calibration curve: 0.00687943 * x + 0.709507
Response type: Internal Std (Ref 2), Area * (IS Conc. / IS Area)
Curve type: Linear, Origin: Exclude, Weighting: 1/x, Axis trans: None

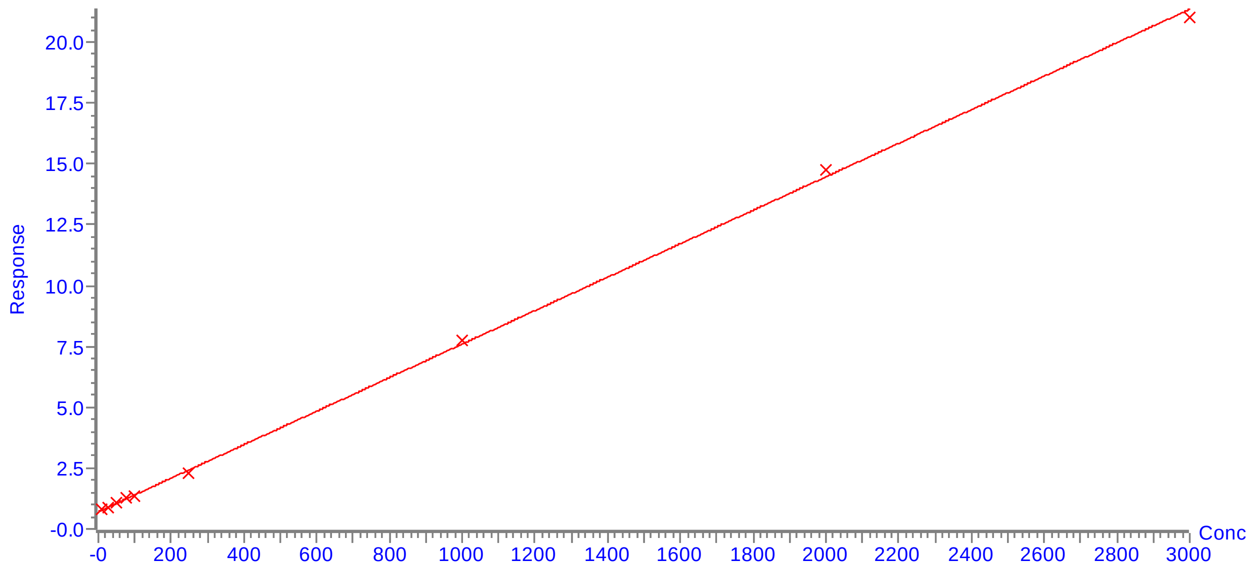

Dihydroorotic acid					
	Std Conc (ng/mL)	Response	Conc.	%Dev	S/N
1	10	0.781	10.3	3.5	1432.641
2	25	0.868	23	-8.1	1102.432
3	50	1.068	52.1	4.1	1668.92
4	75	1.275	82.3	9.7	1280.8
5	100	1.374	96.6	-3.4	1012.984
6	250	2.276	227.8	-8.9	1184.004
7	1000	7.775	1027.1	2.7	1998.545
8	2000	14.727	2037.6	1.9	2702.132
9	3000	21.027	2953.3	-1.6	3671.185

Compound name: 2,2-dimethyl glutaric acid
Correlation coefficient: r = 0.998795, r^2 = 0.997591
Calibration curve: 0.0356825 * x + -0.38609
Response type: Internal Std (Ref 9), Area * (IS Conc. / IS Area)
Curve type: Linear, Origin: Exclude, Weighting: 1/x, Axis trans: None

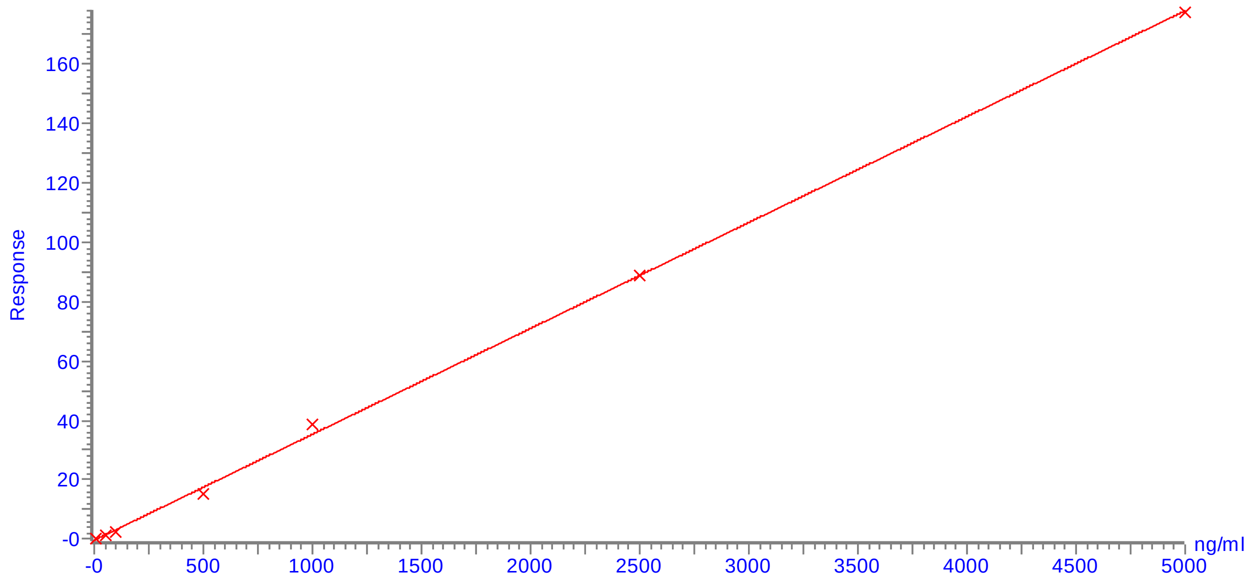

2,2-dimethyl glutaric acid					
	Std Conc (ng/mL)	Response	Conc.	%Dev	S/N
1	10	0.117666	14.12	41.2	91.188
2	50	1.056333	40.42	-19.2	153.423
3	100	2.476715	80.23	-19.8	213.698
4	500	15.489613	444.92	-11	298.275
5	1000	38.418582	1087.50	8.7	177.175
6	2500	89.099081	2507.81	0.3	184.401
7	5000	177.491396	4985.00	-0.3	382.643

Compound name: 2-Hydroxyoctanoic acid
Correlation coefficient: r = 0.996977, r^2 = 0.993962
Calibration curve: 0.111386 * x + 0.106068
Response type: Internal Std (Ref 4), Area * (IS Conc. / IS Area)
Curve type: Linear, Origin: Exclude, Weighting: 1/x, Axis trans: None

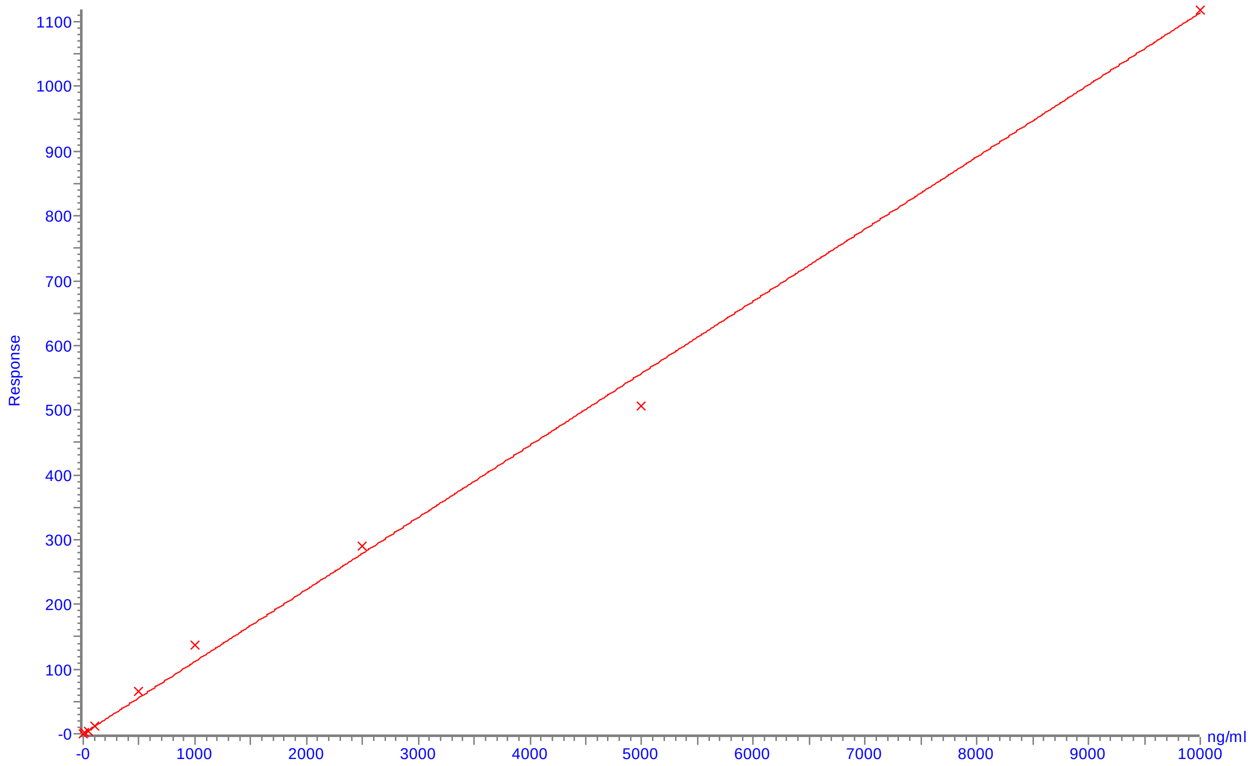

2-Hydroxy octanoic acid					
	Std Conc (ng/mL)	Response	Conc.	%Dev	S/N
1	1	0.106	0.5	-52.6	394.858
2	5	0.769	6.2	24.5	461.058
3	10	1.412	11.8	18	578.191
4	50	5.085	43.7	-12.7	401.847
5	100	12.2	105.4	5.4	740.947
6	500	66.702	578.2	15.6	351.503
7	1000	114.295	991.1	-0.9	369.303
8	2500	293.928	2549.5	2	193.089
9	5000	597.148	5180.1	3.6	269.731
10	10000	1118.094	9699.5	-3	463.901

Compound name: 3-Hydroxy octanoic acid
Correlation coefficient: r = 0.996814, r^2 = 0.993637
Calibration curve: 0.0257817 * x + -0.0668806
Response type: Internal Std (Ref 4), Area * (IS Conc. / IS Area)
Curve type: Linear, Origin: Exclude, Weighting: 1/x, Axis trans: None

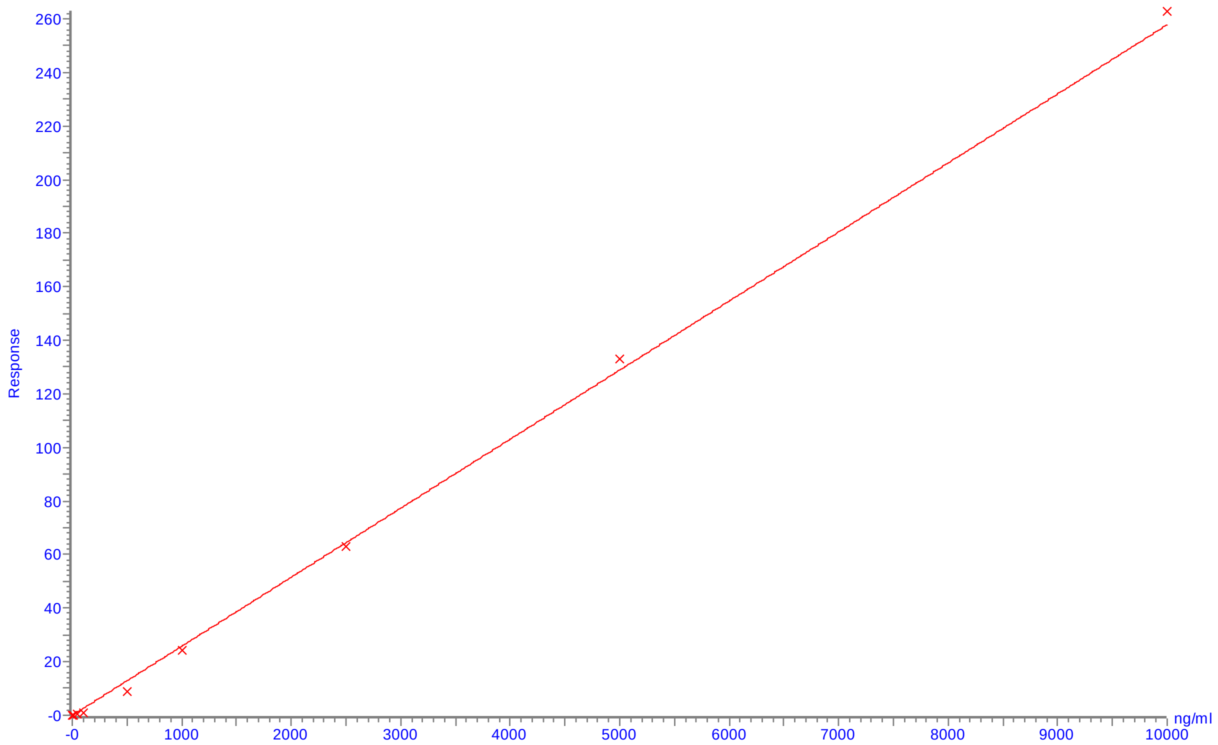

3-Hydroxy octanoic acid					
	Std Conc (ng/mL)	Response	Conc.	%Dev	S/N
1	5	0.044	4.3	-14.6	144.845
2	10	0.09	6.1	-38.7	342.514
3	50	0.604	26.8	-46.4	340.841
4	100	0.858	37	-63	185.768
5	500	8.712	352.7	-29.5	393.728
6	1000	24.354	981.6	-1.8	447.17
7	2500	62.968	2533.9	1.4	144.924
8	5000	115.681	4653.1	-6.9	753.258
9	10000	262.8	10567.5	5.7	859.763

Compound name: Hippuric acid
Correlation coefficient: r = 0.997902, r^2 = 0.995808
Calibration curve: 0.000401555 * x + 0.01879
Response type: Internal Std (Ref 2), Area * (IS Conc. / IS Area)
Curve type: Linear, Origin: Exclude, Weighting: 1/x, Axis trans: None

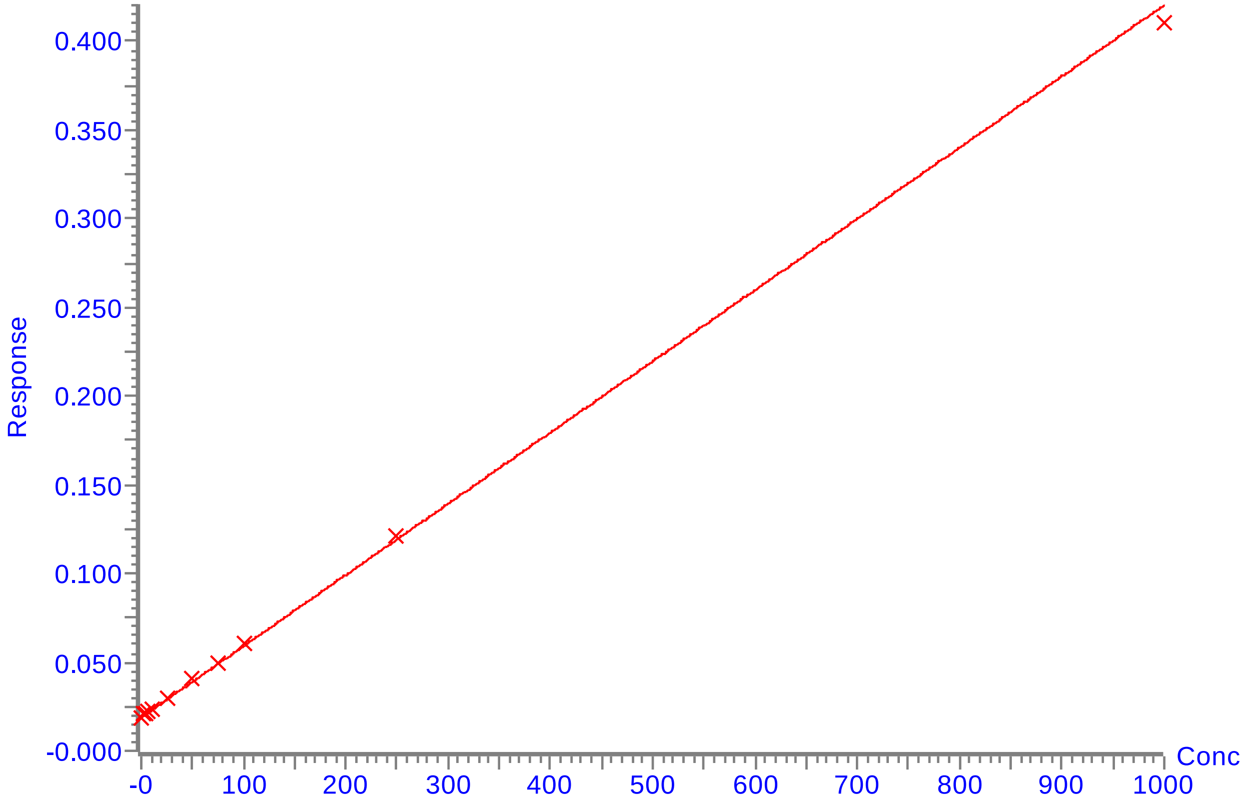

Hippuric acid					
	Std Conc (ng/mL)	Response	Conc.	%Dev	S/N
1	5	0.021	5.8	16.5	47.737
2	7.5	0.022	7.7	2.4	64.29
3	10	0.024	12.6	26.2	28.452
4	25	0.03	28.3	13.1	47
5	50	0.04	53.5	7	134.372
6	75	0.05	77.9	3.8	119.733
7	100	0.061	105.1	5.1	103.963
8	250	0.121	255.5	2.2	108.609
9	1000	0.411	975.7	-2.4	343.67

Compound name: Arginine
Correlation coefficient: r = 0.998952, r^2 = 0.997906
Calibration curve: 1.95965 * x + -14.6425
Response type: Internal Std (Ref 9), Area * (IS Conc. / IS Area)
Curve type: Linear, Origin: Exclude, Weighting: 1/x, Axis trans: None

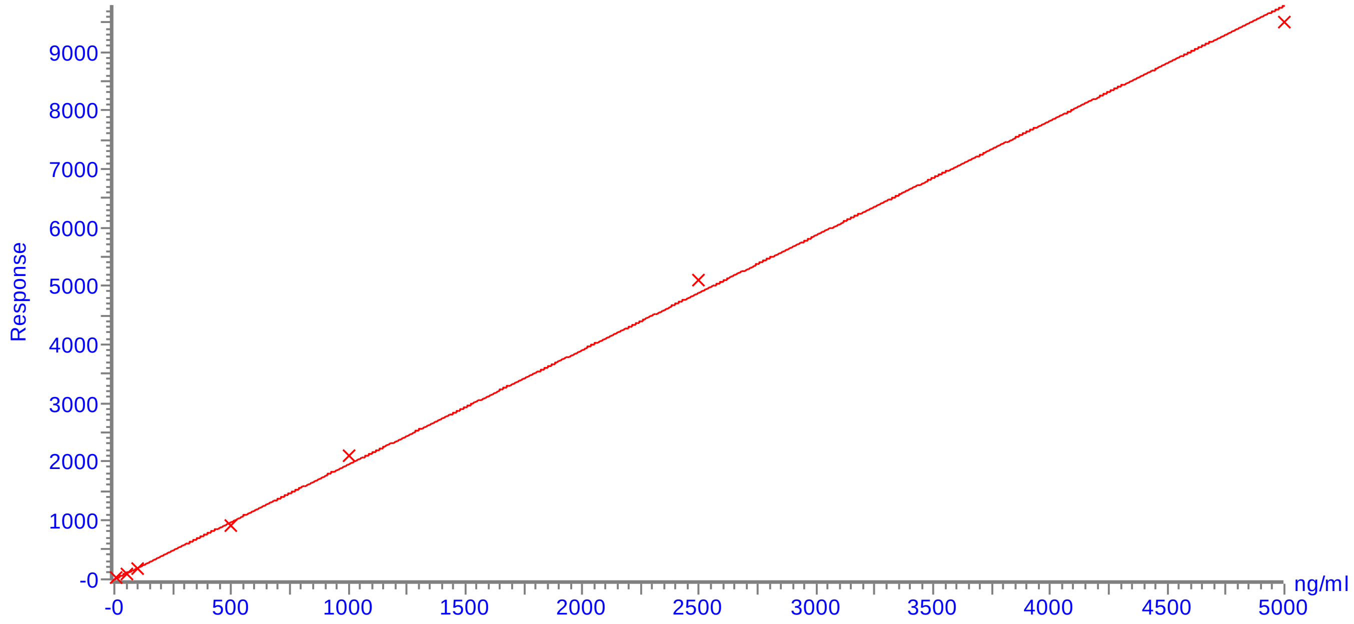

Arginine					
	Std Conc (ng/mL)	Response	Conc.	%Dev	S/N
1	5	0.422	4.5	-9.1	344.129
2	7.5	0.679	7.7	2.7	333.202
3	10	0.856	9.9	-1.2	470.464
4	25	2.124	25.5	1.9	568.347
5	50	4.312	52.4	4.8	1465.04
6	75	6.281	76.6	2.2	1766.518
7	100	8.201	100.3	0.3	5199.527
8	250	20.002	245.5	-1.8	5029.683

Compound name: 10-Undecenoic acid
Correlation coefficient: r = 0.998897, r^2 = 0.997795
Calibration curve: 0.00658025 * x + 0.0014644
Response type: Internal Std (Ref 2), Area * (IS Conc. / IS Area)
Curve type: Linear, Origin: Exclude, Weighting: 1/x, Axis trans: None

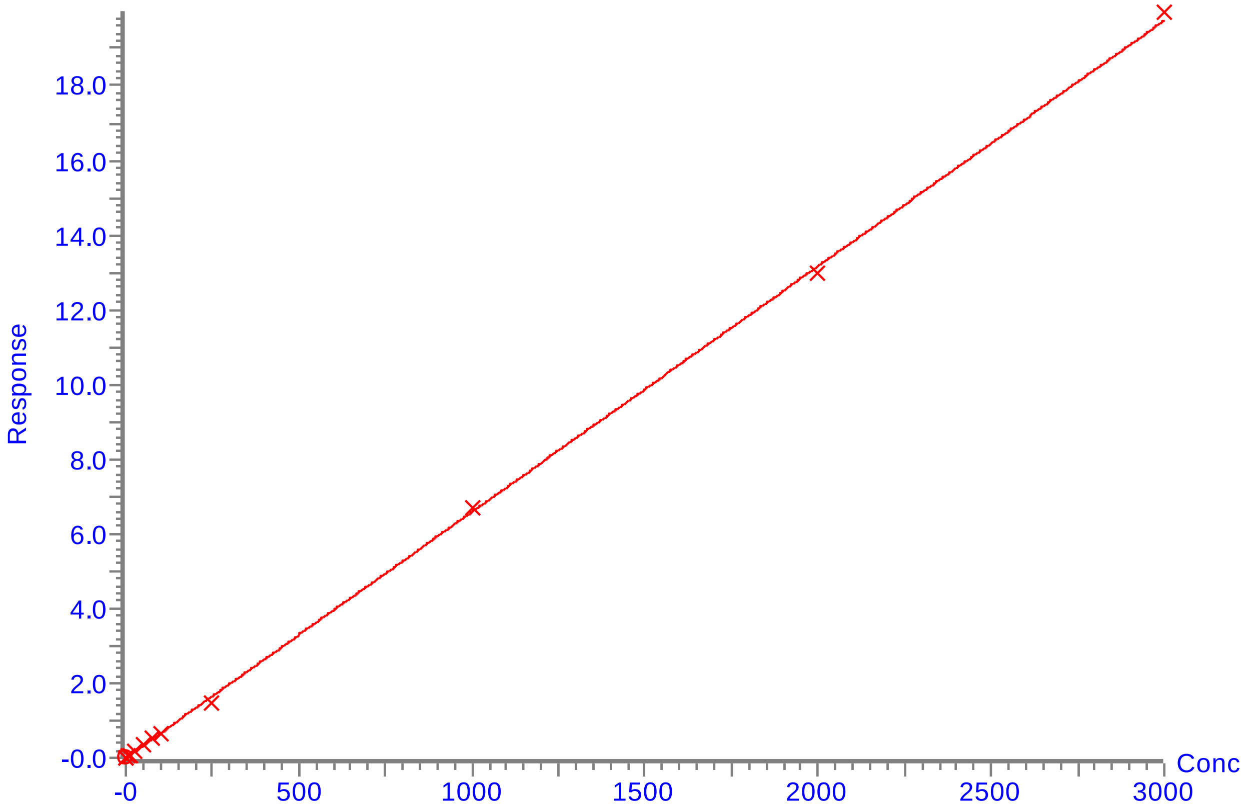

10-undecenoic acid					
	Std Conc (ng/mL)	Response	Conc.	%Dev	S/N
1	5	0.035	5.1	1.1	81.014
2	7.5	0.055	8.2	9.2	152.38
3	10	0.071	10.6	6	166.352
4	25	0.185	27.9	11.5	290.09
5	50	0.364	55.2	10.3	425.348
6	75	0.515	78	4	689.435
7	100	0.665	100.9	0.9	719.426
8	250	1.458	221.4	-11.5	880.615
9	1000	6.67	1013.4	1.3	3513.382
10	2000	12.971	1971.1	-1.4	2934.238
11	3000	19.966	3034	1.1	1799.157

Compound name: Tridecanoic acid
Correlation coefficient: r = 0.999471, r^2 = 0.998941
Calibration curve: 0.0161738 * x + 0.0606132
Response type: Internal Std (Ref 2), Area * (IS Conc. / IS Area)
Curve type: Linear, Origin: Exclude, Weighting: 1/x, Axis trans: None

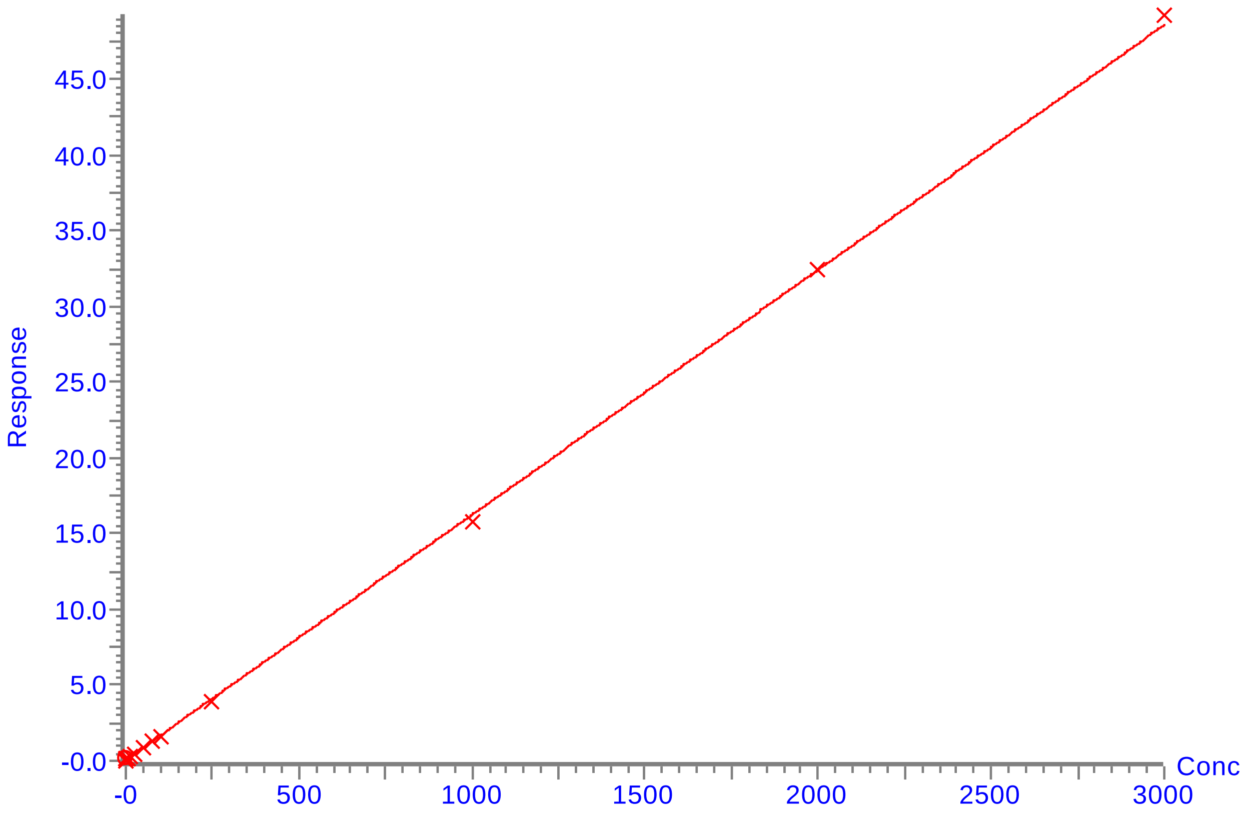

Tridecanoic acid					
	Std Conc (ng/mL)	Response	Conc.	%Dev	S/N
1	0.5	0.069	0.5	6.3	0.5
2	1	0.078	1.1	5.9	1
3	2.5	0.101	2.5	0.2	2.5
4	7.5	0.173	6.9	-7.7	7.5
5	10	0.224	10.1	0.9	10
6	50	0.85	48.8	-2.4	50
7	100	1.654	98.5	-1.5	100
8	250	3.909	237.9	-4.8	250
9	1000	15.831	975	-2.5	1000
10	2000	32.369	1997.6	-0.1	2000
11	3000	49.23	3040	1.3	3000

Compound name: 2/3-Hydroxy Dodecanoic acid
Correlation coefficient: r = 0.999673, r^2 = 0.999346
Calibration curve: 0.00594629 * x + 0.000476821
Response type: Internal Std (Ref 2), Area * (IS Conc. / IS Area)
Curve type: Linear, Origin: Exclude, Weighting: 1/x, Axis trans: None

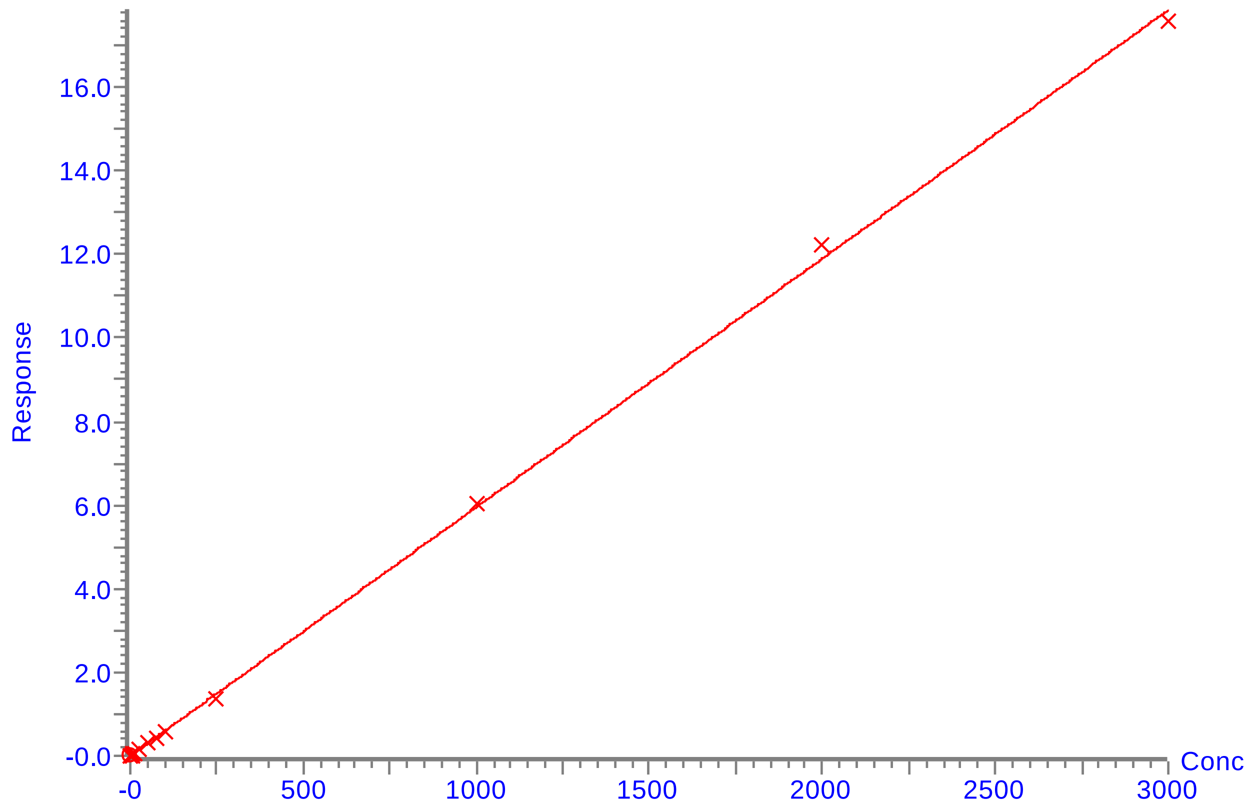

2/3-Hydroxy Dodecanoic acid					
	Std Conc (ng/mL)	Response	Conc.	%Dev	S/N
1	5	0.026	4.3	-13.5	75.201
2	7.5	0.044	7.3	-2.4	128.067
3	10	0.055	9.1	-8.7	126.026
4	25	0.137	23	-8	327.292
5	50	0.296	49.7	-0.6	485.893
6	75	0.422	70.9	-5.4	404.852
7	100	0.57	95.8	-4.2	549.867
8	250	1.381	232.2	-7.1	723.783
9	1000	6.042	1016	1.6	2228.381
10	2000	12.216	2054.2	2.7	2423.403
11	3000	17.601	2959.9	-1.3	1049.675

Compound name: Myristic acid
Correlation coefficient: r = 0.997841, r^2 = 0.995686
Calibration curve: 0.0156622 * x + 0.0735729
Response type: Internal Std (Ref 2), Area * (IS Conc. / IS Area)
Curve type: Linear, Origin: Exclude, Weighting: 1/x, Axis trans: None

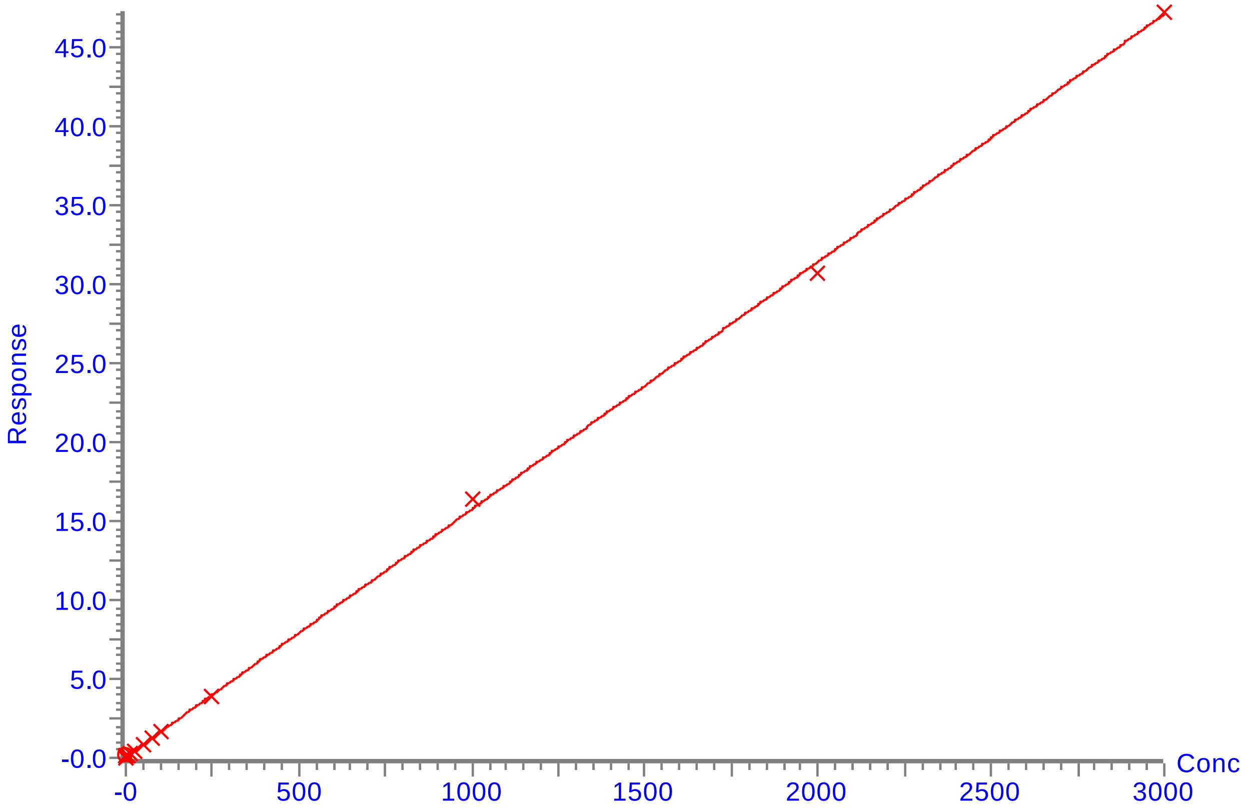

Myristic acid					
	Std Conc (ng/mL)	Response	Conc.	%Dev	S/N
1	1	0.09	1.1	5	209.817
2	5	0.154	5.1	2.8	310.251
3	10	0.238	10.5	4.9	565.674
4	25	0.466	25	0.1	641.044
5	50	0.879	51.4	2.8	1210.16
6	75	1.24	74.5	-0.7	1756.053
7	100	1.689	103.1	3.1	2051.857
8	250	3.902	244.5	-2.2	2207.526
9	1000	16.332	1038.1	3.8	4383.967
10	2000	30.673	1953.7	-2.3	4971.424
11	3000	47.194	3008.5	0.3	4167.69

Compound name: Dodecanedioic acid
Correlation coefficient: r = 0.999513, r^2 = 0.999026
Calibration curve: 0.00201398 * x + 0.0047634
Response type: Internal Std (Ref 2), Area * (IS Conc. / IS Area)
Curve type: Linear, Origin: Exclude, Weighting: 1/x, Axis trans: None

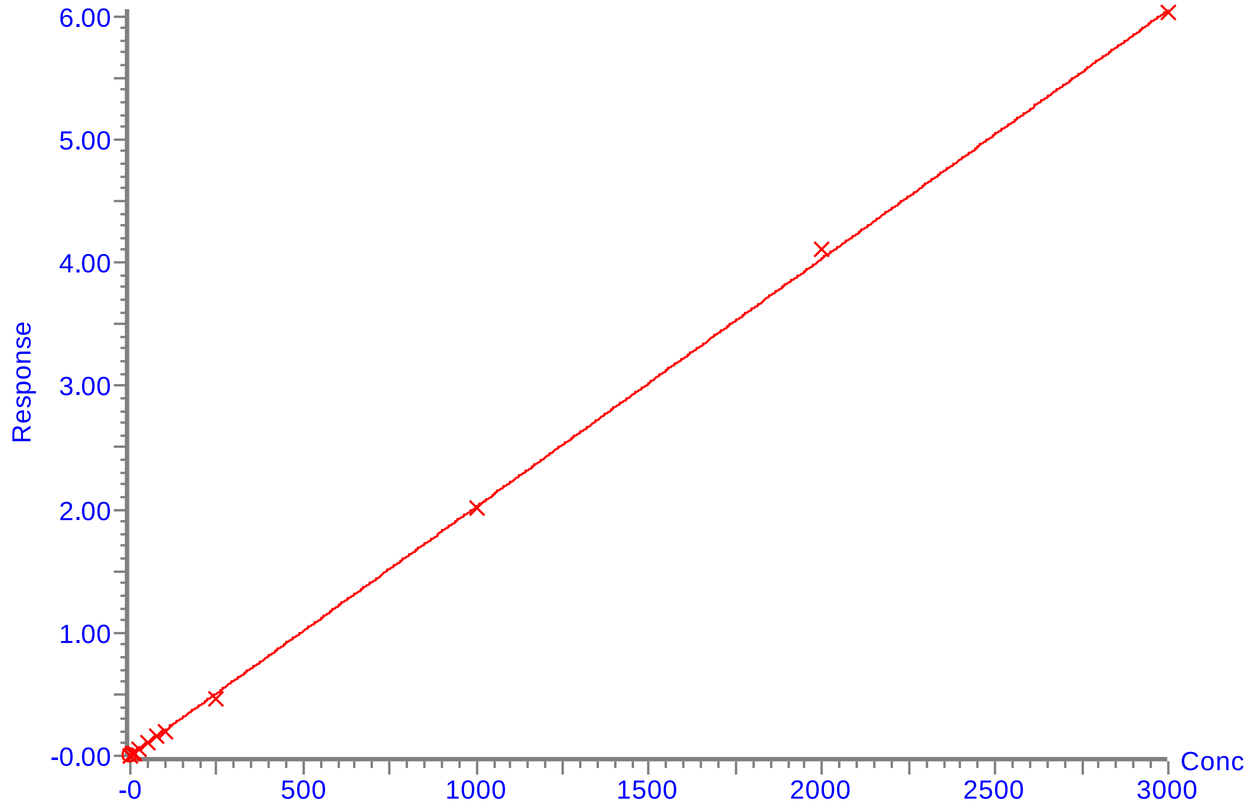

Dodecanedioic acid					
	Std Conc (ng/mL)	Response	Conc.	%Dev	S/N
1	1	0.007	1	1.6	8.145
2	5	0.015	5.1	1.7	16.362
3	7.5	0.02	7.5	-0.5	13.32
4	10	0.025	10.2	2.2	34.392
5	50	0.105	49.6	-0.7	84.76
6	100	0.194	94	-6	124.554
7	250	0.469	230.6	-7.8	119.77
8	1000	2.012	996.4	-0.4	772.149
9	2000	4.117	2041.7	2.1	693.657
10	3000	6.026	2989.6	-0.3	342.897

Compound name: Hydroxy Myristic acid
Correlation coefficient: r = 0.995931, r^2 = 0.991878
Calibration curve: 0.0183036 * x + 0.0072617
Response type: Internal Std (Ref 2), Area * (IS Conc. / IS Area)
Curve type: Linear, Origin: Exclude, Weighting: 1/x, Axis trans: None

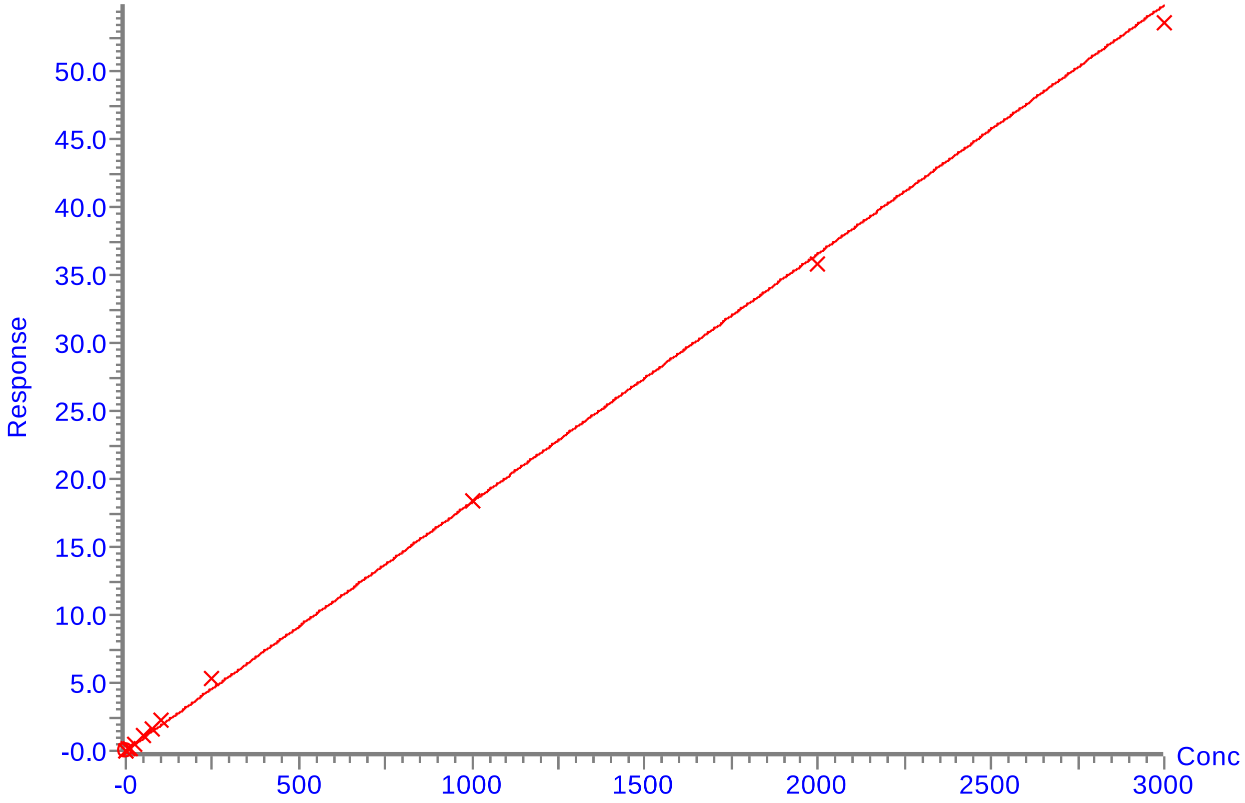

Hydroxy Myristic acid					
	Std Conc (ng/mL)	Response	Conc.	%Dev	S/N
1	5	0.115	5.9	18.2	187.74
2	7.5	0.168	8.8	17.4	270.668
3	10	0.238	12.6	26.1	623.022
4	25	0.553	29.8	19.3	1124.301
5	50	1.103	59.9	19.7	957.243
6	75	1.677	91.2	21.6	1710.829
7	100	2.255	122.8	22.8	2215.766
8	250	5.388	294	17.6	3260.147
9	1000	18.475	1008.9	0.9	1589.311
10	2000	35.864	1959	-2.1	4322.782
11	3000	53.666	2931.6	-2.3	2639.124

Compound name: Pentadecanoic acid
Correlation coefficient: r = 0.998564, r^2 = 0.997131
Calibration curve: 0.0134157 * x + 0.091921
Response type: Internal Std (Ref 2), Area * (IS Conc. / IS Area)
Curve type: Linear, Origin: Exclude, Weighting: 1/x, Axis trans: None

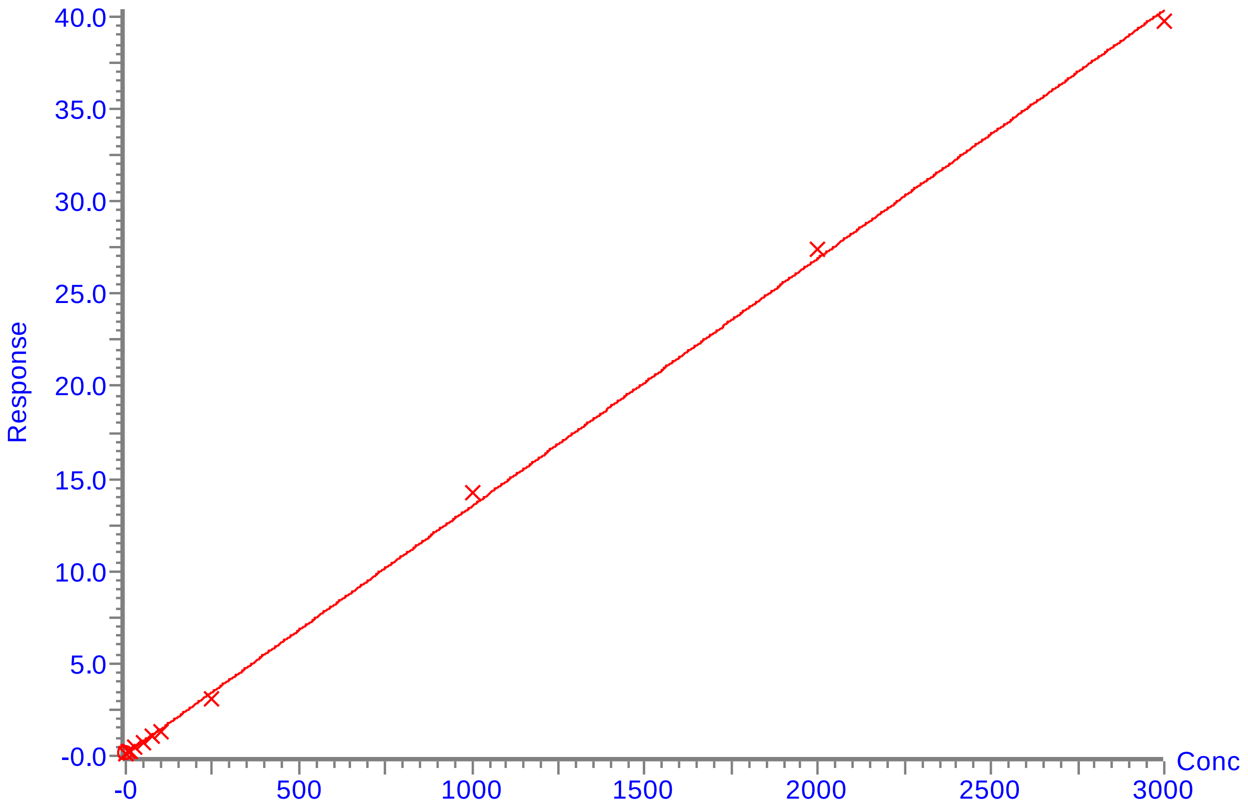

Pentadecanoic acid					
	Std Conc (ng/mL)	Response	Conc.	%Dev	S/N
1	0.5	0.099	0.5	5.4	363.658
2	1	0.104	0.9	-6.5	358.519
3	2.5	0.15	4.4	74.5	218.5
4	10	0.209	8.7	-12.9	706.547
5	25	0.42	24.5	-2.2	1084.423
6	50	0.733	47.8	-4.4	1728.278
7	75	1.047	71.2	-5.1	2058.311
8	100	1.341	93.1	-6.9	2012.386
9	250	3.076	222.4	-11	2535.801
10	1000	14.194	1051.1	5.1	8344.425
11	2000	27.41	2036.3	1.8	10764.43
12	3000	39.693	2951.9	-1.6	13692.682

Compound name: Palmitoleic acid
Correlation coefficient: r = 0.998733, r^2 = 0.997467
Calibration curve: 0.00426706 * x + 0.00263467
Response type: Internal Std (Ref 2), Area * (IS Conc. / IS Area)
Curve type: Linear, Origin: Exclude, Weighting: 1/x, Axis trans: None

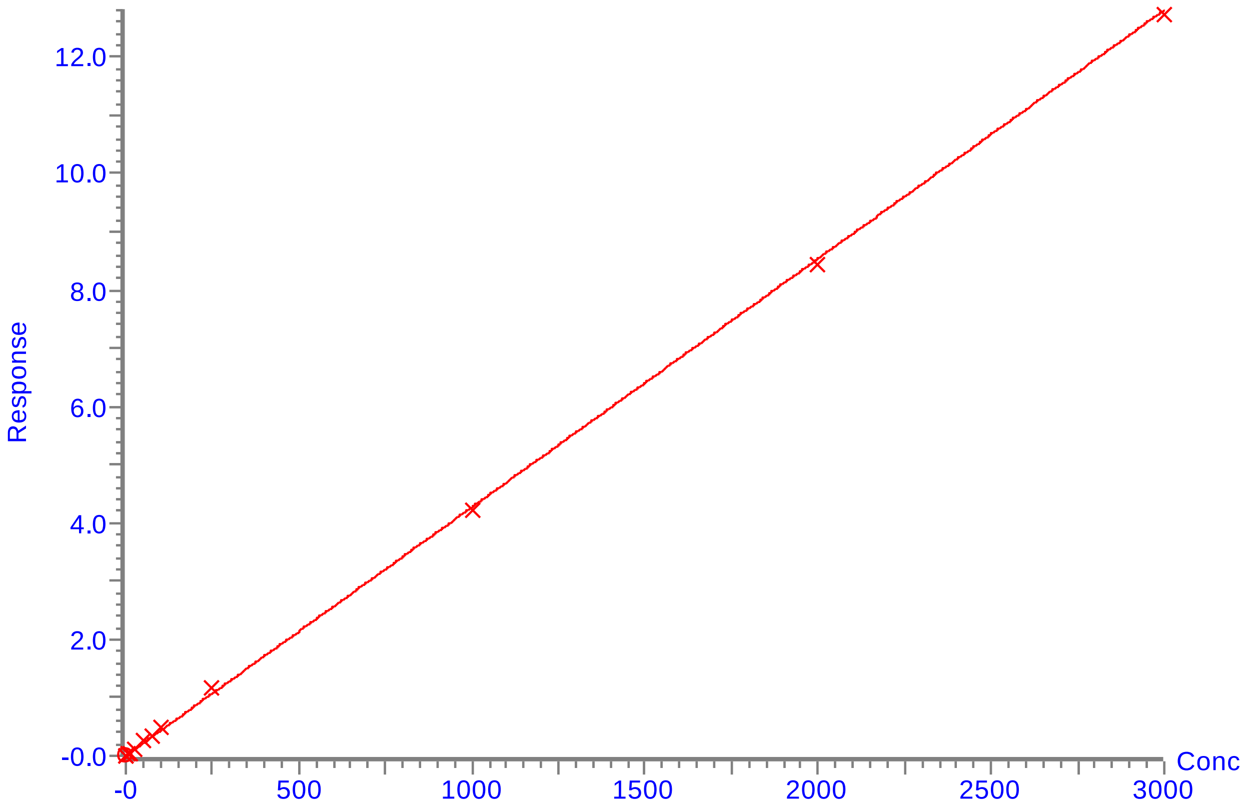

Palmitoleic acid					
	Std Conc (ng/mL)	Response	Conc.	%Dev	S/N
1	1	0.007	1	-1.1	11.876
2	5	0.024	5.1	1	88.446
3	7.5	0.035	7.7	2.5	106.638
4	10	0.041	9	-10.5	148.634
5	25	0.113	25.9	3.7	231.977
6	50	0.259	60	20	707.551
7	75	0.353	82.1	9.4	671.369
8	100	0.474	110.4	10.4	795.536
9	250	1.182	276.4	10.6	1471.554
10	1000	4.205	984.9	-1.5	3420.18
11	2000	8.452	1980.1	-1	2702.507
12	3000	12.728	2982.3	-0.6	2775.838

Compound name: Heptadecenoic acid
Correlation coefficient: r = 0.998315, r^2 = 0.996633
Calibration curve: 0.0137598 * x + 0.0026414
Response type: Internal Std (Ref 2), Area * (IS Conc. / IS Area)
Curve type: Linear, Origin: Exclude, Weighting: 1/x, Axis trans: None

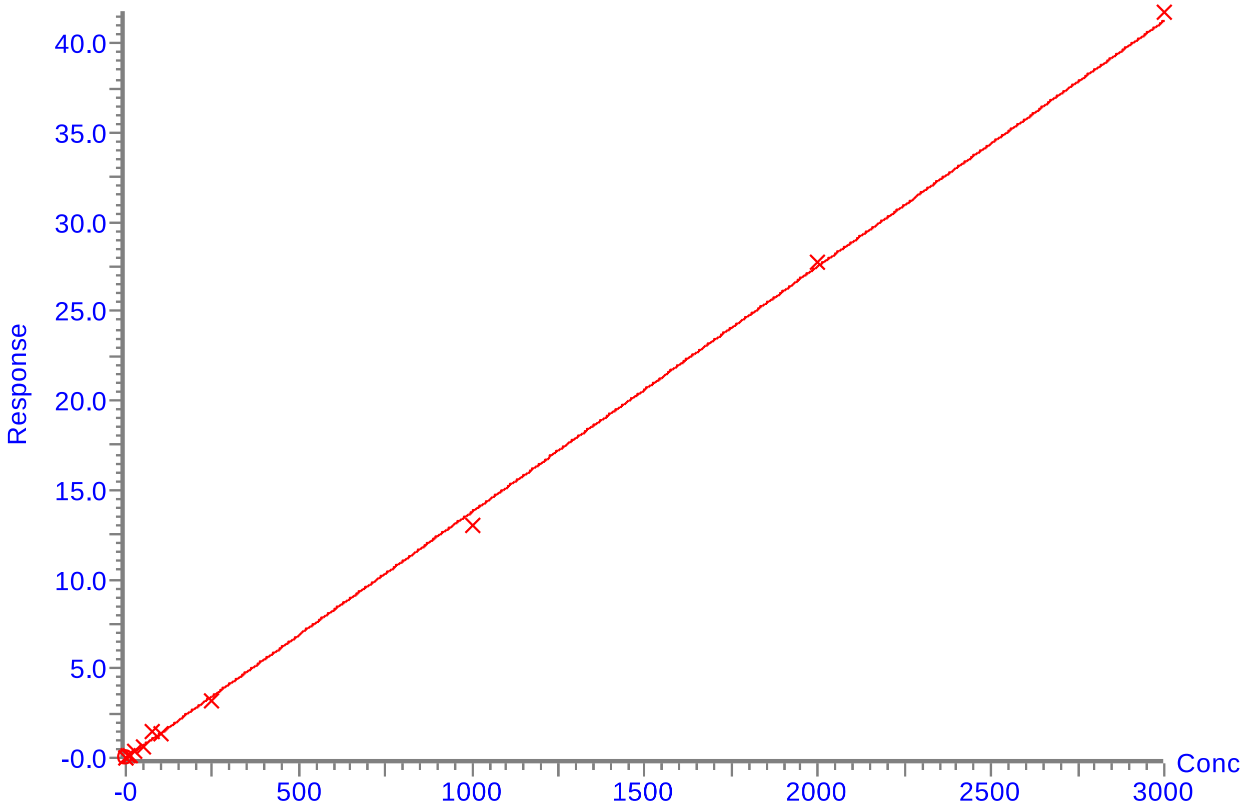

Heptadecenoic acid					
	Std Conc (ng/mL)	Response	Conc.	%Dev	S/N
1	5	0.075	5.3	5.1	102.27
2	7.5	0.102	7.2	-3.4	98.516
3	10	0.132	9.4	-6	229.273
4	25	0.341	24.6	-1.6	295.633
5	50	0.583	42.2	-15.7	450.271
6	75	1.472	106.8	42.4	1026.598
7	100	1.333	96.7	-3.3	923.408
8	250	3.166	229.9	-8	1321.608
9	1000	13.014	945.6	-5.4	3961.499
10	2000	27.813	2021.1	1.1	3366.878
11	3000	41.752	3034.2	1.1	3187.396

Compound name: Heptadecanoic acid
Correlation coefficient: r = 0.998497, r^2 = 0.996997
Calibration curve: 0.0140287 * x + 0.0761505
Response type: Internal Std (Ref 2), Area * (IS Conc. / IS Area)
Curve type: Linear, Origin: Exclude, Weighting: 1/x, Axis trans: None

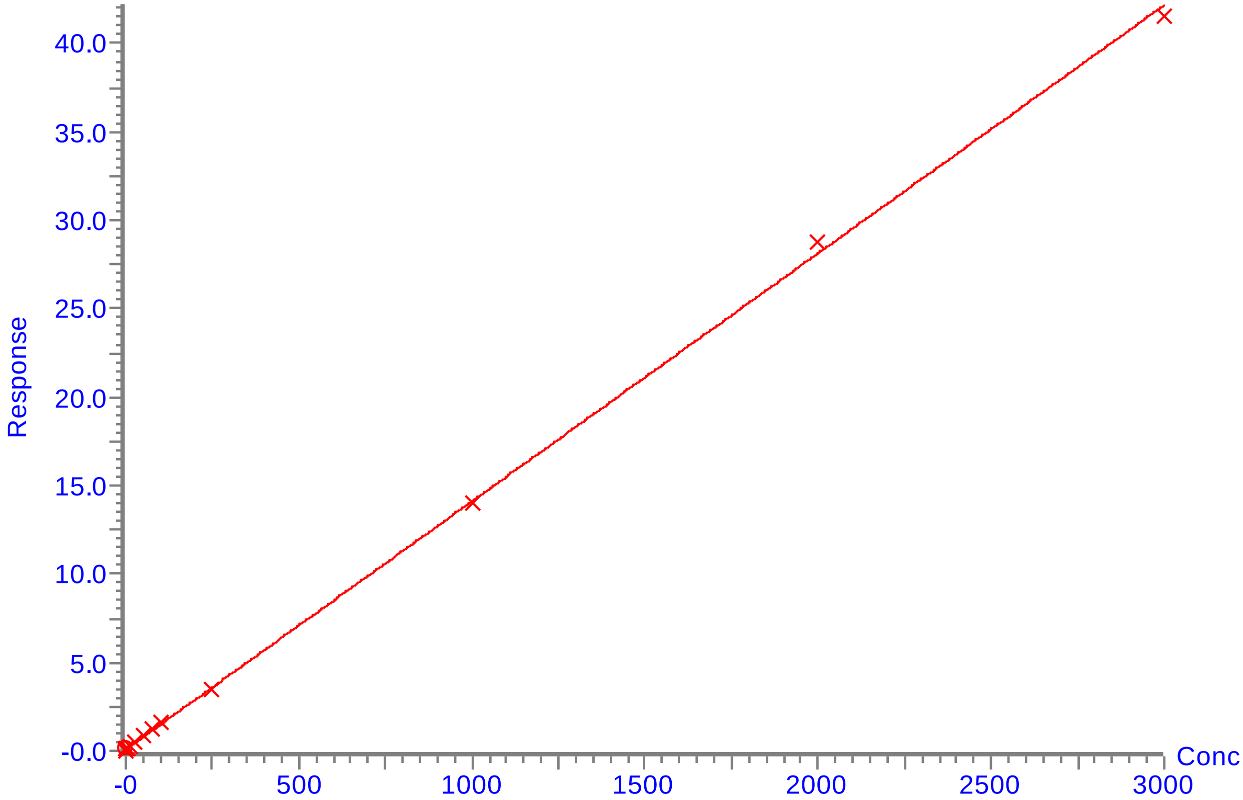

Heptadecanoic acid					
	Std Conc (ng/mL)	Response	Conc.	%Dev	S/N
1	1	0.09	1	-1.2	276.607
2	5	0.152	5.4	8	709.384
3	10	0.228	10.9	8.5	587.996
4	50	0.816	52.7	5.5	2298.744
5	75	1.178	78.6	4.8	2995.649
6	100	1.565	106.1	6.1	2138.893
7	250	3.507	244.6	-2.2	3574.939
8	1000	14.073	997.8	-0.2	11299.334
9	2000	28.737	2043.1	2.2	10419.188
10	3000	41.511	2953.6	-1.5	12254.106

Compound name: Hydroxy Palmitic acid
Correlation coefficient: r = 0.998731, r^2 = 0.997463
Calibration curve: 0.0110666 * x + 0.00292661
Response type: Internal Std (Ref 2), Area * (IS Conc. / IS Area)
Curve type: Linear, Origin: Exclude, Weighting: 1/x, Axis trans: None

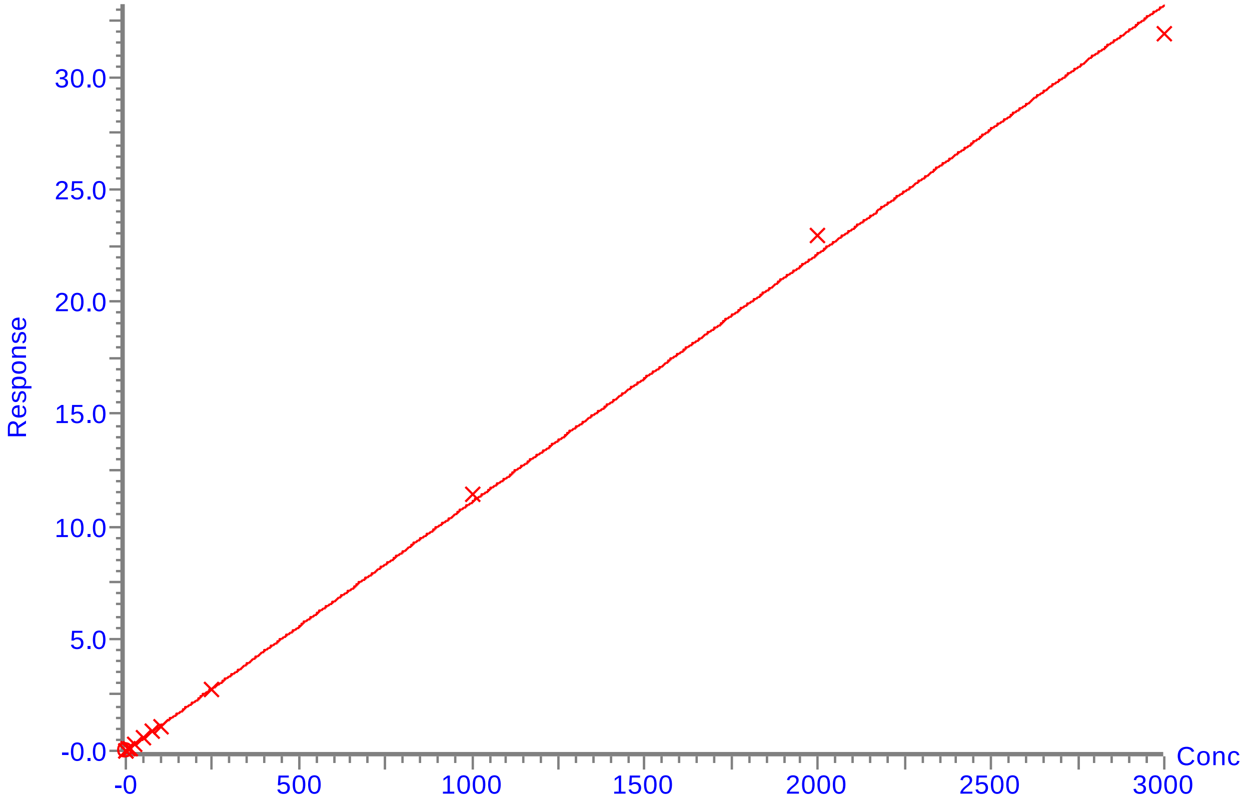

Hydroxy palmitic acid					
	Std Conc (ng/mL)	Response	Conc.	%Dev	S/N
1	5	0.063	5.4	8.8	104.988
2	7.5	0.09	7.9	5	215.196
3	10	0.116	10.3	2.5	300.859
4	25	0.279	24.9	-0.3	547.866
5	50	0.612	55.1	10.2	693.024
6	75	0.903	81.3	8.4	790.14
7	100	1.078	97.1	-2.9	989.615
8	250	2.756	248.8	-0.5	1798.74
9	1000	11.432	1032.7	3.3	4180.396
10	2000	22.995	2077.6	3.9	3293.208
11	3000	31.927	2884.7	-3.8	2393.853

Compound name: Linoeliaidic acid
Correlation coefficient: r = 0.999447, r^2 = 0.998895
Calibration curve: 0.00110851 * x + -0.000189746
Response type: Internal Std (Ref 2), Area * (IS Conc. / IS Area)
Curve type: Linear, Origin: Exclude, Weighting: 1/x, Axis trans: None

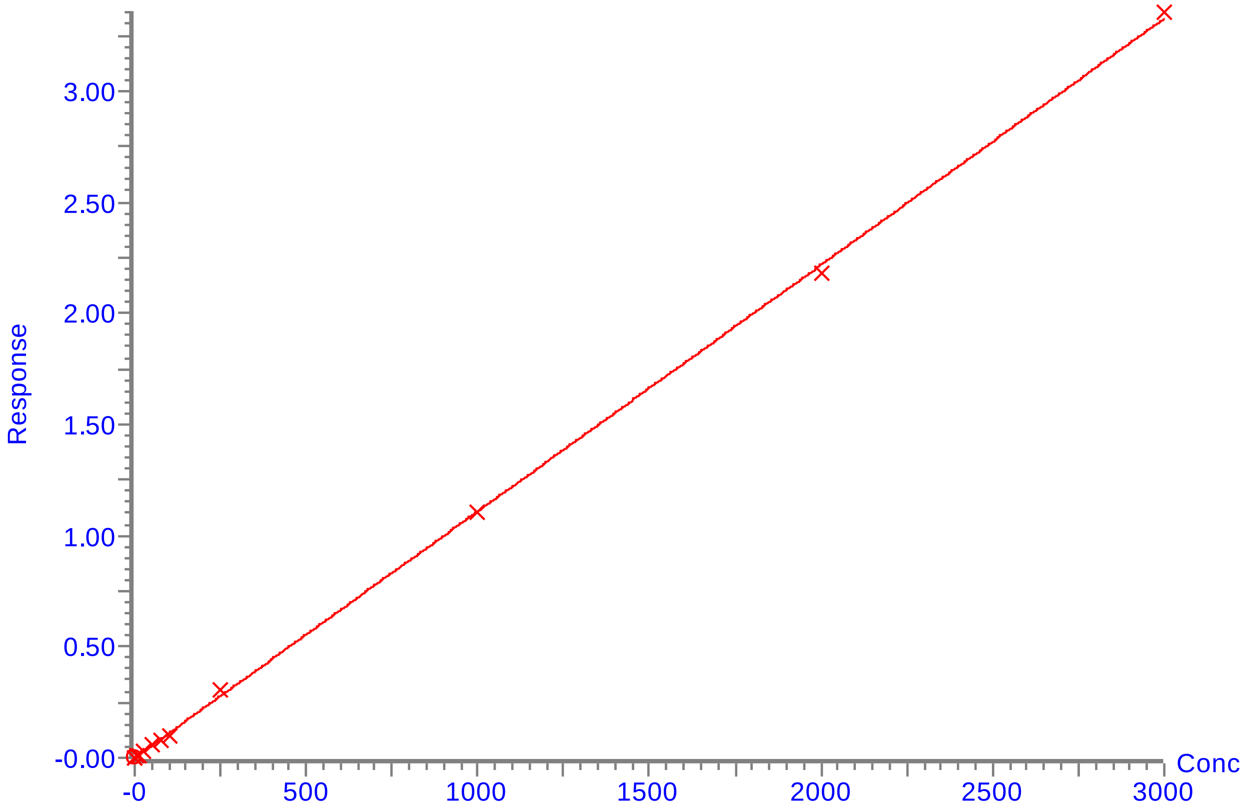

Linoelaidic acid					
	Std Conc (ng/mL)	Response	Conc.	%Dev	S/N
1	5	0.005	5.1	2.6	7.934
2	7.5	0.008	7.6	1	47529
3	10	0.012	10.7	6.6	29.364
4	25	0.026	23.9	-4.3	51.805
5	50	0.056	51	2	174.215
6	75	0.077	69.8	-6.9	58.605
7	100	0.098	88.3	-11.7	105.284
8	250	0.306	276.6	10.6	122.065
9	1000	1.107	998.9	-0.1	343.617
10	2000	2.18	1966.6	-1.7	316.201
11	3000	3.354	3025.6	0.9	196.14

Compound name: 12 (Z)-Conjugated Linoleic acid
Correlation coefficient: r = 0.998756, r^2 = 0.997513
Calibration curve: 0.00102806 * x + 0.000569885
Response type: Internal Std (Ref 2), Area * (IS Conc. / IS Area)
Curve type: Linear, Origin: Exclude, Weighting: 1/x, Axis trans: None

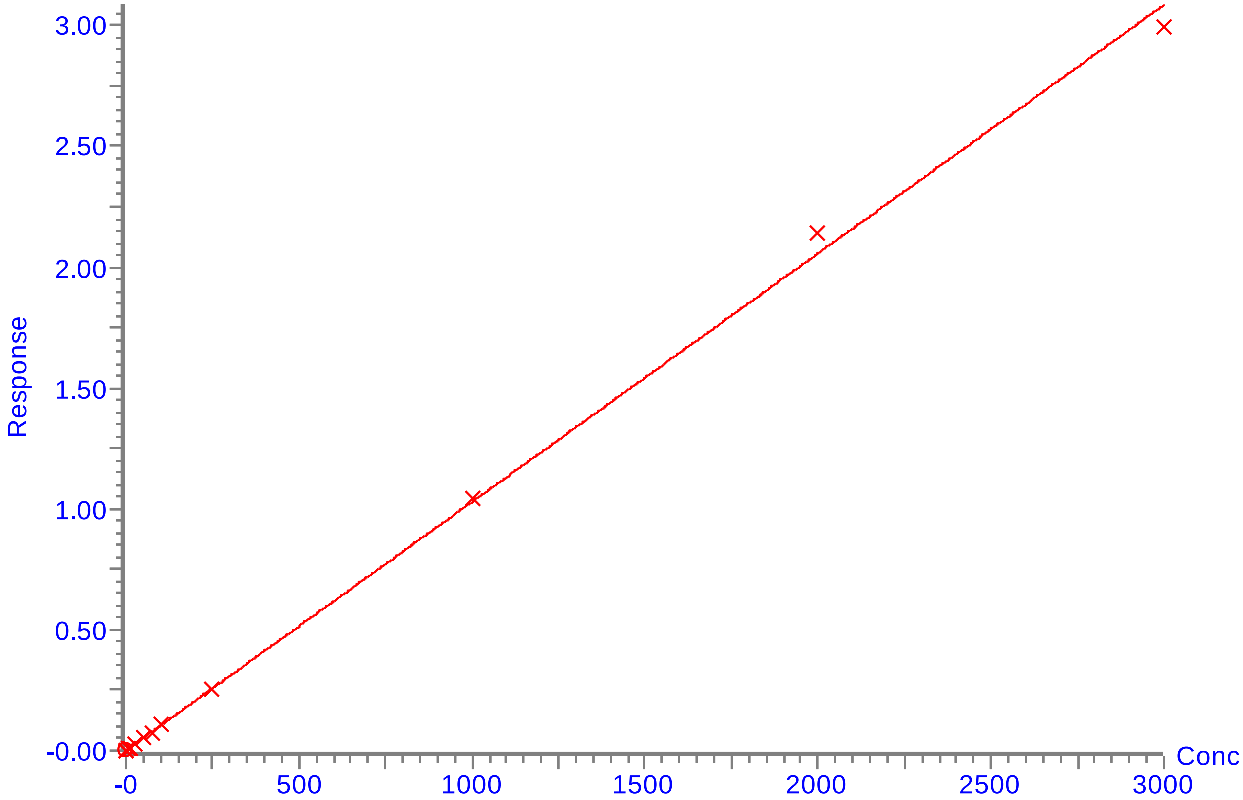

12 (Z)-Conjugated Linoleic acid					
	Std Conc (ng/mL)	Response	Conc.	%Dev	S/N
1	5	0.006	5.1	1.8	20.342
2	7.5	0.009	7.9	4.9	49.527
3	10	0.01	9.4	-5.6	45.033
4	25	0.026	24.5	-1.9	130.461
5	50	0.055	52.8	5.6	213.843
6	75	0.077	74.1	-1.1	187.888
7	100	0.108	104.2	4.2	273.089
8	250	0.254	246.3	-1.5	346.345
9	1000	1.044	1014.6	1.5	1455.32
10	2000	2.138	2078.9	3.9	1053.672
11	3000	2.991	2908.5	-3	657.434

Compound name: Oleic acid
Correlation coefficient: r = 0.999328, r^2 = 0.998656
Calibration curve: 0.007765 * x + 0.014601
Response type: Internal Std (Ref 2), Area * (IS Conc. / IS Area)
Curve type: Linear, Origin: Exclude, Weighting: 1/x, Axis trans: None

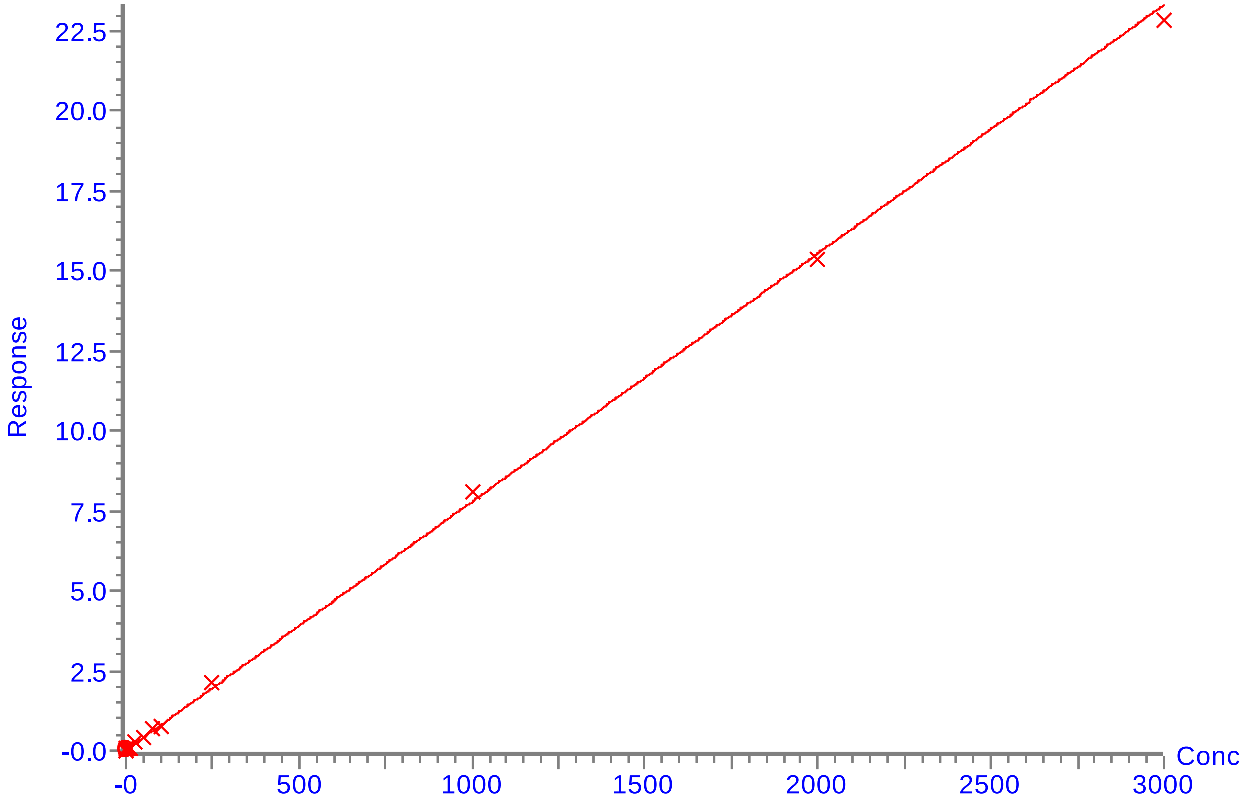

Oleic acid/ Elaidic acid					
	Std Conc (ng/mL)	Response	Conc.	%Dev	S/N
1	0.5	0.019	0.5	8.9	54.504
2	2.5	0.033	2.3	-7.4	107.855
3	5	0.064	6.4	27.6	208.393
4	10	0.099	10.9	8.6	343.074
5	50	0.434	54	7.9	1273.842
6	75	0.677	85.3	13.8	1560.042
7	100	0.773	97.7	-2.3	2213.919
8	250	2.125	271.8	8.7	2916.637
9	1000	8.097	1040.9	4.1	9092.642
10	2000	15.362	1976.5	-1.2	8931.033
11	3000	22.846	2940.3	-2	4990.092

Compound name: Stearic acid
Correlation coefficient: r = 0.998386, r^2 = 0.996774
Calibration curve: 0.0115371 * x + 0.118623
Response type: Internal Std (Ref 2), Area * (IS Conc. / IS Area)
Curve type: Linear, Origin: Exclude, Weighting: 1/x, Axis trans: None

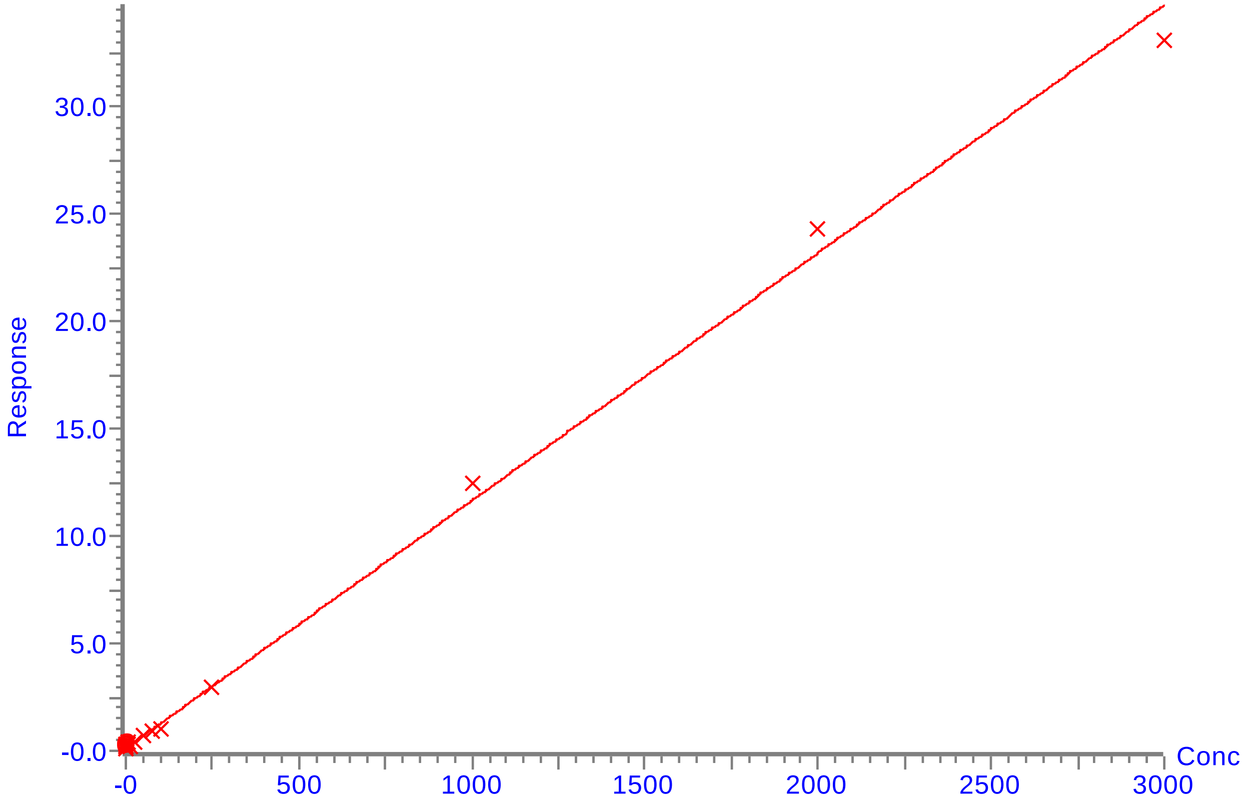

Stearic acid					
	Std Conc (ng/mL)	Response	Conc.	%Dev	S/N
1	10	0.227	9.4	-6.1	679.262
2	25	0.458	29.4	17.5	1529.29
3	50	0.717	51.9	3.7	2615.256
4	75	0.964	73.3	-2.3	2451.263
5	100	1.022	78.3	-21.7	2472.615
6	250	2.967	246.9	-1.2	2744.846
7	1000	12.422	1066.4	6.6	12633.493
8	2000	24.27	2093.4	4.7	12328.398
9	3000	33.125	2860.9	-4.6	8414.797

Compound name: 9-Cis Retinoic acid
Correlation coefficient: r = 0.999836, r^2 = 0.999673
Calibration curve: 0.0137313 * x + 0.00204714
Response type: Internal Std (Ref 2), Area * (IS Conc. / IS Area)
Curve type: Linear, Origin: Exclude, Weighting: 1/x, Axis trans: None

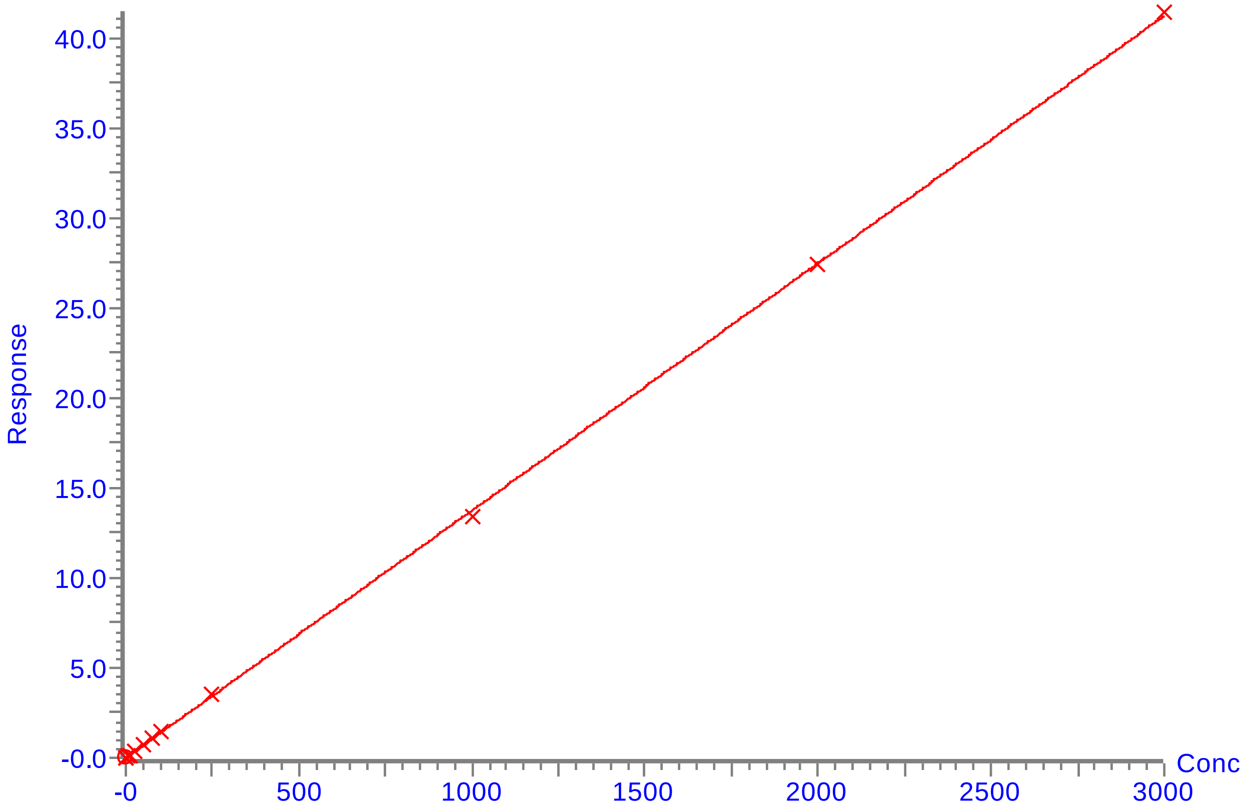

ATRA/9-cis RA*					
	Std Conc (ng/mL)	Response	Conc.	%Dev	S/N
1	5	0.076	5.4	7.6	328.88
2	7.5	0.105	7.5	-0.4	593.677
3	10	0.133	9.5	-4.9	700.496
4	25	0.343	24.8	-0.8	715.793
5	50	0.753	54.7	9.3	2072.766
6	75	1.059	77	2.6	2585.369
7	100	1.47	106.9	6.9	3202.88
8	250	3.536	257.3	2.9	4272.171
9	1000	13.351	972.2	-2.8	4385.433
10	2000	27.356	1992.1	-0.4	5731.976
11	3000	41.405	3015.2	0.5	7656.861

Compound name: Eicosapentoic acid
Correlation coefficient: r = 0.999242, r^2 = 0.998485
Calibration curve: 0.00061325 * x + 0.00182942
Response type: Internal Std (Ref 2), Area * (IS Conc. / IS Area)
Curve type: Linear, Origin: Exclude, Weighting: 1/x, Axis trans: None

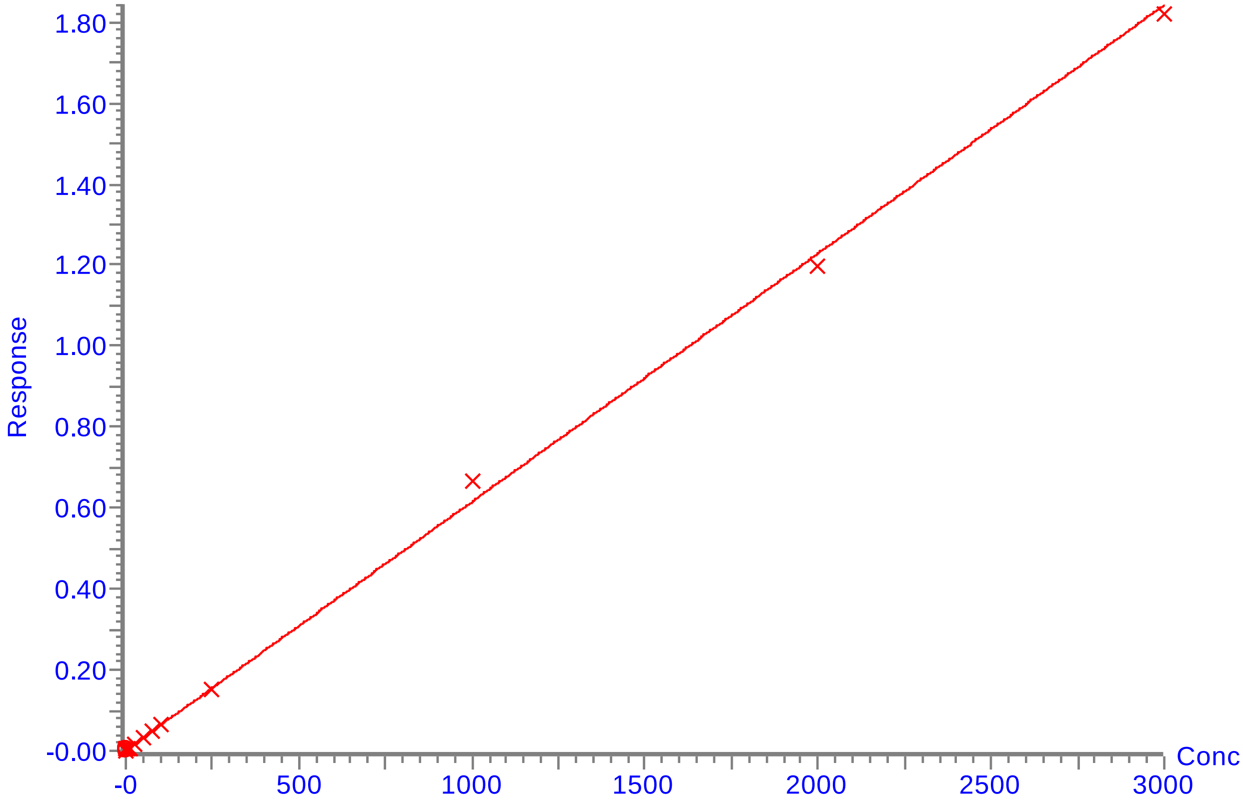

Eicosapentoic acid					
	Std Conc (ng/mL)	Response	Conc.	%Dev	S/N
1	5	0.005	4.7	-5.2	11.69
2	7.5	0.006	7.3	-2.5	18.975
3	10	0.008	9.5	-5.3	22.926
4	50	0.034	52.8	5.6	70.393
5	100	0.065	103.1	3.1	94.996
6	250	0.153	247	-1.2	240.832
7	1000	0.668	1087.1	8.7	501.716
8	2000	1.198	1950.7	-2.5	573.391
9	3000	1.817	2960.6	-1.3	766.304

Compound name: Arachidonic acid
Correlation coefficient: r = 0.997152, r^2 = 0.994312
Calibration curve: 0.000244034 * x + 0.0023204
Response type: Internal Std (Ref 2), Area * (IS Conc. / IS Area)
Curve type: Linear, Origin: Exclude, Weighting: 1/x, Axis trans: None

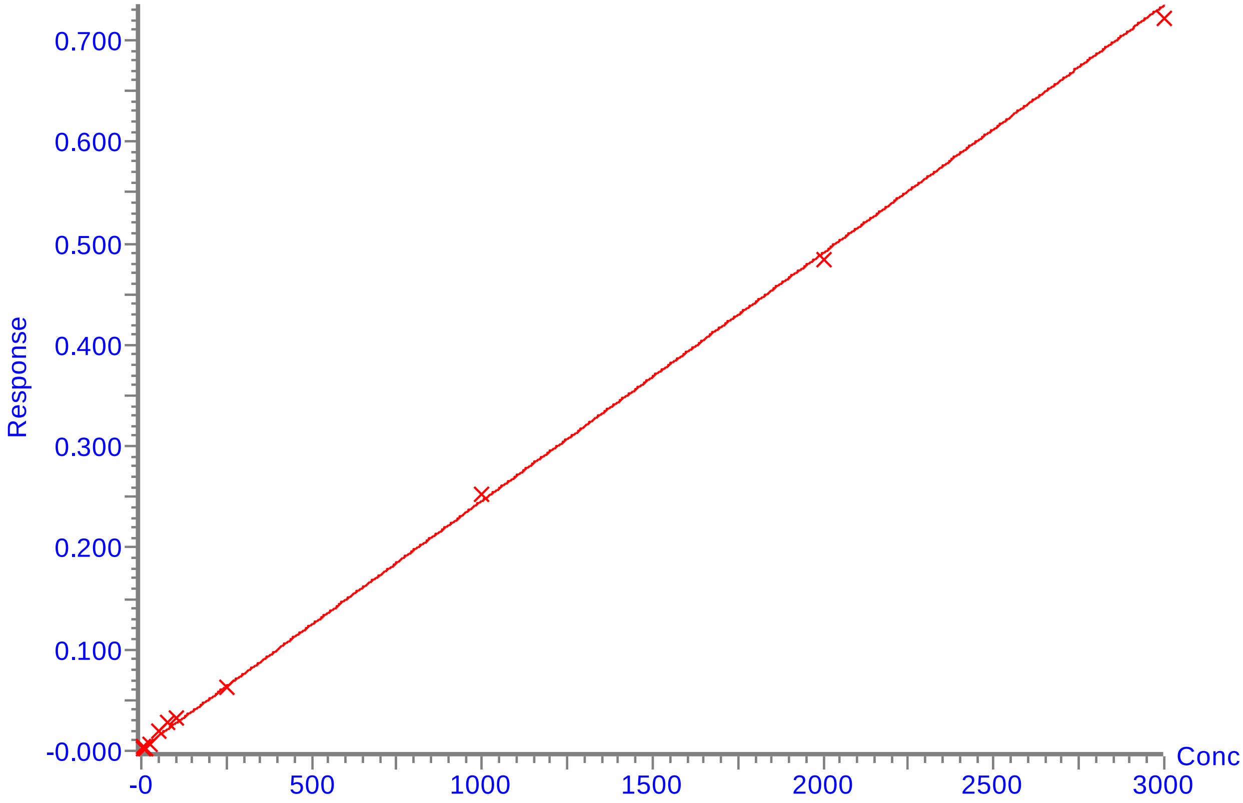

Arachidonic acid					
	Std Conc (ng/mL)	Response	Conc.	%Dev	S/N
1	5	0.004	6.4	28.3	19.804
2	25	0.007	20.5	-17.9	46.666
3	100	0.033	125	25	86.985
4	250	0.062	246	-1.6	215.079
5	1000	0.253	1026.5	2.7	334.591
6	2000	0.483	1970.7	-1.5	426.774
7	3000	0.721	2944.6	-1.8	382.969

Compound name: Cis-11-Eicosenoic acid
Correlation coefficient: r = 0.998829, r^2 = 0.997660
Calibration curve: 0.00606426 * x + 0.0371558
Response type: Internal Std (Ref 2), Area * (IS Conc. / IS Area)
Curve type: Linear, Origin: Exclude, Weighting: 1/x, Axis trans: None

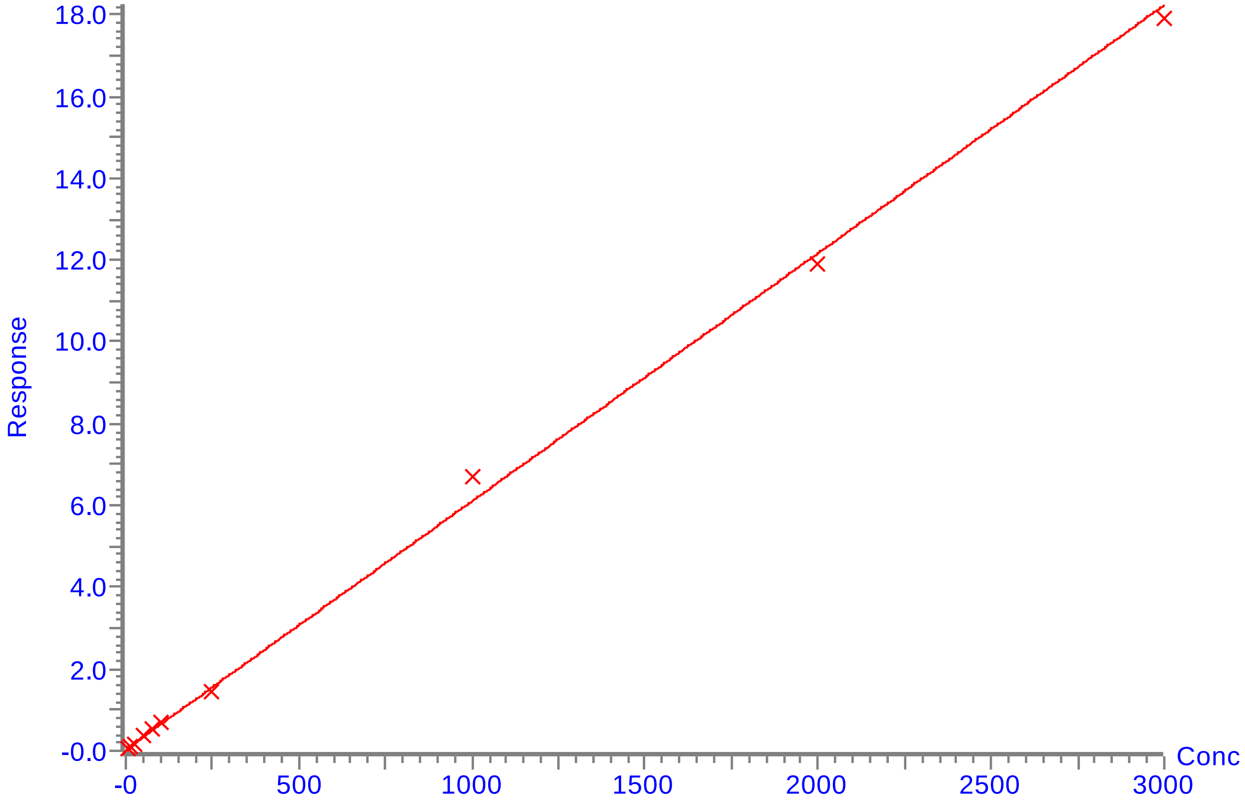

Cis-11-Eicosenoic acid					
	Std Conc (ng/mL)	Response	Conc.	%Dev	S/N
1	5	0.062	4.1	-18.8	36.156
2	10	0.099	10.2	2.5	257.704
3	50	0.372	55.2	10.5	393.266
4	75	0.523	80.1	6.7	871.83
5	100	0.687	107.2	7.2	598.323
6	250	1.45	233	-6.8	689.51
7	1000	6.708	1099.9	10	2895.52
8	2000	11.891	1954.7	-2.3	3528.598
9	3000	17.905	2946.5	-1.8	2713.495

Compound name: Docosahexanoic acid
Correlation coefficient: r = 0.999459, r^2 = 0.998918
Calibration curve: 0.00330324 * x + 0.0268456
Response type: Internal Std (Ref 2), Area * (IS Conc. / IS Area)
Curve type: Linear, Origin: Exclude, Weighting: 1/x, Axis trans: None

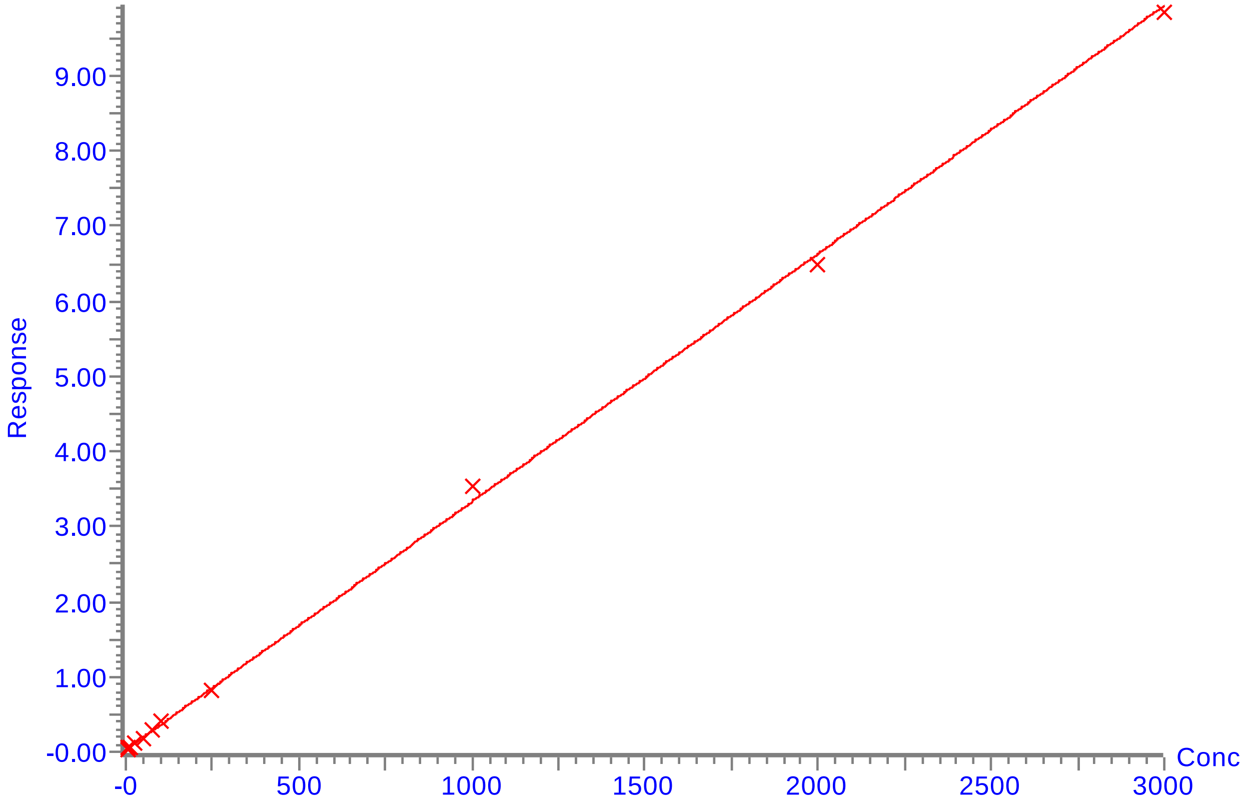

Docosahexanoic acid					
	Std Conc (ng/mL)	Response	Conc.	%Dev	S/N
1	5	0.041	4.3	-14.4	122.235
2	7.5	0.051	7.3	-3.3	133.223
3	10	0.06	10	-0.3	175.761
4	25	0.109	24.8	-0.9	178.293
5	50	0.19	49.3	-1.3	409.613
6	100	0.395	111.6	11.6	618.861
7	250	0.826	242	-3.2	1181.065
8	1000	3.523	1058.3	5.8	3092.259
9	2000	6.491	1957	-2.1	3529.447
10	3000	9.858	2976.3	-0.8	2172.369

Compound name: Heneicosanoic acid
Correlation coefficient: r = 0.999456, r^2 = 0.998913
Calibration curve: 0.00305218 * x + 0.0109285
Response type: Internal Std (Ref 2), Area * (IS Conc. / IS Area)
Curve type: Linear, Origin: Exclude, Weighting: 1/x, Axis trans: None

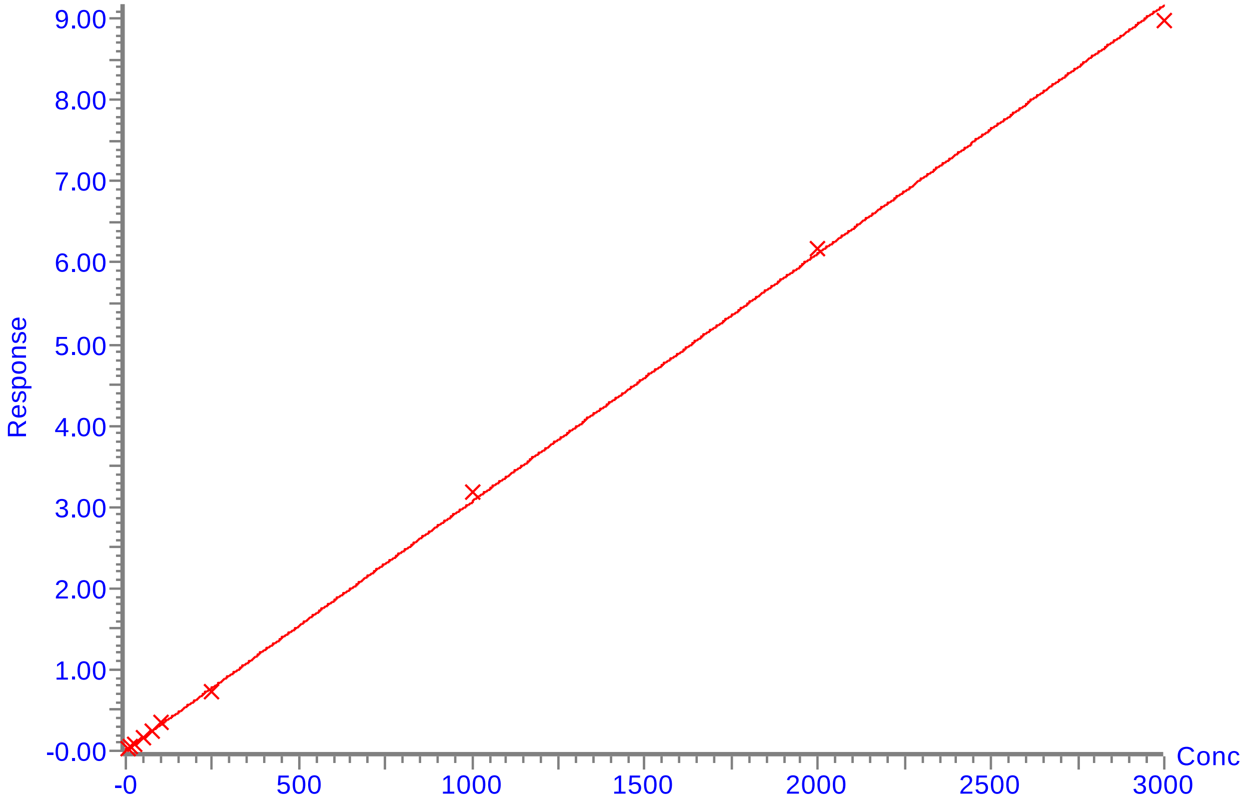

Heneicosanoic acid					
	Std Conc (ng/mL)	Response	Conc.	%Dev	S/N
1	5	0.023	3.9	-21.1	118.01
2	7.5	0.031	6.7	-11.3	72.407
3	10	0.044	10.7	6.9	117.077
4	25	0.093	26.9	7.7	242.978
5	50	0.162	49.5	-1	350.94
6	75	0.254	79.8	6.4	615.026
7	100	0.364	115.5	15.5	826.18
8	250	0.73	235.8	-5.7	2347.317
9	1000	3.178	1037.8	3.8	6827.286
10	2000	6.166	2016.7	0.8	9642.639
11	3000	8.982	2939.2	-2	5897.276

Compound name: Erucic acid
Correlation coefficient: r = 0.999371, r^2 = 0.998743
Calibration curve: 0.00769466 * x + 0.0538197
Response type: Internal Std (Ref 2), Area * (IS Conc. / IS Area)
Curve type: Linear, Origin: Exclude, Weighting: 1/x, Axis trans: None

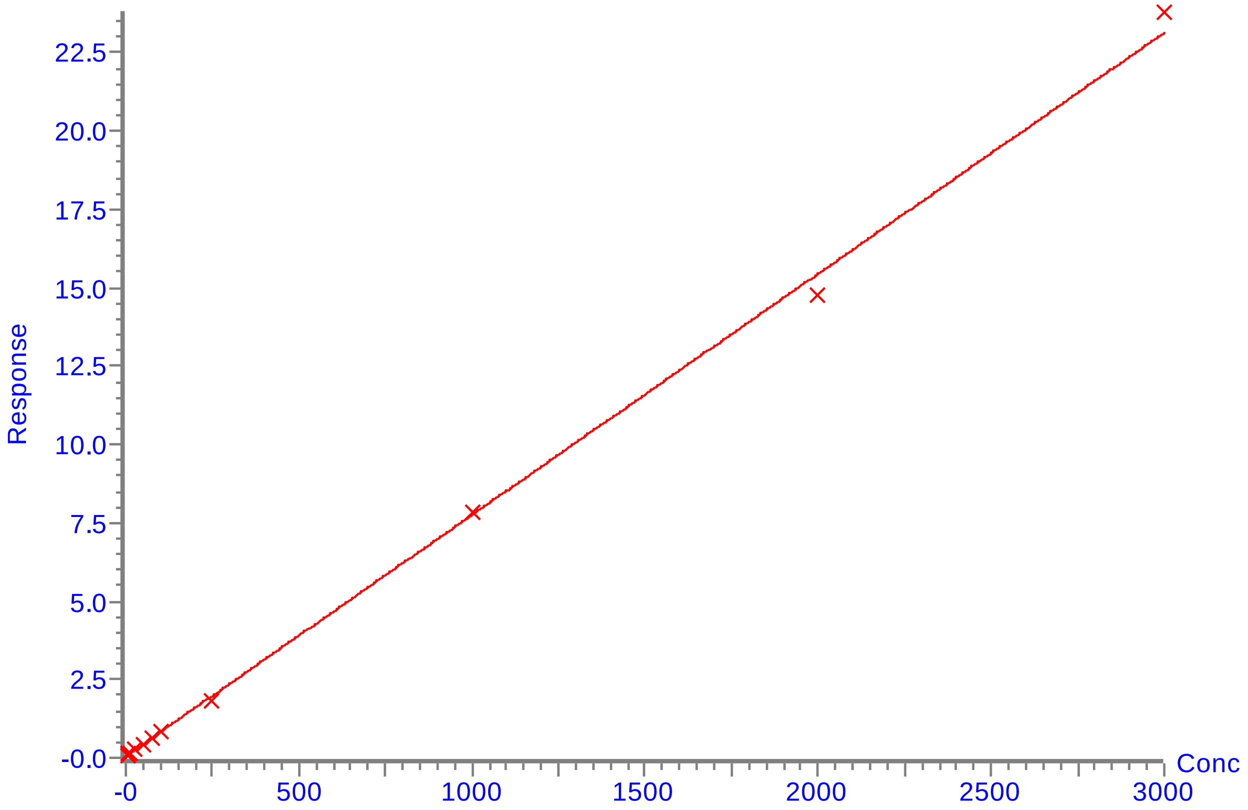

Erucic acid					
	Std Conc (ng/mL)	Response	Conc.	%Dev	S/N
1	5	0.091	4.9	-2.3	558.851
2	7.5	0.11	7.3	-2.5	492.309
3	10	0.126	9.4	-5.5	445.142
4	25	0.272	28.4	13.4	1750.-
5	50	0.44	50.2	0.5	2770.539
6	75	0.636	75.6	0.8	2446.582
7	100	0.846	103	3	5774.891
8	250	1.843	232.6	-7	4391.293
9	1000	7.833	1011	1.1	30413.483
10	2000	14.791	1915.2	-4.2	13979.304
11	3000	23.791	3084.9	2.8	15593.152

Compound name: 2-Phenyl-2propyl-succinic acid
Correlation coefficient: r = 0.999212, r^2 = 0.998424
Calibration curve: 9.82132e-005 * x + 0.00588662
Response type: Internal Std (Ref 2), Area * (IS Conc. / IS Area)
Curve type: Linear, Origin: Exclude, Weighting: 1/x, Axis trans: None

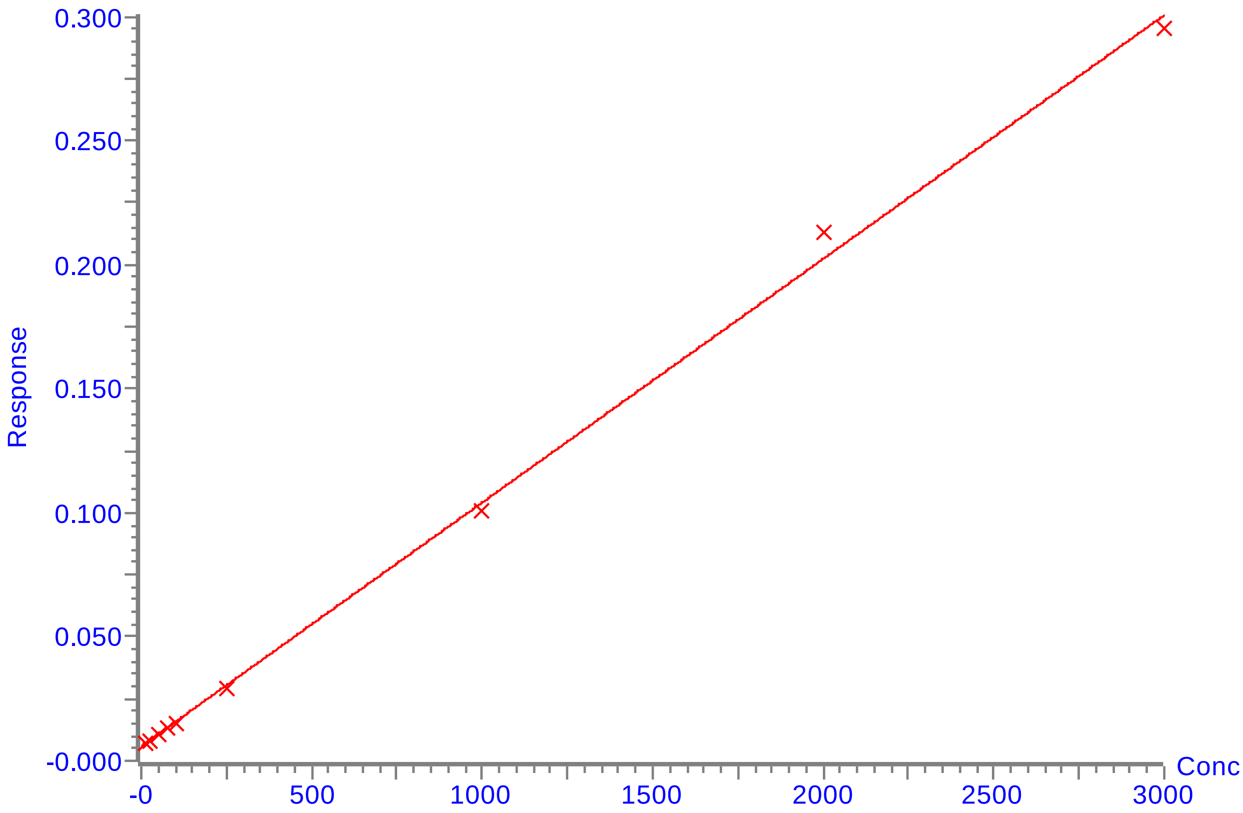

2-Phenyl-2-propyl-succinic acid					
	Std Conc (ng/mL)	Response	Conc.	%Dev	S/N
1	10	0.007	11.7	17.5	39.249
2	25	0.008	23.2	-7.2	198.082
3	50	0.011	51.2	2.4	164.202
4	75	0.013	74.5	-0.7	311.305
5	100	0.015	92.7	-7.3	69.705
6	250	0.029	237.2	-5.1	380.154
7	1000	0.101	968.9	-3.1	1860.709
8	2000	0.213	2108.3	5.4	849.824
9	3000	0.295	2942.2	-1.9	1910.75

Supplementary Table 3. List of metabolites with respective MRM transitions, retention time (RT), limit of detection (LOD), linearity range, single to noise (S/N) ratio and r^2 values.

S. No.	Name of Metabolite	MRM trace	RT (min)	LOD (ng/mL)	Linearity range (ng/mL)	S/N at LOD	r^2 value
1	Glyceric acid	213.26>196.01	0.71	0.25	2.5-3000	1554	0.997
2	Glyoxalic acid	180.913>152.000	4.02	1	5.0-3000	10.6	0.999
3	Propionic acid	181.259>166.060	4.03	0.5	5.0-3000	31	0.998
4	Pyruvic acid	194.929>179	4.16	1	10.0-3000	2589	0.999
5	Alanine	196.273>181.01	4.16	1	5.0-3000	275	0.998
6	2-Oxo butyric acid	209.270>194.035	4.23	0.5	2.5-3000	130	0.993
7	Malonic acid	211.143>152.07	3.92	0.01	1.0-3000	184	0.999
8	Serine	212.270>165.062	3.92	1	5.0-3000	31	0.999
9	4-Methyl-2-oxo-pentanoic acid	223.216>180	4.82	0.01	10.0-5000	1133	0.996
10	Fumaric acid	223.252>165.061	4.82	0.01	10.0-5000	1296	0.999
11	Maleic acid	245.179>182.998	3.61	0.05	10.0-5000	734	0.996
12	Valine	224.33>165.063	4.76	0.05	5.0-3000	291	0.997
13	Succinic acid	225.195>166.01	4.05	0.1	1.0-3000	46	0.999
14	Ureidopropionic acid	238.966>166.036	8.71	5	5.0-3000	51	0.999
15	Oxaloacetic acid	239.238>152.02	6.16	5	50.0-3000	6.7	0.999
16	Aspartic acid	240.36>165.061	4.19	0.1	1.0-3000	56	0.999
17	Malic acid	240.679>179.032	3.96	0.1	1.0-3000	23	0.998
18	Homocysteine	242.00>209.00	4.19	0.1	1.0-3000	65	0.998
19	α-Keto glutaric acid	252.871>152.01	4.28	1	5.0-3000	30	0.999
20	Glutamine	253.33>152.04	4.28	0.5	5.0-3000	105	0.999
21	Methionine	256.39>152.06	5.26	0.5	7.5-3000	44	0.999
22	Orotic acid	263.50>165.062	5.49	0.01	1.0-3000	113	0.998
23	3-Methyl adipic acid	267.114>166.061	5.30	0.1	1.0-3000	2670	0.999
24	N-Acetyl glutamine	296.346>165.061	4.79	0.5	5.0-3000	9	0.999
25	Ascorbic acid	301.05>266.01	6.36	0.01	10.0-3000	1313	0.999
26	3-Nitrotyrosine	333.37>181.03	4.85	0.01	10.0-3000	152	0.999
27	Methylmalonic acid	353.14>180.011	5.24	0.1	10.0-3000	55	0.999
28	Docosatetraenoic acid	439.68>179.035	7.43	0.025	10.0-3000	81	0.999
29	Palmitic acid	363.345>179.132	7.31	0.075	10.0-3000	345	0.999
30	Glycine	182.251>166.060	4.02	0.1	1.0-3000	9.6	0.999
31	Sarcosine	196.273>166.060	4.24	0.01	2.5-3000	105	0.999
32	Lactic acid	196.817>143.802	3.95	0.01	0.1-3000	30	0.999
33	Oxalic acid	196.972>151.042	4.34	0.01	1.0-3000	48	0.997
34	γ-Amino butyric acid	210.300>166.051	4.44	0.05	0.5-3000	50	0.997

35	3-Hydrobutyric acid	211.152>179.077	5.09	0.01	1.0-2000	684	0.999
36	Threonine	226.300>166.014	4.24	0.01	1.0-3000	42	0.998
37	Cysteine	228.34>166.036	5.65	0.25	1.0-3000	5.8	0.999
38	Pyroglutamic acid	236.295>165.996	4.89	2.5	25.0-3000	5	0.999
39	Cis-Aconitic acid	281.288>166.06	4.62	0.05	5.0-1000	119	0.999
40	Citric/Isocitric acid	237.259>166.115	5.08	0.001	5.0-3000	91	0.999
41	Glutaconic acid	237.279>166.06	5.13	0.01	10.0-5000	3280	0.994
42	4-Hydroxy proline	238.966>179.133	4.20	0.001	0.5-3000	89	0.991
43	Asparagine/Glutaric acid	239.299>166.06	4.21	0.5	1.0-3000	17	0.996
44	Ornithine	239.299>209.06	6.28	0.01	10-5000	94	0.997
45	Adipic acid	253.16>166.06	4.21	0.05	5.0-3000	31	0.996
46	Glutamic acid	366.23>304.053	6.81	0.01	7.5-3000	1029	0.992
47	Mevalonic acid	255.11 > 219.09	3.93	0.05	5.0-3000	30	0.994
48	2-Hydroxy glutaric acid	255.142>209.101	3.92	0.01	0.1-3000	17	0.998
49	Dihydroorotic acid	265.29>166.06	5.62	0.01	10.0-3000	774	0.999
50	2,2-Dimethylglutaric acid	267.35>180.07	6.51	0.01	10.0-5000	86	0.998
51	2-Hydroxy octanoic acid	267.39>182.02	5.70	0.01	1.0-10000	431	0.994
52	3-Hydroxy octanoic acid	267.39>250.12	6.51	0.01	5.0-10000	291	0.994
53	Hippuric acid	286.355>166.062	4.66	0.01	5.0-1000	57	0.996
54	Arginine	281>166.06	4.61	0.1	5.0-250	86.7	0.999
55	10-Undecenoic acid	291.46>166.06	5.86	0.05	5.0-3000	63	0.998
56	Tridecanoic acid	321.48>166.06	6.61	0.01	0.5-3000	72	0.999
57	2/3-Hydroxy dodecanoic acid	323.5>166.026	6.45	0.1	5.0-3000	15.9	0.999
58	Myristic acid	335.55>166.06	6.85	0.01	1.0-3000	142	0.996
59	Dodecanedioic acid	337.48>165.96	5.45	0.1	1.0-3000	12	0.999
60	3-Hydroxy myristic acid	351.55>166.06	5.65	0.05	5.0-3000	219	0.992
61	Pentadecanoic acid	349.58>166.06	7.09	0.01	0.5-3000	306	0.997
62	Palmitoleic acid	361.59>166.06	6.98	0.01	1.0-3000	12.9	0.997
63	Heptadecenoic acid	375.58>166.06	7.19	0.1	5.0-3000	19.5	0.997
64	Heptadecanoic acid	377.63>166.06	7.58	0.01	1.0-3000	260	0.997
65	16-Hydroxy palmitic acid	379.6>166.06	6.05	0.05	5.0-3000	89	0.997
66	Linoeliaidic acid	387.63>166.06	7.23	5	5.0-3000	7.9	0.999
67	Oleic acid/ Elaidic acid	389.64>166.06	7.45	0.01	0.5-3000	50	0.999
68	Stearic acid	391.66>166.06	7.82	0.01	10.0-3000	23	0.997
69	9-Cis Retinoic acid	407.58>166.06	6.46	0.01	5.0-3000	10	0.999
70	Eicosapentoic acid	409.63>166.06	7.30	5	5.0-3000	11.7	0.998
71	Arachidonic acid	411.65>165.96	7.17	0.05	5.0-3000	19	0.994
72	Cis-11-Eicosenoic acid	417.69>166.06	7.89	0.1	5.0-3000	12	0.998
73	Docosahexanoic acid	435.67>166.06	6.87	0.05	5.0-3000	36	0.999

74	Heneicosanoic acid	433.74>166.06	8.71	0.1	5.0-3000	25	0.999
75	Erucic acid	445.75>166.06	8.38	0.01	5.0-3000	327	0.999
76	2-Phenyl-2-propyl-succinic acid	450.3>166.06	9.91	0.01	10.0-3000	56	0.998

Supplementary Table S4. %Recovery of 76 CCMs in human body fluids, tissue, and cell lines (in ng/mL).

S. No.	Carboxyl-containing metabolites	Recovery from Matrix (%) calculated as[(mean observed concentration/ spiked concentration) × 100%]					
		Tissue	Plasma	Urine	Serum	Saliva	PANC-1
1	Glyceric acid	95.2	96.8	97.5	98.8	97.3	98.2
2	Glyoxalic acid	96.2	97.8	98.5	99.8	98.3	99.2
3	Propionic acid	97.2	98.8	99.5	100.8	99.3	100.2
4	Pyruvic acid	94.2	94.5	95.8	97.8	96.3	97.2
5	Alanine	93.2	96.5	97.8	96.8	95.3	96.2
6	2-Oxo butyric acid	92.2	99.5	100.8	95.8	94.3	95.2
7	Malonic acid	94.2	99.0	100.3	97.8	96.3	97.2
8	Serine	97.2	96.6	97.9	100.8	99.3	103.2
9	4-Methyl-2-oxo-pentanoic acid	96.8	95.8	99.0	100.3	98.8	99.7
10	Fumaric acid	94.3	95.2	96.6	97.9	98.7	97.2
11	Maleic acid	94.7	97.2	97.5	98.4	97.1	97.6
12	Valine	98.3	100.2	102.0	94.2	101.6	101.3
13	Succinic acid	92.5	99.7	99.9	98.9	99.4	95.4
14	Ureidopropionic acid	94.3	97.2	101.5	101.7	96.3	97.2
15	Oxaloacetic acid	92.5	94.0	94.1	95.0	94.5	95.4
16	Aspartic acid	96.4	100.3	96.0	96.9	98.4	99.4
17	Malic acid	94.3	97.9	100.0	100.9	96.3	97.2
18	Homocysteine	92.5	99.0	94.7	96.0	96.2	95.4
19	α-Keto glutaric acid	92.5	94.1	99.9	96.1	94.6	95.5
20	Glutamine	97.1	98.7	97.9	100.7	99.1	100.0
21	Methionine	95.5	95.2	94.6	99.0	97.5	98.4
22	Orotic acid	100.0	97.2	94.5	103.5	102.0	94.2
23	3-Methyl adipic acid	97.9	100.2	98.4	101.4	99.9	98.9
24	N-Acetyl glutamine	99.5	99.7	96.3	103.0	101.5	101.7
25	Ascorbic acid	92.1	97.2	96.2	95.7	94.1	95.0
26	3-Nitrotyrosine	94.0	95.6	96.3	97.6	96.0	96.9
27	Methylmalonic acid	98.0	99.6	100.2	101.5	100.0	100.9
28	Docosatetraenoic acid	96.0	97.6	98.3	99.6	98.0	98.9
29	Palmitic acid	97.6	99.2	99.8	101.1	104.5	100.5
30	Glycine	98.3	99.9	100.5	101.9	100.3	101.2
31	Sarcosine	96.3	97.9	98.6	99.9	98.4	99.3

32	Lactic acid	93.0	94.6	95.2	96.5	95.0	95.9
33	Oxalic acid	92.6	94.1	94.8	96.1	94.6	95.5
34	γ–Αμινο βυτψριχ αχιδ	97.3	98.9	99.5	100.8	99.3	100.2
35	3-Hydrobutyric acid	92.5	94.0	94.7	96.0	94.5	95.4
36	Threonine	98.6	100.1	100.8	102.1	98.3	101.5
37	Cysteine	95.2	96.8	97.5	98.8	97.2	98.1
38	Pyroglutamic acid	94.8	96.4	97.0	98.4	96.8	97.7
39	Cis-Aconitic acid	94.2	95.8	96.5	97.8	96.2	97.1
40	Citric/Isocitric acid	92.5	94.0	94.7	96.0	94.5	95.4
41	Glutaconic acid	96.0	97.6	98.2	99.5	98.0	98.9
42	4-Hydroxy proline	92.5	94.0	94.7	96.0	94.5	95.4
43	Asparagine/Glutaric acid	96.8	98.3	99.0	100.3	98.8	99.7
44	Ornithine	96.4	98.0	98.7	100.0	98.4	99.4
45	Adipic acid	98.3	99.9	100.6	101.9	100.4	101.3
46	Glutamic acid	95.9	97.5	98.1	99.5	97.9	98.8
47	Mevalonic acid	95.5	97.1	97.7	99.0	97.5	98.4
48	2-Hydroxy glutaric acid	97.0	98.6	99.3	100.6	99.0	100.0
49	Dihydroorotic acid	97.5	99.0	99.7	101.0	99.5	100.4
50	2,2-Dimethylglutaric acid	100.4	101.9	102.6	103.9	102.4	103.3
51	2-Hydroxy octanoic acid	97.3	98.8	99.5	100.8	99.3	100.2
52	3-Hydroxy octanoic acid	98.8	100.4	101.1	102.4	100.8	101.8
53	Hippuric acid	98.8	100.4	101.1	102.4	100.8	101.7
54	Arginine	99.9	101.5	103.1	103.5	101.9	102.8
55	10-Undecenoic acid	97.5	99.1	99.7	101.0	99.5	100.4
56	Tridecanoic acid	97.1	98.6	99.3	100.6	99.1	100.0
57	2/3-Hydroxy dodecanoic acid	98.6	100.2	100.8	102.2	100.6	101.5
58	Myristic acid	101.9	103.4	104.1	104.4	103.9	104.8
59	Dodecanedioic acid	99.5	101.0	101.7	103.0	101.5	102.4
60	3-Hydroxy myristic acid	99.0	100.6	101.3	102.6	101.1	102.0
61	Pentadecanoic acid	98.4	99.9	100.6	101.9	100.4	101.3
62	Palmitoleic acid	98.0	99.6	100.2	101.5	100.0	100.9
63	Heptadecenoic acid	99.9	101.5	102.1	103.5	101.9	102.8
64	Heptadecanoic acid	96.2	97.8	98.5	99.8	98.3	99.2
65	16-Hydroxy palmitic acid	98.6	100.2	100.8	102.2	100.6	101.5
66	Linoeliaidic acid	99.1	100.6	101.3	102.6	101.1	102.0
67	Oleic acid/ Elaidic acid	98.6	100.2	100.9	102.2	100.7	101.6
68	Stearic acid	101.1	99.9	100.6	101.9	100.4	101.3
69	9-Cis Retinoic acid	101.9	94.0	94.7	96.0	94.5	95.4
70	Eicosapentoic acid	98.2	99.8	100.4	101.8	100.2	101.1
71	Arachidonic acid	99.9	101.5	102.1	103.5	103.9	102.8
72	Cis-11-Eicosenoic acid	99.1	100.6	101.3	102.6	104.7	102.0
73	Docosahexanoic acid	99.1	100.6	101.3	102.6	101.1	102.0

74	Heneicosanoic acid	99.9	101.5	102.1	103.5	101.9	102.8
75	Erucic acid	94.0	95.6	96.3	97.6	96.1	97.0
76	2-Phenyl-2-propyl-succinic acid	99.8	101.4	102.0	103.3	101.8	102.7

Tree plot for the Study.

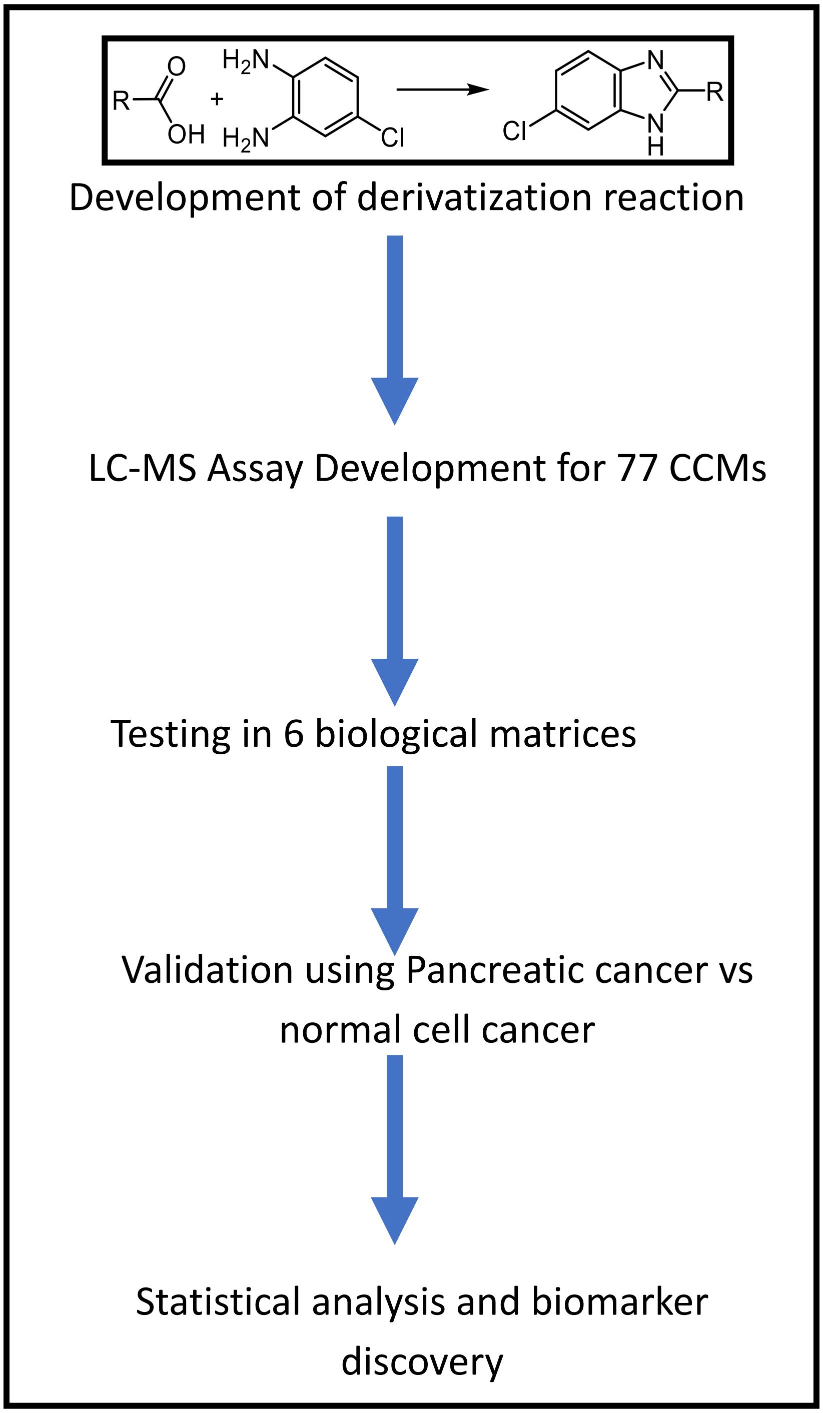

Supplementary Table S5. List of dysregulated CCMs (carbon containing metabolites) in pancreatic cancer cell lines (PANC-1 and PPCL68 individually as well as combined) when compared to normal pancreatic epithelial cells (HPDE and HPNE individually as well as combined).

	cancer vs normal				PANC1 vs HPNE				PANC1 vs HPDE				PPCL68 vs HPNE				PPCL68 vs HPDE			
	FC	log2(FC)	FDR	-log10(FD	FC	log2(FC)	FDR	-log10(FDI	FC	log2(FC)	FDR	-log10(FDI	FC	log2(FC)	FDR	-log10(FDI	FC	log2(FC)	FDR	-log10(FDR)
Arachidonic acid	↑ 8.848	3.1454	9.03E-12	11.044	↑ 6.5316	2.7074	6.15E-06	5.2109	↑ 7.7749	2.9588	8.83E-06	5.0538	↑ 9.7498	3.2854	2.20E-06	5.6573	↑ 11.606	3.5368	4.48E-06	5.3489
Palmitoleic acid	↑ 2.354	1.2354	8.41E-09	8.0753	↑ 2.7821	1.4762	6.15E-06	5.2109	↑ 2.125	1.0875	5.13E-05	4.2902	↑ 2.6548	1.4086	4.43E-05	4.3535	↑ 2.0278	1.0199	0.000372	3.4298
Docosahexanoic acid	↑ 2.636	1.3984	1.06E-08	7.9763	↑ 3.2489	1.6999	3.33E-06	5.477	↑ 2.4903	1.3163	0.000131	3.8831	↑ 2.8264	1.499	4.43E-05	4.3535	↑ 2.1665	1.1153	0.001611	2.793
Oleic acid	↑ 4.119	2.0421	4.15E-08	7.3821	↑ 7.0423	2.816	4.07E-06	5.3905	↑ 4.1668	2.0589	0.000109	3.9631	↑ 4.037	2.0133	8.86E-07	6.0526	↑ 2.3886	1.2562	0.000372	3.4298
Oxaloacetic acid	↑ 2.876	1.524	4.63E-08	7.3344	↑ 2.4067	1.2671	0.000127	3.8956	↑ 4.1614	2.0571	8.34E-06	5.0789	↑ 2.1324	1.0925	0.000351	3.4551	↑ 3.6871	1.8825	1.45E-05	4.8373
Elaidic acid	↑ 4.277	2.0967	7.54E-07	6.1228	↑ 8.3357	3.0593	3.33E-06	5.477	↑ 4.456	2.1558	6.12E-05	4.213	↑ 3.943	1.9793	2.04E-05	4.6914	↑ 2.1078	1.0758	0.002392	2.6212
Heneicosanoic acid	↑ 3.62	1.856	2.15E-06	5.6678	↑ 5.5035	2.4604	6.15E-06	5.2109	↑ 2.1032	1.0726	0.001176	2.9297	↑ 7.5889	2.9239	2.00E-06	5.6992	↑ 2.9001	1.5361	0.000147	3.8326
Glutamine	↑ 2.038	1.0269	1.08E-05	4.9665	↑ 1.7116	0.77531	0.001996	2.6999	↑ 2.8387	1.5052	1.06E-05	4.9761	↑ 1.5546	0.63651	0.019341	1.7135	↑ 2.5783	1.3664	0.000275	3.5602
2-oxo-butyric acid	↑ 2.207	1.1418	3.27E-05	4.4858	↑ 2.1797	1.1241	0.00224	2.6498	↑ 2.9299	1.5509	5.39E-06	5.2683	↑ 1.6685	0.73859	0.077597	1.1102	↑ 2.2428	1.1653	0.005436	2.2647
Adipic acid	↑ 1.785	0.83628	3.27E-05	4.4858	↑ 1.4789	0.56456	0.021064	1.6765	↑ 2.1489	1.1036	0.000716	3.1448	↑ 1.5353	0.61852	0.003681	2.4341	↑ 2.2308	1.1576	0.000232	3.6354
Linoeliaidic acid	↑ 2.717	1.4418	3.27E-05	4.4858	↑ 2.9305	1.5511	0.000355	3.4498	↑ 1.6218	0.69758	0.004771	2.3214	↑ 4.6951	2.2312	0.000341	3.4676	↑ 2.5984	1.3776	0.002886	2.5398
Arginine	↑ 2.404	1.2655	4.93E-05	4.307	↑ 1.6323	0.70689	0.012714	1.8957	↑ 2.9922	1.5812	8.32E-05	4.0801	↑ 2.0834	1.0589	0.019132	1.7182	↑ 3.8192	1.9333	0.000644	3.191
Gluconic acid	↑ 3.386	1.7595	4.93E-05	4.307	↑ 3.2123	1.6836	0.007092	2.1493	↑ 4.4731	2.1613	1.06E-05	4.9761	↑ 2.605	1.3813	0.050999	1.2924	↑ 3.6275	1.859	0.005687	2.2451
Heptadecanoic acid	↑ 3.543	1.8248	5.07E-05	4.2948	↑ 2.5638	1.3583	0.012714	1.8957	↑ 2.4705	1.3048	0.015628	1.8061	↑ 4.6551	2.2188	0.000894	3.0485	↑ 4.4857	2.1653	0.001405	2.8524
9-Cis Retinoic acid	↑ 2.119	1.0833	5.34E-05	4.2722	↑ 2.0455	1.0324	0.007164	2.1449	↑ 2.073	1.0517	0.00483	2.316	↑ 2.1641	1.1138	0.013582	1.867	↑ 2.1933	1.1331	0.009867	2.0058
Alpha-keto glutaric acid	↑ 2.218	1.1492	5.34E-05	4.2722	↑ 1.703	0.76809	0.006192	2.2082	↑ 3.1184	1.6408	0.000459	3.338	↑ 1.7262	0.78757	0.014897	1.8269	↑ 3.1608	1.6603	0.001179	2.9285
Dodecanedioic acid	↑ 1.679	0.74785	5.34E-05	4.2722	↑ 2.3551	1.2358	6.15E-06	5.2109	↑ 1.5127	0.59708	0.008392	2.0761	↑ 1.9387	0.95512	6.29E-05	4.2016	↑ 1.2452	0.3164	0.098915	1.0047
Valine	↑ 1.542	0.62498	6.47E-05	4.189	↑ 1.5948	0.67336	0.007164	2.1449	↑ 1.3145	0.39448	0.013874	1.8578	↑ 1.8185	0.86273	0.002142	2.6691	↑ 1.4988	0.58385	0.002392	2.6212
Mevalonic acid	↓ 0.509	-0.97563	7.35E-05	4.1335	↓ 0.5136	-0.96122	0.007282	2.1378	↓ 0.5905	-0.75996	0.010964	1.96	↓ 0.4372	-1.1936	0.006269	2.2028	↓ 0.5027	-0.99238	0.00927	2.0329
Glutamic acid	↑ 1.752	0.80897	9.52E-05	4.0214	↑ 1.5483	0.63071	0.004351	2.3614	↑ 2.3058	1.2052	0.000292	3.5349	↑ 1.3801	0.46475	0.065899	1.1811	↑ 2.0552	1.0393	0.002886	2.5398
Succinic acid	↑ 1.701	0.76671	9.52E-05	4.0214	↑ 1.5974	0.67573	0.009749	2.0111	↑ 2.1445	1.1006	0.000716	3.1448	↑ 1.3713	0.45557	0.060181	1.2205	↑ 1.841	0.88047	0.003326	2.4781
Homocysteine	↑ 3.664	1.8734	9.76E-05	4.0104	↑ 3.2328	1.6928	0.005799	2.2366	↑ 5.935	2.5692	1.06E-05	4.9761	↑ 2.4271	1.2792	0.11242	0.94917	↑ 4.4559	2.1557	0.013895	1.8571
Alanine	↑ 1.431	0.51708	0.00012475	3.9039	↑ 1.504	0.58876	0.007917	2.1014	↑ 1.4068	0.49247	0.008276	2.0822	↑ 1.4569	0.54293	0.015451	1.811	↑ 1.3629	0.44664	0.018115	1.742
N-Acetyl glutamine	↑ 1.9	0.92617	0.00012475	3.9039	↑ 2.3038	1.204	0.000671	3.1733	↑ 1.5725	0.65304	0.032448	1.4888	↑ 2.3804	1.2512	0.002299	2.6385	↑ 1.6248	0.70022	0.054399	1.2644
Hydroxy Myristic acid	↑ 2.212	1.1454	0.00012962	3.8873	↑ 2.2737	1.185	0.004034	2.3943	↑ 3.1549	1.6576	0.000369	3.433	↑ 1.5326	0.61598	0.11242	0.94917	↑ 2.1266	1.0885	0.009734	2.0117
2/3-Hydroxy Dodecanoic acid	↑ 3.891	1.9603	0.00014392	3.8419	↑ 4.2307	2.0809	0.001138	2.9437	↑ 5.9867	2.5818	3.35E-05	4.4747	↑ 2.4107	1.2695	0.13352	0.87446	↑ 3.4113	1.7703	0.038149	1.4185
Pyruvic acid	↑ 1.393	0.47781	0.00014392	3.8419	↑ 1.3804	0.46507	0.016538	1.7815	↑ 1.3295	0.41091	0.012411	1.9062	↑ 1.4581	0.54413	0.013964	1.855	↑ 1.4044	0.48998	0.00927	2.0329
2-Phenyl-2-propyl-succinic acid	↑ 3.453	1.7878	0.00014625	3.8349	↑ 3.9655	1.9875	0.00224	2.6498	↑ 1.6411	0.71465	0.0982	1.0079	↑ 7.8305	2.9691	0.000351	3.4551	↑ 3.2406	1.6962	0.005687	2.2451
Ornithine	↓ 0.525	-0.9284	0.00019489	3.7102	↓ 0.4972	-1.0081	0.009304	2.0313	↓ 0.5866	-0.76959	0.021922	1.6591	↓ 0.4736	-1.0782	0.012067	1.9184	↓ 0.5588	-0.83968	0.023601	1.6271
Eicosapentoic acid	↑ 2.73	1.449	0.00022661	3.6447	↑ 7.7616	2.9563	4.06E-05	4.3911	↑ 1.9167	0.93862	0.000109	3.9631	↑ 6.0244	2.5908	0.000174	3.7602	↑ 1.4877	0.57308	0.008134	2.0897
Pentadecanoic acid	↑ 1.629	0.70382	0.0002689	3.5704	↑ 2.6033	1.3803	5.28E-06	5.2776	↑ 1.4811	0.56669	0.002094	2.6791	↑ 1.8884	0.91715	2.04E-05	4.6914	↑ 1.0744	0.10351	0.31317	0.50422
Hydroxy Palmitic acid	↑ 3.037	1.6027	0.00029919	3.5241	↑ 3.2578	1.7039	0.009304	2.0313	↑ 4.383	2.1319	0.001677	2.7756	↑ 2.0366	1.0262	0.11086	0.95522	↑ 2.74	1.4542	0.020392	1.6905
3-NitroTyrosine	↑ 2.178	1.1229	0.00031653	3.4996	↑ 2.3902	1.2571	0.005799	2.2366	↑ 1.4348	0.52081	0.14775	0.83047	↑ 3.4158	1.7722	0.000894	3.0485	↑ 2.0504	1.0359	0.010131	1.9943
Threonine	↑ 1.859	0.89431	0.00032551	3.4874	↑ 1.4883	0.57362	0.016212	1.7902	↑ 2.3867	1.255	0.001834	2.7365	↑ 1.5295	0.61304	0.063956	1.1941	↑ 2.4528	1.2944	0.005511	2.2588
Sarcosine	↓ 0.645	-0.63342	0.00040312	3.3946	↓ 0.7259	-0.46212	0.049299	1.3072	↓ 0.6851	-0.54564	0.00272	2.5654	↓ 0.6018	-0.73266	0.027351	1.563	↓ 0.5679	-0.81618	0.008134	2.0897
Asparagine	↓ 0.536	-0.90057	0.00048272	3.3163	↓ 0.5001	-0.99962	0.012714	1.8957	↓ 0.58	-0.78598	0.025451	1.5943	↓ 0.4975	-1.0073	0.021946	1.6586	↓ 0.5769	-0.79363	0.040584	1.3916
Stearic acid	↑ 1.531	0.61449	0.00048272	3.3163	↑ 1.7742	0.82713	0.004354	2.3611	↑ 1.2534	0.32586	0.05747	1.2406	↑ 1.924	0.94408	0.003681	2.4341	↑ 1.3592	0.44281	0.038454	1.4151
Gamma-amino butyric acid	↑ 1.349	0.43145	0.00057463	3.2406	↑ 1.4285	0.51452	0.012148	1.9155	↑ 1.3159	0.39606	0.058643	1.2318	↑ 1.3841	0.46891	0.008177	2.0874	↑ 1.275	0.35045	0.054399	1.2644
Palmitic acid	↑ 1.546	0.6285	0.00066541	3.1769	↑ 2.311	1.2085	0.000186	3.7303	↑ 1.3431	0.42556	0.002098	2.6781	↑ 1.895	0.9222	0.001823	2.7392	↑ 1.1013	0.13926	0.27228	0.56499
Histamine	↑ 1.329	0.40979	0.00096704	3.0146	↑ 1.2705	0.34543	0.023616	1.6268	↑ 1.4721	0.55792	0.001176	2.9297	↑ 1.2045	0.26845	0.21485	0.66786	↑ 1.3957	0.48094	0.026172	1.5822
Cis-11-Eicosenoic acid	↑ 2.546	1.3482	0.0009833	3.0073	↑ 12.713	3.6682	1.81E-06	5.7415	↑ 1.2943	0.37218	0.16134	0.79225	↑ 14.84	3.8914	8.86E-07	6.0526	↑ 1.5108	0.59535	0.037663	1.4241
Myristic acid	↑ 1.356	0.43899	0.0013715	2.8628	↑ 1.4732	0.55892	0.018834	1.7251	↑ 1.3932	0.47836	0.025238	1.5979	↑ 1.316	0.39615	0.053722	1.2698	↑ 1.2445	0.31559	0.069145	1.1602
Heptadecenoic acid	↑ 1.828	0.87035	0.0014779	2.8304	↑ 2.9678	1.5694	0.000113	3.946	↑ 1.9206	0.94157	0.003774	2.4232	↑ 1.6851	0.75287	0.013964	1.855	↑ 1.0905	0.12505	0.77339	0.1116
4-methyl-2-Oxo-pentanoic acid	↑ 1.29	0.36721	0.0030173	2.5204	↑ 1.063	0.088149	0.53864	0.2687	↑ 1.3674	0.45139	0.000716	3.1448	↑ 1.2296	0.2982	0.13352	0.87446	↑ 1.5817	0.66144	0.000372	3.4298
Fumaric acid	↑ 1.266	0.34039	0.0030173	2.5204	↑ 1.1379	0.18642	0.30449	0.51642	↑ 1.2729	0.34812	0.008392	2.0761	↑ 1.26	0.33344	0.10144	0.9938	↑ 1.4095	0.49514	0.001428	2.8452
4-Hydroxy proline	↓ 0.699	-0.51703	0.0031082	2.5075	↓ 0.667	-0.58429	0.022387	1.65	↓ 0.7916	-0.33719	0.13086	0.88318	↓ 0.6206	-0.68818	0.024728	1.6068	↓ 0.7366	-0.44107	0.093744	1.0281
Glyoxalic acid	↑ 1.7	0.76528	0.0035744	2.4468	↑ 2.053	1.0377	0.009438	2.0251	↑ 2.3332	1.2223	0.005788	2.2375	↑ 1.1423	0.1919	0.27604	0.55902	↑ 1.2982	0.37654	0.091028	1.0408
Methyl adipic acid	↑ 1.208	0.27282	0.0037914	2.4212	↑ 1.2894	0.36672	0.013756	1.8615	↑ 1.2284	0.29674	0.052211	1.2822	↑ 1.187	0.24727	0.063956	1.1941	↑ 1.1308	0.17729	0.20539	0.68742
Docosatetraenoic acid	↓ 0.456	-1.1321	0.0042389	2.3728	↓ 0.3859	-1.3739	0.037749	1.4231	↓ 0.8116	-0.3011	0.22754	0.64295	↓ 0.2873	-1.7994	0.016242	1.7894	↓ 0.6043	-0.72664	0.054399	1.2644
Cis-Aconitic acid	↑ 1.745	0.80315	0.010359	1.9847	↓ 0.969	-0.04548	0.64382	0.19123	↑ 5.4082	2.4351	0.000128	3.8923	↑ 1.0886	0.12243	0.71117	0.14802	↑ 6.0757	2.6031	0.000141	3.8505
Ureidopropionic acid	↓ 0.728	-0.45874	0.015418	1.812	↓ 0.6799	-0.55666	0.057692	1.2389	↓ 0.8007	-0.32074	0.23446	0.62992	↓ 0.6656	-0.58726	0.051528	1.288	↓ 0.7839	-0.35134	0.20722	0.68358
Glycine	↑ 1.452	0.53814	0.02261	1.6457	↑ 1.6572	0.72878	0.048303	1.316	↑ 1.8893	0.91783	0.014551	1.8371	↑ 1.0686	0.095734	0.70038	0.15467	↑ 1.2182	0.28479	0.21975	0.65807
Hippuric acid	↓ 0.456	-1.1327	0.029037	1.5371	↓ 0.4255	-1.2328	0.057692	1.2389	↓ 0.4606	-1.1184	0.23446	0.62992	↓ 0.4545	-1.1376	0.092501	1.0339	↓ 0.492	-1.0232	0.26773	0.5723
10-Undecenoic acid	↑ 1.341	0.42355	0.032812	1.484	↑ 1.1799	0.23865	0.5624	0.24996	↑ 1.4048	0.49038	0.16969	0.77034	↑ 1.2878	0.36492	0.14427	0.84082	↑ 1.5333	0.61665	0.002392	2.6212
Lactic acid	↓ 0.8	-0.3211	0.033188	1.479	↓ 0.915	-0.12823	0.51258	0.29023	↓ 0.6318	-0.66236	0.000658	3.1815	↑ 1.0446	0.062993	0.70038	0.15467	↓ 0.7214	-0.47114	0.003326	2.4781
Oxalic acid	↑ 1.358	0.44153	0.04644	1.3331	↑ 1.5508	0.63303	0.005299	2.2758	↑ 1.631	0.70573	0.003774	2.4232	↑ 1.0985	0.13558	0.85935	0.065832	↑ 1.1553	0.20827	0.66952	0.17424
Erucic acid	↑ 1.482	0.56729	0.087516	1.0579	↑ 4.4684	2.1598	0.003432	2.4645	↓ 0.5985	-0.74061	0.025451	1.5943	↑ 8.0762	3.0137	0.000341	3.4676	↑ 1.0817	0.11331	0.95831	0.018493
Ascorbic acid	↑ 1.189	0.25007	0.091005	1.0409	↑ 1.3726	0.45689	0.015175	1.8189	↑ 1.3635	0.44735	0.010521	1.9779	↑ 1.0138	0.019835	0.92049	0.035982	↑ 1.0072	0.010288	0.98689	0.00573
Citric acid/isocitric acid	↑ 1.194	0.25564	0.091005	1.0409	↑ 2.0655	1.0465	5.28E-06	5.2776	↑ 1.0109	0.015577	0.89068	0.050276	↑ 1.5678	0.64875	0.000245	3.6117	↓ 0.7673	-0.38217	0.016386	1.7855
Propionic acid	↑ 1.245	0.31586	0.10986	0.95915	↑ 1.3872	0.47218	0.049414	1.3061	↑ 1.5984	0.67664	0.013874	1.8578	↓ 0.9378	-0.09262	0.59965	0.2221	↑ 1.0806	0.11185	0.66952	0.17424
Glutaconic acid	↑ 1.167	0.22236	0.11646	0.93383	↑ 1.9691	0.97755	1.57E-05	4.8031	↓ 0.988	-0.01748	0.90903	0.041421	↑ 1.5228	0.60669	0.002032	2.6921	↓ 0.764	-0.38834	0.018983	1.7216

Supplementary Table S6. List of dysregulated CCMs (carbon containing metabolites) in the media isolated from pancreatic cancer cell lines (PANC-1 and PPCL68) and normal pancreatic epithelial cells (HPDE and HPNE) when compared to the control media used for the growth of each mentioned cell line, respectively.

	PANC1 media vs IMEM media				PPCL68 media vs Advanced media				HPNE media vs KSFM media				HPDE media vs KSFM media			
	**FC**	**log2(FC)**	**FDR**	**-log10(FDR)**	**FC**	**log2(FC)**	**FDR**	**-log10(FDR)**	**FC**	**log2(FC)**	**FDR**	**-log10(FDR)**	**FC**	**log2(FC)**	**FDR**	**-log10(FDR)**
Gamma-amino butyric acid	↑ 25.183	4.6544	5.71E-08	7.2433	↑ 23.73	4.5689	4.68E-10	9.3298	↑ 108.7	6.7637	4.80E-10	9.319	↑ 91.88	6.5216	5.99E-08	7.2227
Pyruvic acid	↑ 4.6663	2.2223	4.05E-06	5.3926	↑ 10.38	3.3756	2.29E-08	7.6409	↑ 10.95	3.4524	7.66E-08	7.1157	↑ 11.74	3.5538	8.92E-06	5.0496
Alanine	↑ 4.3167	2.1099	6.57E-06	5.1826	↓ 0.793	-0.33469	0.47658	0.32186	↑ 11	3.4587	2.11E-07	6.6759	↑ 12.33	3.6244	3.40E-05	4.4689
Ornithine	↑ 1.5934	0.67214	0.072642	1.1388	↑ 1.116	0.1585	0.61387	0.21193	↑ 2.388	1.2555	0.00048162	3.3173	↑ 1.924	0.94394	0.15755	0.80258
Oxalic acid	↓ 0.876	-0.19095	0.80591	0.093711	↓ 0.258	-1.9519	3.54E-05	4.451	↑ 2.361	1.2396	0.0029331	2.5327	↑ 1.402	0.48744	0.71846	0.1436
Oxaloacetic acid	↑ 1.8359	0.87646	0.003023	2.5195	↓ 0.975	-0.036322	0.79663	0.098742	↑ 2.33	1.2203	0.0029331	2.5327	↑ 1.866	0.89955	0.14389	0.84198
Malonic acid	↑ 1.5462	0.62877	0.15917	0.79815	↑ 1.525	0.60868	0.15795	0.80149	↑ 2.811	1.4909	0.0045586	2.3412	↑ 2.007	1.0049	0.1395	0.85542
4-Hydroxy proline	↑ 1.7393	0.79849	0.013532	1.8686	↓ 0.98	-0.029092	0.89044	0.050393	↑ 2.208	1.1427	0.0045586	2.3412	↑ 2.02	1.0141	0.14792	0.82998
Ureidopropionic acid	↑ 1.7659	0.82042	0.032511	1.488	↓ 0.97	-0.043488	0.76912	0.11401	↑ 2.21	1.1442	0.0054824	2.261	↑ 1.985	0.98879	0.14389	0.84198
3-Hydrobutyric acid	↑ 1.514	0.59835	0.20442	0.68948	↑ 1.342	0.42463	0.17476	0.75756	↑ 2.751	1.4597	0.005542	2.2563	↑ 2.054	1.0382	0.1395	0.85542
Asparagine	↑ 1.866	0.89992	0.002082	2.6814	↓ 0.794	-0.33295	0.60437	0.2187	↑ 2.079	1.0558	0.013878	1.8577	↑ 1.664	0.73447	0.26209	0.58155
Propionic acid	↑ 1.4222	0.50814	0.1803	0.74401	↑ 1.159	0.21222	0.76912	0.11401	↑ 1.491	0.5765	0.017544	1.7559	↑ 1.844	0.88303	0.1395	0.85542
4-methyl-2-Oxo-pentanoic acid	↓ 0.8766	-0.18998	0.68951	0.16146	↓ 0.565	-0.82411	0.17476	0.75756	↓ 0.572	-0.80551	0.025733	1.5895	↓ 0.571	-0.8082	0.1395	0.85542
Myristic acid	↑ 1.0351	0.049809	0.94238	0.025775	↑ 1.201	0.26467	0.24221	0.6158	↑ 1.529	0.61273	0.038979	1.4092	↑ 1.319	0.39958	0.26209	0.58155
Threonine	↓ 0.899	-0.15366	0.8823	0.054384	↓ 0.954	-0.067305	0.79663	0.098742	↑ 3.009	1.5892	0.049564	1.3048	↑ 2.952	1.5615	0.41426	0.38273
4-Pyridoxic acid	↓ 0.9441	-0.08304	0.83856	0.076464	↑ 11.92	3.575	1.18E-06	5.9293	↑ 2.398	1.2617	0.12836	0.89155	↑ 1.138	0.18688	0.91616	0.03803
Heneicosanoic acid	↑ 1.1634	0.21836	0.83856	0.076464	↑ 2.473	1.3063	0.0082731	2.0823	↑ 1.886	0.91526	0.37426	0.42682	↑ 1.942	0.95738	0.28794	0.54069
Docosahexanoic acid	↓ 0.7865	-0.34646	0.80591	0.093711	↑ 2.217	1.1483	0.015069	1.8219	↑ 1.114	0.15542	0.6685	0.1749	↓ 0.864	-0.21155	0.90625	0.042753
3-Hydroxy Octanoic acid	↓ 0.7941	-0.33263	0.46886	0.32895	↑ 18.67	4.2224	8.64E-10	9.0635	↑ 1.026	0.037329	0.92561	0.033572	↓ 0.52	-0.9439	0.1043	0.9817
