# Supplementary material for: An Optimized Method for LC–MS-Based Quantification of Endogenous Organic Acids: Metabolic Perturbations in Pancreatic Cancer
Source: Int J Mol Sci. 2024 May 28;25(11):5901. doi: 10.3390/ijms25115901 (PMC11172734; doi:10.3390/ijms25115901)
Supplement: Supplementary file 1 [file ijms-25-05901-s001.zip › Jain et al_Bulleted SOP.pdf]

1. Protocol for Derivatization of Carboxylic acid containing Metabolites (CCMs)

A. Derivatization of standards/internal standards (IS)

Reagents and Equipment:

- 2 mL Glass Tubes (Thermo Scientific™ 60180A-SV9-2P)
- 1 mL Pipette and Tips
- 200 µL Pipette and Tips
- 4-Chloro-o-phenylenediamine (4-Cl-*o*-PD, 108871-Sigma-Aldrich)
- Standard metabolite or labelled metabolite (Sigma-Aldrich)
- Hydrochloric Acid (A466-250, Optima™, Fisher Chemical™)
- Ethyl Acetate, HPLC Grade (02-002-830 J.T.Baker™)
- 1.5 mL Eppendorf tube
- LC-MS Grade Methanol (A456-4, Optima™, Fisher Chemical™)
- LC-MS Grade Water (W6-4, Optima™, Fisher Chemical™)
- LC-MS Vials
- Vortex Mixer
- 4 °C Refrigerator
- -20 °C Freezer
- 2 mL Tube Centrifuge
- Sonicator
- Heat bath
- Speed Vacuum
- Nitrogen Evaporator
- -80 °C Refrigerator

Procedure:

1. Weigh 1 mmol of the desired standard (listed in Supplementary Information Fig. S1) and internal standards (Main text, Section 2.6) to be subjected to derivatization in 2 mL glass vial.
2. Correspondingly weigh 1.1 mmol of 4-Cl-*o*-PD for monocarboxylic acids (CAs) and 2.1 mmol for dicarboxylic acids (di-CAs) for derivatization and added to the same glass vial having standard.
3. Add 200 µl LC-MS grade methanol having 5N HCl.
4. The reaction mixture was vortexed for 30 seconds, followed by sonication for 30 min at ambient temperature.
5. Temperature of reaction mixture was elevated to 60 °C followed by sonication for 12 h.
6. The reaction was quenched by drying the reaction mixture at 60 °C under vacuum or by nitrogen flushing.
7. Reaction mixture was re-suspended in 400 µL of ethyl acetate, vortexed well and centrifuged at 13,000g for 20 min at 4 °C to get a clear supernatant.
8. Supernatant was transferred to 2 mL glass tube.
9. This supernatant was dried either by N2 flushing or speed vacuum.
10. The dried derivatized product was stored at -80 °C until further use (pause-point: safe for 2 months).

11. The dried derivatized product was reconstituted as 1.0 mg/mL in methanol and used as further working solution of all standards and IS.

B. Enrichment and Extraction of derivatized CCM standards/internal standards for MRM development

Reagents and Equipment:

- C18 Reversed phase silica gel (Millipore Sigma, 60756)
- Macro Spin Column Silica C18 (Harvard Apparatus, 74-4101)
- LC-MS Grade Acetonitrile (A955-4)
- LC-MS Grade Water (W6-4)
- Desiccator
- Water bath
- 2 mL Tube Centrifuge
- 1.5 mL Eppendorf tube

Procedure:

1. 1 mg/mL working solution of derivatized standards and internal standards from Step A were further enriched by column chromatography for the purpose of MRM development. 10 µL of each working solution were mixed, individually, with C18 reversed phase silica gel (50 µg) and dried in a desiccator under vacuum or at 50 °C in a water bath for 1h, to get free flowing slurry.
2. Slurry was loaded on MacroSpin column/cartridge and eluted sequentially while increasing ACN percentage in water.
3. Elution was performed by centrifugation (800 rpm for 1.5 min at controlled temp: 25 °C). Four fractions (Fr) each 1 mL, Fr-1:100% water, Fr-2: 5% ACN in water, Fr-3: 10% ACN in water and Fr-4: 70% ACN in water, were collected.
4. The Fr-4 (1 mL) having concentration of 10 µg/mL was used as further working solution for MRM development.

Note: Under conditions we used, (C-18 cartridge, silica amount, elution speed, centrifugation etc.), all derivatized CAs eluted completely in Fr-4. This step can be adjusted according to laboratory conditions, and if required, higher ACN content and/or more volume of a particular ACN/water mixture can be used for complete elution of CA derivatives and final volume can be adjusted after drying and reconstituted to 1 mL of ACN:water (1:1) for MRM development.

C. Extraction and Enrichment of derivatized CCM standards for calibration curve

Reagents and Equipment:

- C18 Reversed phase silica gel (Millipore Sigma, 60756)
- Macro Spin Column Silica C18 (Harvard Apparatus, 74-4101)
- LC-MS Grade Acetonitrile (A955-4)
- LC-MS Grade Water (W6-4)
- Desiccator
- Water bath

- 2 mL Tube Centrifuge
- 1.5 mL Eppendorf tube
- Mass spec glass vial
- Speed Vacuum
- Nitrogen Evaporator

Procedure:

1. 1 mg/mL working solution of derivatized standards from Step A were further enriched by column chromatography for the purpose of calibration curve. 10 µL of each working solution of individual standards was mixed and dried either by N_2 flushing or speed vacuum followed by dissolving in 10 µL of methanol.
2. The solution was mixed with C18 reversed phase silica gel (50 µg) and dried in a desiccator under vacuum or at 50 °C in a water bath for 1h, to get free flowing slurry.
3. Slurry was loaded on MacroSpin column/cartridge and eluted sequentially while increasing ACN percentage in water.
4. Elution was performed by centrifugation (800 rpm for 1.5 min at controlled temp: 25 °C). Four fractions (Fr) each 1 mL, Fr-1:100% water, Fr-2: 5% ACN in water, Fr-3: 10% ACN in water and Fr-4: 70% ACN in water, were collected.
5. The Fr-4 (1mL) having concentration of 10 µg/mL was dried either by N_2 flushing or speed vacuum and dissolved in 1mL of ACN:water (1:1) containing derivatized internal standard (100 ng/mL).
6. The obtained stock solution (10 µg/mL) was diluted serially and transferred to mass spec vials for LC-MS analysis to generate calibration curves. Calibration curve range of the metabolites is mentioned in the Main text Table 1.
   Note: The individually enriched and extracted metabolites in step B can be mixed together and dried either by N_2 flushing or speed vacuum followed by dissolving in 1mL of ACN:water (1:1) containing derivatized internals (100 ng/mL) to use as stock solution for calibration curve.

D. Derivatization of biological samples

Reagents and Equipment:

- 2 mL Glass Tubes (Thermo Scientific™ 60180A-SV9-2P)
- 4-Chloro-*o*-phenylenediamine (4-Cl-*o*-PD, 108871-Sigma-Aldrich)
- Standard metabolite or labelled metabolite (Sigma-Aldrich)
- Hydrochloric Acid (A466-250, Optima™, Fisher Chemical™)
- Ethyl Acetate, HPLC Grade (02-002-830 J.T.Baker™)
- LC-MS Grade Methanol (A456-4, Optima™, Fisher Chemical™)
- LC-MS Grade Water (W6-4, Optima™, Fisher Chemical™)
- 2 µL Plasma/NIST Plasma/Serum samples
- 5 µL Urine/Saliva Samples
- 10 mg Tissue sample
- 50K Cells
- 2 mL Tube Centrifuge

- 1 mL Pipette and Tips
- 200 µL Pipette and Tips
- Sonicator
- Heat bath
- Speed Vacuum
- Nitrogen Evaporator
- -80 °C Refrigerator
- 1.5 mL Eppendorf tube
- LC-MS Vials
- Vortex Mixer
- 4 °C Refrigerator
- -20 °C Freezer

Procedure:

1. Thaw biological samples (plasma, serum, saliva, urine, NIST Plasma, tissue, cell pellets) at 4 °C.
2. Aliquot the biological sample as per their mentioned volume (2 µL- plasma, 2 µL- serum, 2 µL- NIST Plasma, 5 µL- saliva, 5 µL- urine, 10 mg- tissue, 50K cells) for the analysis in safe-lock 1.5 mL Eppendorf tubes or 2.0 mL small glass vial.
3. A 2.0 mg/mL solution of 4-Cl-*o*-PD in methanol was prepared as a derivatizing reagent.
4. 50 µL of the derivatizing reagent solution was directly added to the aliquoted biological samples followed by 100 µl of methanol and 5 µl of 5N HCl.
5. Tissue samples used for the analysis were homogenized with 100 µl of methanol for 1 min followed by addition of 50 µL of the derivatizing reagent solution and 5 µl of 5N HCl.
6. The reaction mixture was vortexed for 2-5 minutes, followed by sonication for 30 min at ambient temperature.
7. Temperature of reaction mixture was elevated to 60 °C and sonicated for another 12 h.
8. The reaction was quenched by drying the reaction mixture in speed vacuum at 60 ^{0}C under vacuum or by nitrogen flushing.
9. Reaction mixture was re-suspended in 400 µL of ethyl acetate, vortexed well and centrifuged at 13,000g for 20 min at 4 °C to get a clear supernatant.
10. Supernatant was transferred to 2 mL glass tube.
11. The supernatant was dried either by N2 flushing or speed vacuum.
12. The dried reaction mass containing derivatized CCMs from biological matrices were stored at -80 °C until further use (pause-point: safe for 2 months).
13. The dried reaction product was reconstituted in 100 µL of methanol and used as further working solution.

E. Extraction and Enrichment of derivatized CCMs from biological samples for analysis
Reagents and Equipment:

- C18 Reversed phase silica gel (Millipore Sigma, 60756)
- Macro Spin Column Silica C18 (Harvard Apparatus, 74-4101)
- LC-MS Grade Acetonitrile (A955-4)
- LC-MS Grade Water (W6-4)

- Desiccator
- Water bath
- 2 mL Tube Centrifuge
- 1.5 mL Eppendorf tube
- Mass spec glass vial
- Speed Vacuum
- Nitrogen Evaporator

Procedure:

1. The obtained crude reaction masses containing derivatized CCMs from all the biological matrices in Step D were further enriched by column chromatography for the purpose of analysis. 10 µL of the working solution from each biological sample were individually mixed with C18 reversed phase silica gel (50 µg) and dried in a desiccator under vacuum or at 50 °C in a water bath for 1h, to get free flowing slurry.
2. Slurry was loaded on MacroSpin column/cartridge and eluted sequentially while increasing ACN percentage in water.
3. Elution was performed by centrifugation (800 rpm for 1.5 min at controlled temp: 25 °C). Four fractions (Fr) each 1 mL, Fr-1:100% water, Fr-2: 5% ACN in water, Fr-3: 10% ACN in water and Fr-4: 70% ACN in water, were collected.
4. The Fr-4 (1mL) was dried either by N_2 flushing or speed vacuum followed by dissolving in 400 µL of ACN:water (8:2) containing derivatized internal standards (100 ng/mL).
5. The above prepared solution was transferred to mass spec vials for LC-MS analysis.

2. Protocol for the Analysis of Derivatized Carboxylic containing Metabolites using Xevo-TQS

Notes:

Reagents and Equipment:

- Xevo-TQS coupled with Acquity UPLC
- Prepared LC-MS standards and biological samples
- LC-MS Grade Water (W6-4)
- LC-MS Grade Acetonitrile (A955-4)
- Acquity UPLC BEH C18 Column, 130Å, 1.7 µm, 2.1 mm X 100 mm (Waters™ 186002352)
- LC-MS Grade formic acid (A117-50)

Procedure:

- This method was designed to resolve derivatized CCMs using an Acquity UPLC system connected to an ESI (+) mode coupled with a triple quadrupole mass spectrometer (Xevo-TQS, Waters Corporation, MA, USA) to facilitate accurate quantitation.
- 5 µL of the prepared sample was injected onto the Acquity UPLC BEH C18 Column, 130Å, 1.7 µm, 2.1 mm X 100 mm kept at 35 °C online with Xevo-TQ-S operated in the multiple reaction monitoring (MRM) mode.
- The binary gradient consisted of water with 0.2% formic acid (solvent A) and acetonitrile with 0.2% formic acid (solvent B) at a flow rate of 0.4 mL/min. The gradient was set as follows:
  a. A gradient elution was used over 15 min with a flow rate of 0.4 mL/min:
     - Initial: 100% A
     - 2-8 min 100-0% A
     - 8-12 min 0% A
     - 12-14 min 0-100% A
     - 14-15 min 100% A
- The tune page parameters were as follows:
  a. Capillary voltage: 3.0 kV
  b. Source temperature: 150 °C
  c. Desolvation temperature: 500 °C
  d. Cone gas flow: 150 L/h
  e. Desolvation gas flow: 900 L/h
